# Synthesis of Saturated N‐Heterocycles via a Catalytic Hydrogenation Cascade

**DOI:** 10.1002/adsc.202200601

**Published:** 2022-06-23

**Authors:** Tobias Wagener, Marco Pierau, Arne Heusler, Frank Glorius

**Affiliations:** ^1^ Westfälische Wilhelms-Universität Münster Organisch-Chemisches Institut Corrensstraße 40 48149 Münster Germany

**Keywords:** Domino reactions, Heterogeneous catalysis, Hydrogenation, Nitrogen heterocycles, Rhodium

## Abstract

Saturated N‐heterocycles are prominent motifs found in various natural products and pharmaceuticals. Despite the increasing interest in this class of compounds, the synthesis of saturated bicyclic azacycles requires tedious multi‐step syntheses. Herein, we present a one‐pot protocol for the synthesis of octahydroindoles, decahydroquinolines, and octahydroindolizines through a cascade reaction.

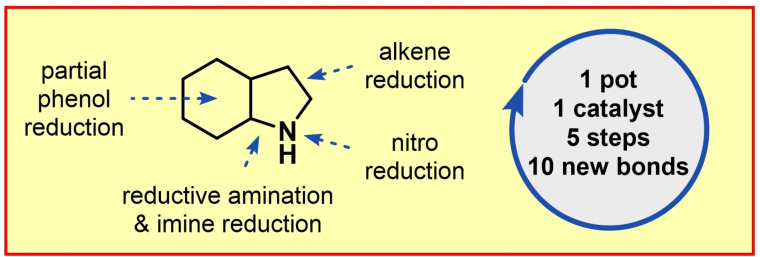

N‐Heterocycles are key structures of natural products, pharmaceuticals, and agrochemical compounds.^[1]^ In case of monocyclic N‐heterocycles, both aromatic and saturated rings are among the most frequently found moieties in small molecule drugs approved by the FDA.^[2]^ However, saturated bicyclic N‐heterocycles occur significantly less than their aromatic counterparts in FDA‐approved drugs, although the saturated motifs are prominent in natural products.^[3]^ This shortcoming might be rationalized by the limited access, since tedious, multi‐step syntheses are required for bicyclic, saturated N‐heterocycles.^[4]^

For example, strategies for accessing octahydroindoles include three general synthetic operations: A cyclization step of a monocyclic precursor, the hydrogenation of an aromatic system and the adjustment of the final N‐oxidation state (Scheme 1). Therefore, the two main approaches for synthesizing octahydroindoles are the cyclization of complex, saturated precursors and the hydrogenation of indoles. While the synthesis of aromatic indoles^[5]^ by ring‐closing of suitable precursors has been intensively investigated, the hydrogenation of indoles often requires harsh conditions such as high hydrogen pressure or toxic solvents.^[6]^ Likewise, the cyclization of saturated, more‐complex building blocks demands multi‐step syntheses and is often not broadly applicable.^[7]^

**Scheme 1:**
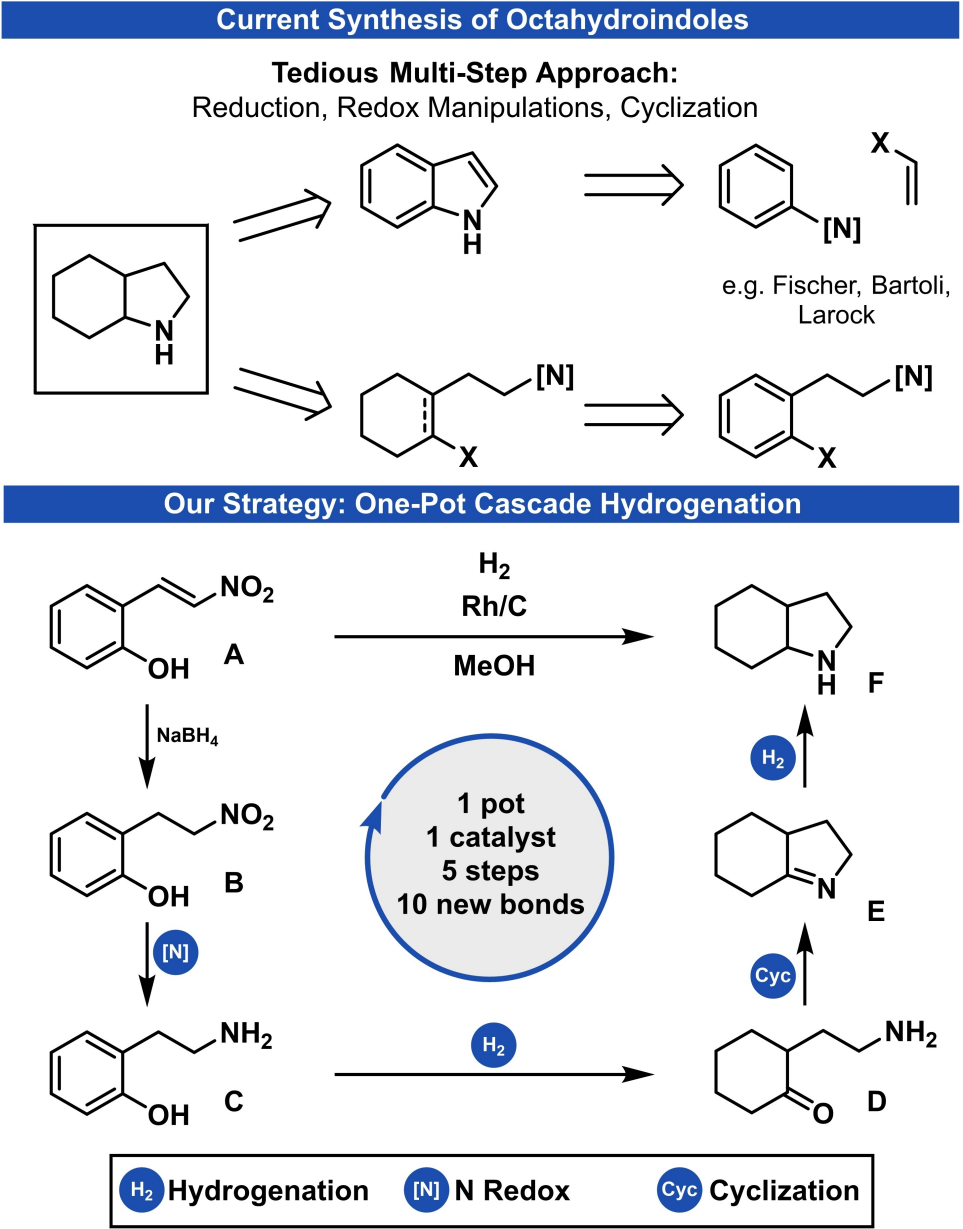
General synthetic steps in current strategies for the synthesis of octahydroindoles and this work.

Hence, our aim was to develop a more efficient protocol for the synthesis of saturated N‐heterocycles by combining all necessary steps in a single synthetic operation. We envisioned arene hydrogenation as a powerful methodology for the formation of complex, saturated scaffolds.^[8]^ Based on our previous progress in combining (hetero)arene hydrogenation with further synthetic manipulations,^[9]^ we were seeking for a new cascade approach to synthesize saturated N‐heterocycles.^[10]^

We identified the intramolecular reductive amination of a cyclohexanone derivative, as an intermediate of a partial phenol hydrogenation,^[11]^ as a promising strategy.^[12]^ After further retro‐synthetic planning, we found 2‐(2‐nitrovinyl)phenols to be suitable starting materials for our strategy. These substrates are easily accessible in a single reaction step from commercially available salicylic aldehydes through a Henry‐reaction.

Our new cascade strategy combines multiple reductions, the necessary adjustment of the final N‐oxidation state from a highly oxidized precursor, and a cyclization step (Scheme 1). Within this approach, the 2‐nitrovinyl substituent of **A** is reduced to the 2‐aminoethyl substituent **C** in a stepwise manner. The partial reduction^[13]^ of **C** yields cyclohexanone intermediate **D**, which undergoes an intramolecular reductive‐amination and thereby interrupts a full arene hydrogenation. Eventually, imine intermediate **E** is reduced to give the desired octahydroindole **F**. The overall desired reaction sequence implements the addition of seven equivalents of molecular hydrogen with three equivalents of water as the only by‐product and forges ten new σ‐bonds in the final product.

During optimization of the reaction conditions, we found rhodium on carbon as the most efficient catalyst for our transformation and methanol as solvent of choice (see Supporting Information for further details). Depending on the reaction conditions, we observed the formation of dimers and trimers as an undesired pathway for side‐products. In addition to an intermolecular reductive amination, the reduction of the vinyl‐nitro group plays a crucial role during our reaction sequence and comprises the potential for the formation of dimeric species.^[14]^ To our delight, we discovered that a prereduction of 2‐(2‐nitrovinyl)phenol with NaBH_4_ selectively forms 2‐(2‐ nitroethyl)phenol and thereby significantly increases the yield of the desired octahydroindole in the overall process. To summarize the final reaction procedure, 2‐(2‐nitrovinyl)phenol is reduced with one equivalent of NaBH_4_ in methanol within 30 minutes, followed by the addition of rhodium on carbon to perform the remaining reaction steps in the presence of 5 to 10 bar hydrogen gas in the same pot.

With the optimized reaction conditions in hand, we investigated the reaction scope for the synthesis of octahydroindoles (Figure 1). For the ease of purification, octahydroindole products were protected by the addition of benzyl chloroformate to the crude reaction mixture after the hydrogenation. Cbz‐protected octahydroindole (**1**) was obtained in 79% yield with >95:5 d.r. as the all‐*cis* product. The 2‐ethyl substituent of octahydroindole **2** resulted in a slightly decreased diastereoselectivity while maintaining a high reaction yield. In contrast, a series of 3‐substituted octahydroindoles **3**–**6** were isolated with a decrease in diastereoselectivity with three isomers being observed. 7‐Methylated octahydroindole **7** was isolated in 39% yield with all four possible diastereoisomers being detected. In the case of 6‐methylated octahydroindole **8** only two diastereoisomers in an almost one to one ratio were isolated in good yield. 5‐Methyl substituted octahydroindole **9** was isolated in good yield and 85:15 diastereoselectivity. Interestingly, a mixture of THF and water increased the yields of octahydroindoles **11** and **12** while no enhancement could be achieved for substrates with less steric demand. Further, multi‐substituted octahydroindole **13** was isolated in high diastereoselectivity but diminished yield. Octahydroindoles with various functional groups **14**–**16** were isolated, proving the tolerance for reductive‐labile esters or desirable fluorinated substituents.^[15]^

**Figure 1:**
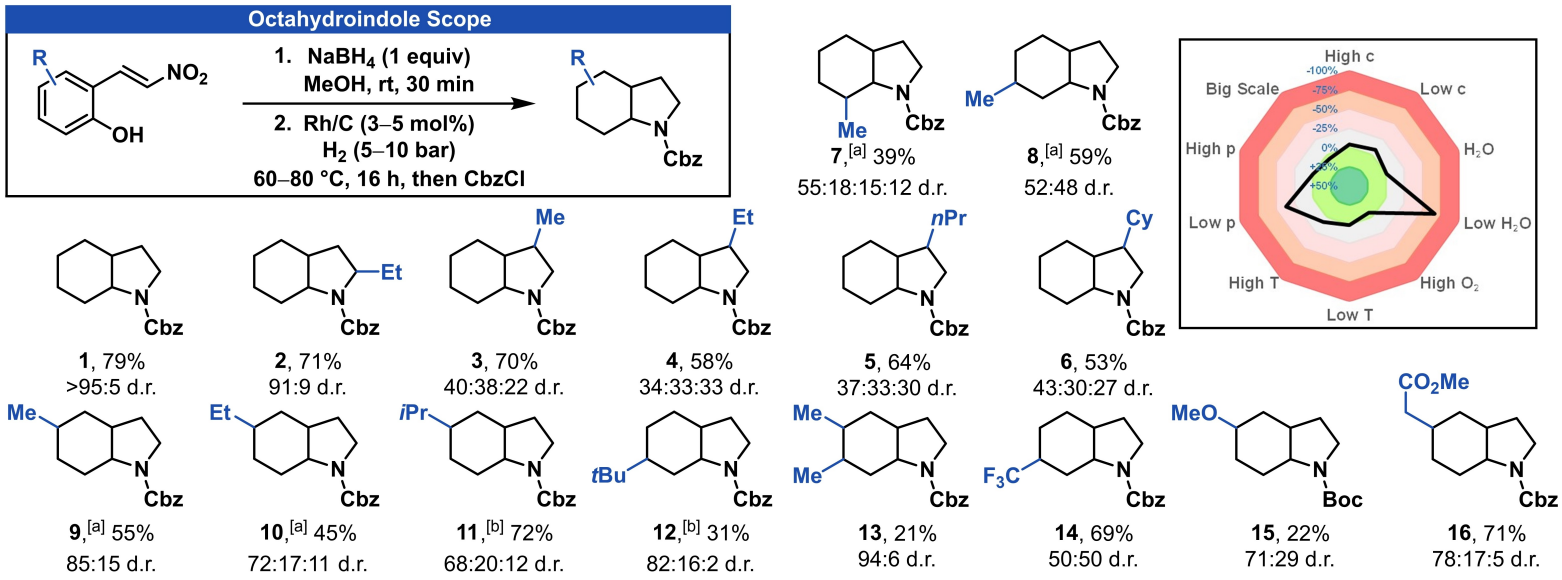
Reaction scope for the synthesis of octahydroindoles. See supporting information for experimental details. Diastereoselectivities were determined by GC‐MS and represent only the observed isomers. [a] 1.5 equiv. aq. NaOH (2 m) was added. [b] The reaction was done in THF:H_2_O (1:1).

After investigation of the octahydroindole scope, we were interested if we could extend our procedure to the synthesis of other saturated N‐heterocycles. By replacing the nitro‐group of our starting materials with a nitrile we were aiming for the construction of a new six‐membered ring to give decahydroquinoline products.

The required 3‐(2‐hydroxyphenyl)acrylonitrile starting materials are easily accessible and were synthesized from salicylic aldehydes in Wittig‐reactions. Applying analogous reaction conditions, Cbz‐protected decahydroquinoline **17** was isolated in 35% yield and 55:45 d.r. A series of substituted decahydroquinolines **18**–**23** were isolated in moderate yields and varying diastereoselectivities (Figure 2).

**Figure 2:**
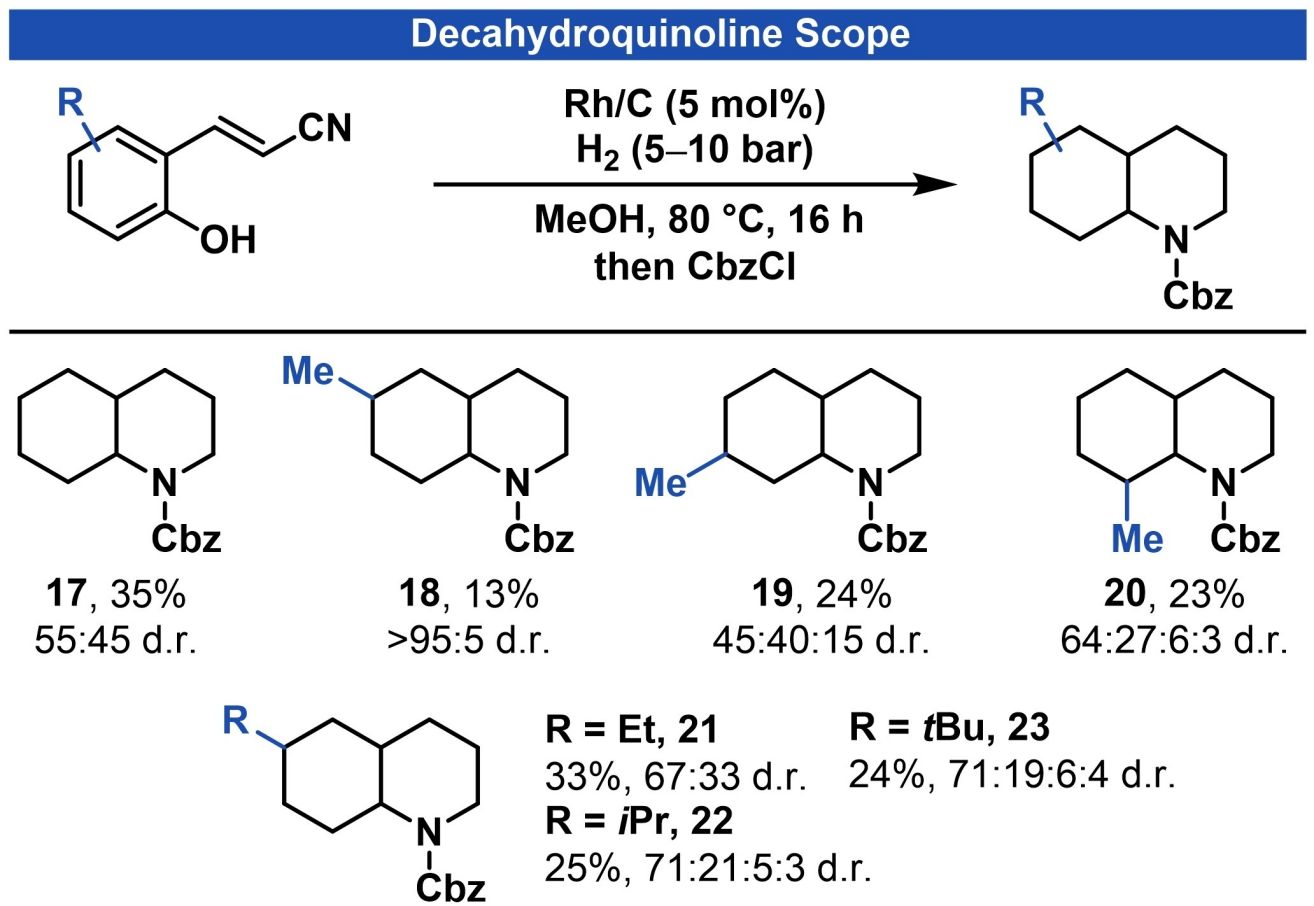
Reaction scope for the synthesis of decahydroquinolines. See supporting information for experimental details. Diastereoselectivities were determined by GC‐MS and represent only the observed isomers.

In addition, we performed a reaction‐condition‐based sensitivity assessment for our synthesis of octahydroindoles (Figure 1).^[16]^ Small deviations in temperature and concentration as well as additional water, higher pressure or oxygen did not have a significant impact on the reaction yield. However, decreased product formation was observed for low hydrogen pressure (3 bar) and water‐free conditions. It should be noted, that all reactions in this study were carried out in methanol of puriss. p.a. quality without further drying.

Intrigued by these results, we were wondering if our strategy could be further advanced to the synthesis of saturated, tertiary N‐heterocycles carrying a bridgehead nitrogen.

The intramolecular cyclization of a piperidine with a propanone carbonyl group^[17]^ was identified to potentially give access to the valuable octahydroindolizine motif.^[18]^ For this purpose, we investigated the hydrogenation of pyridine **24** to give 3‐methyloctahydroindolizine (**25**) (Scheme 2).

**Scheme 2:**
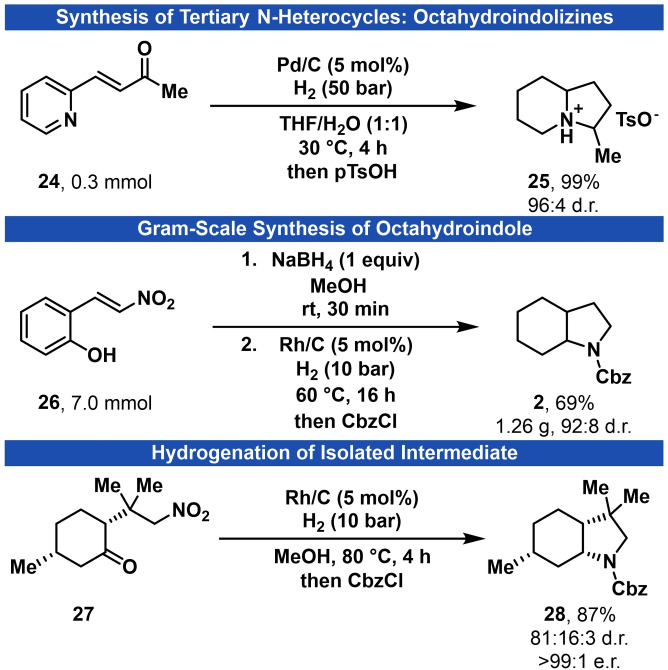
Extension of our strategy for the synthesis of octahydroindolizine **25**, gram‐scale synthesis of octahydroindole 2 and hydrogenation of model intermediate **27** to give enantioenriched octahydroindole **28**.

Crucial for this reaction pathway is the efficient hydrogenation of the aromatic pyridine core while tolerating the carbonyl group. An irreversible reduction of the carbonyl to the corresponding alcohol would prohibit the formation of the desired indolizidine. After a short screen of reaction conditions, we found that palladium on carbon in a mixture of THF and water yields **25** in 99% yield and 96:4 diastereomeric ratio. Moreover, we carried out a cascade hydrogenation of phenol **26** on gram‐scale and isolated octahydroindole **2** in 69% yield, showing the applicability of our approach for large scale reactions (Scheme 2).

After investigation of the synthetic applicability of our new approach, we became interested in the mechanistic pathway and the origin of diastereoselectivity of our octahydroindole synthesis. While the *cis*‐isomer of a saturated (bi)cycle is generally favored by a heterogeneous arene hydrogenation,^[19]^ our approach involves intermediates **I** and **II** with the potential for tautomerization and thereby influencing the stereochemical outcome (Figure 3).

**Figure 3:**
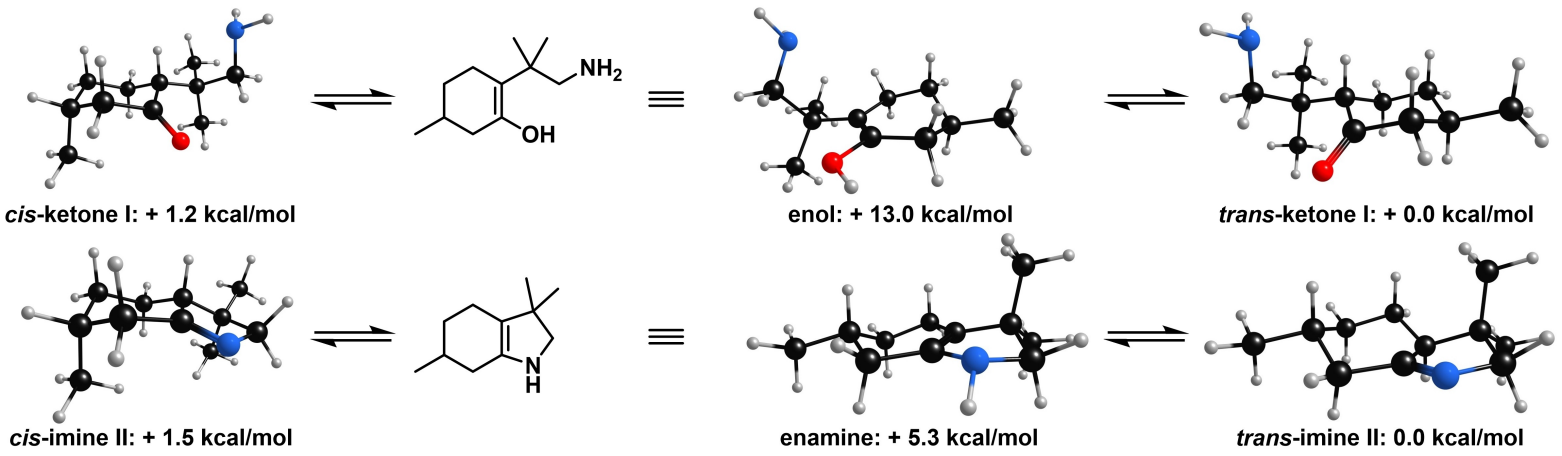
Tautomerization of intermediary occurring ketone **I** and imine **II**, which can influence the stereochemical product formation.

A comparison of the free enthalpies for the tautomerization equilibria of intermediates **I** and **II** obtained from DFT‐ calculations indicates that tautomerization of ketone **I** is less likely to occur than tautomerization of imine intermediate **II**. For a better understanding of the origin of the diastereoselectivity, we synthesized enantioenriched ketone **27** in a single step from commercially available (+)‐pulegone (Scheme 2).

This ketone represents a diastereomeric‐pure intermediate of our hydrogenation cascade and was exposed to the catalytic hydrogenation conditions. After protection with CbzCl, octahydroindole **28** was obtained in 81:16:3 d.r. and >99:1 e.r. for the main diastereomer. We rationalize the formation of the minor diastereomer by tautomerization of ketone **I** and/or imine **II**. Thus, the mechanism for the formation of **28** proceeds predominantly with retention of the stereocenters and tautomerization influences the stereochemical outcome only marginally. The stereochemistry of our cascade reaction is therefore mainly determined by the heterogeneous hydrogenation of the phenol starting material and imine intermediate (See Supporting Information for further information).

## Conclusion

In summary, we developed a new one‐pot cascade protocol for the synthesis of saturated N‐heterocycles starting from easily accessible precursors. Our approach combines multiple reductions, the adjustment of the desired N‐oxidation state and a cyclization through intramolecular reductive‐amination. In total, five individual reaction steps are carried out in one pot using only one catalyst and up to seven equivalents of molecular hydrogen are added. A broad series of octahydroindoles, decahydroquinolines, and a decahydroindolizine were obtained and isolated. The applicability of our strategy was demonstrated by the synthesis of protected octahydroindole **2** on gram‐scale. Further, mechanistic insights were investigated through the formation of enantioenriched octahydroindole **28** and DFT‐calculations.

## Experimental Section

**General Procedure for the hydrogenation of 2‐(2‐nitrovinyl)phenols**: A 20 mL glass vial equipped with a stir bar was charged with the corresponding phenol (0.3 mmol, 1.0 equiv.) and NaBH_4_ (11.4 mg, 0.3 mmol, 1.0 equiv.). Methanol (6 mL) was added and the mixture was stirred for 30 minutes at room temperature. Rh/C (30.9 mg, 5 mol%, 5 wt%) was added to mixture, the vial was covered with perforated aluminium foil and placed in a 150 mL stainless steel autoclave under air. The autoclave was pressurized and depressurized four times with hydrogen gas before the final hydrogen pressure was set to 5–10 bar. If not otherwise stated, the reaction mixture was stirred at 80 °C for 16 h. After the autoclave was carefully depressurized, NEt_3_ (0.9 mmol, 3.0 equiv.) and benzyl chloroformate (0.9 mmol, 3.0 equiv.) were added, and stirring continued for 30 minutes at room temperature. The mixture was filtered over silica gel using Et_2_O as eluent, an aliquot was used for determination of the diastereoselectivity by GC‐MS analysis and the solvent was removed in vacuo. The product was purified by column chromatography on silical gel.

## Supporting information

As a service to our authors and readers, this journal provides supporting information supplied by the authors. Such materials are peer reviewed and may be re‐organized for online delivery, but are not copy‐edited or typeset. Technical support issues arising from supporting information (other than missing files) should be addressed to the authors.

Supporting Information
