# Supplementary material for: Synthesis of Saturated N‐Heterocycles via a Catalytic Hydrogenation Cascade
Source: Adv Synth Catal. 2022 Jun 23;364(19):3366–71. doi: 10.1002/adsc.202200601 (PMC9796080; doi:10.1002/adsc.202200601)

Advanced Synthesis & Catalysis

Supporting Information

Synthesis of Saturated N-Heterocycles via a Catalytic Hydrogenation Cascade

Tobias Wagener,[+] Marco Pierau,[+] Arne Heusler, and Frank Glorius*© 2022 The Authors. Advanced Synthesis & Catalysis published by Wiley-VCH GmbH.. This is an open access article under the terms of the Creative Commons Attribution License, which permits use, distribution and reproduction in any medium, provided the original work is properly cited.

Supporting Information

Synthesis of Saturated N-Heterocycles via a Catalytic Hydrogenation Cascade

Tobias Wagener, Marco Pierau, Arne Heusler, and Frank Glorius*

glorius@uni-muenster.de

Organisch-Chemisches Institut, Westfälische Wilhelms-Universität Münster,

Corrensstraße 40, 48149 Münster

Table of Contents

1. General Information S2
2. Synthesis of Starting Materials S3
3. Investigation of Reaction Parameters S16
4. Reaction-Condition-Based Sensitivity Assessment S17
5. Catalytic Hydrogenation Reactions S19
6. Big-Scale Reaction S33
7. Mechanistic Investigation and DFT-Calculation S35
8. Literature S60
9. NMR Spectra S61

1. General Information

Unless otherwise noted, all reactions for starting material synthesis were carried out under an atmosphere of argon in oven-dried glassware. Catalytic hydrogenation reactions were prepared under air without further care of excluding moisture. Reaction temperatures are reported as the temperature of the bath surrounding the vessel unless otherwise stated. The solvents used for starting material synthesis were purified by distillation over the drying agents indicated in parentheses and were transferred under argon: toluene (CaH_2), dichloromethane (CaH_2), and THF (Na-benzophenone). Ethanol (4 Å) and methanol (3 Å) were purchased as dry solvents from commercial suppliers and stored over molecular sieves. Solvents for hydrogenation reactions (methanol, THF) were purchased as reagent grade solvents (puriss. p.a.; >99%) and used as received. All hydrogenation reactions were carried out in Berghof High Pressure Reactors using hydrogen gas.

Commercially available chemicals were obtained from Acros Organics, Aldrich Chemical Co., Strem Chemicals, Alfa Aesar, ABCR, Combi-Blocks, Chempur and TCI Europe and used as received. Heterogeneous catalysts were obtained from Johnson Matthey (Rh/C, 5 wt%, Ru/C, 5 wt%), Evonik Industries (Pd/C, 5 wt%) and Alfa Aesar (Pt/C, 5 wt%) and used as received.

Analytical thin layer chromatography (TLC) was performed on silica gel 60 F254 aluminum plates (Merck). TLC plates were visualized by exposure to short wave ultraviolet light (254 nm, 366 nm) and were dipped into a solution of $KMnO_4$. Flash chromatography was performed on Acros Organics silica gel (35-70 mesh) under a positive pressure of argon, eluting with the specified solvent system. GC-MS was conducted on an Agilent Technologies Intuvo 9000 (G3952A) with an Agilent 5977B GC/MSD. applying an HP-5MS ultra inert Intuvo GC column (19091S-233UI-INT). Alternatively, GC-MS was performed on an Agilent Technologies 7890A GC-system with an Agilent 5975C VL MSD using a DB-HeavyWAX column (0.25 mm x 30 m, 0.25 µm) for separation of certain diastereomeric octahydroindoles. ESI mass spectra were recorded on a Bruker Daltonics MicrOTof and a Thermo Fischer Scientific Orbitrap LTQ XL. ^{1}H, ^{13}C, and ^{19}F NMR spectra were recorded on a Bruker NEO 400, AgilentDD2 500 or AgilentDD2 600 in the indicated solvents. Chemical shifts (δ) are given in ppm relative to TMS. The residual solvent signals were used as references and the chemical shifts converted to the TMS scale (chloroform-*d*: δ_H = 7.26 ppm, δ_C = 77.16 ppm; methanol-d_4: δ_H = 3.31 ppm, δ_C = 49.0 ppm; dimethyl sulfoxide-d_6: δ_H = 2.50 ppm, δ_C = 39.52 ppm). ^{19}F NMR spectra are referenced according to the proton resonance of TMS as the primary reference for the unified chemical shift scale (IUPAC recommendation 2001).

2. Synthesis of Starting Materials

General Procedure 1 for the Mg-enabled ortho formylation of phenols (GP1):

Substituted salicylaldehydes were synthesized based on a modified literature procedure by Casiraghi.[1] Since this reaction is sensitive towards moisture, the following reagents were dried prior to use: $MgCl_2$ and paraformaldehyde were dried over P_2O_5, NEt_3 was stored over activated 4 Å molecular sieves and acetonitrile was dried using a solvent purification system. An oven-dried screw-cap flask was charged with $MgCl_2$ (1.5 equiv.) and the corresponding phenol (1.0 equiv., if solid). The flask was evacuated and refilled with Argon three times. The solids were suspended in acetronitrile (0.2 M), the corresponding phenol (1.0 equiv., if liquid) and NEt_3 (3.75 equiv.) were added. The flask was closed and stirred for 30 minutes at room temperature. Paraformaldehyde (6.75 equiv.) was added and the mixture was heated at 80 °C until the phenol was converted completely (three hours to overnight). The yellow suspension was allowed to come to room temperature, quenched with 2 N HCl solution and diluted with H_2O. The reaction mixture was extracted with Et_2O two times, the combined organic phases were dried over $MgSO_4$ and concentrated. The residue was purified by column chromatography on silica gel.

General Procedure 2 for the synthesis of salicylaldehydes via Duff-reaction (GP2):

An oven-dried screw-cap flask was charged with hexamethylenetetramine (1.0 equiv.) and the corresponding phenol (1.0 equiv., if solid). The flask was evacuated and refilled with Argon three times. The solids were dissolved in trifluoroacetic acid (0.33 M) and the corresponding phenol was added (1.0 equiv., if liquid). The flask was closed and stirred overnight at 90 °C. After cooling to room temperature, the mixture was diluted with H_2O and extracted with Et_2O for two times. The combined organic phased were dried over $MgSO_4$ and concentrated *in vacuo*. The residue was purified by column chromatography on silical gel.

General Procedure 3 for the synthesis of 2-(2-nitrovinyl)phenols via Henry-reaction (GP3):

An oven-dried screw-cap flask was charged with NH_4OAc (0.2 equiv.) and the corresponding salicylaldehyde (1.0 equiv., if solid). The flask was evacuated and refilled with Argon three times. The solids were dissolved in $MeNO_2$ (1.0 M) and acetic acid (2.5 M), followed by the addition of the corresponding salicylaldehyde (1.0 equiv., if liquid). The flask was closed and stirred overnight at 110 °C. After cooling to room temperature, the mixture was diluted with H_2O and extracted with Et_2O for two times. The combined organic phases were dried over $MgSO_4$ and concentrated *in vacuo*. The residue was purified by column chromatography on

silical gel. If necessary, the desired product was further purified by precipitation from a concentrated solution in dichloromethane using *n*-pentane.

General Procedure 4 for the synthesis of 2-(2-nitroethyl)phenols (GP4):

In an oven-dried screw-cap flask, the corresponding 2-(2-nitrovinyl)phenol (1.0 equiv.) was dissolved in dry THF (~0.1 M) under an atmosphere of Argon. The solution was cooled to −40 °C and the corresponding Grignard solution (3.0 equiv.) was added dropwise. The mixture was stirred for 2–3 h at −40 °C and was then allowed to come to room temperature. Aqueous hydrochloric acid (2 N) was added followed by addition of H_2O and extraction with ethyl acetate for three times. The combined organic phases were dried over $MgSO_4$ and concentrated *in vacuo*. The residue was purified by column chromatography on silical gel.

General Procedure 5 for the synthesis of 3-(2-hydroxyphenyl)acrylonitriles via Wittig-reaction (GP-5):

In an oven-dried screw-cap flask, (cyanomethyl)triphenylphosphonium chloride (1.5 equiv.) and KO*t*Bu (1.5 equiv.) were suspended in dry THF (0.2 M) under an atmosphere of Argon. The flask was closed and the suspension was stirred for one hour at 50 °C. After cooling to room temperature, the corresponding salicylaldehyde (1.0 equiv.) was added and the mixture was stirred overnight at 80 °C. After cooling to room temperature, the reaction was quenched by the addition of saturated aqueous NH_4Cl solution, diluted with H_2O and extracted with dichloromethane for three times. The combined organic phases were dried over $MgSO_4$ and the solvent was removed *in vacuo*. The residue was purified by column chromatography on silical gel.

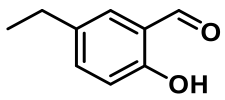

5-Ethyl-2-hydroxybenzaldehyde: The title compound was prepared according to GP1 from 4-ethylphenol (1.22 g, 10.0 mmol, 1.0 equiv.), $MgCl_2$ (1.43 g, 15.0 mmol, 1.5 equiv.), NEt_3 (5.23 mL, 37.5 mmol, 3.75 equiv.), and paraformaldehyde (2.03 g, 67.5 mmol, 6.75 equiv.) in acetonitrile (50 mL) at 90 °C. The product was isolated after column chromatography (eluent: *n*-pentane/EtOAc 95:5) as colorless liquid (1.32 g, 8.8 mmol, 88%).

^{1}H NMR (400 MHz, Chloroform-*d*) 10.86 (s, 1H), 9.87 (s, 1H), 7.39 – 7.34 (m, 2H), 6.92 (d, *J* = 8.3 Hz, 1H), 2.64 (q, *J* = 7.6 Hz, 2H), 1.24 (t, *J* = 7.6 Hz, 3H). **^{13}C{^{1}H} NMR** (101 MHz, Chloroform-*d*) 196.8, 159.9, 137.1, 135.8, 132.3, 120.5, 117.6, 27.8, 15.7. **HRMS** (ESI) m/z calculated for [$C_9H_9O_2$] ([M–H]$^-$) 149.0608, found 149.0606.

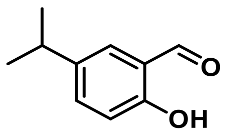

2-Hydroxy-5-isopropylbenzaldehyde: The title compound was prepared according to GP1 from 4-isopropylphenol (1.36 g, 10.0 mmol, 1.0 equiv.), $MgCl_2$ (1.43 g, 15.0 mmol, 1.5 equiv.), NEt_3 (5.23 mL, 37.5 mmol, 3.75 equiv.), and paraformaldehyde (2.03 g, 67.5 mmol, 6.75 equiv.) in acetonitrile (50 mL) at 90 °C. The product was isolated after column chromatography (eluent: *n*-pentane/EtOAc 95:5) as colorless liquid (1.58 g, 9.6 mmol, 96%).

^{1}H NMR (400 MHz, Chloroform-*d*) 10.86 (s, 1H), 9.88 (s, 1H), 7.44 – 7.37 (m, 2H), 6.93 (d, *J* = 8.5 Hz, 1H), 2.91 (hept, *J* = 6.9 Hz, 1H), 1.25 (d, *J* = 6.9 Hz, 6H). **^{13}C{^{1}H} NMR** (101 MHz, Chloroform-*d*) 196.8, 159.9, 140.5, 135.8, 130.9, 120.5, 117.6, 33.2, 24.1. **HRMS** (ESI) m/z calculated for [$C_{10}H_{11}O_2$] ([M–H]$^-$) 163.0765, found 163.0762.

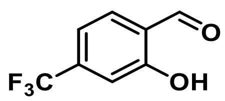

2-Hydroxy-4-(trifluoromethyl)benzaldehyde: The title compound was prepared according to GP2 from commercial 3-(trifluoromethyl)phenol (1.22 mL, 10 mmol, 1.0 equiv.) and hexamethylenetetramine (2.80 g, 20 mmol, 2.0 equiv.) in trifluoroacetic acid (10 mL) at 80 °C. The product was obtained after column chromatography (eluent: n-pentane/EtOAc 19:1) as a 3:1 mixture with 2-hydroxy-5-(trifluoromethyl)benzaldehyde (506.5 mg, 2.66 mmol, 27%). It was used without further purification for the synthesis of (*E*)-2-(2-nitrovinyl)-4-(trifluoromethyl)phenol.

The analytic data are in accordance with the literature.[2]

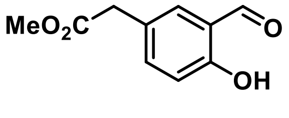

Methyl 2-(3-formyl-4-hydroxyphenyl)acetate: The title compound was prepared according to GP1 from methyl (4-hydroxyphenyl) acetate (1.66 g, 10.0 mmol, 1.0 equiv.), $MgCl_2$ (1.43 g, 15.0 mmol, 1.5 equiv.), NEt_3 (5.23 mL, 37.5 mmol, 3.75 equiv.), and paraformaldehyde (2.03 g, 67.5 mmol, 6.75 equiv.) in acetonitrile (50 mL) at 90 °C. The product was isolated after column chromatography (eluent: *n*-pentane/EtOAc 6:1) as white solid (1.53 g, 7.9 mmol, 79%).

^{1}H NMR (400 MHz, Chloroform-*d*) 10.94 (s, 1H), 9.87 (d, *J* = 0.6 Hz, 1H), 7.47 (d, *J* = 2.3 Hz, 1H), 7.44 (dd, *J* = 8.5, 2.3 Hz, 1H), 6.95 (d, *J* = 8.5 Hz, 1H), 3.70 (s, 3H), 3.61 (s, 2H). **^{13}C{^{1}H} NMR** (101 MHz,

Chloroform-*d*) 196.5, 171.8, 160.9, 138.2, 134.2, 125.6, 120.6, 118.0, 52.3, 39.9. **HRMS** (ESI) m/z calculated for [$C_{10}H_9O_4$] ([M–H]$^-$) 193.0495, found 193.0506.

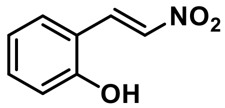

(*E*)-2-(2-Nitrovinyl)phenol: The title compound was prepared according to GP3 from salicylaldehyde (1.1 mL, 10.0 mmol, 1.0 equiv.), NH_4OAc (154 mg, 2.0 mmol, 0.2 equiv.), nitromethane (10.0 mL) and acetic acid (4.0 mL) at 110 °C. The product was isolated after column chromatography (eluent: n-pentane/EtOAc 9:1, later 4:1) as yellow solid, which was further purified by precipitation from a concentrated solution in dichloromethane with *n*-pentane (0.91 g, 5.5 mmol, 55%).

^{1}H NMR (400 MHz, Chloroform-*d*) 8.14 (d, *J* = 13.6 Hz, 1H), 7.98 (d, *J* = 13.5 Hz, 1H), 7.44 (d, *J* = 7.8 Hz, 1H), 7.35 (td, *J* = 7.7, 1.7 Hz, 1H), 7.01 (t, *J* = 7.6 Hz, 1H), 6.87 (d, *J* = 8.3 Hz, 1H), 5.98 – 5.83 (m, 1H). **^{13}C{^{1}H} NMR** (101 MHz, Chloroform-*d*) 156.1, 138.7, 135.7, 133.4, 132.8, 121.7, 117.8, 116.6. **HRMS** (ESI) m/z calculated for [$C_8H_6NO_3$] ([M–H]$^-$) 164.0342, found 164.0351.

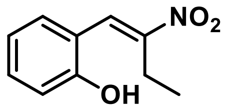

2-(2-Nitrobutyl)phenol: An oven-dried screw-cap flask was charged with 1-nitropropane (2.2 mL, 25.0 mmol, 5.0 equiv.), 2-hydroxybenzaldehyde (0.53 mL, 5.0 mmol, 1.0 equiv.), and acetic acid (0.97 mL, 17.0 mmol, 3.4 equiv.) under an atmosphere of Argon. The mixture was cooled to 0 °C and pyrrolidine (1.2 mL, 14.5 mmol, 2.9 equiv.) was added dropwise. Stirring was continued for four hours at 0 °C and the mixture was allowed to come to room temperature. Water was added followed by extraction with Et_2O for three times. The combined organic phased were dried over $MgSO_4$ and concentrated *in vacuo*. The residue was purified by column chromatography on silica gel (eluent: *n*-pentane/EtOAc 9:1) to give the desired product as yellow solid (276 mg, 1.4 mmol, 29%).

^{1}H NMR (400 MHz, Chloroform-*d*) 8.21 (s, 1H), 7.34 – 7.23 (m, 2H), 6.99 (t, *J* = 7.6 Hz, 1H), 6.89 (d, *J* = 8.2 Hz, 1H), 5.82 (s, 1H), 2.81 (q, *J* = 7.4 Hz, 2H), 1.24 (t, *J* = 7.4 Hz, 3H). **^{13}C{^{1}H} NMR** (101 MHz, Chloroform-*d*) 154.7, 153.6, 131.7, 129.6, 129.0, 121.1, 119.9, 116.2, 21.0, 12.6. **HRMS** (ESI) m/z calculated for [$C_{10}H_{10}NO_3$] ([M–H]$^-$) 192.0666, found 192.0665.

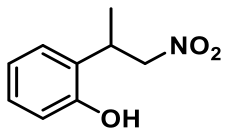

2-(1-Nitropropan-2-yl)phenol: The title compound was prepared according to GP4 from (*E*)-2-(2-nitrovinyl)phenol (495 mg, 3.0 mmol, 1.0 equiv.) and methylmagnesium bromide (3 mL, 9.0 mmol, 3.0 equiv., 3 M in Et_2O) in THF (25 mL). The product was isolated after column chromatography (eluent: *n*-pentane/EtOAc 9:1) as light yellow oil (232 mg, 1.3 mmol, 43%).

^{1}H NMR (400 MHz, Chloroform-*d*) 7.18 – 7.11 (m, 2H), 6.93 (td, *J* = 7.5, 1.2 Hz, 1H), 6.74 (dd, *J* = 7.9, 1.2 Hz, 1H), 5.01 (bs, 1H), 4.75 (dd, *J* = 12.0, 6.5 Hz, 1H), 4.53 (dd, *J* = 12.0, 8.4 Hz, 1H), 3.92 (dp, *J* = 8.5, 7.0 Hz, 1H), 1.41 (d, *J* = 7.0 Hz, 3H). **^{13}C{^{1}H} NMR** (101 MHz, Chloroform-*d*) 153.2, 128.6, 128.4, 127.2, 121.5, 116.0, 80.4, 33.8, 17.1. **HRMS** (ESI) m/z calculated for [$C_9H_{10}NO_3$] ([M–H]$^-$) 180.0655, found 180.0667.

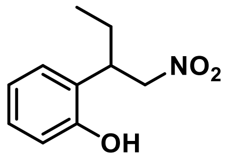

2-(1-Nitrobutan-2-yl)phenol: The title compound was prepared according to GP4 from (*E*)-2-(2-nitrovinyl)phenol (495 mg, 3.0 mmol, 1.0 equiv.) and ethylmagnesium bromide (3.0 mL, 9.0 mmol, 3.0 equiv., 3 M in Et_2O) in THF (25 mL). The product was isolated after column chromatography (eluent: *n*-pentane/EtOAc 9:1) as light yellow oil (252 mg, 1.3 mmol, 43%).

^{1}H NMR (400 MHz, Chloroform-*d*) 7.16 – 7.07 (m, 1H), 6.90 (t, *J* = 7.5 Hz, 1H), 6.74 (d, *J* = 8.0 Hz, 1H), 4.75 (dd, *J* = 12.1, 7.6 Hz, 1H), 4.64 (dd, *J* = 12.2, 7.2 Hz, 1H), 3.71 – 3.61 (m, 1H), 1.92 – 1.70 (m, 2H), 0.86 (t, *J* = 7.4 Hz, 3H). **^{13}C{^{1}H} NMR** (101 MHz, Chloroform-*d*) 153.8, 129.4, 128.6, 125.5, 121.2, 116.1, 79.4, 41.3, 24.5, 11.9. **HRMS** (ESI) m/z calculated for [$C_{10}H_{12}NO_3$] ([M–H]$^-$) 194.0812, found 194.0824.

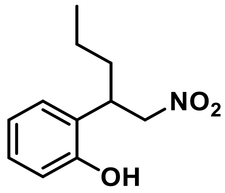

2-(1-Nitropentan-2-yl)phenol: The title compound was prepared according to GP4 from (*E*)-2-(2-nitrovinyl)phenol (495 mg, 3.0 mmol, 1.0 equiv.) and propylmagnesium chloride (4.5 mL, 9.0 mmol, 3.0 equiv., 2 M in Et_2O) in THF (25 mL). The product was isolated after column chromatography (eluent: *n*-pentane/EtOAc 9:1) as light yellow oil (207 mg, 1.0 mmol, 33%).

^{1}H NMR (400 MHz, Chloroform-*d*) 7.15 – 7.09 (m, 2H), 6.90 (td, *J* = 7.5, 1.1 Hz, 1H), 6.73 (dd, *J* = 7.9, 1.2 Hz, 1H), 4.73 (dd, *J* = 12.2, 7.8 Hz, 1H), 4.62 (dd, *J* = 12.2, 7.2 Hz, 1H), 3.76 (dtd, *J* = 10.0, 7.5, 5.2 Hz, 1H), 1.88 – 1.79 (m, 1H), 1.71 – 1.60 (m, 1H), 1.32 – 1.19 (m, 2H), 0.93 – 0.86 (m, 3H). OH proton missing. **^{13}C{^{1}H} NMR** (101 MHz, Chloroform-*d*) 153.7, 129.4, 128.6, 125.8, 121.4, 116.1, 79.7, 39.4, 33.6, 20.5, 14.0. **HRMS** (ESI) m/z calculated for [$C_{11}H_{14}NO_3$] ([M–H]$^-$) 208.0968, found 208.0980.

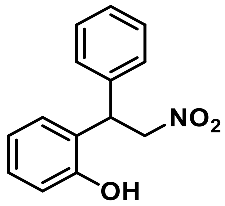

2-(2-Nitro-1-phenylethyl)phenol: The title compound was prepared according to GP4 from (*E*)-2-(2-nitrovinyl)phenol (330 mg, 2.0 mmol, 1.0 equiv.) and phenylmagnesium bromide (2.0 mL, 6.0 mmol, 3.0 equiv., 3 M in Et_2O) in THF (20 mL). The product was isolated after column chromatography (eluent: *n*-pentane/EtOAc 9:1) as light yellow oil (293 mg, 1.2 mmol, 60%).

^{1}H NMR (400 MHz, Chloroform-*d*) 7.31 – 7.18 (m, 5H), 7.11 – 7.00 (m, 2H), 6.84 (t, *J* = 7.5 Hz, 1H), 6.67 (dd, *J* = 8.1, 1.5 Hz, 1H), 5.40 – 5.30 (m, 1H), 5.18 (t, *J* = 8.1 Hz, 1H), 5.06 (dd, *J* = 12.9, 7.3 Hz, 1H), 4.95 (dd, *J* = 12.9, 8.8 Hz, 1H). **^{13}C{^{1}H} NMR** (101 MHz, Chloroform-*d*) 153.2, 138.8, 129.0, 128.9, 128.0, 127.6, 125.9, 121.3, 116.2, 78.0, 43.7. *Note: Some signals are overlapping.* **HRMS** (ESI) m/z calculated for [$C_{14}H_{13}NO_3Na$] ([M+Na]$^+$) 266.0788, found: 266.0788.

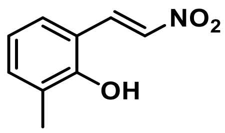

(*E*)-2-Methyl-6-(2-nitrovinyl)phenol: The title compound was prepared according to GP3 from 3-methyl-2-hydroxybenzaldehyde (1.21 mL, 10.0 mmol, 1.0 equiv.), NH_4OAc (154 mg, 2.0 mmol, 0.2 equiv.), nitromethane (10.0 mL) and acetic acid (4.0 mL) at 110 °C. The product was isolated after column chromatography (eluent: n-pentane/EtOAc 9:1, later 4:1) as yellow solid, which was further purified by precipitation from a concentrated solution in dichloromethane with *n*-pentane (0.68 g, 3.8 mmol, 38%).

^{1}H NMR (400 MHz, Chloroform-*d*) 8.18 (d, *J* = 13.6 Hz, 1H), 7.96 (d, *J* = 13.7 Hz, 1H), 7.30 (d, *J* = 7.7 Hz, 1H), 7.26 (d, *J* = 7.4 Hz, 1H), 6.92 (t, *J* = 7.6 Hz, 1H), 5.67 (s, 1H), 2.32 (s, 3H). **^{13}C{^{1}H} NMR** (101 MHz, Chloroform-*d*) 154.7, 138.4, 136.0, 134.7, 130.5, 123.7, 121.3, 117.5, 15.7. **HRMS** (ESI) m/z calculated for [$C_8H_6NO_3$] ([M–H]$^-$) 178.0499, found 178.0508.

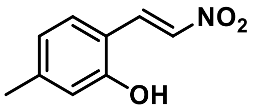

(*E*)-5-Methyl-2-(2-nitrovinyl)phenol: The title compound was prepared according to GP3 from 2-hydroxy-4-methylbenzaldehyde (1.36 g, 10.0 mmol, 1.0 equiv.), NH_4OAc (154 mg, 2.0 mmol, 0.2 equiv.), nitromethane (10.0 mL) and acetic acid (4.0 mL) at 110 °C. The product was isolated after column chromatography (eluent: *n*-pentane/EtOAc 9:1, later 4:1) as yellow solid (1.16 g, 6.5 mmol, 65%).

^{1}H NMR (400 MHz, Chloroform-*d*) 8.11 (d, *J* = 13.5 Hz, 1H), 7.93 (d, *J* = 13.5 Hz, 1H), 7.31 (d, *J* = 7.9 Hz, 1H), 6.82 (d, *J* = 7.8 Hz, 1H), 6.67 (s, 1H), 5.87 (s, 1H), 2.34 (s, 3H). **^{13}C{^{1}H} NMR** (101 MHz, Chloroform-*d*) 156.1, 144.8, 137.9, 135.8, 132.7, 122.8, 117.2, 115.2, 21.7. **HRMS** (ESI) m/z calculated for [$C_8H_6NO_3$] ([M–H]$^-$) 178.0499, found 178.0508.

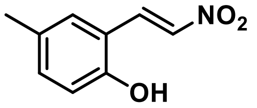

(*E*)-4-Methyl-2-(2-nitrovinyl)phenol: The title compound was prepared according to GP3 from 5-methyl-2-hydroxybenzaldehyde (1.36 g, 10.0 mmol, 1.0 equiv.), NH_4OAc (154 mg, 2.0 mmol, 0.2 equiv.), nitromethane (10.0 mL) and acetic acid (4.0 mL) at 110 °C. The product was isolated after column chromatography (eluent: n-pentane/EtOAc 9:1, later 4:1) as yellow solid, which was further purified by precipitation from a concentrated solution in dichloromethane with *n*-pentane (0.88 g, 4.9 mmol, 49%).

^{1}H NMR (400 MHz, Chloroform-*d*) 8.10 (d, *J* = 13.6 Hz, 1H), 7.94 (d, *J* = 13.6 Hz, 1H), 7.23 (d, *J* = 2.1 Hz, 1H), 7.15 (dd, *J* = 8.3, 2.2 Hz, 1H), 6.75 (d, *J* = 8.2 Hz, 1H), 5.49 (s, 1H), 2.30 (s, 3H). **^{13}C{^{1}H} NMR** (101 MHz, Chloroform-*d*) 153.9, 138.6, 135.7, 134.1, 132.8, 131.1, 117.5, 116.4, 20.4. **HRMS** (ESI) m/z calculated for [$C_9H_8NO_3$] ([M–H]$^-$) 178.0510, found 178.0508.

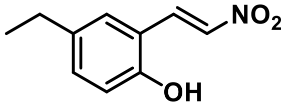

(*E*)-4-Ethyl-2-(2-nitrovinyl)phenol: The title compound was prepared according to GP3 from 5-ethyl-2-hydroxybenzaldehyde (1.30 g, 8.7 mmol, 1.0 equiv.), NH_4OAc (134 mg, 1.7 mmol, 0.2 equiv.), nitromethane (8.7 mL) and acetic acid (3.5 mL) at 110 °C. The product was isolated after column chromatography (eluent: *n*-pentane/EtOAc 9:1, later 6:1) as yellow solid (1.11 g, 5.8 mmol, 67%).

^{1}H NMR (400 MHz, Chloroform-*d*) 8.12 (d, *J* = 13.6 Hz, 1H), 7.95 (d, *J* = 13.6 Hz, 1H), 7.25 (d, *J* = 2.2 Hz, 1H), 7.18 (dd, *J* = 8.3, 2.2 Hz, 1H), 6.78 (d, *J* = 8.3 Hz, 1H), 2.61 (q, *J* = 7.6 Hz, 2H), 1.22 (t, *J* = 7.6 Hz, 3H). **^{13}C{^{1}H} NMR** (101 MHz, Chloroform-*d*) 154.1, 138.6, 137.6, 135.8, 133.0, 131.7, 117.5, 116.5, 27.9, 15.7. **HRMS** (ESI) m/z calculated for [$C_{10}H_{10}NO_3$] ([M–H]$^-$) 192.0655, found 192.0664.

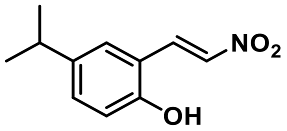

(*E*)-4-Isopropyl-2-(2-nitrovinyl)phenol: The title compound was prepared according to GP1 from 2-hydroxy-5-isopropylbenzaldehyde (1.55 g, 9.4 mmol, 1.0 equiv.), NH_4OAc (146 mg, 1.9 mmol, 0.2 equiv.), nitromethane (9.5 mL) and acetic acid (3.8 mL) at 110 °C. The product was isolated after column chromatography (eluent: *n*-pentane/EtOAc 9:1, later 6:1) as yellow solid (1.16 g, 5.6 mmol, 59%).

^{1}H NMR (400 MHz, Chloroform-*d*) 8.13 (d, *J* = 13.6 Hz, 1H), 7.99 (d, *J* = 13.5 Hz, 1H), 7.26 (d, *J* = 2.1 Hz, 1H), 7.21 (dd, *J* = 8.3, 2.3 Hz, 1H), 6.81 (d, *J* = 8.3 Hz, 1H), 2.87 (hept, *J* = 6.9 Hz, 1H), 1.23 (d, *J* = 6.9 Hz, 6H). **^{13}C{^{1}H} NMR** (101 MHz, Chloroform-*d*) 154.4, 142.1, 138.4, 136.3, 131.7, 130.5, 117.4, 116.6, 33.3, 24.1. **HRMS** (ESI) m/z calculated for [$C_{11}H_{13}NO_3Na$] ([M+Na$^+$]) 230.0788, found 230.0785.

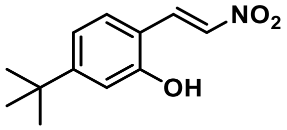

(*E*)-5-(*tert*-Butyl)-2-(2-nitrovinyl)phenol: The title compound was prepared according to GP1 from 4-(*tert*-butyl)-2-hydroxybenzaldehyde (0.77 g, 4.3 mmol, 1.0 equiv.), NH_4OAc (67 mg, 0.9 mmol, 0.2 equiv.), nitromethane (5.0 mL) and acetic acid (2.0 mL) at 110 °C. The product was isolated after column chromatography (eluent: *n*-pentane/EtOAc 9:1) as yellow solid (0.50 g, 2.2 mmol, 52%).

^{1}H NMR (400 MHz, Chloroform-*d*) 8.13 (d, *J* = 13.6 Hz, 1H), 7.99 (d, *J* = 13.5 Hz, 1H), 7.35 (d, *J* = 8.2 Hz, 1H), 7.02 (dd, *J* = 8.1, 1.8 Hz, 1H), 6.90 (d, *J* = 1.8 Hz, 1H), 6.29 (s, 1H), 1.31 (s, 9H). **^{13}C{^{1}H} NMR** (101 MHz, Chloroform-*d*) 158.2, 156.3, 137.9, 136.1, 132.7, 119.0, 115.0, 113.8, 35.2, 31.0. **HRMS** (ESI) m/z calculated for [$C_{12}H_{14}NO_3$] ([M–H]$^-$) 220.0968, found 220.0979.

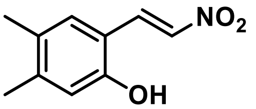

(*E*)-4,5-Dimethyl-2-(2-nitrovinyl)phenol: The title compound was prepared according to GP3 from 4,5-dimethyl-2-hydroxybenzaldehyde (1.14 g, 7.6 mmol, 1.0 equiv.), NH_4OAc (117 mg, 1.5 mmol, 0.2 equiv.). nitromethane (6.7 mL) and acetic acid (3.2 mL) at 110 °C. The product was isolated after column chromatography (eluent: *n*-pentane/EtOAc 9:1) as red solid (1.08 g, 5.6 mmol, 74%).

^{1}H NMR (400 MHz, Chloroform-*d*) 8.09 (d, *J* = 13.6 Hz, 1H), 7.91 (d, *J* = 13.5 Hz, 1H), 7.17 (s, 1H), 6.63 (s, 1H), 5.33 (s, 1H), 2.25 (s, 3H), 2.21 (s, 3H). **^{13}C{^{1}H} NMR** (101 MHz, Chloroform-*d*) 154.4, 143.4, 137.7, 135.8, 133.3, 129.9, 117.8, 115.2, 20.2, 18.8. **HRMS** (ESI) m/z calculated for [$C_{10}H_{10}NO_3$] ([M–H]$^-$) 192.0666, found: 192.0663.

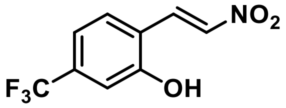

(*E*)-2-(2-Nitrovinyl)-5-(trifluoromethyl)phenol: The title compound was prepared according to GP3 from 2-hydroxy-4-(trifluoromethyl)benzaldehyde (660 mg, 3.5 mmol, 1.0 equiv.), NH_4OAc (53.5 mg, 0.7 mmol, 0.2 equiv.), nitromethane (3.1 mL) and acetic acid (1.4 mL) at 110 °C. The product was isolated after column chromatography (eluent: *n*-pentane/EtOAc 9:1) as yellowish solid (267 mg, 1.1 mmol, 33%).

^{1}H NMR (400 MHz, Methanol-*d*4) 8.13 (qd, *J* = 13.7, 2.2 Hz, 1H), 7.70 (dd, *J* = 8.5, 2.5 Hz, 1H), 7.17 (dd, *J* = 4.5, 2.7 Hz, 1H). OH proton missing. **^{13}C{^{1}H} NMR** (101 MHz, Methanol-*d*4) 159.5, 140.8, 134.9, 133.9, 126.4, 123.7, 122.4, 117.2, 113.7. **^{19}F{^{1}H}-NMR** (376 MHz, Chloroform-*d*): –63.3. **HRMS** (ESI) m/z calculated for [$C_9H_5F_3NO_3$] ([M–H]$^-$) 232.0227, found: 232.0221.

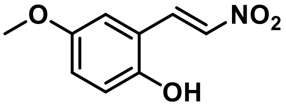

(*E*)-4-Methoxy-2-(2-nitrovinyl)phenol: The title compound was prepared according to GP1 from 2-hydroxy-5-methoxybenzaldehyde (1.25 mL, 10.0 mmol, 1.0 equiv.), NH_4OAc (154 mg, 2.0 mmol, 0.2 equiv.), nitromethane (10.0 mL) and acetic acid (4.0 mL) at 110 °C. The product was isolated after column chromatography (eluent: n-pentane/EtOAc 7:3) as orange solid, which was further purified by precipitation from a concentrated solution in dichloromethane with *n*-pentane (0.95 g, 4.9 mmol, 49%).

^{1}H NMR (400 MHz, Dimethyl sulfoxide-*d*6) 10.39 (s, 1H), 8.19 (s, 2H), 7.29 (d, *J* = 3.1 Hz, 1H), 6.99 (dd, *J* = 9.0, 3.1 Hz, 1H), 6.90 (d, *J* = 8.9 Hz, 1H), 3.72 (s, 3H). **^{13}C{^{1}H} NMR** (101 MHz, Dimethyl sulfoxide-*d*6) 152.5, 152.3, 137.4, 135.3, 121.2, 117.3, 116.9, 113.8, 55.6. **HRMS** (ESI) m/z calculated for [$C_9H_8NO_4Na$] ([M+Na$^+$]) 194.0448, found 194.0457.

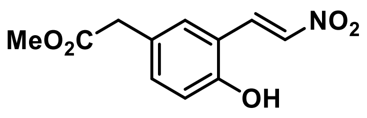

(*E*)-4-Hydroxy-3-(2-nitrovinyl)benzyl acetate: The title compound was prepared according to GP1 from methyl 2-(3-formyl-4-hydroxyphenyl)acetate (1.50 g, 7.7 mmol, 1.0 equiv.), NH_4OAc (122 mg, 1.6 mmol, 0.2 equiv.), nitromethane (10.0 mL) and acetic acid (4.0 mL) at 110 °C. The product was isolated after column chromatography (eluent: *n*-pentane/EtOAc 3:1) as yellow solid (1.09 g, 4.6 mmol, 59%).

^{1}H NMR (400 MHz, Dimethyl sulfoxide-*d*6) 10.86 (s, 1H), 8.18 – 8.07 (m, 2H), 7.58 (d, *J* = 2.3 Hz, 1H), 7.26 (dd, *J* = 8.4, 2.2 Hz, 1H), 6.93 (d, *J* = 8.4 Hz, 1H), 3.61 (s, 3H), 3.58 (s, 2H). **^{13}C{^{1}H} NMR** (101 MHz, Dimethyl sulfoxide-*d*6) 171.7, 157.2, 137.4, 135.3, 134.9, 132.3, 125.6, 116.6, 116.4, 51.7 **HRMS** (ESI) m/z calculated for [$C_{11}H_{10}O_5$] ([M–H]$^-$) 236.0554, found 236.0560.

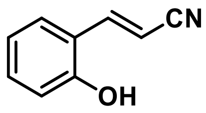

(*E*)-3-(2-Hydroxyphenyl)acrylonitrile: The title compound was prepared according to GP5 from 2-hydroxybenzaldehyde (0.52 mL, 5.0 mmol, 1.0 equiv.), (cyanomethyl)triphenylphosphonium chloride (2.53 g, 7.5 mmol, 1.5 equiv.) and KO*t*Bu (842 mg, 7.5 mmol, 1.5 equiv.) in THF (40 mL) at 80 °C. The product was isolated after column chromatography (eluent: *n*-pentane/EtOAc 3:1) as light yellow solid (579 mg, 4.0 mmol, 80%).

The analytical data are in agreement with the literature.[3]

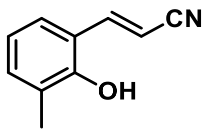

(*E*)-3-(2-Hydroxy-3-methylphenyl)acrylonitrile: The title compound was prepared according to GP5 from 3-methyl-2-hydroxybenzaldehyde (0.36 mL, 3.0 mmol, 1.0 equiv.), (cyanomethyl)triphenylphosphonium chloride (1.52 g, 4.5 mmol, 1.5 equiv.) and KO*t*Bu (505

mg, 4.5 mmol, 1.5 equiv.) in THF (25 mL) at 80 °C. The product was isolated after column chromatography (eluent: *n*-pentane/EtOAc 4:1) as light yellow solid (445 mg, 2.8 mmol, 93%).

^{1}H NMR (400 MHz, Chloroform-*d*) 7.68 (d, *J* = 16.7 Hz, 1H), 7.23 (d, *J* = 7.9 Hz, 1H), 7.19 (d, *J* = 7.5 Hz, 1H), 6.07 (d, *J* = 16.8 Hz, 1H), 5.51 (s, 1H), 2.28 (s, 3H). **^{13}C{^{1}H} NMR** (101 MHz, Chloroform-*d*) 153.3, 147.0, 133.6, 126.8, 123.9, 121.0, 120.9, 119.2, 96.7, 15.8. **HRMS** (ESI) m/z calculated for [$C_{10}H_8NO$] ([M–H]$^-$) 158.0600, found 158.0611.

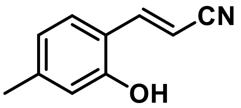

(*E*)-3-(2-Hydroxy-4-methylphenyl)acrylonitrile: The title compound was prepared according to GP5 from 4-methyl-2-hydroxybenzaldehyde (408 mg, 3.0 mmol, 1.0 equiv.), (cyanomethyl)triphenylphosphonium chloride (1.52 g, 4.5 mmol, 1.5 equiv.) and KO*t*Bu (505 mg, 4.5 mmol, 1.5 equiv.) in THF (25 mL) at 80 °C. The product was isolated after column chromatography (eluent: *n*-pentane/EtOAc 4:1) as light yellow solid (379 mg, 2.4 mmol, 79%).

^{1}H NMR (400 MHz, Chloroform-*d*) 7.57 (d, *J* = 16.8 Hz, 1H), 7.23 (d, *J* = 8.0 Hz, 1H), 6.76 (d, *J* = 8.0 Hz, 1H), 6.64 (s, 1H), 6.08 (d, *J* = 16.7 Hz, 1H), 2.32 (s, 3H). **^{13}C{^{1}H} NMR** (101 MHz, Chloroform-*d*) 155.1, 146.8, 143.4, 129.4, 122.3, 119.4, 118.6, 117.1, 95.9, 21.6. **HRMS** (ESI) m/z calculated for [$C_{10}H_8NO$] ([M–H]$^-$) 158.0600, found 158.0609.

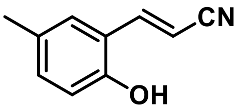

(*E*)-3-(2-Hydroxy-5-methylphenyl)acrylonitrile: The title compound was prepared according to GP5 from 5-methyl-2-hydroxybenzaldehyde (681 mg, 5.0 mmol, 1.0 equiv.), (cyanomethyl)triphenylphosphonium chloride (2.53 g, 7.5 mmol, 1.5 equiv.) and KO*t*Bu (842 mg, 7.5 mmol, 1.5 equiv.) in THF (30 mL) at 80 °C. The product was isolated after column chromatography (eluent: *n*-pentane/EtOAc 5:1) as light yellow solid (656 mg, 4.1 mmol, 82%).

^{1}H NMR (400 MHz, Chloroform-*d*) 7.58 (d, *J* = 16.7 Hz, 1H), 7.14 (d, *J* = 2.4 Hz, 1H), 7.07 (dd, *J* = 8.2, 2.4 Hz, 1H), 6.73 (d, *J* = 8.2 Hz, 1H), 6.13 (d, *J* = 16.7 Hz, 1H), 5.85 (s, 1H), 2.28 (s, 3H). **^{13}C{^{1}H} NMR** (101 MHz, Chloroform-*d*) 153.1, 147.1, 133.0, 130.5, 129.6, 120.7, 119.3, 116.5, 96.6, 20.5. **HRMS** (ESI) m/z calculated for [$C_{10}H_8NO$] ([M–H]$^-$) 158.0600, found 158.0608.

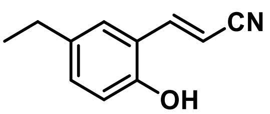

(*E*)-3-(5-Ethyl-2-hydroxyphenyl)acrylonitrile: The title compound was prepared according to GP5 from 5-ethyl-2-hydroxybenzaldehyde (436 mg, 2.9 mmol, 1.0 equiv.), (cyanomethyl)triphenylphosphonium chloride (1.47 g, 4.4 mmol, 1.5 equiv.) and KO*t*Bu (488 mg, 4.4 mmol, 1.5 equiv.) in THF (15 mL) at 80 °C. The product was isolated after column chromatography (eluent: *n*-pentane/EtOAc 5:1) as light yellow solid (429 mg, 2.5 mmol, 85%).

^{1}H NMR (400 MHz, Chloroform-*d*) 7.60 (d, *J* = 16.8 Hz, 1H), 7.15 (d, *J* = 2.2 Hz, 1H), 7.11 (dd, *J* = 8.2, 2.2 Hz, 1H), 6.77 (d, *J* = 8.2 Hz, 1H), 6.15 (d, *J* = 16.7 Hz, 1H), 5.95 (bs, 1H), 2.58 (q, *J* = 7.6 Hz, 2H), 1.21 (t, *J* = 7.6 Hz, 3H). **^{13}C{^{1}H} NMR** (101 MHz, Chloroform-*d*) 153.3, 147.3, 136.9, 131.9, 128.6, 120.8, 119.3, 116.5, 96.6, 28.0, 15.8. **HRMS** (ESI) m/z calculated for [$C_{11}H_{11}NONa$] ([M+Na^{+}]) 196.0733, found 196.0731.

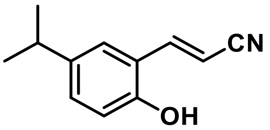

(*E*)-3-(2-Hydroxy-5-isopropylphenyl)acrylonitrile: The title compound was prepared according to GP5 from 5-isopropyl-2-hydroxybenzaldehyde (476 mg, 2.9 mmol, 1.0 equiv.), (cyanomethyl)triphenylphosphonium chloride (1.47 g, 4.4 mmol, 1.5 equiv.) and KO*t*Bu (488 mg, 4.4 mmol, 1.5 equiv.) in THF (15 mL) at 80 °C. The product was isolated after column chromatography (eluent: *n*-pentane/EtOAc 5:1) as light yellow solid (443 mg, 2.4 mmol, 82%).

^{1}H NMR (400 MHz, Chloroform-*d*) 7.60 (d, *J* = 16.8 Hz, 1H), 7.17 (d, *J* = 2.2 Hz, 1H), 7.14 (dd, *J* = 8.3, 2.3 Hz, 1H), 6.77 (d, *J* = 8.3 Hz, 1H), 6.16 (d, *J* = 16.7 Hz, 1H), 5.83 (bs, 1H), 2.85 (hept, *J* = 6.9 Hz, 1H), 1.22 (d, *J* = 6.9 Hz, 6H). **^{13}C{^{1}H} NMR** (101 MHz, Chloroform-*d*) 153.3, 147.4, 141.7, 130.5, 127.3, 120.7, 119.3, 116.5, 96.7, 33.3, 24.2. **HRMS** (ESI) m/z calculated for [$C_{12}H_{13}NONa$] ([M+Na^{+}]) 210.0889, found 210.0888.

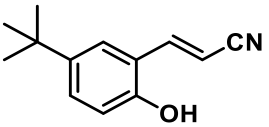

(*E*)-3-(5-(*tert*-Butyl)-2-hydroxyphenyl)acrylonitrile: The title compound was prepared according to GP5 from 5-(*tert*-butyl)-2-hydroxybenzaldehyde (890 mg, 5.0 mmol, 1.0 equiv.), (cyanomethyl)triphenylphosphonium chloride (2.53 g, 7.5 mmol, 1.5 equiv.) and KO*t*Bu (842

mg, 7.5 mmol, 1.5 equiv.) in THF (15 mL) at 80 °C. The product was isolated after column chromatography (eluent: *n*-pentane/EtOAc 5:1) as light yellow solid (837 mg, 3.9 mmol, 79%).

^{1}H NMR (400 MHz, Chloroform-*d*) 7.60 (d, *J* = 16.8 Hz, 1H), 7.34 – 7.27 (m, 2H), 6.82 – 6.75 (m, 1H), 6.19 (d, *J* = 16.7 Hz, 1H), 6.04 (s, 1H), 1.30 (s, 9H). **^{13}C{^{1}H} NMR** (101 MHz, Chloroform-*d*) 153.2, 147.8, 143.9, 129.6, 126.4, 120.3, 119.4, 116.3, 96.7, 34.2, 31.5. **HRMS** (ESI) m/z calculated for [$C_{13}H_{15}NONa$] ([M+Na$^+$]) 223.1046, found 223.1043.

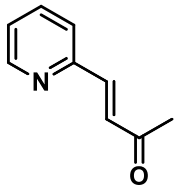

(*E*)-4-(Pyridin-2-yl)but-3-en-2-one: The title compound was prepared according to GP5 from picolinaldehyde (0.76 mL, 8.0 mmol, 1.0 equiv.), (2-oxopropyl)triphenylphosphonium chloride (3.69 g, 10.4 mmol, 1.3 equiv.) and KO*t*Bu (1.17 g, 10.4 mmol, 1.3 equiv.) in THF (50 mL) at 80 °C. The product was isolated after column chromatography (eluent: *n*-pentane/EtOAc 1:1) as colorless liquid (842 mg, 5.7 mmol, 71%).

^{1}H NMR (400 MHz, Chloroform-*d*) 8.63 (d, *J* = 4.8 Hz, 1H), 7.70 (td, *J* = 7.7, 1.8 Hz, 1H), 7.50 (d, *J* = 16.0 Hz, 1H), 7.46 (d, *J* = 7.9 Hz, 1H), 7.26 (ddd, *J* = 7.9, 4.8, 1.3 Hz, 1H), 7.12 (d, *J* = 16.1 Hz, 1H), 2.38 (s, 3H). **^{13}C{^{1}H} NMR** (101 MHz, Chloroform-*d*) 198.6, 153.3, 150.3, 142.0, 136.9, 130.3, 124.4, 124.3, 28.2. **HRMS** (ESI) m/z calculated for [$C_9H_{10}NO$] ([M+H$^+$]) 148.0757, found 148.0753.

3. Investigation of Reaction Parameters

General Procedure for the hydrogenation of nitrovinyl-substituted phenoles:

A 4 mL screw cap glass vial equipped with a stir bar was charged with 2-(2-nitrovinyl)phenol (16.5 mg, 0.1 mmol, 1.0 equiv.) and $NaBH_4$ (3.8 mg, 0.1 mmol, 1.0 equiv.). Methanol (1 mL) was added and the mixture was stirred for 30 minutes at room temperature. Rh/C (10.3 mg, 5 mol%, 5 wt%) was added to mixture, the vial was screwed shut and the lid pierced with a needle, which is used to equilibrate the gas phase. The vial was placed in a 150 mL stainless steel autoclave under air. The autoclave was pressurized and depressurized four times with hydrogen gas before the final hydrogen pressure was set to 5 bar. The reaction mixture was stirred at 60 °C for 16 h. After the autoclave was carefully depressurized, NEt_3 (0.3 mmol, 3.0 equiv.), benzyl chloroformate (0.3 mmol, 3.0 equiv.) and internal standard mesitylene (13.9 µL, 0.1 mmol, 1.0 equiv.) were added, and stirring continued for 30 minutes at room temperature. An aliquot was filtered over silica gel and was used for determination of the reaction yield by GC-FID analysis.

Table S1: Investigation of reaction parameters.

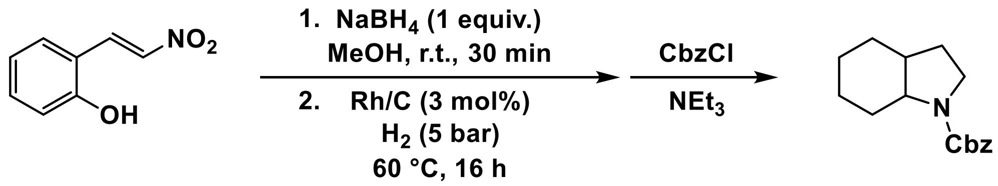

entry	deviation	yield A
**1**	none	70%
**2**	no $NaBH_4$	9%
**3**	THF/H_2O (1:1) instead MeOH	49%
**4**	no $NaBH_4$, THF instead MeOH	<5%
**5**	no $NaBH_4$, $CHCl_3$ instead MeOH	<5%
**6**	no $NaBH_4$, *i*PrOH instead MeOH	9%
**7**	Pd/C (5 wt%) instead Rh/C	17%
**8**	Ru/C (5 wt%) instead Rh/C	<5%
**9**	Pt/C (5 wt%) instead Rh/C	<5%
**10**	0.5 mL MeOH, c = 0.2 M	60%
**11**	2.0 mL MeOH, c = 0.05 M	70%

4. Reaction-Condition-Based Sensitivity Assessment

Following a procedure recently developed by our group,[4] a reaction-condition-based sensitivity screen was conducted. Key reaction parameters were varied in a systematic manner and the reaction results were compared to the standard reaction conditions. Table S2 gives an overview of the experimental results.

Preparation of the stock solution: (*E*)-2-(2-nitrovinyl)phenol (132.1 mg, 0.8 mmol, 1.0 equiv.) was dissolved in MeOH (7.3 mL, 0.11 M) and $NaBH_4$ (30.3 mg, 0.8 mmol, 1.0 equiv.) was added. The mixture was stirred for 30 minutes at room temperature.

Standard reaction conditions: n = 0.10 mmol, c = 0.1 M, V = 1 mL, T = 60 °C, $p(H_2)$ = 5 bar.

Standard reaction procedure: A 4 ml glass vial (screw-cap) equipped with a stir bar was charged with 5 wt% Rh/C (6.2 mg, 3 mol%), stock solution (0.9 mL) and MeOH (0.1 mL) (total concentration c = 0.10 M, total volume V = 1 mL). The glass vial was placed in a 150 ml stainless steel autoclave under air. The autoclave was pressurized and depressurized four times with hydrogen gas before the final hydrogen pressure was set to 5 bar. The reaction mixture was stirred at 60 °C for 16 h. After the autoclave was carefully depressurized, NEt_3 (42 µL, 3.0 equiv.), CbzCl (43 µL, 3.0 equiv.) and mesitylene (13.9 µl) were carefully added and the mixture was stirred for 30 minutes at room temperature. The product yield was determined using GC-FID after filtration.

Gram-scale conditions: n = 7.0 mmol, c = 0.1 M, V = 70 mL, air atmosphere, T = 60 °C, $p(H_2)$ = 10 bar.

Analysis: By systematic variation we determined the influence of the reaction parameters of concentration, temperature, hydrogen pressure, water content and oxygen content on the reaction outcome. Interestingly, small changes in the concentration, the temperature, a higher hydrogen pressure, the addition of water, the presence of oxygen and an increase in the reaction scale showed only minor effects on the reaction yield. However, both, lowering the hydrogen pressure to 3 bar as well as the absence of water significantly decreased the reaction yield.

Table S2: Investigation of the reaction-condition-based sensitivity.

entry	experiment	deviation	yield	yield dev.
**1**	standard	none	65%	-
**2**	high *c*	no extra MeOH	62%	−4%
**3**	low *c*	+0.2 ml MeOH	60%	−8%
**4**	H_2O	+10 µL H_2O	65%	0%
**5**	low H_2O	water-free solvent	15%	−67%
**6**	high O_2	autoclave was not flushed	69%	+7%
**7**	low *T*	*T* = 50 °C	64%	−1%
**8**	high *T*	*T* = 70 °C	60%	−8%
**9**	low *p*	*p* = 3 bar	41%	−37%
**10**	high *p*	*p* = 7 bar	65%	0%
**11**	big scale	*n*(substrate) = 7.0 mmol	69%	+6%

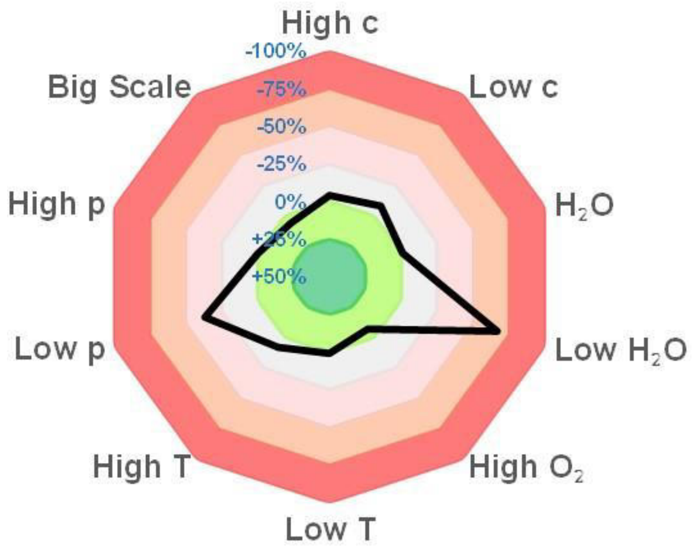

Figure S1. Radar diagram of the sensitivity assessment.

5. Catalytic Hydrogenation Reactions

General Procedure A for the hydrogenation of 2-(2-nitrovinyl)phenols (GP-A):

A 20 mL glass vial equipped with a stir bar was charged with the corresponding phenol (0.3 mmol, 1.0 equiv.) and $NaBH_4$ (11.4 mg, 0.3 mmol, 1.0 equiv.). Methanol (6 mL) was added and the mixture was stirred for 30 minutes at room temperature. Rh/C (30.9 mg, 5 mol%, 5 wt%) was added to mixture, the vial was covered with perforated aluminum foil and placed in a 150 mL stainless steel autoclave under air. The autoclave was pressurized and depressurized four times with hydrogen gas before the final hydrogen pressure was set to 5–10 bar. If not otherwise stated, the reaction mixture was stirred at 80 °C for 16 h. After the autoclave was carefully depressurized, NEt_3 (0.9 mmol, 3.0 equiv.) and benzyl chloroformate (0.9 mmol, 3.0 equiv.) were added, and stirring continued for 30 minutes at room temperature. The mixture was filtered over silica gel using Et_2O as eluent, an aliquot was used for determination of the diastereoselectivity by GC-MS analysis and the solvent was removed *in vacuo*. The product was purified by column chromatography on silical gel.

General Procedure B for the hydrogenation of 2-(2-nitroethyl)phenols and (2-hydroxyphenyl)acrylonitriles (GP-B):

A 20 mL glass vial equipped with a stir bar was charged with the corresponding phenol (0.3 mmol, 1.0 equiv.) and Rh/C (30.9 mg, 5 mol%, 5 wt%). Methanol (6 mL) was added and the vial was covered with perforated aluminum foil and placed in a 150 mL stainless steel autoclave under air. The autoclave was pressurized and depressurized four times with hydrogen gas before the final hydrogen pressure was set to 5–10 bar. If not otherwise stated, the reaction mixture was stirred at 80 °C for 16 h. After the autoclave was carefully depressurized, NEt_3 (0.9 mmol, 3.0 equiv.) and benzyl chloroformate (0.9 mmol, 3.0 equiv.) were added, and stirring continued for 30 minutes at room temperature. The mixture was filtered over silica gel using Et_2O as eluent, an aliquot was used for determination of the diastereoselectivity by GC-MS analysis and the solvent was removed *in vacuo*. The product was purified by column chromatography on silical gel.

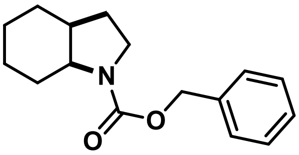

Benzyl octahydro-1*H*-indole-1-carboxylate : The title compound was prepared according to GP-A from 2-(2-nitrovinyl)phenol (49.5 mg, 0.30 mmol, 1.0 equiv.), $NaBH_4$ (11.4 mg, 0.30 mmol, 1.0 equiv.) and 5 wt% Rh/C (18.5 mg, 3 mol%) in methanol (0.9 mL) at 60 °C with 5 bar H_2 pressure. The product was isolated after column chromatography (eluent: *n*-pentane/EtOAc 95:5) as colorless liquid (59.1 mg, 0.23 mmol, 76%, >95:5 d.r. by GC-MS). The relative configuration of the product was confirmed by comparing the retention time in gas chromatography with a reference sample from a cis-selective synthesis known in the literature.[5]

^{1}H NMR (500 MHz, 363 K, Dimethyl sulfoxide-*d*6) 7.39 – 7.27 (m, 5H), 5.11 – 5.03 (m, 2H), 3.70 (dt, *J* = 10.0, 6.2 Hz, 1H), 3.42 (ddd, *J* = 11.3, 8.8, 2.7 Hz, 1H), 3.34 (dt, *J* = 10.5, 8.4 Hz, 1H), 2.27 – 2.18 (m, 1H), 1.92 – 1.82 (m, 2H), 1.78 – 1.69 (m, 1H), 1.68 – 1.60 (m, 2H), 1.58 – 1.51 (m, 1H), 1.45 – 1.24 (m, 3H), 1.17 (ddt, *J* = 16.1, 12.5, 6.0 Hz, 1H). **^{13}C{^{1}H} NMR** (126 MHz, 363 K, Dimethyl sulfoxide-*d*6) 153.4, 137.0, 127.8, 127.1, 126.8, 65.1, 55.8, 44.5, 36.3, 26.7, 25.9, 25.3, 22.4, 20.2. **HRMS** (ESI) m/z calculated for [$C_{16}H_{21}NO_2Na$] ([M+Na$^+$]) 282.1465, found 282.1467.

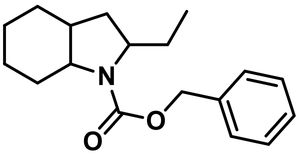

Benzyl 2-ethyloctahydro-1*H*-indole-1-carboxylate: The title compound was prepared according to GP-A from (*E*)-2-(2-nitrobut-1-en-1-yl)phenol (58.0 mg, 0.30 mmol, 1.0 equiv.), $NaBH_4$ (11.4 mg, 0.30 mmol, 1.0 equiv.) and 5 wt% Rh/C (18.5 mg, 3 mol%) in methanol (3.0 mL) at 60 °C with 5 bar H_2 pressure. The product was isolated after column chromatography (eluent: *n*-pentane/EtOAc 98:2, later 95:5) as colorless liquid (61.0 mg, 0.21 mmol, 71%, 91:9 d.r. by GC-MS). The product was isolated as a mixture of diastereomers.

^{1}H NMR (500 MHz, 363 K, Dimethyl sulfoxide-*d*6) 7.39 – 7.27 (m, 5H), 5.11 – 5.03 (m, 2H), 3.79 (dt, *J* = 10.7, 6.4 Hz, 1H), 3.70 – 3.57 (m, 1H), 2.24 – 2.14 (m, 1H), 2.11 – 2.01 (m, 1H), 1.97 – 1.83 (m, 2H), 1.72 – 1.57 (m, 4H), 1.47 – 1.25 (m, 3H), 1.22 – 1.07 (m, 2H), 0.84 (t, *J* = 7.7 Hz, 3H). **^{13}C{^{1}H} NMR** (126 MHz, 363 K, Dimethyl sulfoxide-*d*6) 153.7, 137.0, 127.8, 127.1, 126.8, 65.1, 58.6, 57.2, 35.2, 32.4, 28.4, 27.7, 25.2, 23.1, 19.7, 9.3. **HRMS** (ESI) m/z calculated for [$C_{18}H_{25}NO_2Na$] ([M+Na$^+$]) 310.1778, found 310.1783.

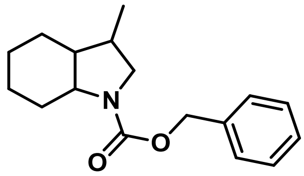

Benzyl 3-methyloctahydro-1*H*-indole-1-carboxylate: The title compound was prepared according to GP-B from 2-(1-nitropropan-2-yl)phenol (54.4 mg, 0.30 mmol, 1.0 equiv.) and 5 wt% Rh/C (30.9 mg, 5 mol%) in methanol (6.0 mL) at 80 °C with 10 bar H_2 pressure. The product was isolated after column chromatography (eluent: *n*-pentane/EtOAc 95:5) as colorless liquid (57.5 mg, 0.21 mmol, 70%, 40:38:22 d.r. by GC-MS). The product was obtained as a mixture of diastereomers. The analytical data of a 1:1 mixture of two diastereomers are given.

^{1}H NMR (500 MHz, 363 K, Dimethyl sulfoxide-*d*$_6$) 7.38 – 7.28 (m, 10H), 5.11 – 5.02 (m, 4H), 3.73 (q, *J* = 4.5 Hz, 1H), 3.66 (dd, *J* = 10.6, 7.8 Hz, 1H), 3.60 (dd, *J* = 10.6, 7.8 Hz, 1H), 3.06 – 2.99 (m, 1H), 2.91 (td, *J* = 10.3, 3.4 Hz, 1H), 2.83 (t, *J* = 10.4 Hz, 1H), 2.66 – 2.59 (m, 1H), 2.48 – 2.39 (m, 1H), 2.23 – 2.12 (m, 1H), 2.01 – 1.94 (m, 1H), 1.87 (d, *J* = 9.2 Hz, 1H), 1.78 – 1.61 (m, 4H), 1.56 – 1.45 (m, 2H), 1.35 (dt, *J* = 11.9, 5.1 Hz, 1H), 1.29 – 1.02 (m, 8H), 0.96 (d, *J* = 6.4 Hz, 3H), 0.92 (d, *J* = 6.9 Hz, 3H). **^{13}C{^{1}H} NMR** (126 MHz, 363 K, Dimethyl sulfoxide-*d*$_6$) 155.3, 155.3, 137.8, 137.8, 128.7, 128.7 128.0, 128.0, 127.9, 127.8, 66.1, 66.0, 64.3, 58.6, 55.1, 53.3, 52.9, 41.4, 36.0, 35.1, 31.9, 27.6, 27.3, 25.8, 24.8, 24.1, 22.2, 20.8, 14.8, 12.7. **HRMS** (ESI) m/z calculated for [$C_{17}H_{23}NO_2Na$] ([M+Na$^+$]) 296.1621, found 296.1620.

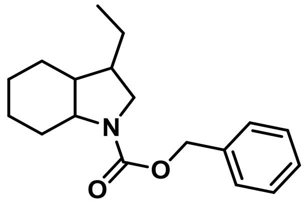

Benzyl 3-ethyloctahydro-1*H*-indole-1-carboxylate: The title compound was prepared according to GP-B from 2-(1-nitropropan-2-yl)phenol (58.6 mg, 0.30 mmol, 1.0 equiv.) and 5 wt% Rh/C (30.9 mg, 5 mol%) in methanol (6.0 mL) at 80 °C with 10 bar H_2 pressure. The product was isolated after column chromatography (eluent: *n*-pentane/EtOAc 95:5) as colorless liquid (49.7 mg, 0.17 mmol, 58%, 34:33:33 d.r. by GC-MS). The product was obtained as a mixture of diastereomers. The analytical data of a 1:1 mixture of two diastereomers are given.

^{1}H NMR (599 MHz, 363 K, Dimethyl sulfoxide-*d*$_6$) 7.37 – 7.26 (m, 10H), 5.10 – 5.01 (m, 4H), 3.72 – 3.68 (m, 1H), 3.65 (t, *J* = 9.1 Hz, 1H), 3.59 (t, *J* = 9.4 Hz, 1H), 3.03 (t, *J* = 11.0 Hz, 1H), 2.93 – 2.84 (m, 2H), 2.63 – 2.58 (m, 1H), 2.54 – 2.49 (m, 1H), 2.05 – 1.98 (m, 1H), 1.98 – 1.90 (m, 1H), 1.90 – 1.85 (m, 1H), 1.76 – 1.67 (m, 2H), 1.66 – 1.53 (m, 3H), 1.52 – 1.40 (m, 2H), 1.38 – 1.02 (m, 12H), 0.91 – 0.83 (m,

6H). **$^{13}C\{^{1}H\}$ NMR** (151 MHz, 363 K, Dimethyl sulfoxide-*d*6) 154.5, 154.4, 136.9, 136.9, 127.8, 127.8, 127.1, 127.1, 126.9, 126.9, 65.1, 65.1, 63.5, 57.7, 52.3, 50.8, 50.4, 42.1, 41.9, 39.1, 31.0, 27.2, 26.3, 24.9, 24.0, 23.3, 22.7, 21.0, 19.7, 19.6, 11.7, 11.3. **HRMS** (ESI) m/z calculated for [$C_{17}H_{23}NO_2Na$] ([M+Na$^+$]) 310.1778, found 310.1774.

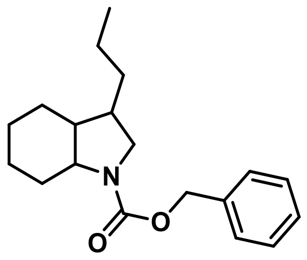

Benzyl 3-propyloctahydro-1*H*-indole-1-carboxylate: The title compound was prepared according to GP-B from 2-(1-nitropentan-2-yl)phenol (62.8 mg, 0.30 mmol, 1.0 equiv.) and 5 wt% Rh/C (30.9 mg, 5 mol%) in methanol (6.0 mL) at 80 °C with 10 bar H_2 pressure. The product was isolated after column chromatography (eluent: *n*-pentane/EtOAc 95:5) as colorless liquid (58.2 mg, 0.19 mmol, 64%, 37:33:30 d.r. by GC-MS). The product was obtained as a mixture of diastereomers. The analytical data of a 1:1 mixture of two diastereomers are given.

^{1}H NMR (500 MHz, 363 K, Dimethyl sulfoxide-*d*6) 7.39 – 7.28 (m, 10H), 5.11 – 5.03 (m, 4H), 3.72 (q, *J* = 4.1 Hz, 1H), 3.67 (dd, *J* = 10.6, 7.7 Hz, 1H), 3.60 (dd, *J* = 10.7, 7.7 Hz, 1H), 3.08 – 3.02 (m, 1H), 2.94 – 2.85 (m, 2H), 2.66 – 2.59 (m, 1H), 2.58 – 2.51 (m, 1H), 2.09 – 1.98 (m, 2H), 1.92 – 1.86 (m, 1H), 1.78 – 1.68 (m, 2H), 1.68 – 1.61 (m, 2H), 1.57 – 1.40 (m, 3H), 1.39 – 1.05 (m, 16H), 0.92 – 0.87 (m, 6H). **$^{13}C\{^{1}H\}$ NMR** (126 MHz, 363 K, Dimethyl sulfoxide-*d*6) 155.5, 155.3, 137.8, 137.8, 128.7, 128.7 128.0, 128.0, 127.9, 127.8, 69.5, 66.1, 64.4, 58.6, 55.1, 53.6, 52.1, 51.6, 41.2, 40.3, 33.3, 31.9, 29.8, 28.1, 27.2, 25.8, 24.9, 24.3, 22.1, 21.2, 21.0, 20.7, 14.5, 14.4. **HRMS** (ESI) m/z calculated for [$C_{19}H_{27}NO_2Na$] ([M+Na$^+$]) 324.1934, found 324.1929.

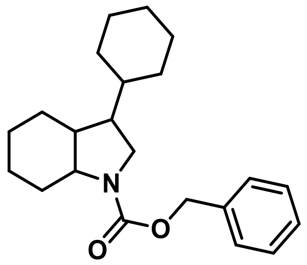

Benzyl 3-cyclohexyloctahydro-1*H*-indole-1-carboxylate: The title compound was prepared according to GP-B from 2-(2-nitro-1-phenylethyl)phenol (73.0 mg, 0.30 mmol, 1.0 equiv.) and 5 wt% Rh/C (30.9 mg, 5 mol%) in methanol (6.0 mL) at 80 °C with 10 bar H_2 pressure. The product was isolated after column chromatography (eluent: *n*-pentane/EtOAc 95:5) as

colorless liquid (63.5 mg, 0.19 mmol, 62%, 43:30:27 d.r. by GC-MS). The product was obtained as a mixture of diastereomers. The analytical data of a mixture of two diastereomers are given.

^{1}H NMR (599 MHz, 363 K, Dimethyl sulfoxide-*d*6) 7.39 – 7.28 (m, 10H), 5.11 – 5.02 (m, 4H), 3.71 – 3.67 (m, 1H), 3.60 (t, *J* = 9.1 Hz, 1H), 3.51 (t, *J* = 9.2 Hz, 1H), 3.10 (t, *J* = 11.0 Hz, 1H), 3.04 (t, *J* = 10.7 Hz, 1H), 2.91 (t, *J* = 10.3 Hz, 1H), 2.64 (d, *J* = 11.5 Hz, 2H), 2.07 – 2.01 (m, 1H), 1.94 (d, *J* = 12.1 Hz, 1H), 1.77 – 1.47 (m, 16H), 1.47 – 1.32 (m, 4H), 1.31 – 0.99 (m, 16H), 0.98 – 0.88 (m, 2H). **^{13}C{^{1}H} NMR** (151 MHz, 363 K, Dimethyl sulfoxide-*d*6) 154.6, 154.5, 136.9, 136.9, 127.8, 127.8, 127.1, 127.1, 126.9, 126.9, 65.1, 65.1, 63.7, 57.9, 49.4, 49.3, 47.9, 45.9, 45.5, 38.2, 37.1, 34.8, 31.0, 30.9, 30.9, 30.4, 28.3, 28.2, 26.2, 25.6, 25.6, 25.5, 25.5, 25.0, 24.9, 24.9, 23.9, 23.5, 20.9, 19.7. **HRMS** (ESI) m/z calculated for [$C_{22}H_{31}NO_2Na$] ([M+Na$^+$]) 364.2247, found 364.2238.

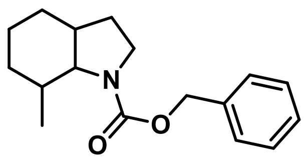

Benzyl 7-methyloctahydro-1*H*-indole-1-carboxylate: The title compound was prepared according to GP-A from (*E*)-2-methyl-6-(2-nitrovinyl)phenol (53.8 mg, 0.30 mmol, 1.0 equiv.), $NaBH_4$ (11.4 mg, 0.30 mmol, 1.0 equiv.) and 5 wt% Rh/C (30.9 mg, 5 mol%) in methanol (6.0 mL) with aq. NaOH solution (0.23 mL, 1.5 equiv., 2 N solution) at 80 °C with 10 bar H_2 pressure. The product was isolated after column chromatography (eluent: *n*-pentane/EtOAc 95:5) as colorless liquid (32.3 mg, 0.12 mmol, 39%, 55:18:12:12 d.r. by GC-MS). Only the analytical data of the main diastereomer are given.

^{1}H NMR (500 MHz, 363 K, Dimethyl sulfoxide-*d*6) 7.38 – 7.27 (m, 5H), 5.12 – 5.04 (m, 2H), 3.81 (t, *J* = 7.4 Hz, 1H), 3.42 – 3.37 (m, 2H), 2.36 – 2.21 (m, 2H), 1.81 – 1.72 (m, 2H), 1.69 – 1.59 (m, 2H), 1.56 – 1.37 (m, 3H), 1.32 – 1.23 (m, 1H), 0.82 (d, *J* = 7.5 Hz, 3H). **^{13}C{^{1}H} NMR** (126 MHz, 363 K, Dimethyl sulfoxide-*d*6) 153.5, 137.0, 127.8, 127.1, 126.8, 65.2, 57.9, 45.6, 36.0, 28.9, 28.8, 28.4, 25.3, 15.1, 14.1. **HRMS** (ESI) m/z calculated for [$C_{17}H_{23}NO_2Na$] ([M+Na$^+$]) 296.1621, found 296.1621.

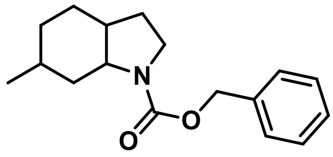

Benzyl 6-methyloctahydro-1*H*-indole-1-carboxylate: The title compound was prepared according to GP-A from (*E*)-5-methyl-2-(2-nitrovinyl)phenol (53.8 mg, 0.30 mmol, 1.0 equiv.), $NaBH_4$ (11.4 mg, 0.30 mmol, 1.0 equiv.) and 5 wt% Rh/C (30.9 mg, 5 mol%) in methanol

(6.0 mL) with aq. NaOH solution (0.23 mL, 1.5 equiv., 2 N solution) at 60 °C with 5 bar H_2 pressure. The product was isolated after column chromatography (eluent: *n*-pentane/EtOAc 95:5) as colorless liquid (48.0 mg, 0.18 mmol, 59%, 52:48 d.r. by GC-MS). Only the analytical data of the later-eluting diastereomer are given.

^{1}H NMR (500 MHz, 363 K, Dimethyl sulfoxide-*d*6) 7.39 – 7.27 (m, 5H), 5.07 (s, 2H), 3.76 (dt, *J* = 11.8, 6.3 Hz, 1H), 3.47 – 3.37 (m, 1H), 3.31 (q, *J* = 9.8 Hz, 1H), 2.26 – 2.18 (m, 1H), 1.91 (p, *J* = 11.2 Hz, 2H), 1.75 – 1.62 (m, 3H), 1.45 – 1.38 (m, 1H), 1.36 – 1.26 (m, 1H), 1.02 (qd, *J* = 12.3, 5.3 Hz, 1H), 0.91 – 0.77 (m, 4H). **^{13}C{^{1}H} NMR** (126 MHz, 363 K, Dimethyl sulfoxide-*d*6) 153.2, 137.0, 127.8, 127.1, 126.8, 65.1, 56.1, 44.3 (broad), 35.5 (broad, two signals), 29.3, 28.5, 25.4 (broad), 24.9. **HRMS** (ESI) m/z calculated for [$C_{17}H_{23}NO_2Na$] ([M+Na$^+$]) 296.1621, found 296.1621.

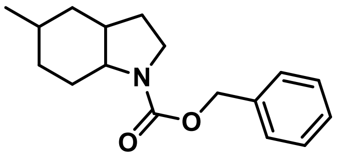

Benzyl 5-methyloctahydro-1*H*-indole-1-carboxylate: The title compound was prepared according to GP-A from (*E*)-4-methyl-2-(2-nitrovinyl)phenol (53.8 mg, 0.30 mmol, 1.0 equiv.), $NaBH_4$ (11.4 mg, 0.30 mmol, 1.0 equiv.) and 5 wt% Rh/C (30.9 mg, 5 mol%) in methanol (6.0 mL) with aq. NaOH solution (0.23 mL, 1.5 equiv., 2 N solution) at 60 °C with 5 bar H_2 pressure. The product was isolated after column chromatography (eluent: *n*-pentane/EtOAc 95:5) as colorless liquid (44.7 mg, 0.16 mmol, 55%, 85:15 d.r. by GC-MS). Only the analytical data of the main diastereomer are given.

^{1}H NMR (500 MHz, 363 K, Dimethyl sulfoxide-*d*6) 7.39 – 7.28 (m, 5H), 5.12 – 5.02 (m, 2H), 3.64 (q, *J* = 4.6 Hz, 1H), 3.50 (ddd, *J* = 11.1, 8.7, 2.6 Hz, 1H), 3.40 (td, *J* = 10.2, 6.9 Hz, 1H), 2.47 – 2.39 (m, 1H), 2.22 – 2.14 (m, 1H), 1.83 (dtd, *J* = 12.2, 9.2, 6.2 Hz, 1H), 1.61 – 1.35 (m, 5H), 0.92 – 0.82 (m, 5H). **^{13}C{^{1}H} NMR** (126 MHz, 363 K, Dimethyl sulfoxide-*d*6) 154.4, 136.9, 127.8, 127.1, 126.9, 65.1, 56.0, 45.3, 37.4, 35.0, 29.1, 29.1, 28.1, 25.3, 21.8. **HRMS** (ESI) m/z calculated for [$C_{17}H_{23}NO_2Na$] ([M+Na$^+$]) 296.1621, found 296.1623.

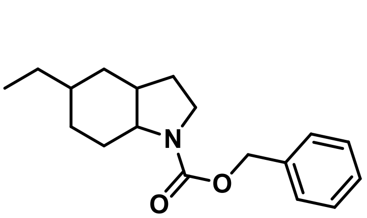

Benzyl 5-ethyloctahydro-1*H*-indole-1-carboxylate: The title compound was prepared according to GP-A from (*E*)-4-ethyl-2-(2-nitrovinyl)phenol (58.0 mg, 0.30 mmol, 1.0 equiv.), $NaBH_4$ (11.4 mg, 0.30 mmol, 1.0 equiv.) and 5 wt% Rh/C (30.9 mg, 5 mol%) in methanol

(6.0 mL) with aq. NaOH solution (0.23 mL, 1.5 equiv., 2 N solution) at 60 °C with 5 bar H_2 pressure. The product was isolated after column chromatography (eluent: *n*-pentane/EtOAc 95:5) as colorless liquid (38.8 mg, 0.14 mmol, 45%, 72:17:11 d.r. by GC-MS). Only the analytical data of the main diastereomer are given.

^{1}H NMR (500 MHz, 363 K, Dimethyl sulfoxide-*d*6) 7.38 – 7.28 (m, 5H), 5.12 – 5.03 (m, 2H), 3.65 (q, *J* = 4.8 Hz, 1H), 3.49 (ddd, *J* = 11.2, 8.7, 2.9 Hz, 1H), 3.40 (td, *J* = 9.9, 6.9 Hz, 1H), 2.44 – 2.33 (m, 1H), 2.18 (ddt, *J* = 11.7, 8.7, 5.9 Hz, 1H), 1.84 (dtd, *J* = 12.2, 9.0, 6.2 Hz, 1H), 1.66 – 1.58 (m, 1H), 1.57 – 1.40 (m, 3H), 1.28 – 1.17 (m, 3H), 0.92 – 0.82 (m, 5H). **^{13}C{^{1}H} NMR** (126 MHz, 363 K, Dimethyl sulfoxide-*d*6) 154.4, 136.9, 127.8, 127.1, 126.9, 65.1, 56.4, 45.3, 37.4, 35.9, 32.6, 29.2, 28.7, 25.6, 25.2, 10.6. **HRMS** (ESI) m/z calculated for [$C_{18}H_{25}NO_2Na$] ([M+Na$^+$]) 310.1778, found 310.1775.

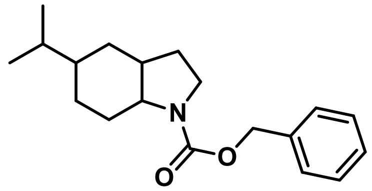

Benzyl 5-isopropyloctahydro-1*H*-indole-1-carboxylate: The title compound was prepared according to GP-A from (*E*)-4-isopropyl-2-(2-nitrovinyl)phenol (62.2 mg, 0.30 mmol, 1.0 equiv.), $NaBH_4$ (11.4 mg, 0.30 mmol, 1.0 equiv.) and 5 wt% Rh/C (30.9 mg, 5 mol%) in a mixture of H_2O (3 mL) and THF (3.0 mL) at 80 °C with 10 bar H_2 pressure. The product was isolated after column chromatography (eluent: *n*-pentane/EtOAc 95:5) as colorless liquid (65.4 mg, 0.22 mmol, 72%, 68:20:12 d.r. by GC-MS). Only the analytical data of the main diastereomer are given.

^{1}H NMR (599 MHz, 363 K, Dimethyl sulfoxide-*d*6) 7.38 – 7.28 (m, 5H), 5.12 – 5.02 (m, 2H), 3.64 (d, *J* = 5.4 Hz, 1H), 3.49 (t, *J* = 9.8 Hz, 1H), 3.40 (q, *J* = 9.3 Hz, 1H), 2.43 – 2.34 (m, 1H), 2.22 – 2.14 (m, 1H), 1.90 – 1.82 (m, 1H), 1.61 – 1.55 (m, 1H), 1.55 – 1.47 (m, 2H), 1.45 – 1.37 (m, 2H), 1.15 – 1.08 (m, 1H), 0.98 – 0.90 (m, 2H), 0.90 – 0.83 (m, 6H). **^{13}C{^{1}H} NMR** (151 MHz, 363 K, Dimethyl sulfoxide-*d*6) 154.4, 136.9, 127.8, 127.1, 126.9, 65.2, 56.3, 45.4, 40.6, 37.7, 31.7, 29.8, 29.4, 25.5, 22.8, 19.1, 19.0. **HRMS** (ESI) m/z calculated for [$C_{19}H_{27}NO_2Na$] ([M+Na$^+$]) 324.1934, found 324.1934.

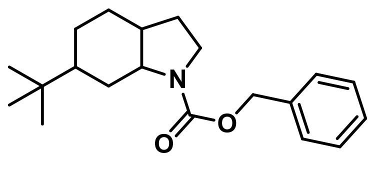

Benzyl 6-(*tert*-butyl)octahydro-1*H*-indole-1-carboxylate: The title compound was prepared according to GP-A from (*E*)-5-(*tert*-butyl)-2-(2-nitrovinyl)phenol (66.4 mg, 0.3 mmol, 1.0 equiv.), $NaBH_4$ (25.0 mg, 0.66 mmol, 2.2 equiv.) and 5 wt% Rh/C (30.9 mg, 5 mol%) in

tetrahydrofuran (3.0 mL) and water (3.0 mL) at 80 °C with 50 bar H_2 pressure. The product was isolated after column chromatography (eluent: *n*-pentane/EtOAc 94:6) as colorless liquid (28.9 mg, 0.09 mmol, 31%, 82:16:2 d.r. by GC-MS). Only the analytical data of the main diastereomer are given.

^{1}H NMR (500 MHz, 363 K, Dimethyl sulfoxide-d_6) 7.37 – 7.34 (m, 4H), 7.30 (ddt, *J* = 5.9, 3.3, 2.1 Hz, 1H), 5.07 (d, *J* = 3.1 Hz, 2H), 3.76 (q, *J* = 4.6 Hz, 1H), 3.50 (ddd, *J* = 11.2, 8.7, 2.6 Hz, 1H), 3.41 (td, *J* = 10.2, 7.0 Hz, 1H), 2.58 – 2.51 (m, 1H), 2.17 – 2.08 (m, 1H), 1.87 – 1.77 (m, 1H), 1.74 – 1.67 (m, 1H), 1.67 – 1.61 (m, 1H), 1.52 – 1.43 (m, 1H), 1.26 – 1.11 (m, 2H), 1.05 – 0.92 (m, 2H), 0.79 (d, *J* = 1.7 Hz, 9H). **^{13}C{^{1}H} NMR** (126 MHz, 363 K, Dimethyl sulfoxide-d_6) 154.4, 136.9, 127.8, 127.1, 127.0, 65.1, 57.3, 45.4, 40.7, 36.9, 31.5, 28.9, 27.0, 26.9, 26.7, 24.4. **HRMS** (ESI) m/z calculated for [$C_{20}H_{29}NO_2Na$] ([M+Na]$^+$) 338.2091, found: 338.2090.

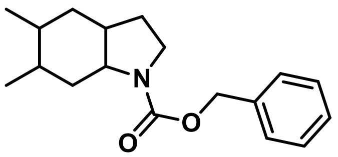

Benzyl 5,6-dimethyloctahydro-1*H*-indole-1-carboxylate: The title compound was prepared according to GP-A from (*E*)-4,5-dimethyl-2-(2-nitrovinyl)phenol (58.0 mg, 0.3 mmol, 1.0 equiv.), $NaBH_4$ (11.4 mg, 0.30 mmol, 1.0 equiv.) and 5 wt% Rh/C (30.9 mg, 5 mol%) in methanol (6.0 mL) at 80 °C with 50 bar H_2 pressure. The product was isolated after column chromatography (eluent: *n*-pentane/EtOAc 9:1) as colorless liquid (17.9 mg, 0.06 mmol, 21%, 94:6 d.r. by GC-MS). Only the analytical data of the main diastereomer are given.

^{1}H NMR (500 MHz, 363 K, Dimethyl sulfoxide-d_6) 7.37 – 7.33 (m, 5H), 5.08 (d, *J* = 1.1 Hz, 2H), 3.73 – 3.67 (m, 1H), 3.42 – 3.28 (m, 2H), 2.24 – 2.15 (m, 1H), 1.89 – 1.49 (m, 6H), 1.40 (dt, *J* = 13.5, 6.4 Hz, 1H), 0.88 (dd, *J* = 7.1, 0.8 Hz, 3H), 0.85 – 0.81 (m, 1H), 0.79 (dd, *J* = 6.8, 0.9 Hz, 3H). **^{13}C{^{1}H} NMR** (126 MHz, 363 K, Dimethyl sulfoxide-d_6) 153.7, 137.0, 127.8, 127.1, 126.9, 65.1, 56.2, 44.8, 36.3, 31.4, 31.2, 30.8, 30.6, 30.4, 29.0, 16.1. **HRMS** (ESI) m/z calculated for [$C_{18}H_{25}NO_2Na$] ([M+Na$^+$]) 310.1778, found 310.1777.

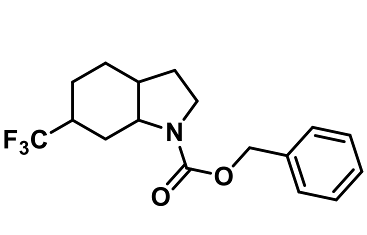

Benzyl 6-(trifluoromethyl)octahydro-1*H*-indole-1-carboxylate: The title compound was prepared according to GP-A from (*E*)-2-(2-nitrovinyl)-4-(trifluoromethyl)phenol (70.0 mg, 0.3 mmol, 1.0 equiv.), $NaBH_4$ (11.4 mg, 0.30 mmol, 1.0 equiv.) and 5 wt% Rh/C (30.9 mg,

5 mol%) in in methanol (6.0 mL) at 60 °C with 10 bar H_2 pressure. The product was isolated after column chromatography (eluent: *n*-pentane/EtOAc 9:1) yellowish oil (67.7 mg, 0.21 mmol, 69%, 50:50 d.r. by GC-MS). Only the analytical data of the main diastereomer are given.

^{1}H NMR (500 MHz, 363 K, Dimethyl sulfoxide-*d*$_6$) 7.66 – 7.56 (m, 5H), 5.39 – 5.36 (m, 2H), 4.16 (dt, *J* = 11.1, 6.4 Hz, 1H), 3.78 – 3.68 (m, 1H), 3.61 (q, *J* = 10.2 Hz, 1H), 2.62 – 2.48 (m, 2H), 2.42 (s, 1H), 2.20 (p, *J* = 11.1 Hz, 1H), 2.13 – 2.00 (m, 3H), 1.99 – 1.91 (m, 1H), 1.66 (qd, *J* = 12.5, 5.0 Hz, 1H), 1.43 (td, *J* = 12.7, 11.0 Hz, 1H).**^{13}C{^{1}H} NMR** (126 MHz, 363 K, Dimethylsulfoxid-*d6*). 153.3, 136.8, 127.9, 127.3 (q, *J* = 278.0 Hz), 127.5, 127.2, 126.9, 65.3, 54.7, 44.4, 38.5 (q, *J* = 26.4 Hz), 35.5, 25.7, 23.4, 18.9 (d, *J* = 2.6 Hz). **^{19}F{^{1}H}-NMR** (470 MHz, 363 K, Dimethyl sulfoxide-*d*$_6$). -72.1 **HRMS** (ESI) m/z calculated for [$C_{17}H_{20}NO_2F_3Na$] ([M+Na$^+$]) 350.1338, found 350.1338.

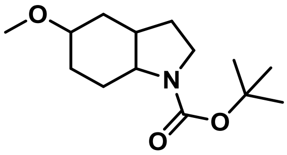

Benzyl 5-methoxyoctahydro-1*H*-indole-1-carboxylate: The title compound was prepared according to GP-A from (*E*)-4-methoxy-2-(2-nitrovinyl)phenol (58.6 mg, 0.30 mmol, 1.0 equiv.), $NaBH_4$ (11.4 mg, 0.30 mmol, 1.0 equiv.) and 5 wt% Rh/C (30.9 mg, 5 mol%) in methanol (3.0 mL) at 80 °C with 10 bar H_2 pressure. The product was isolated after column chromatography (eluent: *n*-pentane/EtOAc 9:1, later 7:3) as colorless liquid (17.2 mg, 0.07 mmol, 22%, 71:29 d.r. by GC-MS). Only the analytical data of the main diastereomer are given.

^{1}H NMR (500 MHz, 363 K, Dimethyl sulfoxide-*d*$_6$) 3.57 (dt, *J* = 8.4, 5.8 Hz, 1H), 3.34 – 3.22 (m, 3H), 3.22 (s, 3H), 2.20 – 2.12 (m, 1H), 1.95 – 1.86 (m, 1H), 1.83 – 1.70 (m, 3H), 1.68 – 1.52 (m, 3H), 1.49 – 1.44 (m, 1H), 1.41 (s, 9H). **^{13}C{^{1}H} NMR** (126 MHz, 363 K, Dimethyl sulfoxide-*d*$_6$) 77.5, 74.5, 55.4, 54.6, 44.7, 35.8, 29.6, 28.2, 27.8, 26.9, 25.5, 21.8. **HRMS** (ESI) m/z calculated for [$C_{14}H_{25}NO_3Na$] ([M+Na$^+$]) 278.1727, found 278.1728.

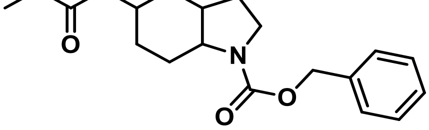

Benzyl 5-(2-methoxy-2-oxoethyl)octahydro-1*H*-indole-1-carboxylate: The title compound was prepared according to GP-A from methyl (*E*)-2-(4-hydroxy-3-(2-nitrovinyl)phenyl)acetate (71.2 mg, 0.30 mmol, 1.0 equiv.), $NaBH_4$ (11.4 mg, 0.30 mmol, 1.0 equiv.) and 5 wt% Rh/C

(30.9 mg, 5 mol%) in methanol (3.0 mL) at 80 °C with 10 bar H_2 pressure. The product was isolated after column chromatography (eluent: *n*-pentane/EtOAc 4:1) as colorless liquid (71.0 mg, 0.21 mmol, 71%, 78:17:5 d.r. by GC-MS). Only the analytical data of the main diastereomer are given.

^{1}H NMR (500 MHz, 363 K, Dimethyl sulfoxide-*d*$_6$) 7.39 – 7.27 (m, 5H), 5.12 – 5.02 (m, 2H), 3.65 (q, *J* = 4.6 Hz, 1H), 3.60 (d, *J* = 0.9 Hz, 3H), 3.50 (ddd, *J* = 11.1, 8.7, 2.6 Hz, 1H), 3.40 (td, *J* = 10.2, 6.8 Hz, 1H), 2.47 – 2.37 (m, 1H), 2.26 – 2.15 (m, 3H), 1.89 – 1.70 (m, 2H), 1.66 – 1.59 (m, 1H), 1.58 – 1.39 (m, 3H), 1.00 – 0.91 (m, 2H). **^{13}C{^{1}H} NMR** (126 MHz, 363 K, Dimethyl sulfoxide-*d*$_6$) 171.9, 154.5, 136.9, 127.9, 127.2, 126.9, 65.2, 56.0, 50.4, 45.3, 40.4, 37.2, 32.4, 31.6, 29.1, 25.7, 25.1. **HRMS** (ESI) m/z calculated for [$C_{19}H_{25}NO_4Na$] ([M+Na$^+$]) 354.1676, found 354.1676.

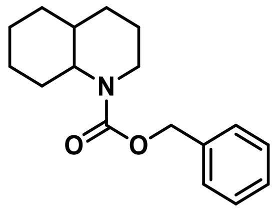

Benzyl octahydroquinoline-1(2*H*)-carboxylate: The title compound was prepared according to GP-B from (*E*)-3-(2-hydroxyphenyl)acrylonitrile (43.6 mg, 0.3 mmol, 1.0 equiv.) and 5 wt% Rh/C (30.9 mg, 5 mol%) in methanol (6.0 mL) at 60 °C with 10 bar H_2 pressure. The product was isolated after column chromatography (eluent: *n*-pentane/EtOAc 19:1) as colorless liquid (29.8 mg, 0.11 mmol, 36%, 65:35 d.r. by GC-MS). Only the analytical data of the main diastereomer are given.

^{1}H NMR (400 MHz, Chloroform-*d*) 7.41 – 7.27 (m, 5H), 5.13 (d, *J* = 3.0 Hz, 2H), 4.25 – 3.91 (m, 1H), 3.65 (ddd, *J* = 13.8, 6.5, 3.1 Hz, 1H), 3.30 (ddd, *J* = 13.5, 10.3, 4.7 Hz, 1H), 3.09 (td, *J* = 11.0, 3.3 Hz, 1H), 2.83 (t, *J* = 13.1 Hz, 1H), 2.15 (dt, *J* = 11.8, 3.5 Hz, 1H), 1.91 – 0.80 (m, 10H). **^{13}C{^{1}H} NMR** (101 MHz, Chloroform-*d*) 156.0, 137.3, 128.6, 127.9, 127.7, 66.7, 53.5, 39.2, 35.2, 31.5, 26.0, 25.8, 24.0, 20.4. **HRMS** (ESI) m/z calculated for [$C_{17}H_{23}NO_2Na$] ([M+Na$^+$]) 296.1621, found 296.1612.

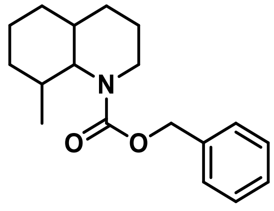

Benzyl 8-methyloctahydroquinoline-1(2*H*)-carboxylate: The title compound was prepared according to GP-B from (*E*)-3-(2-hydroxy-3-methylphenyl)acrylonitrile (47.8 mg, 0.30 mmol, 1.0 equiv.) and 5 wt% Rh/C (30.9 mg, 5 mol%) in methanol (6.0 mL) at 80 °C with 10 bar H_2

pressure. The product was isolated after column chromatography (eluent: *n*-pentane/EtOAc 95:5) as colorless liquid (11.4 mg, 0.04 mmol, 13%, >95:5 d.r. by GC-MS).

^{1}H NMR (599 MHz, Chloroform-*d*) 7.38 – 7.33 (m, 4H), 7.32 – 7.28 (m, 1H), 5.18 – 5.11 (m, 2H), 4.00 (dd, *J* = 13.8, 7.5 Hz, 1H), 3.38 (dd, *J* = 11.7, 4.2 Hz, 1H), 2.99 (ddd, *J* = 13.8, 12.4, 5.3 Hz, 1H), 2.48 (dtd, *J* = 10.1, 5.0, 2.7 Hz, 1H), 1.87 – 1.75 (m, 2H), 1.70 – 1.65 (m, 1H), 1.65 – 1.44 (m, 6H), 1.16 – 1.07 (m, 1H), 0.99 (qd, *J* = 12.0, 4.4 Hz, 1H), 0.93 (d, *J* = 7.2 Hz, 3H). **^{13}C{^{1}H} NMR** (151 MHz, Chloroform-*d*) 156.3, 137.4, 128.6, 127.9, 127.6, 66.8, 64.1, 38.9, 33.7, 31.8, 31.4, 30.3, 26.3, 22.3, 20.7, 11.9. **HRMS** (ESI) m/z calculated for [$C_{17}H_{23}NO_2Na$] ([M+Na$^+$]) 310.1778, found 310.1775.

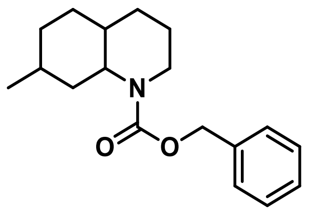

Benzyl 7-methyloctahydroquinoline-1(2*H*)-carboxylate: The title compound was prepared according to GP-B from (*E*)-3-(2-hydroxy-4-methylphenyl)acrylonitrile (47.8 mg, 0.30 mmol, 1.0 equiv.) and 5 wt% Rh/C (30.9 mg, 5 mol%) in methanol (6.0 mL) at 80 °C with 10 bar H_2 pressure. The product was isolated after column chromatography (eluent: *n*-pentane/EtOAc 98:2) as colorless liquid (26.1 mg, 0.09 mmol, 30%, 45:40:15 d.r. by GC-MS).

^{1}H NMR (599 MHz, 363 K, Dimethyl sulfoxide-*d*$_6$) 7.39 – 7.28 (m, 10H), 5.15 – 5.02 (m, 4H), 4.34 – 4.29 (m, 1H), 4.14 – 4.08 (m, 1H), 3.91 – 3.84 (m, 2H), 2.87 – 2.76 (m, 2H), 2.11 – 2.04 (m, 1H), 2.00 (t, *J* = 13.5 Hz, 1H), 1.87 – 1.51 (m, 11H), 1.50 – 1.22 (m, 6H), 1.19 – 1.12 (m, 2H), 1.13 – 1.06 (m, 1H), 1.04 – 0.93 (m, 5H), 0.93 – 0.87 (m, 3H). **^{13}C{^{1}H} NMR** (151 MHz, 363 K, Dimethyl sulfoxide-*d*$_6$) 154.5, 154.1, 136.9, 136.8, 127.8, 127.8, 127.1, 127.1, 126.8, 126.8, 65.5, 65.3, 52.5, 47.6, 38.3, 38.1, 34.2, 33.9, 33.5, 31.4, 30.1, 28.4, 28.2, 27.1, 26.9, 24.8, 24.8, 24.6, 22.9, 22.5, 21.6, 16.8. **HRMS** (ESI) m/z calculated for [$C_{17}H_{23}NO_2Na$] ([M+Na$^+$]) 310.1778, found 310.1775.

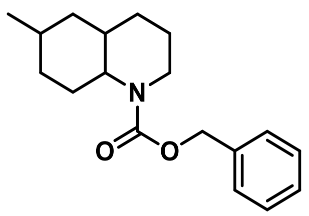

Benzyl 6-methyloctahydroquinoline-1(2*H*)-carboxylate: The title compound was prepared according to GP-B from (*E*)-3-(2-hydroxy-5-methylphenyl)acrylonitrile (47.8 mg, 0.30 mmol, 1.0 equiv.) and 5 wt% Rh/C (30.9 mg, 5 mol%) in methanol (6.0 mL) at 80 °C with 10 bar H_2 pressure. The product was isolated after column chromatography (eluent: *n*-pentane/EtOAc

95:5) as colorless liquid (28.7 mg, 0.10 mmol, 33%, 64:27:6:3 d.r. by GC-MS). The product was obtained as mixture of diastereomers. Only the analytical data of the main diastereomer are given.

^{1}H NMR (400 MHz, Chloroform-*d*) 7.39 – 7.25 (m, 5H), 5.18 – 5.08 (m, 2H), 4.12 (dt, *J* = 12.3, 4.3 Hz, 1H), 4.08 – 3.98 (m, 1H), 2.83 (td, *J* = 13.2, 2.9 Hz, 1H), 1.95 – 1.24 (m, 12H), 1.05 (d, *J* = 7.5 Hz, 3H). **^{13}C{^{1}H} NMR** (126 MHz, Chloroform-*d*) 155.6, 137.3, 128.6, 127.9, 127.8, 66.9, 54.0, 39.8, 37.0, 35.1, 31.0, 27.9, 26.3, 26.1, 21.7, 19.7. **HRMS** (ESI) m/z calculated for [$C_{18}H_{25}NO_2Na$] ([M+Na$^+$]) 310.1778, found 310.1775.

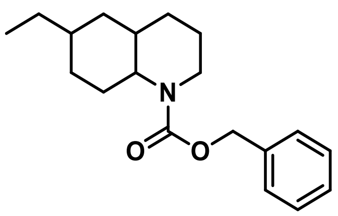

Benzyl 6-ethyloctahydroquinoline-1(2*H*)-carboxylate: The title compound was prepared according to GP-B from (*E*)-3-(2-hydroxy-5-ethylphenyl)acrylonitrile (52.0 mg, 0.30 mmol, 1.0 equiv.) and 5 wt% Rh/C (30.9 mg, 5 mol%) in methanol (6.0 mL) at 80 °C with 10 bar H_2 pressure. The product was isolated after column chromatography (eluent: *n*-pentane/EtOAc 95:5) as colorless liquid (30.2 mg, 0.10 mmol, 33%, 67:33 d.r. by GC-MS). The product was obtained as mixture of diastereomers. Only the analytical data of the main diastereomer are given.

^{1}H NMR (599 MHz, Chloroform-*d*) 7.37 – 7.28 (m, 5H), 5.16 – 5.11 (m, 2H), 4.13 (dt, *J* = 12.3, 4.4 Hz, 1H), 4.02 (ddt, *J* = 13.4, 4.2, 2.0 Hz, 1H), 2.81 (td, *J* = 13.2, 2.9 Hz, 1H), 1.86 – 1.72 (m, 4H), 1.68 – 1.30 (m, 10H), 0.88 (t, *J* = 7.3 Hz, 3H). **^{13}C{^{1}H} NMR** (151 MHz, Chloroform-*d*) 155.6, 137.3, 128.6, 127.9, 127.8, 66.9, 54.1, 39.8, 35.1, 34.8, 33.9, 28.7, 27.9, 27.6, 26.2, 20.2, 13.3. **HRMS** (ESI) m/z calculated for [$C_{19}H_{27}NO_2Na$] ([M+Na$^+$]) 324.1934, found 324.1932.

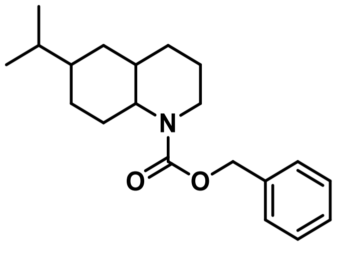

Benzyl 6-isopropyloctahydroquinoline-1(2*H*)-carboxylate: The title compound was prepared according to GP-B from (*E*)-3-(2-hydroxy-5-isopropylphenyl)acrylonitrile (56.2 mg, 0.30 mmol, 1.0 equiv.) and 5 wt% Rh/C (30.9 mg, 5 mol%) in methanol (6.0 mL) at 80 °C with

10 bar H_2 pressure. The product was isolated after column chromatography (eluent: *n*-pentane/EtOAc 95:5) as colorless liquid (23.6 mg, 0.07 mmol, 25%, 71:21:5:3 d.r. by GC-MS). The product was obtained as mixture of diastereomers. Only the analytical data of the main diastereomer are given.

^{1}H NMR (599 MHz, Chloroform-*d*) 7.38 – 7.28 (m, 5H), 5.17 – 5.11 (m, 2H), 4.14 (dt, *J* = 11.9, 4.8 Hz, 1H), 4.03 – 3.98 (m, 1H), 2.82 (td, *J* = 13.0, 2.8 Hz, 1H), 1.85 – 1.73 (m, 3H), 1.72 – 1.59 (m, 6H), 1.58 – 1.50 (m, 1H), 1.39 – 1.31 (m, 2H), 1.21 – 1.14 (m, 1H), 0.91 (d, *J* = 6.7 Hz, 3H), 0.88 (d, *J* = 6.5 Hz, 3H). **^{13}C{^{1}H} NMR** (151 MHz, Chloroform-*d*) 155.6, 137.3, 128.6, 127.9, 127.8, 66.9, 53.6, 39.9, 39.4, 34.9, 32.3, 29.9, 27.6, 27.1, 25.9, 22.2, 21.4, 21.0. **HRMS** (ESI) m/z calculated for [$C_{20}H_{29}NO_2Na$] ([$M+Na^+$]) 338.2097, found 338.2087.

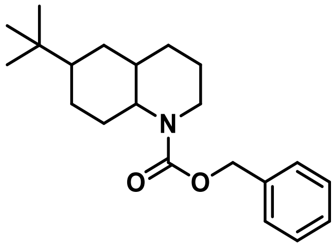

Benzyl 6-(*tert*-buyl)octahydroquinoline-1(2*H*)-carboxylate: The title compound was prepared according to GP-B from (*E*)-3-(2-hydroxy-5-(*tert*-butyl)phenyl)acrylonitrile (60.4 mg, 0.30 mmol, 1.0 equiv.) and 5 wt% Rh/C (30.9 mg, 5 mol%) in methanol (6.0 mL) at 80 °C with 10 bar H_2 pressure. The product was isolated after column chromatography (eluent: *n*-pentane/EtOAc 95:5) as colorless liquid (23.3 mg, 0.07 mmol, 24%, 71:19:6:4 d.r. by GC-MS). The product was obtained as mixture of diastereomers. The diastereomeric ratio was determined by ^{13}C NMR. Only the analytical data of the main diastereomer are given.

^{1}H NMR (599 MHz, Chloroform-*d*) 7.37 – 7.28 (m, 5H), 5.18 – 5.09 (m, 2H), 4.17 (ddd, *J* = 10.6, 6.6, 4.2 Hz, 1H), 3.95 (dt, *J* = 13.2, 3.8 Hz, 1H), 2.92 (ddd, *J* = 13.4, 11.7, 3.6 Hz, 1H), 1.94 – 1.88 (m, 1H), 1.87 – 1.81 (m, 1H), 1.80 – 1.75 (m, 1H), 1.71 – 1.65 (m, 2H), 1.63 – 1.57 (m, 1H), 1.47 – 1.40 (m, 2H), 1.37 – 1.31 (m, 2H), 1.28 – 1.22 (m, 1H), 0.94 – 0.87 (m, 1H), 0.82 (s, 9H). **^{13}C{^{1}H} NMR** (151 MHz, Chloroform-*d*) 155.9, 137.4, 128.6, 127.9, 127.8, 66.9, 50.1, 41.5, 40.5, 32.9, 32.5, 30.8, 28.9, 27.2, 24.0, 23.5, 22.8. **HRMS** (ESI) m/z calculated for [$C_{21}H_{31}NO_2Na$] ([$M+Na^+$]) 352.2247, found 352.2245.

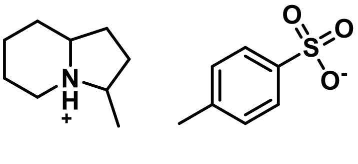

3-Methyloctahydro-1*H*-indolizin-4-ium 4-methylbenzenesulfonate: A 4 mL glass vial equipped with a stir bar was charged with (*E*)-4-(pyridin-2-yl)but-3-en-2-one (44.2 mg,

0.3 mmol, 1.0 equiv.) and Pd/C (32.0 mg, 5 mol%, 5 wt%). The solids were suspended in tetrahydrofuran (1 mL) and water (1 mL). The vial was placed in a 150 mL stainless steel autoclave under air. The autoclave was pressurized and depressurized four times with hydrogen gas before the final hydrogen pressure was set to 50 bar. The mixture was stirred for four hours at 30 °C. After the autoclave was carefully depressurized, 4-methylbenzenesulfonic acid monohydrate (171 mg, 0.9 mmol, 3.0 equiv.) was added and stirring continued for 30 minutes at room temperature. The mixture was filtered over celite using Et_2O as eluent, an aliquot was used for determination of the diastereoselectivity by GC-MS analysis and the solvent was removed *in vacuo*. The residue was purified by column chromatography on silical gel (eluent: CH_2Cl_2/MeOH 95:5, later 90:10) to give the product as off-white solid (95.8 mg, 0.3 mmol, quant., 96:4 d.r. by GC-MS).

^{1}H NMR (599 MHz, Dimethyl sulfoxide-*d*$_6$) 7.49 (d, *J* = 7.9 Hz, 2H), 7.12 (d, *J* = 7.8 Hz, 2H), 3.55 (d, *J* = 11.0 Hz, 1H), 3.27 – 3.18 (m, 1H), 3.09 – 2.99 (m, 1H), 2.69 (t, *J* = 12.7 Hz, 1H), 2.29 (s, 3H), 2.19 – 2.11 (m, 1H), 2.10 – 2.02 (m, 1H), 1.98 – 1.92 (m, 1H), 1.85 – 1.73 (m, 2H), 1.70 – 1.48 (m, 4H), 1.43 – 1.34 (m, 1H), 1.32 (d, *J* = 6.5 Hz, 3H). **^{13}C{^{1}H} NMR** (151 MHz, Dimethyl sulfoxide-*d*$_6$) 145.5, 137.8, 128.1, 125.5, 66.1, 61.6, 48.7, 27.8, 27.5, 26.6, 22.2, 21.8, 20.8, 14.8. **HRMS** (ESI) m/z calculated for [$C_9H_{18}N$] ([M$^+$]) 140.1434, found 140.1432.

6. Gram-Scale Reaction

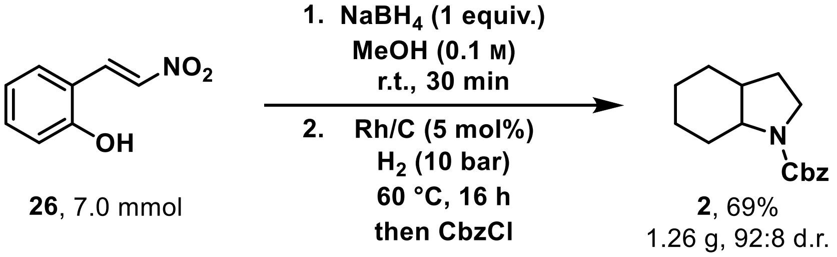

Benzyl octahydro-1*H*-indole-1-carboxylate: In a 110 mL glass vial, (*E*)-2-(2-nitrovinyl)phenol (1.16 g, 7.00 mmol, 1.0 equiv.) was dissolved in MeOH (70 mL). $NaBH_4$ (265 mg, 7.00 mmol, 1.0 equiv.) was added portionwise and the mixture was stirred for 30 minutes at room temperature. Rh/C (720 mg, 5 mol%, 5 wt%) was added, the vial was covered with perforated aluminum and placed in a 400 mL stainless steel autoclave (Berghof BR-300). The autoclave was pressurized and depressurized four times with hydrogen gas before the final hydrogen gas pressure was set to 10 bar. The mixture was stirred for 16 h at 60 °C. After the autoclave was carefully depressurized, NEt_3 (2.93 mL, 21.0 mmol, 3.0 equiv.) and benzyl chloroformate (1.96 mL, 14.0 mmol, 2.0 equiv.) were added, and stirring continued for 30 minutes at room temperature. The mixture was filtered over silica gel using Et_2O as eluent, an aliquot was used for determination of the diastereoselectivity by GC-MS analysis and the solvent was removed *in vacuo*. The residue was purified by column chromatography on silical gel (eluent: *n*-pentane/EtOAc 90:10) to yield the desired product as colorless liquid (1.26 g, 4.86 mmol, 69%, 92:8 d.r.).

The analytical data are in accordance with the data described in chapter 5 for this compound.

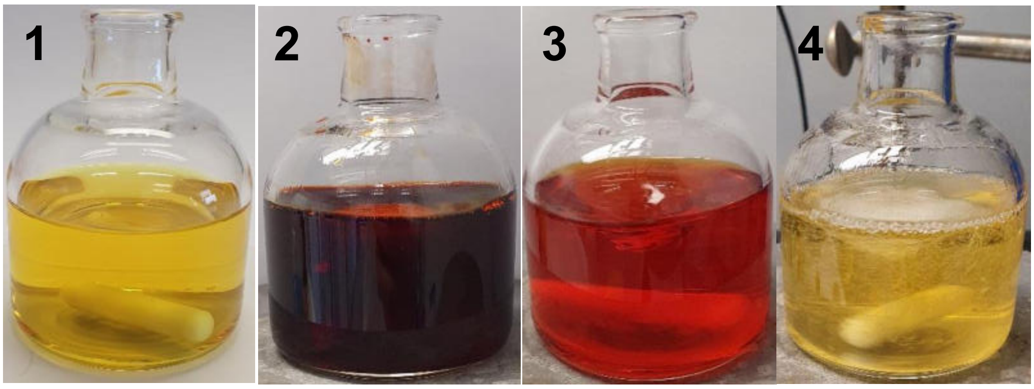

Figure S2: Prereduction of 2-(2-nitrovinyl)phenol with $NaBH_4$. Phenol dissolved in MeOH (**1**) turned dark red after the addition of $NaBH_4$ (**2**), with time the color turned from bright red (**3**) to bright yellow (**4**).

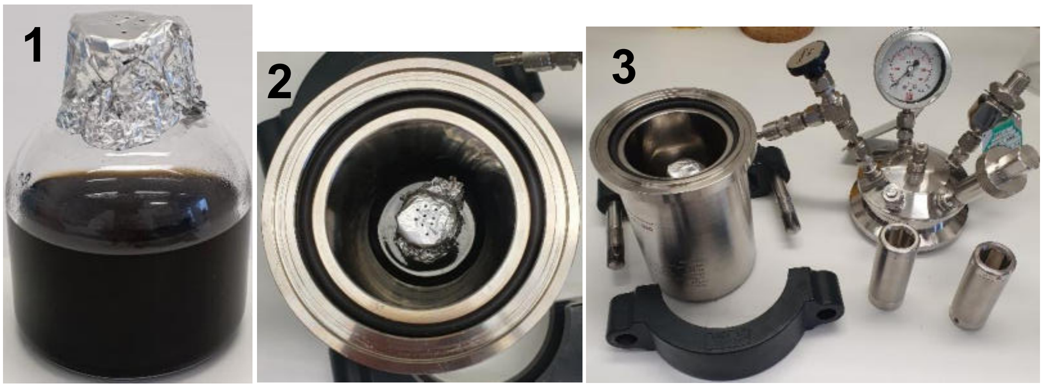

Figure S3: Vial sealed with perforated aluminum foil after addition of Rh/C catalyst (**1**), reaction vial placed in a 300 mL Berghoff High Pressure Autoclave (**2**) and autoclave used for this experiment (**3**).

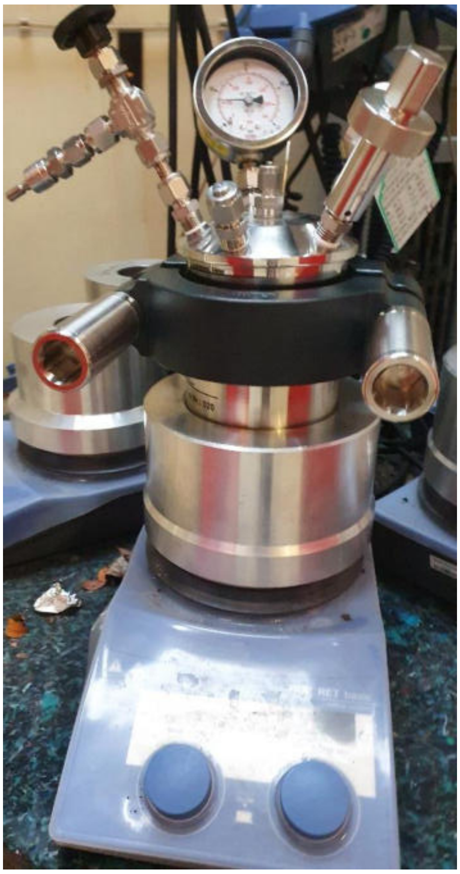

Figure S4: For the catalytic hydrogenation, the autoclave was placed in tightly fitting metal block which was heated to the indicated temperature using a commercially available stirring plate.

7. Mechanistic Investigation and DFT-Calculation

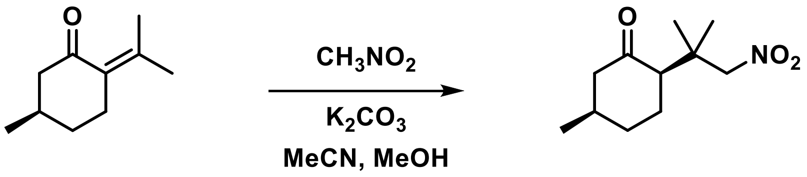

(2*R*,5*R*)-5-Methyl-2-(2-methyl-1-nitropropan-2-yl)cyclohexan-1-one: The title compound was synthesized according to a literature procedure. In an oven-dried screw-cap flask, K_2CO_3 (5.18 g, 50. mmol, 1.5 equiv.) was suspended in MeCN (22 mL) and MeOH (8 mL) followed by the addition of (+)-pulegone (4.1 mL, 25.0 mmol, 1.0 equiv.) and nitromethane (4.0 mL, 75.0 mmol, 3.0 equiv.). The mixture was heated for 22 h at 120 °C, allowed to come to room temperature and then filtered over a plug of silical gel with Et_2O. The crude mixture was purified by column chromatography (eluent: *n*-pentane, later 98:2, 95:5 *n*-pentane/EtOAc) to yield the desired product as colorless liquid (163 mg, 0.77 mmol, 3%, single diastereomer).

R_f (*n*-pentane/EtOAc 90:10): 0.53 (R_f of the *trans*-isomer: 0.57)

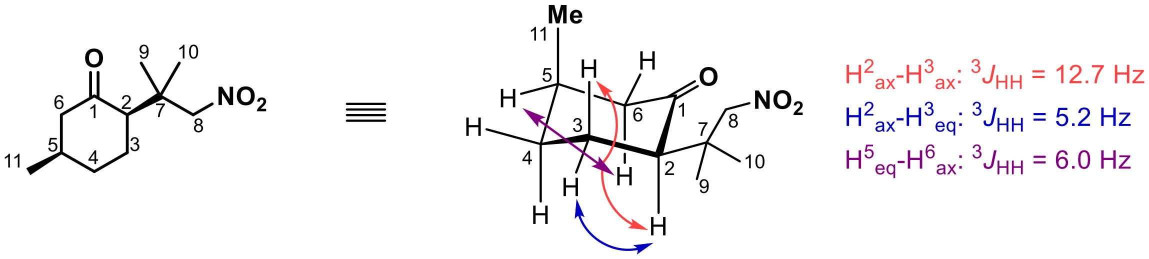

^{1}H NMR (400 MHz, Chloroform-*d*) 5.00 (d, *J* = 10.4 Hz, 1H, H8), 4.29 (d, *J* = 10.4 Hz, 1H, H8), 2.60 (dd, *J* = 13.4, 6.5 Hz, 1H, $H6_{ax}$), 2.53 – 2.40 (m, 2H, H2, H5), 2.11 (ddd, *J* = 12.9, 3.1, 2.0 Hz, 1H, $H6_{eq}$), 2.05 – 1.96 (m, 1H, H3), 1.96 – 1.88 (m, 1H, H4), 1.81 – 1.63 (m, 2H, H3, H4), 1.14 (s, 3H, H9/H10), 1.09 (s, 3H, H9/H10), 0.94 (d, *J* = 7.1 Hz, 3H, H11). **^{13}C{^{1}H} NMR** (100 MHz, Chloroform-*d*) 212.0 (C1), 84.0 (C8), 55.0 (C2), 50.0 (C6), 36.4 (C7), 32.2 (C5), 31.4 (C4), 24.8 (C9/C10), 24.0 (C3), 21.7 (C9/C10), 18.6 (C11). **HRMS** (ESI) m/z calculated for [$C_{11}H_{19}NO_3Na$] ([M+Na$^+$]) 236.1257, found 236.1258.

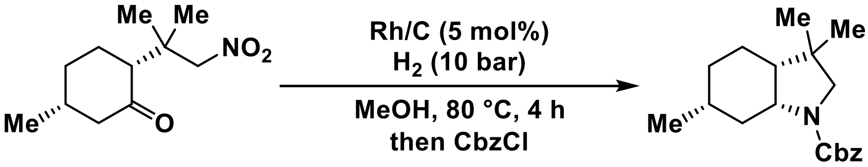

Benzyl (3*R*,6*R*,7*R*)-3,3,6-trimethyloctahydro-1*H*-indole-1-carboxylate: A 20 mL glass vial equipped with a stir bar was charged with (2*R*,5*R*)-5-methyl-2-(2-methyl-1-nitropropan-2-yl)cyclohexan-1-one (64.0 mg, 0.30 mmol, 1.0 equiv.) and Rh/C (30.9 mg, 5 mol%, 5 wt%). Methanol (6 mL) was added and the vial was covered with perforated aluminum foil and placed

in a 150 mL stainless steel autoclave under air. The autoclave was pressurized and depressurized four times with hydrogen gas before the final hydrogen pressure was set to 10 bar. The reaction mixture was stirred at 80 °C for 4 h. After the autoclave was carefully depressurized, NEt_3 (0.9 mmol, 3.0 equiv.) and benzyl chloroformate (0.9 mmol, 3.0 equiv.) were added, and stirring continued for 30 minutes at room temperature. The mixture was filtered over silica gel using Et_2O as eluent, an aliquot was used for determination of the diastereoselectivity by GC-MS analysis and the solvent was removed *in vacuo*. The residue was purified by column chromatography on silical gel (eluent: *n*-pentane/EtOAc 98:2, later 95:5) to yield the desired product as colorless liquid (78.8 mg, 0.26 mmol, 87%, 81:16:3 d.r., >99:1 e.r. for the main diastereomer). Enantiomeric excess of the product was determined after column chromatography using GC-FID analysis.

^{1}H NMR (599 MHz, 363 K, Dimethyl sulfoxide-*d*$_6$) 7.37 – 7.26 (m, 5H), 5.11 – 5.03 (m, 2H), 3.84 (q, *J* = 8.8 Hz, 1H), 3.21 (d, *J* = 10.7 Hz, 1H), 3.14 (d, *J* = 10.8 Hz, 1H), 2.15 – 2.04 (m, 1H), 1.86 – 1.79 (m, 1H), 1.65 – 1.58 (m, 1H), 1.58 – 1.44 (m, 2H), 1.43 – 1.34 (m, 1H), 1.09 – 1.01 (m, 2H), 1.02 (s, 3H), 0.99 (s, 3H), 0.89 – 0.85 (m, 3H). **^{13}C{^{1}H} NMR** (151 MHz, 363 K, Dimethyl sulfoxide-*d*$_6$) 154.8, 137.8, 128.7, 128.0, 127.8, 66.1, 59.8, 58.0, 44.8, 39.9, 36.9, 31.0, 28.2, 27.8, 24.6, 22.3, 21.4. **HRMS** (ESI) m/z calculated for [$C_{19}H_{27}NO_2Na$] ([M+Na$^+$]) 324.1934, found 324.1935.

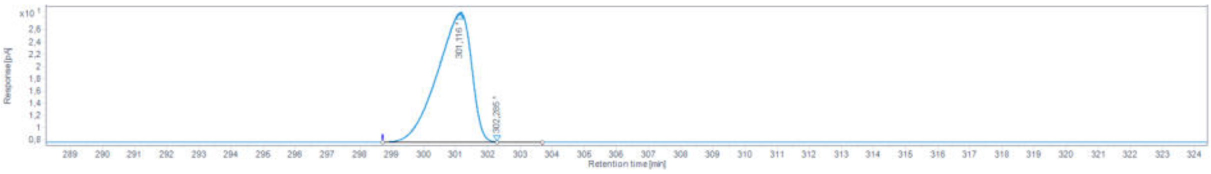

#	Name	Signal description	RT (min)	Area	Area%	Height	Height%	Amount	Concentration	Start time (min)	End time (min)
1		FID1A	301,116	1565,159	99,945	21,239	99,73			298,720	302,279
2		FID1A	302,285	0,854	0,055	0,058	0,27			302,279	303,699

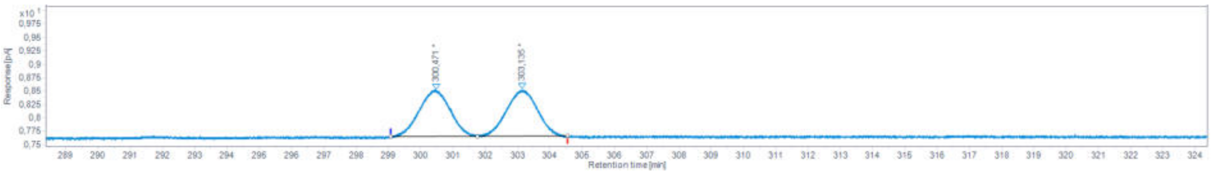

#	Name	Signal description	RT (min)	Area	Area%	Height	Height%	Amount	Concentration	Start time (min)	End time (min)
1		FID1A	300,471	57,614	50,130	0,860	50,05			299,077	301,756
2		FID1A	303,135	57,315	49,870	0,858	49,95			301,756	304,540

Computational investigation of the keto-enol and imine-enamine tautomerism

All computations were carried out using the ORCA 4.2.1 software package.[6] The geometries of all relevant structures were optimized using Handy's range-separated CAM-B3LYP functional[7] and Grimme's D3 dispersion correction[8] in the def2-TZVPP basis set, as developed by Aldrichs and co-workers[9], Weigend's auxiliary basis set def2/J[10], and with the CPCM continuum solvation model[11] (for MeOH). No internal coordinate or symmetric constraints were applied. Optimized geometries were verified to be local minima on the respective potential energy landscape by the absence of negative eigenvalues of the Hessian, as obtained from a harmonic frequency calculation at the same level.

Free enthalpies were calculated from the electronic energy at the stationary point, corrected by zero-point vibrational energy (ZPVE), thermal, enthalpy and entropy corrections, as obtained from the harmonic frequency calculation (see **Table S3**). All geometries were visualized using ChemCraft[12].

Figure S5. Overview and summary of the calculated molecular structures. One enantiomer (*R*) was used representatively to represent the structures. The arrangement of substituents of interest is indicated as either eq (equatorial) or ax (axial).

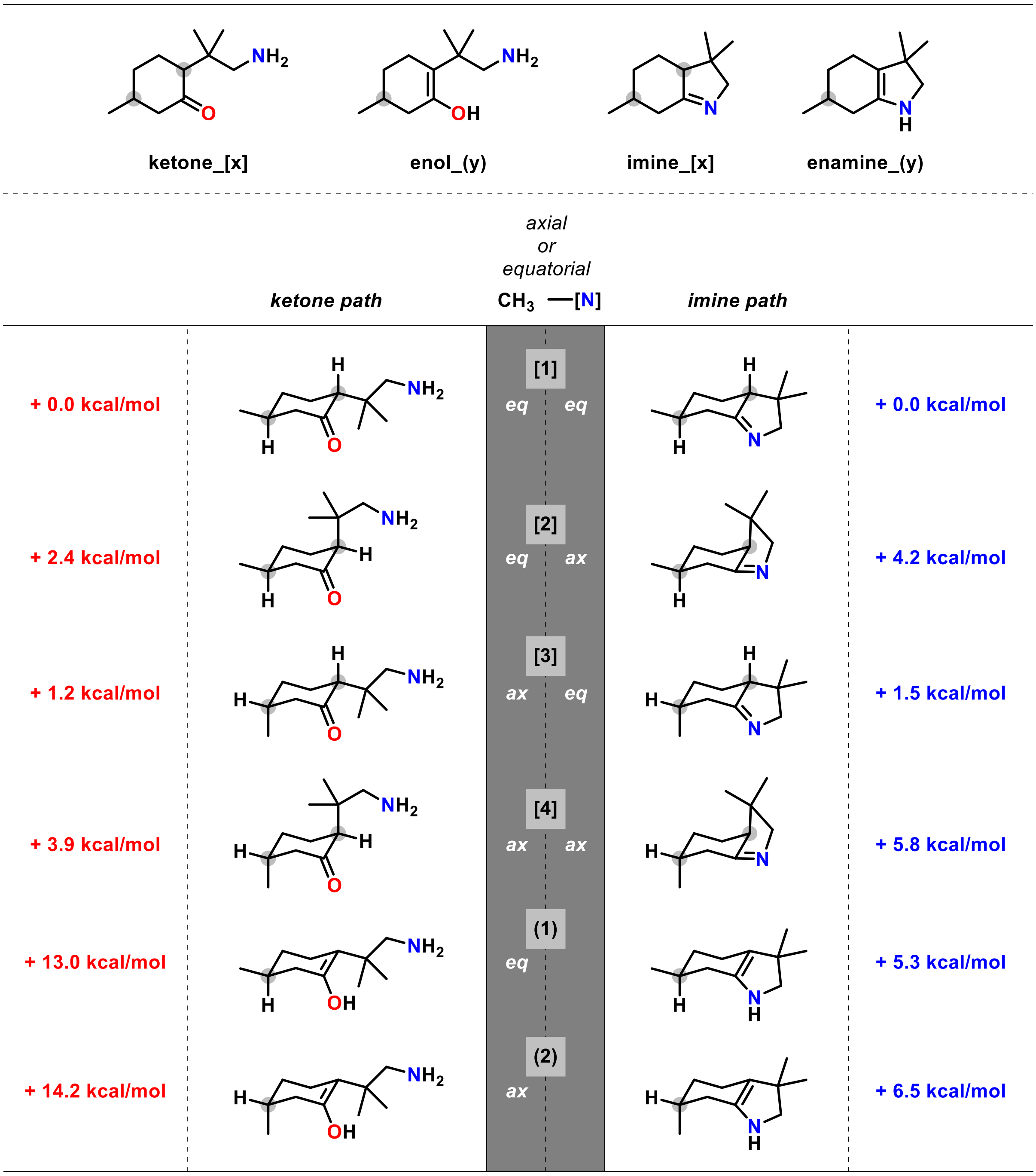

Computational Data:

Table S3. Calculated energies of relevant structures for $T = 80$ °C in methanol.

Comp. No.	Electronic Energy [E_h]	ZPVE [E_h]	Thermal Correction [E_h]	Enthalpy Correction [E_h]	Entropy Correction [E_h]
**01_ketone_1_R**	-561.755867	0.31072958	0.01439451	0.00094421	-0.05424382
**02_ketone_1_S**	-561.756002	0.31077399	0.01438736	0.00094421	-0.05422164
**03_ketone_2_R**	-561.75288	0.31105756	0.01412983	0.00094421	-0.05342263
**04_ketone_2_S**	-561.752909	0.3111118	0.01410639	0.00094421	-0.05337923
**05_ketone_3_R**	-561.754178	0.31086189	0.01433558	0.00094421	-0.05410097
**06_ketone_3_S**	-561.754169	0.31097476	0.01432751	0.00094421	-0.05414568
**07_ketone_4_R**	-561.750823	0.31129746	0.01404483	0.00094421	-0.05326662
**08_ketone_4_S**	-561.75083	0.3112378	0.01405777	0.00094421	-0.05329113
**09_enol_eq_R**	-561.734616	0.31027786	0.01465842	0.00094421	-0.05459216
**10_enol_eq_S**	-561.734624	0.31017772	0.01467342	0.00094421	-0.0545292
**11_enol_ax_R**	-561.733068	0.31045852	0.01461096	0.00094421	-0.05437861
**12_enol_eq_S**	-561.733016	0.31044742	0.01461127	0.00094421	-0.05440231
**13_imine_1_R**	-485.306442	0.28418267	0.01623114	0.00111839	-0.06239195
**14_imine_1_S**	-485.306424	0.28420925	0.01623678	0.00111839	-0.06241416
**15_imine_2_R**	-485.298946	0.28377816	0.01643126	0.00111839	-0.06293029
**16_imine_2_S**	-485.298934	0.28375664	0.01643947	0.00111839	-0.06294624
**17_imine_3_R**	-485.304559	0.28448496	0.01614775	0.00111839	-0.06214946
**18_imine_3_S**	-485.304568	0.2844675	0.01615036	0.00111839	-0.06215729
**19_imine_4_R**	-485.296894	0.28398163	0.01635697	0.00111839	-0.06266143
**20_imine_4_S**	-485.296925	0.28405378	0.01633848	0.00111839	-0.06262778
**21_enamine_eq_R**	-485.29695	0.28369377	0.01670274	0.00111839	-0.06346492
**22_enamine_eq_S**	-485.296939	0.28366071	0.01671226	0.00111839	-0.06348588
**23_enamine_ax_R**	-485.295595	0.28399723	0.01657114	0.00111839	-0.06309226
**24_enamine_ax_S**	-485.295601	0.28402491	0.01656579	0.00111839	-0.06307947

A comparison of the free enthalpies obtained from DFT calculations performed for the keto-enol and imine-enamine tautomerization shows that the enol intermediates are significantly higher in energy in relation to the ketones, than the enamines are to the corresponding imines intermediates. Thus, according to these calculations, the tautomerization of the intermediate imines is more plausible than the tautomerization of the ketones. In connection with the experimental data, it can be concluded that tautomerization, if it has any significant contribution at all, occurs for the imine intermediates. Consequently, the diastereoselectivity of the overall reaction is mainly determined by the hydrogenation of the phenols to the cyclohexanones.

01_ketone_1_R

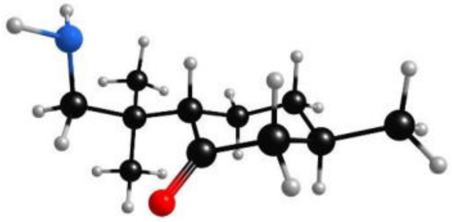

C	-1.66560970758981	-0.11584229855898	0.28178394344015
C	-0.49985136164248	0.84741190309719	0.08456586605682
H	-2.30292168085430	0.23154498103935	1.11031705813755
C	-1.19218571157955	-1.54029633752458	0.56078688531257
H	-2.29738378634886	-0.11833309956825	-0.62433094795444
H	-0.88574766582979	1.85386064151905	-0.12269783940603
C	0.43065437879931	0.40923206698657	-1.06638062113080
C	0.88435156011048	-1.01121142046776	-0.77342598857377
C	1.53883973334312	1.42333287792806	-1.44310795584067
H	-0.20266960009233	0.30233766097108	-1.96305481915912
H	-0.61028578162436	-1.51984568259900	1.49972376633816
C	-0.24174176491157	-1.99628973104873	-0.55603876004750
C	-2.34875765085377	-2.51543535997593	0.73589309282550
H	-0.81071786085633	-2.07681971663050	-1.49867109533645
H	0.19778633157687	-2.97887244453410	-0.33470341219595
O	2.04353345148877	-1.36662515420939	-0.69318076472393
C	2.32466617787539	0.91215688739752	-2.66926935078457
C	2.52867752144875	1.65832377404958	-0.29712716956987
C	0.89339739018071	2.76657156110428	-1.81801696235773
H	3.02232357839346	0.72395748764202	-0.00207523571684
H	2.02546793524856	2.07965081563716	0.58570908196746
H	3.30340233508469	2.37726373496876	-0.60687534113402
H	1.65367760019406	3.44436586595317	-2.23667320181482
H	0.45200299689859	3.26941569722044	-0.94640969621357
H	0.09804681072362	2.64128365553437	-2.56906451866083
H	-2.95694809078580	-2.56643295480061	-0.18181341239579
H	-3.00746741206475	-2.20324535057663	1.56048831338098
H	-1.98606051319092	-3.53093729637395	0.95605580631614
H	0.07697395561671	0.91751668209066	1.02149470671382
H	3.10152620049438	1.67004907432157	-2.89710404170883
N	1.46808425028253	0.61191071992495	-3.80933812740500
H	2.85259606060360	-0.00685627671536	-2.38730028647154

H	2.00521135428835	0.12062887059651	-4.52194674914764
H	1.15441896557267	1.47407816560148	-4.25381222273942

02_ketone_1_S

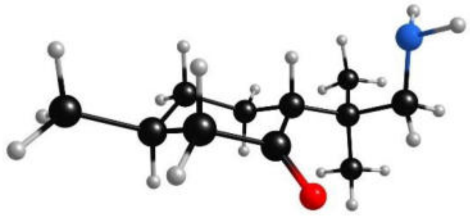

C	-1.50895982806861	-0.13352957565212	-0.30191787700954
C	-0.30362306388058	0.77843751504001	-0.33795654062500
H	-2.33301583199352	0.40342900010439	0.18826289914060
C	-1.20146020257863	-1.46073887658654	0.40687278239127
H	-1.80645715607803	-0.34552814150961	-1.34376241697372
O	-0.35984256427853	1.89965792386365	0.12694842343095
C	0.93396160230904	0.16127011760666	-0.96786128786920
C	1.23236012182530	-1.15404321994637	-0.21752338134768
C	2.14929671084597	1.10062603783017	-1.16521018572267
H	0.59925056168488	-0.12106330813812	-1.98013821927168
H	-0.95987025422069	-1.22343591697665	1.45852749539626
C	0.03667776638536	-2.10060443345266	-0.21644373540713
C	-2.40847469264313	-2.38922114671481	0.39096582033505
H	-0.19677842513629	-2.39828680034089	-1.25424284423807
H	0.29017101507011	-3.02406637198453	0.32788548471708
H	2.09360191273296	-1.65063079334451	-0.68281330328884
C	3.25779789506890	0.34692231165478	-1.91671241071944
C	2.71648317480395	1.60752471140622	0.16500375052278
C	1.73891888274258	2.32082553803164	-2.01674195757281
H	1.52108948514970	-0.93072864854864	0.82281481625000
H	3.07692561574542	0.77589542780326	0.78838002892368
H	1.95964230799221	2.16377607302038	0.73221353863325
H	3.57322596139275	2.27498330755232	-0.01795210531983
H	2.64041363820118	2.95668546000907	-2.12967816193948
H	1.00543473522759	2.90944109904812	-1.45262327164929
N	1.13862260352405	1.94990524830960	-3.29195456603240
H	3.71342337401943	-0.43934415071630	-1.29893162866658
H	4.05994395323262	1.04553258076336	-2.20165007069027
H	2.87783137020781	-0.12805630571429	-2.83456046520564
H	1.85514154089355	1.62293620028472	-3.93931433121595
H	0.73264968074956	2.77402261679841	-3.73212928800490
H	-2.68418019140842	-2.65109805621288	-0.64350544419757
H	-3.28318597474527	-1.91648377307676	0.86287860617454
H	-2.19475572477322	-3.32447165021105	0.93039984705222

03_ketone_2_R

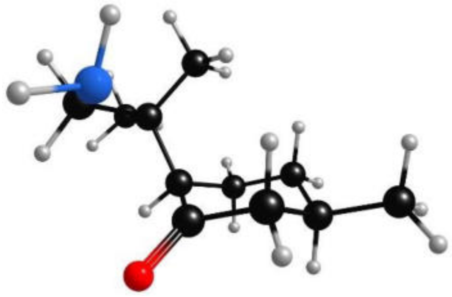

C	-1.75479535910048	-0.70298856663497	0.45366414548200
C	-0.83440730993888	0.43278792134956	0.00600117950629
H	-2.53792850028243	-0.29201607370111	1.11060853285580
C	-0.99607569888288	-1.80575553052209	1.18929007768875
H	-2.27647798930710	-1.14917129518582	-0.40680214242724
H	-1.40538056548102	1.18537328664824	-0.55771680670496
C	0.40424571505059	0.00024570040950	-0.80960127686269
C	1.04469324411387	-1.22070656286559	-0.15869066828743
C	0.19689418731627	-0.09131979496882	-2.35931418847982
H	-0.53264202425965	-1.35782290894395	2.08706383791048
C	0.13419980903428	-2.33241360670313	0.29453213070669
C	-1.91371707076535	-2.93515055595214	1.63867003405492
H	-0.29664146398510	-2.80326106607298	-0.60210027871261
H	0.74534307231544	-3.08441758909458	0.81390570673572
O	2.23408016519637	-1.22863984886946	0.09673868021490
H	1.17240758023969	0.77697807628621	-0.68322824068198
C	-1.09638077169715	-0.79500692660181	-2.78144965451679
C	1.40095199692019	-0.81225203422228	-2.99020791087068
C	0.17113301379738	1.34059237096260	-2.90708187226162
H	-1.16329256126908	-1.81987459070784	-2.39403689222794
H	-1.14336423698799	-0.84904715362627	-3.88081004714114
H	-1.98432237032297	-0.24263874230073	-2.44366654676927
H	-0.62163442586269	1.93939198707862	-2.43541212239960
H	-0.01526510317474	1.33699256435342	-3.99176886951206
H	1.13156007961879	1.84835233671410	-2.72792747485147
H	-2.39508346560733	-3.41241957705781	0.76954917382809
H	-2.70872953207593	-2.56061715912342	2.30148629666297
H	-1.35509311533674	-3.71240444379645	2.18206387094458
H	-0.47193391348811	0.94695104508389	0.91114897274330
N	1.40328114181744	-2.23832231878655	-2.70668996142330
H	0.79276093002349	-2.73622091174611	-3.35228830803577
H	2.33520924746944	-2.62662384799159	-2.83677222879814
H	2.32235828140655	-0.37732619937891	-2.57260450591600
H	1.40813701350583	-0.58496798403172	-4.07431264245395

04_ketone_2_S

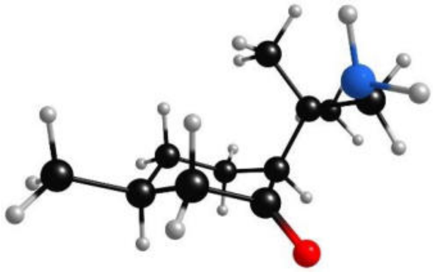

C	-1.61964680148289	-0.36966803349713	0.53801393446101
C	-0.44566799844267	0.46988063687559	0.10567743598880
H	-2.27139021851509	0.25260036775468	1.16791609293755
C	-1.16462752554888	-1.63751055914987	1.27358137646082
H	-2.18440684901465	-0.65130140232329	-0.36361242369740
O	-0.31122686206737	1.61831722722591	0.48382746062376
C	0.65915116543807	-0.21370036094622	-0.69206048540796
C	1.00486466033431	-1.57097059809665	-0.04013801437482
C	0.45368265364382	-0.24019956472718	-2.24443919481059
H	-0.61793562264497	-1.32178811468925	2.18066374118991
C	-0.19392211365658	-2.41732521376774	0.38898138381869
C	-2.35697214322008	-2.48161865009393	1.70413433895687
H	-0.74273109827742	-2.79493356410175	-0.48729094281627
H	0.16952891249097	-3.30431179870839	0.93206022898191
H	1.65804856939401	-2.14891263542268	-0.71058244804934
H	1.60666999439623	-1.36222569043987	0.85944640400636
H	1.51961004479777	0.45461234299367	-0.54253873626555
C	-0.40896806959116	-1.40208383375008	-2.74631676324868
C	1.83940667339147	-0.34987599678662	-2.89168888670733
C	-0.16962207657165	1.09222240069889	-2.69571642346478
H	2.37643146561968	-1.24551442027611	-2.54694368475403
H	2.45715259121332	0.52934100419914	-2.65151222425913
H	1.75151458198465	-0.41560870834339	-3.98682493099027
H	0.06372007303328	-2.37265568516484	-2.54102557359388
H	-0.53294857878244	-1.32296613273852	-3.83816837086871
H	-1.41029494281181	-1.40762203231654	-2.29665171620174
N	-1.56741129936843	1.20814683551496	-2.31230211600902
H	-0.00886819616237	1.19486615764353	-3.78689034127009
H	0.38698842555607	1.91367429823185	-2.21829377050015
H	-2.16663679338657	0.72722330866738	-2.98096567380945
H	-1.85869541678121	2.18350271402684	-2.31650041914904
H	-2.93288159709074	-2.81548881260656	0.82564586249219
H	-3.03724463103721	-1.91081657220490	2.35445462280486
H	-2.03103097683943	-3.37741891368091	2.25454025752555

05_ketone_3_R

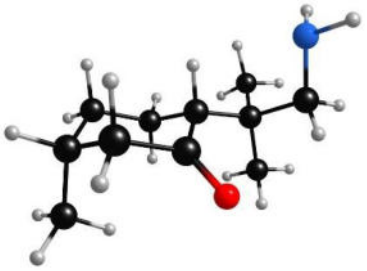

C	-1.58720356439693	-0.23515288573507	-0.01519308097064
C	-0.36894148206712	0.65577878235513	-0.12345736563157
H	-2.36355347819320	0.30753925172976	0.54159426860006
C	-1.24030413236169	-1.57558677713782	0.65467243404869
H	-1.95785482058137	-0.42333456231779	-1.03607766232672
O	-0.36133412381291	1.76174342815034	0.38031555039424
C	0.79756657645618	0.03837330688624	-0.87854921559252
C	1.13930140023483	-1.31250284339419	-0.21298302273969
C	2.01044252776348	0.96375980259038	-1.14647736938872
H	0.36870861974504	-0.19698003709086	-1.86725072865427
C	-0.94395066892163	-1.39005223318830	2.14188741164927
C	-0.07831765627358	-2.22703882560806	-0.10112197955669
H	-2.12077795259933	-2.23150291657780	0.56514144885848
H	-0.41813979091472	-2.49367333634754	-1.11591847067184
H	0.20679031242354	-3.16864920058881	0.39419136633808
H	1.91622172729274	-1.81766164759402	-0.80116724705930
C	3.04253545342861	0.21592252586366	-2.00459755674771
C	2.69061842042342	1.42242421661829	0.14821611063440
C	1.55553077056197	2.21530229725190	-1.92726297125928
H	1.57248487646299	-1.13727848163440	0.78412725477409
H	3.08198929411913	0.56711603648879	0.71855378935103
H	1.99219710058836	1.97597728452969	0.78814796418832
H	3.54376141795556	2.07904908394092	-0.08386973703975
H	2.45565566570905	2.84208106569884	-2.09081356700827
H	0.87704927842920	2.79642757455139	-1.29110469821382
N	0.85402464467770	1.89145592504468	-3.16297998108064
H	3.53339371844629	-0.59311981083287	-1.44620042750821
H	3.83023068493230	0.91099192981092	-2.33482300557657
H	2.58264549496332	-0.22856056370530	-2.90090451892478
H	1.51342118184585	1.57062078958658	-3.87127483499708
H	0.43274806518397	2.73502745775875	-3.54876795720480
H	-0.10232208633982	-0.70083449620343	2.31358137248552
H	-0.68823580788231	-2.35057000555384	2.61429018951845
H	-1.81753166729894	-0.97480213534618	2.66660623731224

06_ketone_3_S

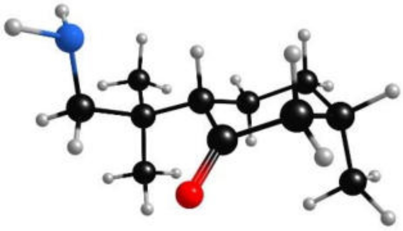

C	-1.63622398770081	-0.26773157604879	0.02406864478700
C	-0.46886061897473	0.70184972088132	-0.14542622663883
H	-2.42174811806826	0.19859197049271	0.63951268915218
C	-1.21268063867563	-1.60105897021677	0.64731950334886
H	-2.08355366728577	-0.46309659963263	-0.96495207758845
H	-0.08907874886849	1.01131352052339	0.84083648625595
C	0.67962220013408	0.09517337300522	-0.98053107401262
C	1.06847461288723	-1.23559517812802	-0.35602382942622
C	1.84070231763905	1.06757180037345	-1.30521774270593
H	0.24413233391566	-0.19427911517501	-1.95179839682204
C	-0.80263897302159	-1.46165713258810	2.11243221187010

C	-0.07743001172770	-2.21014626442239	-0.19283219180505
H	-2.07002963583790	-2.29121163815899	0.60108070856554
H	-0.45828454666693	-2.45253476090733	-1.19842857503897
H	0.31452823311716	-3.13081337775061	0.26111187742884
O	2.18978928045366	-1.52834943718209	0.00989135251826
C	2.85147712092863	0.38310369138340	-2.25006902509178
C	2.58747287135567	1.52494660806800	-0.04731118995492
C	1.28235942344631	2.30696268747586	-2.02164775687072
H	3.01801610392240	0.67108693626635	0.49042450401472
H	1.92012600631416	2.06753244703502	0.63851321673992
H	3.40451807391527	2.21118948316100	-0.32016084262605
H	2.11050690708729	2.92710999467618	-2.39878531856455
H	0.68281861575380	2.93571506192176	-1.34897056392096
H	0.64564974104355	2.03197705161234	-2.87689892657572
N	2.23445848369483	-0.13453215758980	-3.46476459056472
H	3.31475445214817	-0.45640114467838	-1.71764859351729
H	3.65750937341832	1.11758529135121	-2.45172212277755
H	0.08624521934466	-0.82310434567310	2.23486251402592
H	-0.56099304002022	-2.44350943785363	2.54659887550175
H	-1.61518990335911	-1.01744810425820	2.70709012412450
H	-0.82828713701144	1.61515940685786	-0.63685960232642
H	2.00569927713184	0.62999679508797	-4.09928292918840
H	2.90450837956682	-0.71251659990919	-3.96961113231635

07_ketone_4_R

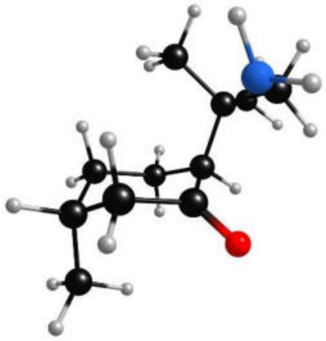

C	-1.69179046774055	-0.34801431094483	0.52815899821604
C	-0.49138845659116	0.46657832861652	0.11698848606939
H	-2.35009001196362	0.29235910386759	1.13244949937875
C	-1.26547902321294	-1.61169439890389	1.29280097247668
H	-2.23191583295628	-0.62937658840033	-0.38648590696480
O	-0.33631014893896	1.61175037605247	0.49774121348154
C	0.61106366530273	-0.24024627979151	-0.66480619657230
C	0.92320921642977	-1.61015147360332	-0.01910747496681
C	0.42413017358269	-0.25903877831520	-2.22013431187017
C	-0.68355211591085	-1.26801118861352	2.66318869125057
C	-0.29993003198026	-2.41899627570932	0.41906121545384
H	-2.16783113838265	-2.22236165911880	1.45626986005140
H	-0.84854904984529	-2.78517098709259	-0.46039767912300
H	0.03852174330176	-3.31279956816142	0.96720845450083
H	1.54112080205949	-2.20941764467959	-0.70389232103233
H	1.55647048736663	-1.42828672669222	0.86300888975227
H	0.19800712903235	-0.61237021821850	2.58729342009550
H	-0.37607944070164	-2.17847341518717	3.19993428810781
H	-1.42680576390731	-0.74330824824270	3.28257259711233

H	1.48234008417922	0.41083964086437	-0.50275565974638
C	-0.45752873562274	-1.40169456251330	-2.73309008881545
C	1.81493474074041	-0.39776870843687	-2.85057260082092
C	-0.16409387560580	1.08723278556664	-2.67759087308462
H	2.32894876227322	-1.30494765536260	-2.50095799823778
H	2.44805325018750	0.46790557424174	-2.60142363527354
H	1.73873409281812	-0.46009216385907	-3.94675181017754
H	-0.00983461917724	-2.38267467962566	-2.52121846584313
H	-0.56516733483990	-1.32080520956887	-3.82650313982646
H	-1.46475506760333	-1.38414271655835	-2.29701759415739
N	-1.56420382534604	1.23304769999993	-2.31356009166484
H	0.01414754823238	1.18765159487107	-3.76622197426815
H	0.40370362649318	1.89569079462557	-2.19130322293857
H	-2.16427310672413	0.76698901820180	-2.99189654361926
H	-1.83411727494876	2.21450854069196	-2.31893899694352

08_ketone_4_S

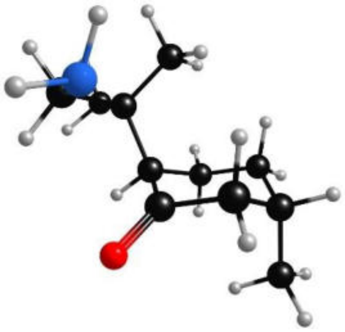

C	-1.80467913687966	-0.75168468248277	0.42803386192013
C	-0.94347122064053	0.43608844849323	-0.00762543573871
H	-2.63230655501143	-0.38367880898806	1.05533804801419
C	-1.00949683734334	-1.81465047899711	1.19320786362484
H	-2.26867623745022	-1.22669672101905	-0.44805977435196
H	-1.54329803971868	1.12861844981081	-0.61630209878677
C	0.35307193137015	0.06781060152370	-0.76552314258110
C	1.03240632922123	-1.11999389799535	-0.09166407801564
C	0.21357365489191	-0.03360238554504	-2.32273170515287
C	-0.53240417933915	-1.33123895631091	2.56185415467969
C	0.16554257898013	-2.28323110643677	0.31982262529677
H	-1.66983607600333	-2.68143765746993	1.35601773374294
H	-0.21496911018986	-2.75460165137235	-0.59701328877972
H	0.79816066748784	-3.01362019309099	0.84412157162819
O	2.20949887469772	-1.06292699226425	0.21112512375543
H	1.07371860682279	0.88366222885386	-0.60915041501487
C	-1.02245983941132	-0.80623240644630	-2.79320856058204
C	1.47872954239130	-0.68735112435751	-2.90539541856923
C	0.13212452000826	1.39529247547625	-2.87288635828331
H	-1.04762048783461	-1.83387427197399	-2.40822799945286
H	-1.02522267891614	-0.86100298881834	-3.89352903265859
H	-1.95153753332355	-0.30407512756864	-2.48940450764381
H	-0.70916005085853	1.95064177730471	-2.43322910252688
H	-0.01153071053361	1.38240227699289	-3.96398028003352
H	1.05637690100539	1.95346529388743	-2.65715403398463
H	-0.65674525841878	1.00748866882867	0.88875807988047
H	0.17763038358432	-0.49327116642014	2.48164191113367

H	-0.02103629302301	-2.14034620944362	3.10518267797665
H	-1.37979819696574	-0.99179397217621	3.17689495316342
N	1.54749435510378	-2.11165598899312	-2.62147890155095
H	2.35811720079858	-0.20367639335148	-2.45230418092379
H	1.51593736824815	-0.45821786432150	-3.98848689350075
H	0.98945089573353	-2.64074866550142	-3.28937139580740
H	2.50332463151642	-2.44876050982671	-2.71604200087694

09_enol_eq_R

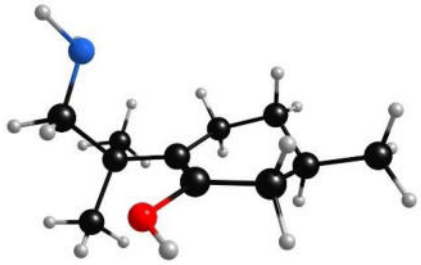

C	-1.79790788011953	-0.29524826528332	-0.35340766453910
C	-0.77596126411528	0.81900141498144	-0.54256067099702
H	-2.74284941289599	0.12300611662687	0.02845388610340
C	-1.27820337109155	-1.38141221763525	0.57963423541648
H	-2.02682350077054	-0.75645320242883	-1.33090631885162
H	-1.08376093605438	1.43826073387288	-1.39724023392302
C	0.63667906168198	0.33360761437692	-0.78972100896663
C	0.92211835309100	-0.96539472975971	-0.60061377435145
C	1.68075771115829	1.34437924219454	-1.29272496491695
H	-0.97479578642187	-0.89635737009835	1.52502269250361
C	-0.03163900466889	-1.98983217555553	-0.05195408562224
C	-2.32556351507748	-2.44061258693374	0.89532310300028
H	-0.32253518325568	-2.68372441772159	-0.86308889269221
H	0.51219507699421	-2.60278181412690	0.68934268587893
O	2.14922918111921	-1.48323807865764	-0.93169794255757
C	2.07743435156689	1.00708250282825	-2.74477773683754
C	2.94112131695690	1.30915939859156	-0.41255222191329
C	1.15721955870422	2.78879528653045	-1.25351225274332
H	3.39844587086554	0.31371624292501	-0.40358631234564
H	2.68944755713773	1.58117887372802	0.62411765960561
H	3.68546919596045	2.03430864142484	-0.77858652266235
H	1.95039062286441	3.47178421340714	-1.59508849460073
H	0.87716165358858	3.08825988698778	-0.23322824110188
H	0.28261884552188	2.94603746760978	-1.89782461749647
H	-2.68272306061747	-2.92350226538036	-0.02898556840848
H	-3.19707746408873	-1.99874238547275	1.40194543072728
H	-1.91690073436157	-3.22766515173853	1.54797995670478
H	-0.79731445752449	1.49185400665079	0.33355139104421
H	2.91062967747956	1.68135056356989	-3.03457278271266
N	0.94791873367278	1.06959984023147	-3.66364425399533
H	2.47734229589511	-0.01580289099257	-2.75989868797572
H	1.21570510116904	0.66375329212665	-4.55924538482837
H	0.71841894510587	2.04102917692250	-3.87044126141862
H	2.19378246052977	-2.40329696380175	-0.63970114452632

10_enol_eq_S

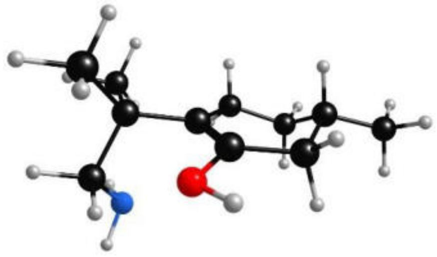

C	-1.38776165525900	-0.11253269092986	0.26317553443078
C	-0.21562586376620	0.73555540536060	-0.14597123998458
H	-1.77372527225164	0.27460668508768	1.22365851507733
C	-1.04894083548658	-1.59320920916656	0.38939608590075
H	-2.20626065101209	0.02416214269131	-0.46846958028202
O	-0.43986167062680	2.08259905781568	-0.00768597866339
C	0.94683216063857	0.26622516012704	-0.62888385812894
C	1.08655527379392	-1.21753083067728	-0.89520335026921
C	2.13471329184280	1.18516195388341	-0.96030254205459
H	-0.41307920431070	-1.71855811086007	1.28441657842023
C	-0.22316790728804	-1.99325280440629	-0.82651923435771
C	-2.29854293048450	-2.44599289198168	0.56200793530864
H	-0.82011345353640	-1.79210552003191	-1.73416871690071
H	-0.01816417632237	-3.07562299450399	-0.81583170229498
H	1.52400811205700	-1.35130165224763	-1.89497738494991
C	3.39785321531556	0.39164625280855	-1.32983592880718
C	2.50060380793140	2.05733351145513	0.25215693294637
C	1.77262399307114	2.10472418236427	-2.14483278479581
H	1.81476269458888	-1.66032176140157	-0.19205697723711
H	2.79819682546190	1.42331347566780	1.10153856484818
H	1.65642514478803	2.67974851577552	0.56886104045536
H	3.35072591920173	2.71513928358547	0.01022857790040
H	2.60410291241682	2.82833205496594	-2.27855626140054
H	0.88555621330599	2.68869413063820	-1.86506622452513
N	1.46498785092782	1.35962466041466	-3.35895841769192
H	3.71434971388583	-0.26830657479839	-0.50929692438578
H	4.22207822596276	1.09385732996909	-1.52952285241162
H	3.26633862260767	-0.23043332077402	-2.22455924756703
H	2.32456062706973	1.01892405770779	-3.78802554414287
H	1.05531438735021	1.98817161747713	-4.04862716523102
H	-2.94504089917908	-2.37540743723896	-0.32800330930167
H	-2.88979765432598	-2.12069311993249	1.43208612322709
H	-2.03907370373561	-3.50594859553490	0.70565650824621
H	-1.27388311463279	2.21836803669031	0.46108282862236

11_enol_ax_R

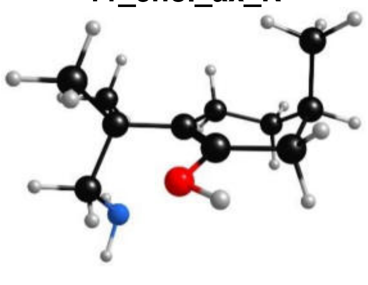

C	-1.52218005577528	-0.07339133967349	0.52050745500040
C	-0.34443335455049	0.72399235334006	0.02789785175896
H	-1.85818159073690	0.36984110035440	1.47537311592740
C	-1.21107536225886	-1.55743061210820	0.70756369699443
H	-2.36101447211056	0.05641792394599	-0.18625642942784
O	-0.52110253622907	2.08112054644924	0.13660507096024
C	0.77789956091644	0.20759495636096	-0.49969364678315
C	0.87148611845762	-1.28820905590307	-0.70681854486570
C	1.96990537479860	1.08233171761293	-0.92262605167776
C	-0.44809749666153	-1.82207564485471	2.00447195071344
C	-0.44902382158995	-2.03050547746957	-0.52899426797811
H	-2.16719018091017	-2.10266667496404	0.75949718662424
H	-1.08232451292879	-1.85756888728875	-1.41532070271727
H	-0.26437089249160	-3.11531033402058	-0.47741168616490
H	1.24274794049664	-1.47753015177289	-1.72434005035583
C	3.18606652223259	0.24298393558325	-1.34413877154486
C	2.43008735105179	1.97104571538137	0.24481856995178
C	1.56576178672301	1.98436842865774	-2.10669556455055
H	1.63647870108242	-1.71335187764321	-0.03322692248195
H	2.75565659761056	1.34779281776544	1.09185054584843
H	1.62474273818967	2.62752701383002	0.59260909691247
H	3.28460559978076	2.59583378821681	-0.06086002512678
H	2.40470597120176	2.68494869212041	-2.30211605058485
H	0.70896662018105	2.59492981209856	-1.79211604826789
N	1.17529947636149	1.22095173062493	-3.28506673482707
H	3.53521664756714	-0.40086154519960	-0.52412145225039
H	4.01499156824311	0.91495625275851	-1.61549237930667
H	2.98271105189544	-0.40144955636108	-2.20911706734642
H	2.00208345038683	0.84719346430682	-3.74952178914873
H	0.74685777242404	1.84467304236480	-3.96761799498599
H	0.47889922463194	-1.23100074655102	2.05323694018953
H	-0.17655167329258	-2.88541569999133	2.09362113366626
H	-1.05863841152742	-1.55448432072301	2.88029592022602
H	-1.34497571316970	2.25602863275229	0.61011364561913

12_enol_ax_S

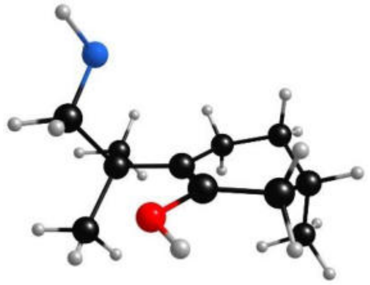

C	-1.59164816784391	-0.63559224754476	-0.43645275144843
C	-0.57008885921085	0.47752517041075	-0.64668505983466
H	-2.58637990050188	-0.20041827683618	-0.25014088692219
C	-1.19312982855484	-1.57177697670440	0.70270224696065
H	-1.67268216599761	-1.22924662111687	-1.36255915621002
H	-0.72058702900069	1.27346209230612	0.10404399733454
C	0.86987755872992	0.01385909781192	-0.62280236014370
C	1.14718743050567	-1.23274899470670	-0.20558126111878
C	1.96202464811141	0.98792079727196	-1.09572803950369

C	-1.12263460302296	-0.85872780404490	2.05218500478571
C	0.13966212644572	-2.21651930325488	0.32654824719685
H	-1.94861752136528	-2.37006775425967	0.78142455003754
H	-0.01733265787960	-3.00826611541276	-0.42747307820872
H	0.58411959071114	-2.71835628650562	1.20511255141673
O	2.42284629870132	-1.73669884057911	-0.26398072871709
C	2.61286839927264	0.46108420320367	-2.39126854005227
C	3.04957939001038	1.14295302087166	-0.01974852607654
C	1.40769193602754	2.39462739290552	-1.37013063205808
H	3.51824764551497	0.18163845509030	0.21867305862928
H	2.61424028157845	1.55203931704225	0.90497907728875
H	3.83192496781145	1.84023105083019	-0.36007168363425
H	2.23194845344126	3.05519389553615	-1.68099148461027
H	0.95216282721617	2.83225456333400	-0.47025508848135
H	0.65159918721386	2.40838431709703	-2.16582025040763
N	1.66009449928824	0.33933112187965	-3.48737933947287
H	3.02876632605197	-0.53386813865889	-2.18440209254795
H	3.47024857500463	1.12371001621268	-2.63331461928918
H	-0.32188287979724	-0.10398476294847	2.06728722190879
H	-0.91948864120519	-1.57436358014702	2.86342762591414
H	-2.07197652766372	-0.34973918333726	2.28045693440241
H	-0.76899069799213	0.95335456709772	-1.61781589259018
H	1.45661609244154	1.25852739803748	-3.87817023249730
H	2.08568664567517	-0.18488892865189	-4.25065846338634
H	2.43983660028242	-2.60355266222969	0.16259965133610

13_imine_1_R

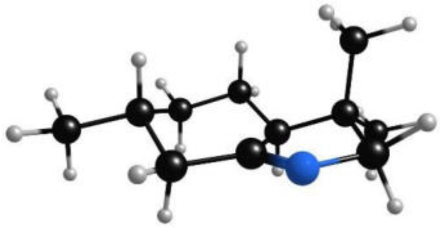

C	-1.47252330042408	-0.42815833348384	-0.04450571185135
C	-0.47029856168755	0.62866778744263	-0.45456035765825
C	-2.48877310718445	0.40307803135856	0.78250580761192
C	-0.81177239628870	-1.65339796436361	0.58719335129036
H	-1.96498551532684	-0.76033459693210	-0.97870314509877
C	0.83641248013058	0.22083247527525	-1.05765059225696
C	1.44124991284836	-1.01335228472138	-0.37450291224645
H	1.53086498494826	1.07370171184222	-1.05319723235977
H	0.64311745443434	-0.03254572865246	-2.11692474580026
H	-0.45476651188089	-1.40852593316530	1.59876964794455
C	0.38570872642208	-2.11528123566172	-0.24138933927475
H	-1.54613968191115	-2.46619641678959	0.69854480688310
H	0.05368059754719	-2.41980924628227	-1.25059165223744
H	0.83995000298250	-3.00565033768063	0.22132297047396
H	1.73920099360808	-0.71864599343239	0.64811017511236
C	2.68316323238067	-1.49396209709613	-1.11404878791903
C	-2.22277179498258	1.81910431800542	0.21068865908254
C	-2.15610494868978	0.39924029990073	2.27736319361349
C	-3.92907854473614	-0.05416777750256	0.57307753699273
N	-0.85497435443154	1.83296241220798	-0.30829414382746
H	-2.36208492451321	2.61171593048642	0.96312404244975

H	-2.91075230349714	2.04930797180937	-0.62255416001903
H	-1.12303875862745	0.73126442532396	2.46281715036779
H	-2.27709616197325	-0.60040743324700	2.71944545767562
H	-2.83085051336101	1.08578874054773	2.81104552356309
H	-4.19849130467422	-0.04359259629865	-0.49409349712035
H	-4.63357779623466	0.60174763018566	1.10849272524757
H	-4.07568171681985	-1.07842233333498	0.95054847050705
H	2.42694394214976	-1.81489543148569	-2.13702988267226
H	3.14759702106156	-2.34871455247017	-0.59936685155540
H	3.43579284873111	-0.69450144178545	-1.19290650691833

14_imine_1_S

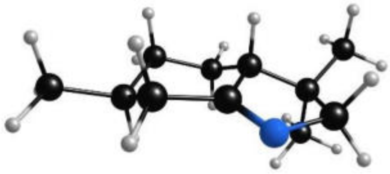

C	-1.50096055896587	-0.32191507092239	0.11511294354159
C	-0.44389750880060	0.76480532532967	0.31147143330047
H	-2.43867779964573	-0.03473455757127	0.61567370525238
C	-1.00097623994024	-1.66920992202155	0.63342303854788
H	-1.73378461845643	-0.43115292072092	-0.95472827570801
H	-0.36663990968840	0.96107014589551	1.39823951033178
C	-0.59639312652884	2.12087465359092	-0.42712711947705
C	0.93428008433098	0.32491300084908	-0.13197431433359
C	1.39214375113951	-1.07314645611886	0.13894686988412
N	1.65156446877870	1.23031666623945	-0.66623385712419
H	-0.80698822509276	-1.60930884304447	1.71981586733595
C	0.28136862340279	-2.10967514860077	-0.07913554834950
H	-1.77997219142822	-2.43561897235424	0.49569169894622
H	0.06095069321425	-2.14259813846601	-1.16158826831483
C	0.73412254316011	-3.49668286868004	0.35845109988312
H	1.69969109516623	-1.11693833393578	1.20059102047314
H	2.28500971267161	-1.29594835012696	-0.46339056314417
C	-1.32982973224611	3.16512963791418	0.40893103816729
C	-1.28884252951763	1.96391964227561	-1.78402016775200
C	0.89308814412814	2.48133524468982	-0.66022847284878
H	-2.37202998431844	2.86038973338124	0.59333689546773
H	-1.35408032309241	4.13809706665028	-0.10676488726345
H	-0.84031046448505	3.30920932979076	1.38418932750894
H	-2.33812828447148	1.65406901147870	-1.67292712991126
H	-0.77863108828160	1.21995647332069	-2.41494304098264
H	-1.27800420282375	2.92447805443773	-2.32127944099369
H	1.28251374739028	3.11743644352698	0.15483118861358
H	1.05157182719366	3.03771114399133	-1.59785337540612
H	1.65056905498653	-3.80294986686769	-0.16894026056419
H	-0.04185529595091	-4.25082856469652	0.15762105638590
H	0.94844833817166	-3.51293355923448	1.43962802853339

15_imine_2_R

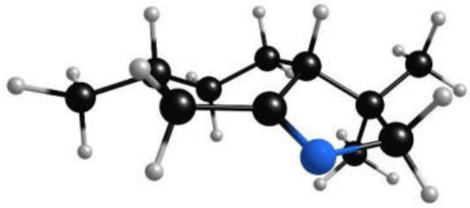

C	-0.91642626893448	-0.30115816553787	0.77081891724383
C	0.21572736394919	0.51853194279041	0.17994392763638
C	-1.25521637242481	-1.67325964077890	0.26357684234268
C	0.48684364913692	1.51152490694943	1.33995433612682
H	-0.24155327262874	1.10117549336614	-0.64111302612521
C	1.38311749754303	-0.30464845653601	-0.39397412769774
C	1.22285439420055	-1.80315114361624	-0.13982515041714
H	2.34058794117095	0.05084971551631	0.01317677634882
H	1.43970231531498	-0.14507069870683	-1.48138331706724
C	-0.14930123390533	-2.29447739593012	-0.60521406293770
H	-0.28414687035166	-1.93647039338817	-1.64156053601909
C	-0.25524506569584	-3.81416697160210	-0.61598309500260
H	1.34067586250204	-2.02576994752526	0.93645579322999
H	2.01889777218221	-2.35608259485076	-0.66283718464413
C	0.99606987835432	2.86467182477522	0.85873584576355
C	1.45059324494363	0.92731118897685	2.37779205138361
C	-0.93137000313220	1.57692384174976	1.95123274382142
H	-1.54174986138043	2.34697375684488	1.44453944768071
H	-0.92572878099598	1.82668587129890	3.02411691360138
N	-1.54076108122702	0.26599288220922	1.72529419230763
H	-1.44827355817138	-2.31001822540337	1.14241560352501
H	-2.20505021453853	-1.63081184479838	-0.29500475806021
H	1.98507024228604	2.76592349376156	0.38398633201362
H	1.10080192640771	3.56829188419236	1.69953416972197
H	0.30911036793135	3.31092214797468	0.12343576684609
H	2.46385724614979	0.80181071729015	1.97021792097025
H	1.10626573151543	-0.05189130564429	2.74352599815334
H	1.52319307301730	1.60327340301312	3.24325435454992
H	0.47983921820094	-4.26155362351430	-1.30210797631820
H	-1.25794043537295	-4.14193931900247	-0.93159778278060
H	-0.07133470604703	-4.22352334387392	0.39086308380282

16_imine_2_S

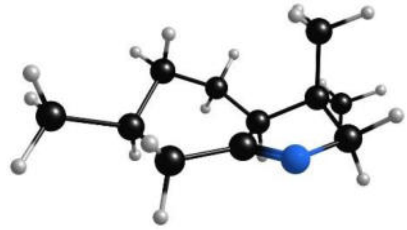

C	-1.40391201157092	-0.63673621276800	0.62797330767235
C	-0.40027435265610	0.51649011634802	0.44744455357843
H	-1.93457788826649	-0.53806105323786	1.58584570082428
C	-0.74120856893492	-2.01029795461148	0.52827406490945
H	-2.17628717052778	-0.57107252196261	-0.15315406501151
C	-0.11821654292665	1.46962577128030	1.63783819461965

H	-0.73703252078217	1.14745666469410	-0.39588608004517
C	1.01099914214517	0.08807872599295	0.09028410785661
C	1.28976701196982	-1.16532944396692	-0.68826083556314
N	1.92524613193849	0.87050292630287	0.50803070138273
H	-0.08783702786553	-2.18695677695529	1.40220043340347
C	0.09099410285184	-2.12577874989471	-0.75034625750677
H	-1.51164653192251	-2.79702634879835	0.54932675510506
H	-0.55911207625472	-1.81095372481407	-1.58617632661497
C	0.55039427723296	-3.55222452166794	-1.02419484317706
H	2.14954414551733	-1.66404134001434	-0.21161559848033
H	1.62056181262097	-0.89637637605435	-1.70522523391090
H	1.16693957327664	-3.60486317252122	-1.93500394126168
H	-0.30662295064937	-4.23029222758065	-1.15468076596898
H	1.15825280524742	-3.93216139604314	-0.18675375713568
C	1.28626997422017	1.97066066440238	1.23083731673438
C	-0.01150040966096	0.71030958479388	2.96422190438328
C	-1.14349584345838	2.59029725861455	1.75929793057787
H	-1.22397387057145	3.16077452942157	0.82131859334539
H	-0.86815031073678	3.29122790328929	2.56296321390647
H	-2.14001268910624	2.18660945503947	1.99853388342313
H	0.72319630821197	-0.10687592621236	2.90464764817880
H	-0.97630162122499	0.27971340599493	3.26803073127845
H	0.31114257001393	1.39554768734066	3.76270627015600
H	1.89818724926610	2.28114045703227	2.09260932064921
H	1.21102728260316	2.84450259655604	0.55774307269119

17_imine_3_R

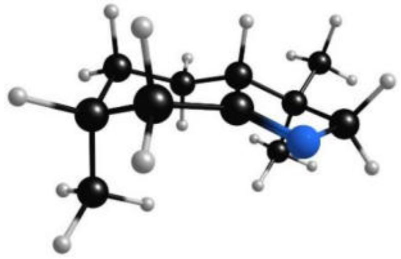

C	-1.49965421654917	-0.37425933234612	0.13121682003364
C	-0.42750983971712	0.70169696768477	0.31486150526123
H	-2.40933079574704	-0.09928954974508	0.68714735177153
C	-0.98870295246590	-1.74017511195681	0.59148327486026
H	-1.79225356304363	-0.44119077108865	-0.92560445186584
H	-0.33210349041732	0.89169565599935	1.40142167802378
C	-0.58251647143500	2.06399950330371	-0.41203926640905
C	0.94657993763608	0.26140274059902	-0.14480863517069
C	1.40516390624944	-1.13900647910916	0.11872450266575
N	1.66187689483558	1.17090794004802	-0.67481995254195
H	-0.78440529475367	-1.71004640772354	1.67561392692414
C	0.29253220448730	-2.16701487671181	-0.14122301321442
H	-1.77082604872175	-2.50099349963696	0.44068706615797
C	0.07279050305058	-2.37840568182813	-1.63856335529571
H	0.61862326535775	-3.12770084678344	0.28813236978368
H	1.69011590436871	-1.18856216010810	1.18462413200116
H	2.30964613769440	-1.35162205131378	-0.46961663005450
H	-0.16320456816478	-1.43654403986517	-2.15779430281718
H	-0.75664085855684	-3.07814514850697	-1.82405165424496

H	0.97760928980863	-2.79094484690039	-2.10976785650088
C	-1.30855566875399	3.10354136550057	0.43626531400484
C	-1.28375560964440	1.91916315140996	-1.76592684707199
C	0.90588396567205	2.42324886101383	-0.65188320794529
H	-2.35001547033166	2.79929650000632	0.62554587050724
H	-1.33473341651524	4.08013319208681	-0.07245187995581
H	-0.81220269040940	3.24001714671699	1.40914960421099
H	-2.33550789190414	1.61943551159959	-1.65124337156914
H	-0.78402504797375	1.17351517187837	-2.40339947390337
H	-1.26640469197941	2.88166852974331	-2.29942292772259
H	1.30193582354085	3.05048159434695	0.16688444302428
H	1.05944075438286	2.98844697168653	-1.58502103294710

18_imine_3_S

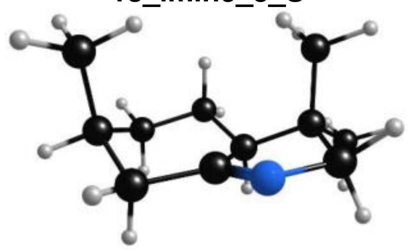

C	-1.43391803471647	-0.44162304844776	-0.08343607611900
C	-0.42057612449027	0.61923290507452	-0.47275592314495
C	-2.44194690277331	0.38401146097308	0.75700173608250
C	-0.79851948827264	-1.69330872600993	0.52274495706869
H	-1.92732151306501	-0.74501957158511	-1.02718584122250
C	0.88265195785889	0.20501745503020	-1.07912560114980
C	1.48859589333446	-1.01762429059056	-0.37274118676825
H	1.57652191766436	1.05738989128446	-1.10368415258038
H	0.66575154474007	-0.07178379483498	-2.12626652382012
H	-0.49720285216780	-1.49470019692615	1.56020007877727
C	0.43318772104485	-2.12836319127496	-0.27182935336615
H	-1.53570654147629	-2.50954423950060	0.56477198880827
H	0.13165393695386	-2.42306556242022	-1.29140302009479
H	0.87973112013570	-3.02060995225824	0.19431971363815
C	2.08505475596257	-0.64101416508075	0.98279460202153
H	2.31078938740556	-1.39349358333471	-1.00193980932064
H	2.89915640112640	0.08844886997661	0.85765352483993
H	2.49675313205998	-1.52519742839040	1.49273252024910
H	1.33992854056493	-0.18485227404478	1.65218820658203
C	-2.18441990085803	1.79607052787539	0.17753900314304
C	-2.08266620211965	0.38387940698878	2.24596422195671
C	-3.88446592455865	-0.07310427604780	0.57460916526950
N	-0.80324137878532	1.82264087435855	-0.31489770641784
H	-2.36485748989853	2.59921798596763	0.90876884657135
H	-2.85110105877285	1.99067910206526	-0.68193954595261
H	-1.04365124656297	0.70460259308226	2.41457448177760
H	-2.21137015546291	-0.61031121036791	2.69717387360051
H	-2.74127766130719	1.08216566645882	2.78409721285411
H	-4.17418953616591	-0.06384311177299	-0.48706733342291
H	-4.57810438719275	0.58302660017069	1.12297333293712
H	-4.02172991020508	-1.09701471641838	0.95533460720251

19_imine_4_R

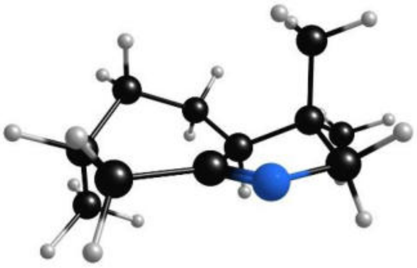

C	-1.35740925642131	-0.69230170541902	0.70017370502769
C	-0.36356073949540	0.46555078302165	0.49559395014272
H	-1.80172263765021	-0.64536045285124	1.70429561146492
C	-0.71480921104911	-2.06638534775301	0.49237564786008
H	-2.19687239656214	-0.57723670076568	-0.00020564907519
C	-0.10057011506273	1.45869120628587	1.65693844383391
H	-0.70612797231709	1.06909809622200	-0.36570625923593
C	1.05707327537125	0.05831709547353	0.14548525671483
C	1.36965982817759	-1.21105440145085	-0.59840131285059
N	1.95190039360669	0.87853362715488	0.53135903777889
H	-0.07573990993701	-2.31128541829790	1.35844152657458
C	0.14528829723734	-2.12350361102055	-0.77617084590929
H	-1.49895738975856	-2.83879585143592	0.45430707398738
C	-0.64612103220140	-1.78133459858492	-2.03853474139797
H	0.51440735140328	-3.15501268158893	-0.88841548785781
H	2.15481737950090	-1.73448166123512	-0.03068981398956
H	1.81675070767382	-0.95980067173630	-1.57427711788530
C	1.28941766651018	1.98126595667225	1.22842383373906
C	0.02978635754090	0.74058881474919	3.00392535623060
C	-1.15191132441996	2.55778047965807	1.75067106975843
H	-1.24917670739413	3.09908098321387	0.79711464647209
H	-0.89064504404874	3.28791851217015	2.53276452373028
H	-2.13764202108279	2.13748755103993	2.00536639459811
H	0.77240566963134	-0.07061684203192	2.95978533473263
H	-0.92653709155985	0.30940846391582	3.33256633032600
H	0.35427906418531	1.45273362500735	3.77773845184897
H	1.89799464261839	2.32991033705713	2.07787203792222
H	1.18970456139726	2.83446260466085	0.53232053630107
H	-0.03371879042321	-1.95022789528480	-2.93708469314832
H	-0.96393629227401	-0.72731670694061	-2.05409672583968
H	-1.55047726319661	-2.40393358990579	-2.12201612185484

20_imine_4_S

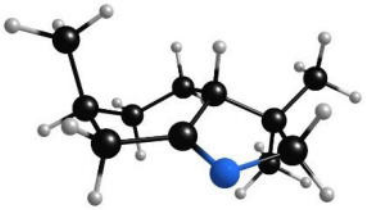

C	-0.93087810849347	-0.39151360572880	0.79365281249833
C	0.20930214406627	0.40826418312926	0.18786455752878
C	-1.27640639222893	-1.77918006770979	0.32750237168825
C	0.47490934946818	1.43393778787140	1.31997313110115

H	-0.25022417406392	0.96973037457531	-0.64705032234280
C	1.37973946868059	-0.42006101974003	-0.37278355913904
C	1.16127636235388	-1.92800450991711	-0.22284338874336
H	2.32153731052705	-0.13704155179800	0.11803454362193
H	1.51684351740468	-0.17973416240063	-1.43671568028852
C	-0.24567033925341	-2.36033560934277	-0.65396934352810
C	-0.55794583579389	-1.98374960417407	-2.10229923705359
H	-0.29978089071395	-3.45780532489297	-0.57999942607636
H	1.30853938136801	-2.22062470232524	0.83128064140669
H	1.92081950575124	-2.47111860678938	-0.80686102551692
H	0.21990601260125	-2.35645458513291	-2.78676284383932
H	-0.62910596628987	-0.89367584886470	-2.23965332920654
H	-1.52064612599595	-2.41509181833026	-2.41550768893399
C	0.99435036843001	2.77019029548844	0.80351290516429
C	1.42614211649846	0.87647432830893	2.38360669748733
C	-0.94873669695028	1.52338052541850	1.91489843706095
H	-1.54987645573562	2.28177962923733	1.38021013515113
H	-0.95247065129417	1.80399016762213	2.98013247774680
N	-1.56436490336368	0.21041343897055	1.72031368349530
H	-1.34811611780858	-2.41411671160320	1.22442191819221
H	-2.28367742343976	-1.77155620503976	-0.12054212204373
H	1.98679340147787	2.65305095472539	0.34036235719776
H	1.09506528541899	3.49801928921195	1.62397198349369
H	0.31561697281861	3.19746376098550	0.04951688243661
H	2.44621990427814	0.74906918669615	1.99425453939881
H	1.08190942442898	-0.09753711907712	2.76352703766491
H	1.48193955585327	1.57006713062587	3.23628085437737

21_enamine_eq_R

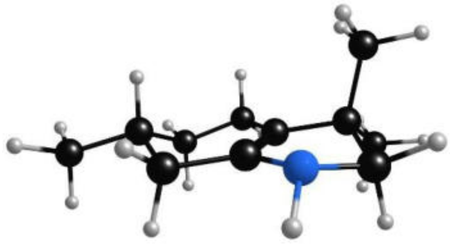

C	-1.23448763437891	-0.28006335463413	0.33609203608971
C	-0.42211911957055	0.60393917719183	-0.26197245033779
C	-2.42509916880351	0.46220470226364	0.92971056159116
C	-0.88878727234271	-1.72510123758033	0.51433938281957
C	0.90777116884312	0.28241977431271	-0.86199077236860
C	1.40820683284331	-1.08645264459852	-0.38210754666473
H	1.63370495870441	1.07011826875635	-0.60008345523298
H	0.83405017528434	0.29226466384339	-1.96555347265897
H	-0.61529111112139	-1.93356575330892	1.56648245939119
C	0.27069498296507	-2.11289832833594	-0.40767153761689
H	-1.76270056168338	-2.36535761798579	0.30561167263881
H	-0.09982872937238	-2.19785702983579	-1.44489371505250
H	0.66062425745490	-3.10523859574064	-0.13056477263052
H	1.72042561950036	-0.96975343080474	0.67169783539553
C	2.61384988827595	-1.55126775183088	-1.18906413355668
C	-2.33335877766913	1.79231195304894	0.14235217344259
C	-2.19342189437084	0.70611139162422	2.42919695525672

C	-3.76656236851166	-0.23540111815966	0.71560616192275
N	-0.91385531882811	1.91802194176635	-0.19862729653036
H	-2.69400054276082	2.65299687335788	0.72650365006539
H	-2.95034808462737	1.71710271857066	-0.77321272385363
H	-1.26903913559100	1.28142923672063	2.58841128190026
H	-2.10099137175676	-0.24636660868038	2.97343982644192
H	-3.03074494912078	1.26929158582484	2.87228482651275
H	-3.92696610362110	-0.46854989628186	-0.34804420159599
H	-4.59911459776476	0.40086442629493	1.05638540428535
H	-3.81692935355493	-1.17810119859876	1.28224371316326
H	-0.72226082548943	2.48803357120503	-1.01869369420664
H	2.33726181949512	-1.71625382555070	-2.24338867590089
H	3.01783164554028	-2.49672169634828	-0.79607203392335
H	3.42144557203270	-0.80308019650607	-1.16900745878642

22_enamine_eq_S

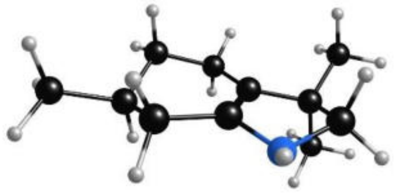

C	-1.53895902980030	-0.30287437203822	0.21856016073160
C	-0.44618269938925	0.64784918812324	-0.15747509577978
H	-2.22134960532839	0.15604464882772	0.95404995826294
C	-0.94774954194541	-1.59309488399126	0.79358214242862
H	-2.16897733251829	-0.53983250549100	-0.66018675583957
C	-0.59864501975788	2.09669869725571	-0.60297862468118
C	0.83485897974458	0.27805536141222	-0.30334289742213
C	1.36340042656316	-1.10170731005118	-0.08197047415238
N	1.64727037207993	1.32070256022478	-0.77812411158043
H	-0.58541472676529	-1.40339099054589	1.81976793770616
C	0.22108268583009	-2.12545100618921	-0.04223589461803
H	-1.72859596712541	-2.36603842961205	0.87345603345850
H	-0.14544807494610	-2.24725062408259	-1.07781798871314
C	0.70691981400766	-3.48093200683031	0.45517546755931
H	1.93586611308480	-1.13731145330485	0.86375375033199
H	2.08060059003031	-1.35717393466267	-0.87971769095617
C	-1.48202353423803	2.94214415621492	0.31184125104777
C	-1.13399578538511	2.14657516952547	-2.04259640474595
C	0.88124179540352	2.55014333212442	-0.55741319371210
H	-2.53049271104089	2.60894275623922	0.26742916339282
H	-1.45815198023789	4.00196620885850	0.01133457351185
H	-1.14766826621451	2.87536341465847	1.35834954889980
H	-2.12950059979787	1.68123457887949	-2.10878360904155
H	-0.45912586052124	1.60742178959374	-2.72427854263706
H	-1.22237331420784	3.18673831657074	-2.39591703768528
H	1.10181130648878	2.97456977199569	0.44047195028396
H	1.10952816719595	3.31824651412734	-1.31239646860991
H	2.59172993735581	1.33447048210067	-0.40136311820662
H	1.55085024039946	-3.84975204234756	-0.14828532500327
H	-0.09500060295455	-4.23366463855507	0.41228781234362
H	1.04855422399020	-3.41287274903049	1.50111348342563

23_enamine_ax_R

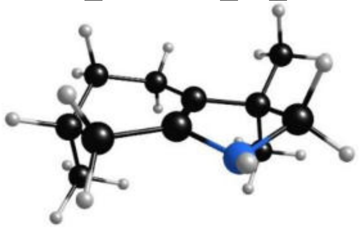

C	-1.49565138715277	-0.36550404921310	0.22541202478484
C	-0.40957496180489	0.59967179816093	-0.12989363382934
H	-2.17749272090920	0.06868946063723	0.97625353898766
C	-0.89451490305813	-1.66646396129597	0.76943480949038
H	-2.12989887396541	-0.57674805234734	-0.65637739271480
C	-0.57778519714919	2.04516087858298	-0.58015451459388
C	0.87612317793478	0.24408358586709	-0.27449837428018
C	1.42246969640369	-1.13093455292241	-0.05711244441099
N	1.67663152234224	1.29749263414615	-0.74593332724898
H	-0.54345544477428	-1.49287873105144	1.80051798925007
C	0.29001615564862	-2.17123826714348	-0.06866522996632
H	-1.66914094793737	-2.44764562587155	0.82650977078795
C	-0.12791171528970	-2.52108900847709	-1.49643685239839
H	0.66870309492843	-3.09095073164543	0.40591148563967
H	1.96732057725939	-1.16605716146229	0.90273596772017
H	2.16595428395004	-1.36002722600957	-0.83847158517655
H	-0.44040613569789	-1.62448911132784	-2.05359261089288
H	-0.96946039921602	-3.23125432201832	-1.50078661947817
H	0.70576463089003	-2.98091912113812	-2.04862847551076
C	-1.47983571453285	2.88190607004803	0.32406991172351
C	-1.10174303825084	2.08084226687264	-2.02448087432360
C	0.89536543620391	2.51724333464239	-0.52562188710601
H	-2.52248002175098	2.53142830013870	0.27509684753848
H	-1.47124327090194	3.94030689094037	0.01772679017205
H	-1.15110303172253	2.82627811163803	1.37301895415678
H	-2.08982774449011	1.60085523997516	-2.09653372345352
H	-0.41351500157318	1.54737489404855	-2.69722530183373
H	-1.20187176624052	3.11781229519465	-2.38409952888989
H	1.10527974387185	2.94138365326755	0.47475831815762
H	1.11846475161260	3.29001968014405	-1.27738665906384
H	2.62023920537225	1.32234082761943	-0.36756737323733

24_enamine_ax_S

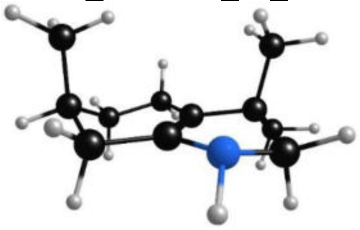

C	-1.18814971330184	-0.28584623609506	0.28871452184657
C	-0.38686068790778	0.60766930356903	-0.31119984376993
C	-2.37953675012491	0.44487101084459	0.89456899251636

C	-0.82141194791258	-1.72347171623682	0.47860570556288
C	0.94614827369362	0.30361230727777	-0.91698086989992
C	1.48055378360492	-1.04289328237061	-0.40108160506047
H	1.65273417148721	1.11633209302354	-0.68032781506087
H	0.85810106658888	0.28629683944055	-2.01760204485911
H	-0.57176194398064	-1.92288992576247	1.53802197847290
C	0.36105254020100	-2.09482002453743	-0.42347280846991
H	-1.67839533596120	-2.38082323537409	0.25349614602866
H	-0.00038017547321	-2.19747326273355	-1.46045154173372
H	0.76734121610220	-3.07632158491081	-0.13167838121447
C	2.10143650361331	-0.89841865488754	0.98787987897057
H	2.27182663555319	-1.38070238043954	-1.08974873247003
H	2.96931781495275	-0.22203475347005	0.96230628142671
H	2.44226238407697	-1.87199660698167	1.37329916249395
H	1.37942240262825	-0.48451323352329	1.70854034444478
C	-2.30887652976820	1.77482646157328	0.10584313431625
C	-2.13020368677466	0.69216641786513	2.39076418857487
C	-3.71648049970746	-0.26597116728567	0.69774738955267
N	-0.89283261556584	1.91617756180325	-0.24280446411743
H	-2.67604438752758	2.63200373940173	0.69109491221713
H	-2.93021542105931	1.69101633562685	-0.80610959906643
H	-1.20896027831070	1.27603922025010	2.53650384960525
H	-2.02087249206369	-0.25906167608083	2.93400095612382
H	-2.96664138428030	1.24763067518112	2.84518806968534
H	-3.88910448982211	-0.49881905538645	-0.36406038447383
H	-4.55108361807607	0.36049238749647	1.05148364681982
H	-3.74859010882626	-1.21035066725463	1.26296722752647
H	-0.71174472605794	2.49008310997713	-1.06255829598889

8. Literature

[1] G. Casiraghi, G. Casnati, G. Puglia, G. Sartori, G. Terenghi, *J. Chem. Soc., Perkin Trans. 1* **1980**, 1862.

[2] T. Neveselý, C. G. Daniliuc, R. Gilmour, *Org. Lett.* **2019**, *21*, 9724.

[3] M. Peters, M. Trobe, H. Tan, R. Kleineweischede, R. Breinbauer, *Chem. Eur. J.* **2013**, *19*, 2442.

[4] L. Pitzer, F. Schäfers, F. Glorius, *Angew. Chem. Int. Ed.* **2019**, *58*, 8572.

[5] D. Clarisse, B. Fenet, F. Fache, *Org. Biomol. Chem.* **2012**, *10*, 6587.

[6] a) F. Neese, *WIREs Comput. Mol. Sci.* **2012**, *2*, 73; b) F. Neese, *WIREs Comput. Mol. Sci.* **2018**, *8*.

[7] T. Yanai, D. P. Tew, N. C. Handy, *Chem. Phys. Lett.* **2004**, *393*, 51.

[8] a) S. Grimme, J. Antony, S. Ehrlich, H. Krieg, *J. Chem. Phys.* **2010**, *132*, 154104; b) S. Grimme, S. Ehrlich, L. Goerigk, *J. Comput. Chem.* **2011**, *32*, 1456.

[9] F. Weigend, R. Ahlrichs, *Phys. Chem. Chem. Phys.* **2005**, *7*, 3297.

[10] F. Weigend, *Phys. Chem. Chem. Phys.* **2006**, *8*, 1057.

[11] a) M. Cossi, N. Rega, G. Scalmani, V. Barone, *J. Comput. Chem.* **2003**, *24*, 669; b) V. Barone, M. Cossi, *J. Phys. Chem. A* **1998**, *102*, 1995.

[12] Chemcraft - graphical software for visualization of quantum chemistry computations. https://www.chemcraftprog.com.

9. NMR Spectra

1H NMR(400 MHz, Chloroform-*d*)

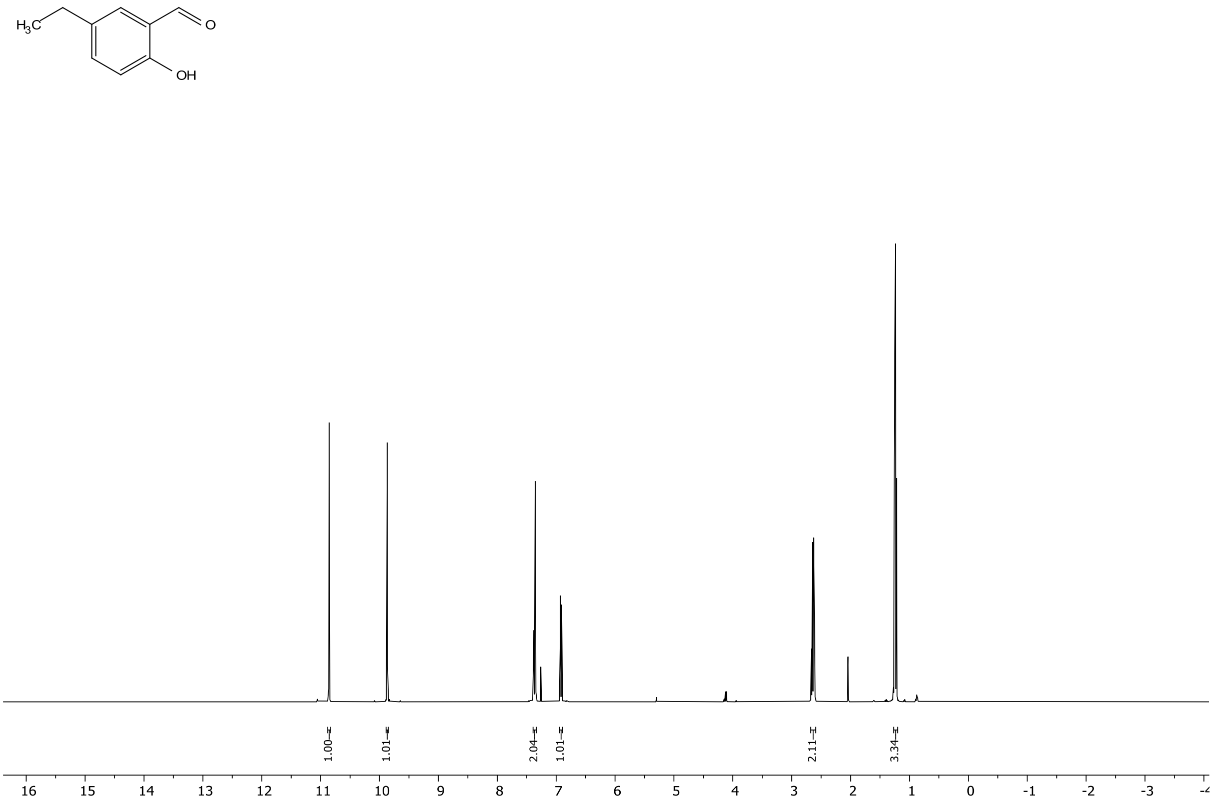

13C{1H} NMR(101 MHz, Chloroform-*d*)

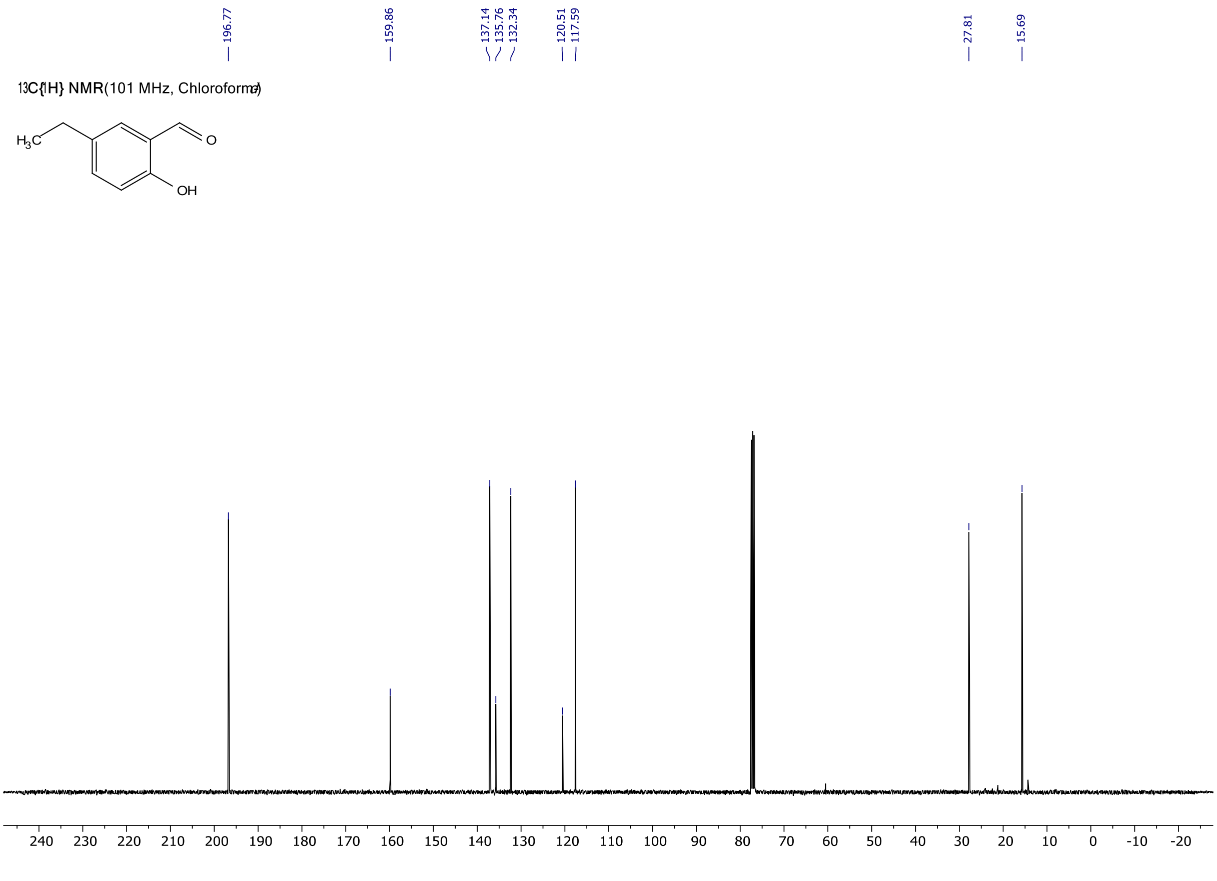

1H NMR(400 MHz, Chloroform-d)

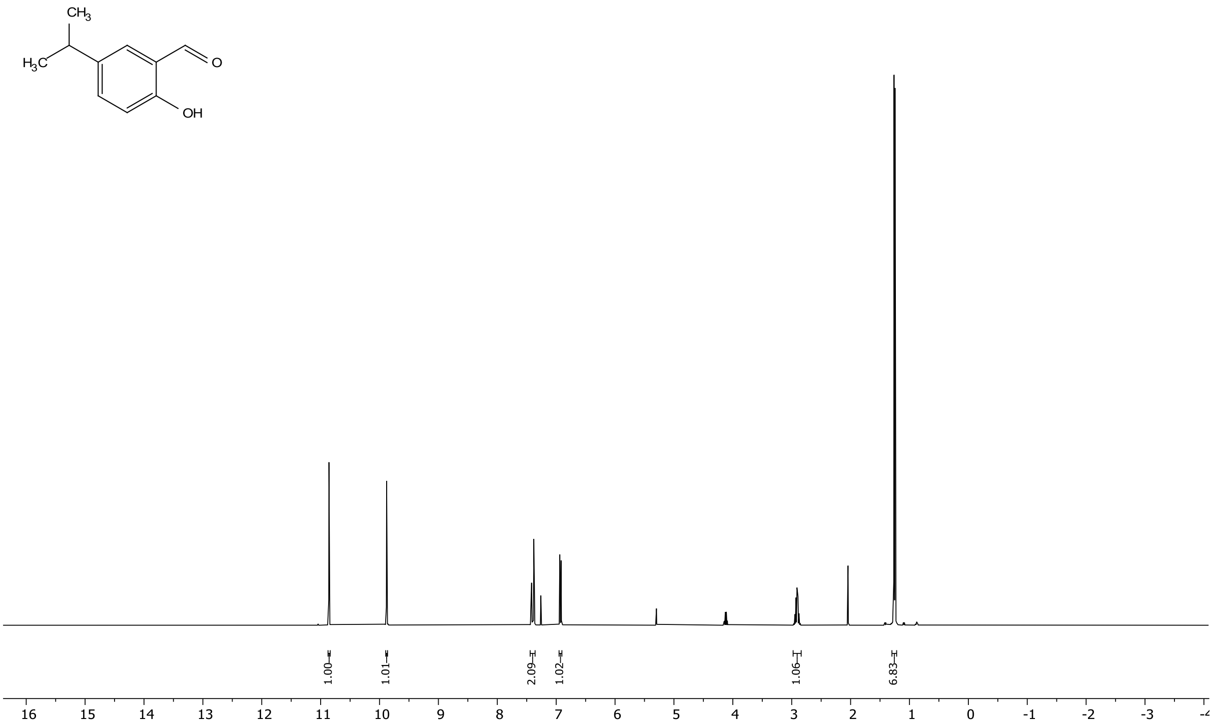

13C{1H} NMR(101 MHz, Chloroform-d)

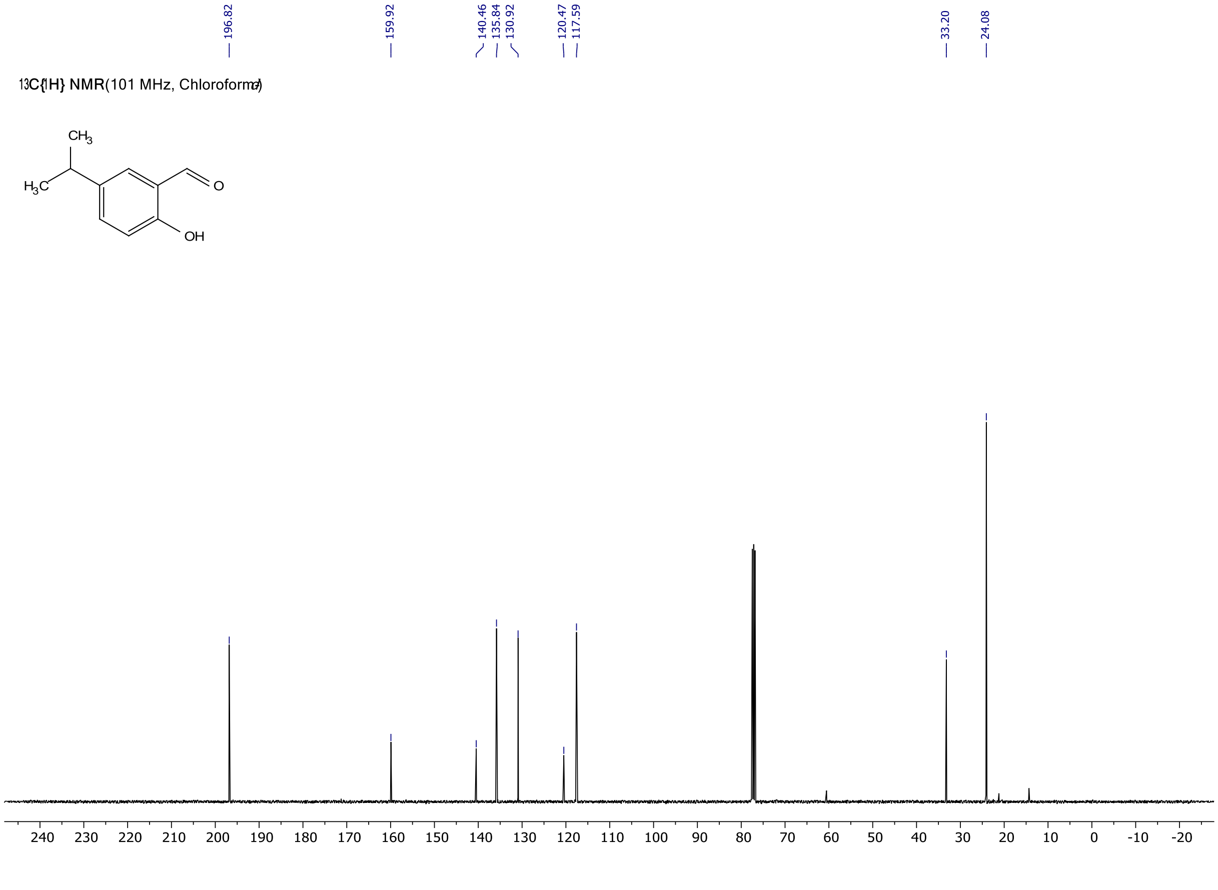

1H NMR(400 MHz, Chloroform-d)

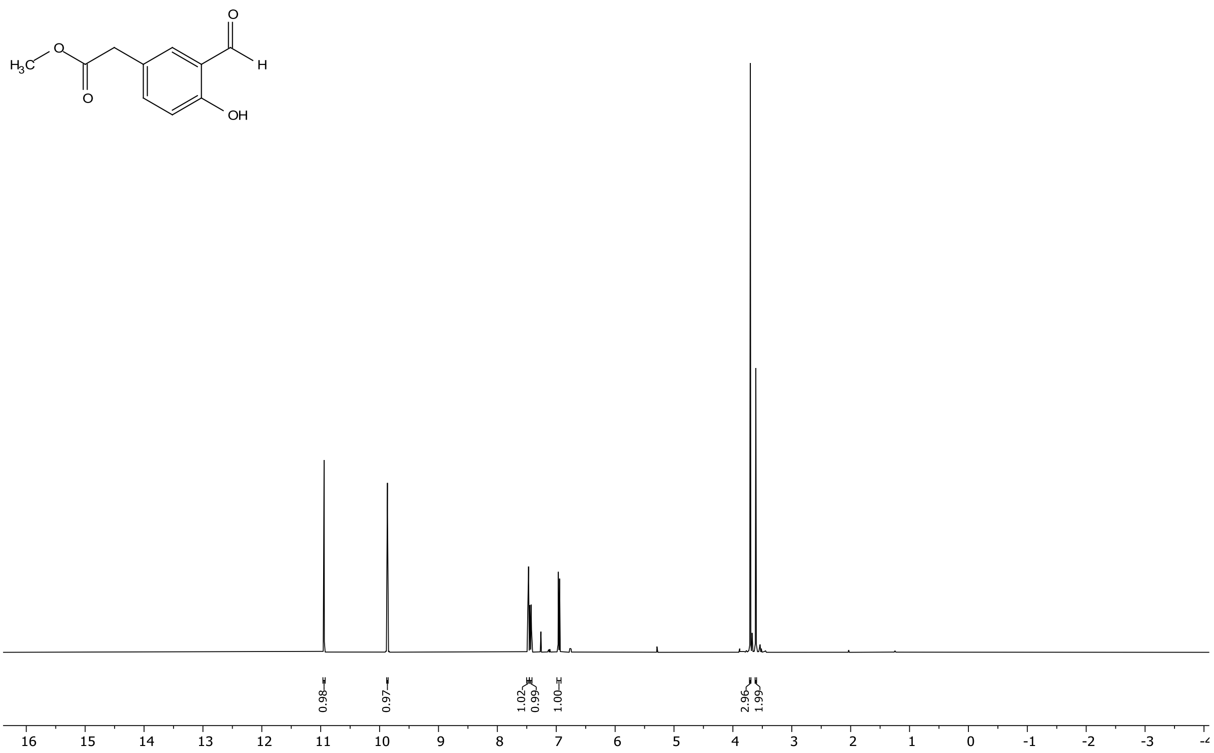

13C{1H} NMR(101 MHz, Chloroform-d)

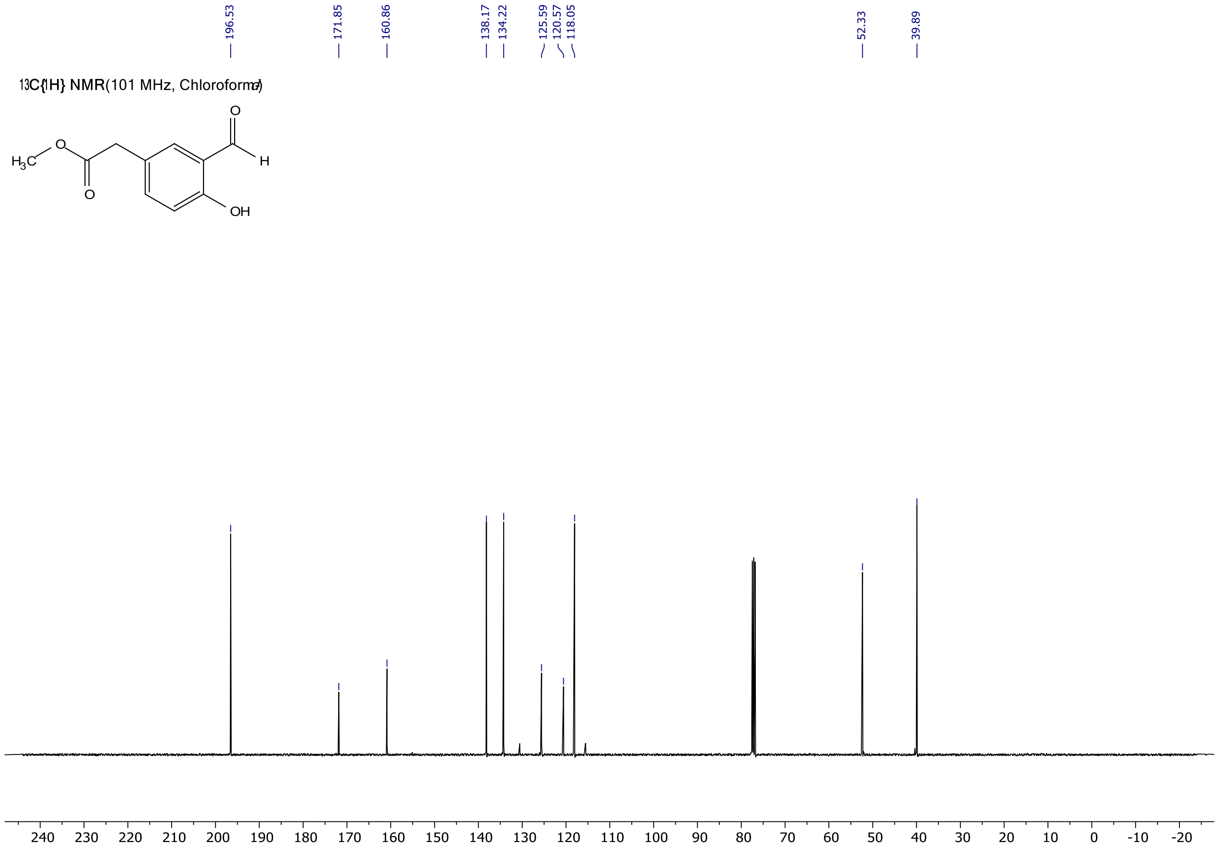

1H NMR(400 MHz, Chloroform-d)

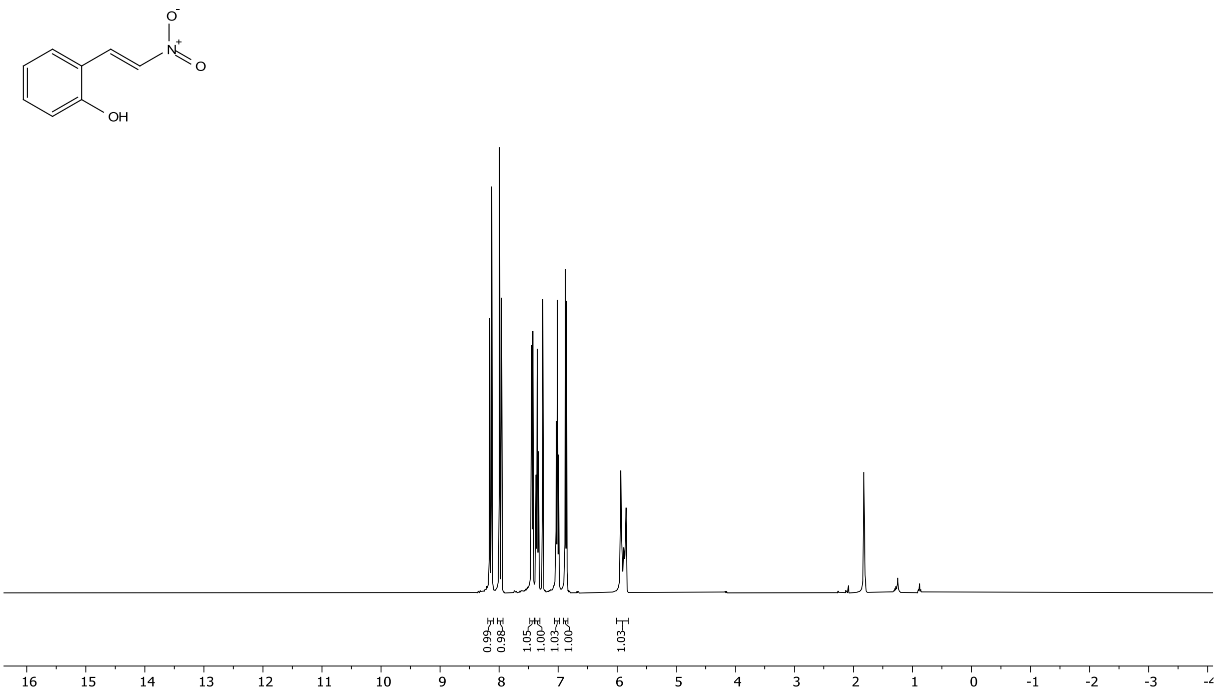

156.13
138.70
135.72
133.40
132.82
121.73
117.81
116.60

13C{1H} NMR(101 MHz, Chloroform-d)

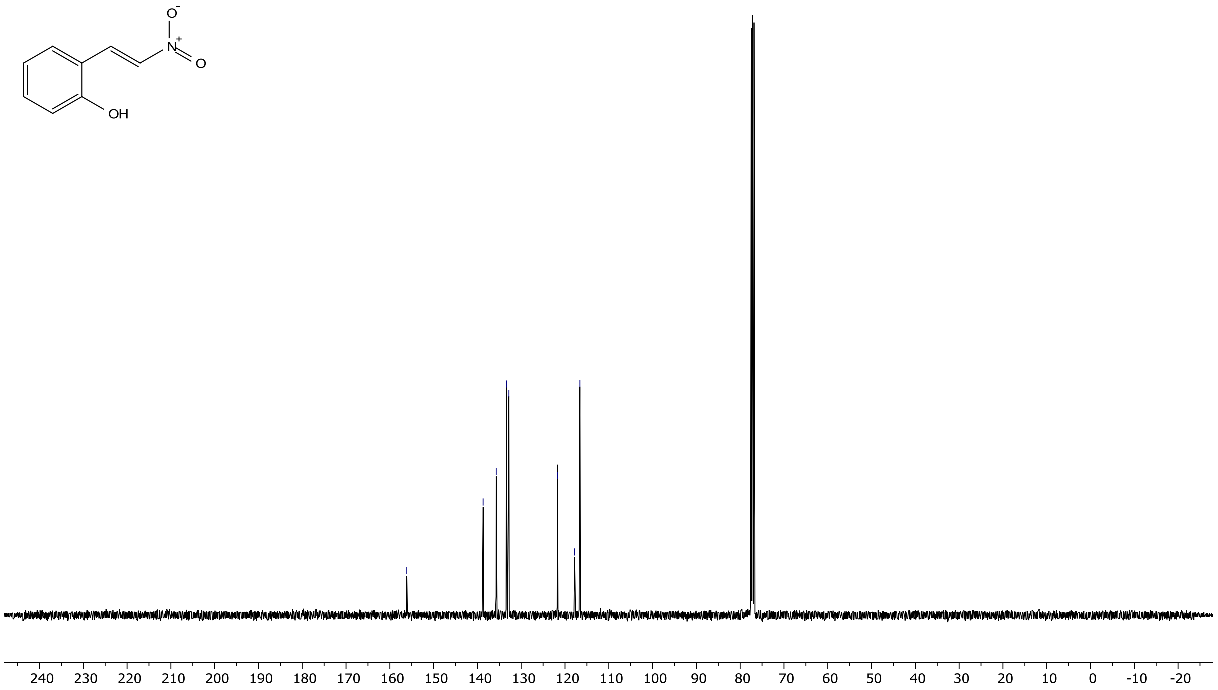

1H NMR(400 MHz, Chloroform-d)

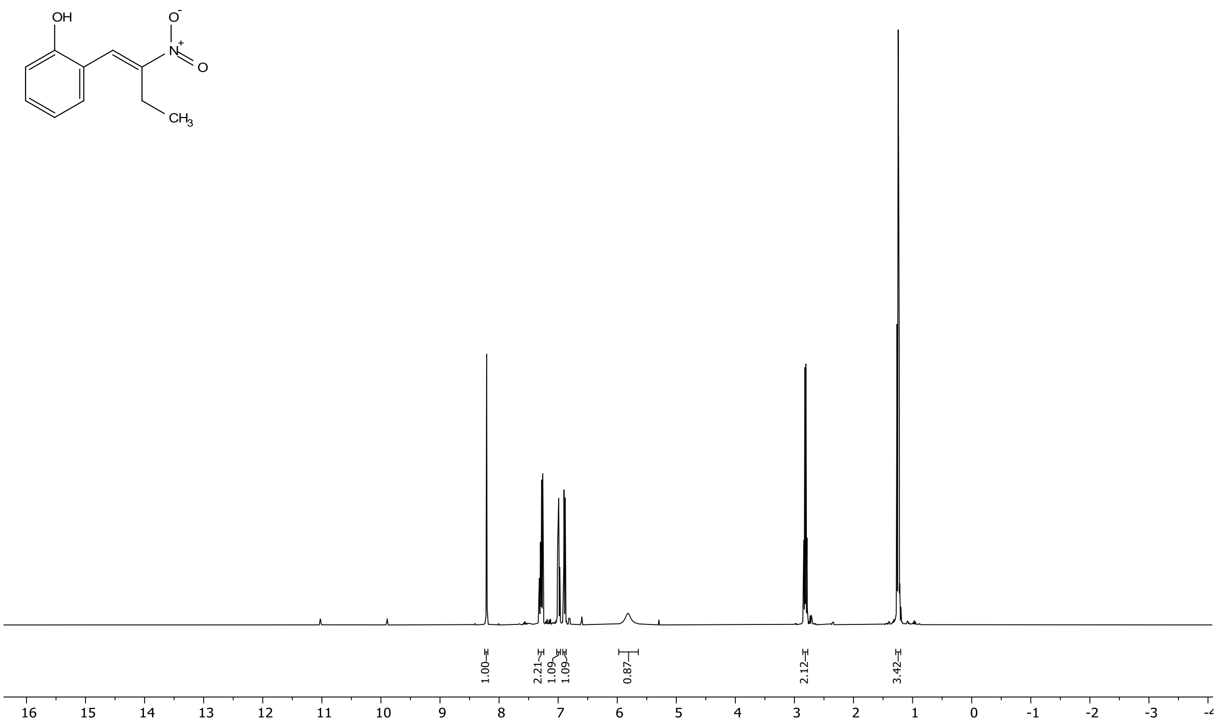

13C{1H} NMR(101 MHz, Chloroform-d)

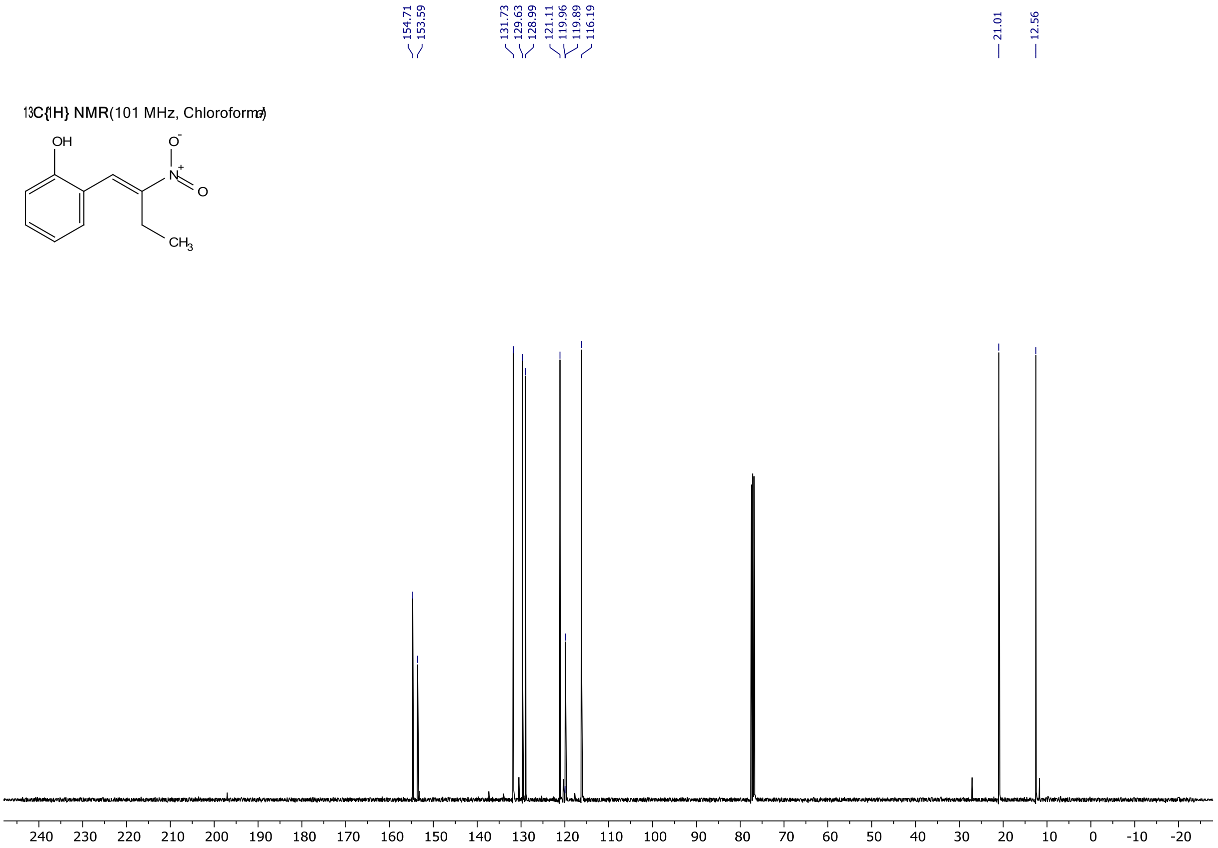

1H NMR(400 MHz, Chloroform-d)

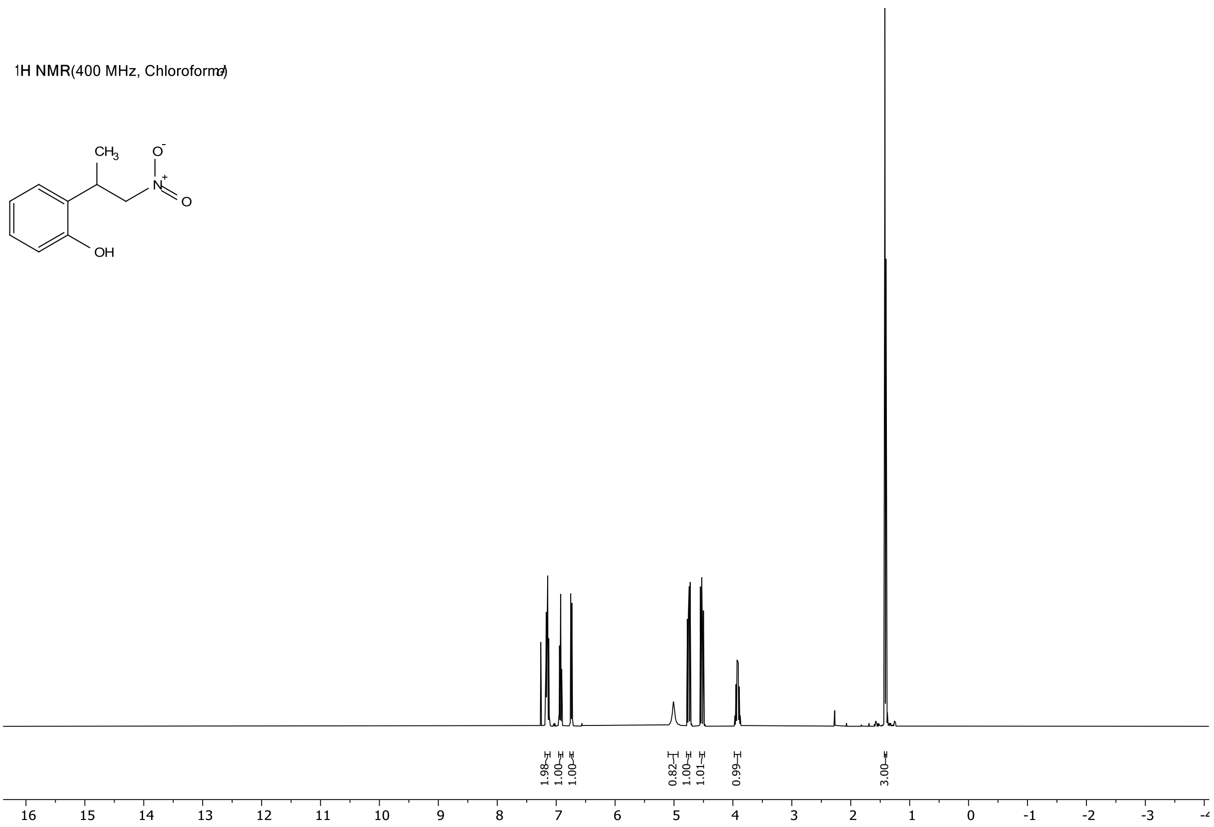

13C{1H} NMR(101 MHz, Chloroform-d)

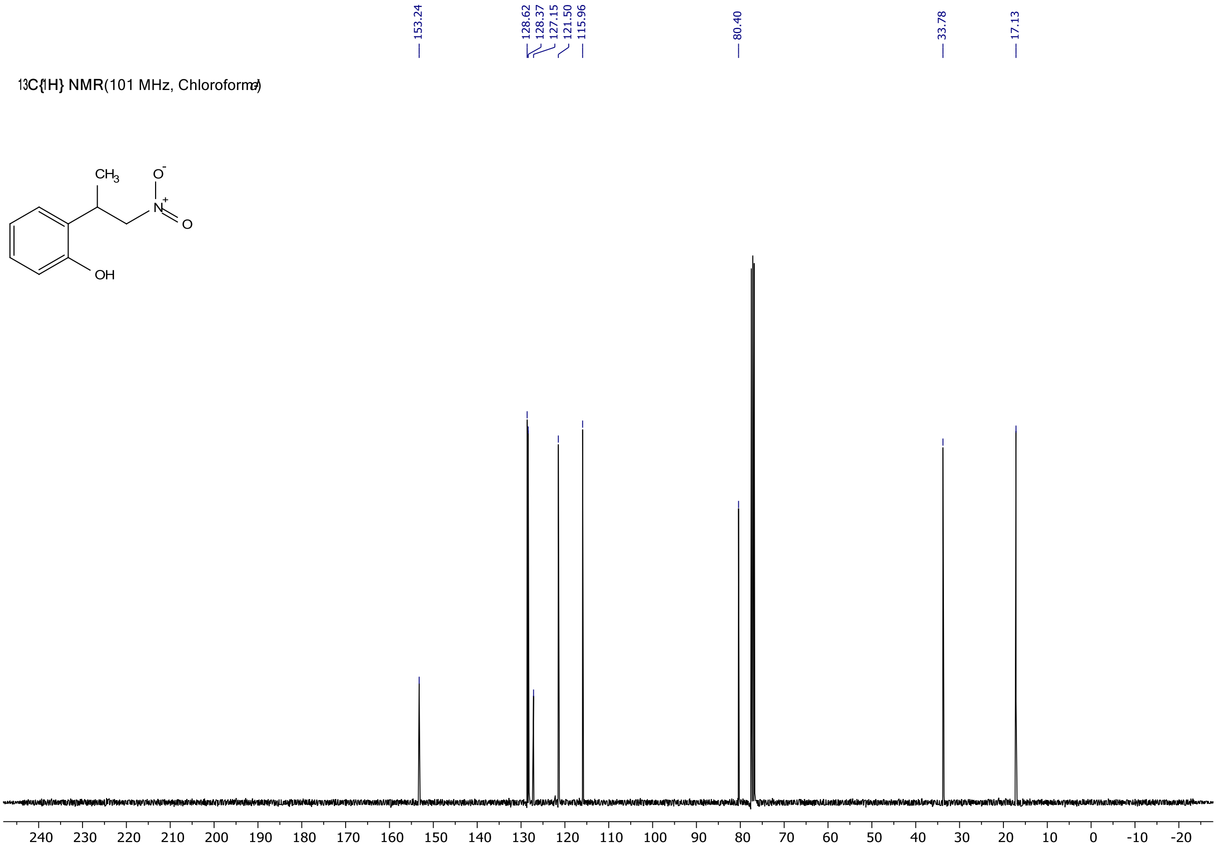

1H NMR(400 MHz, Chloroform-d)

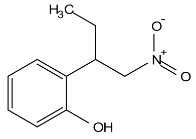

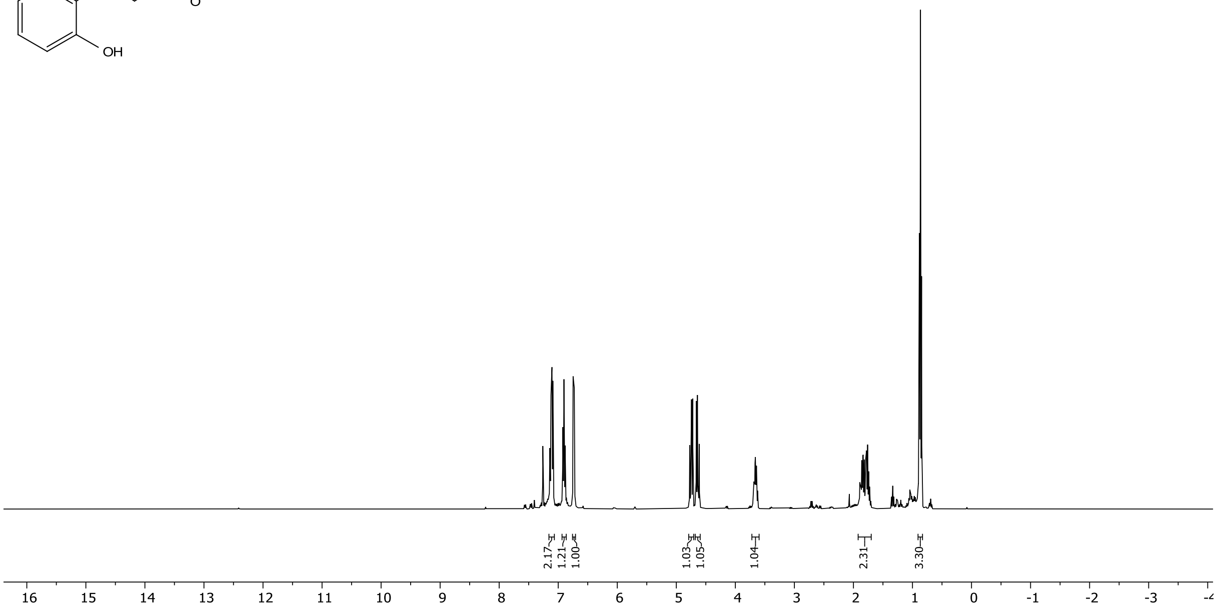

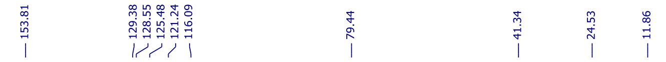

13C{1H} NMR(101 MHz, Chloroform-d)

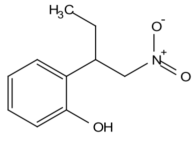

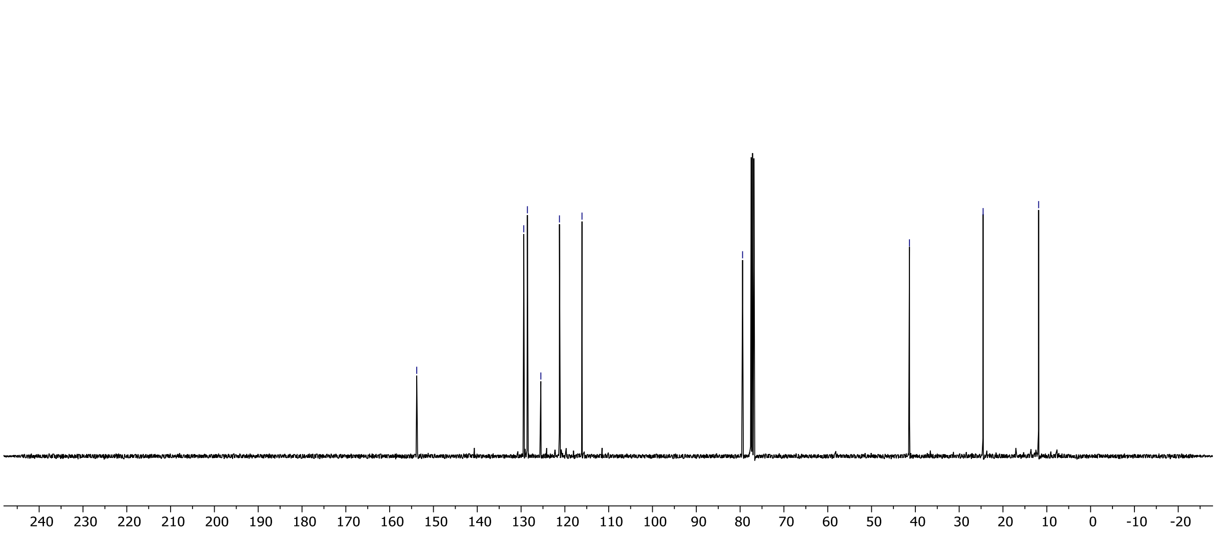

^{1}H NMR(400 MHz, Chloroform-*d*)

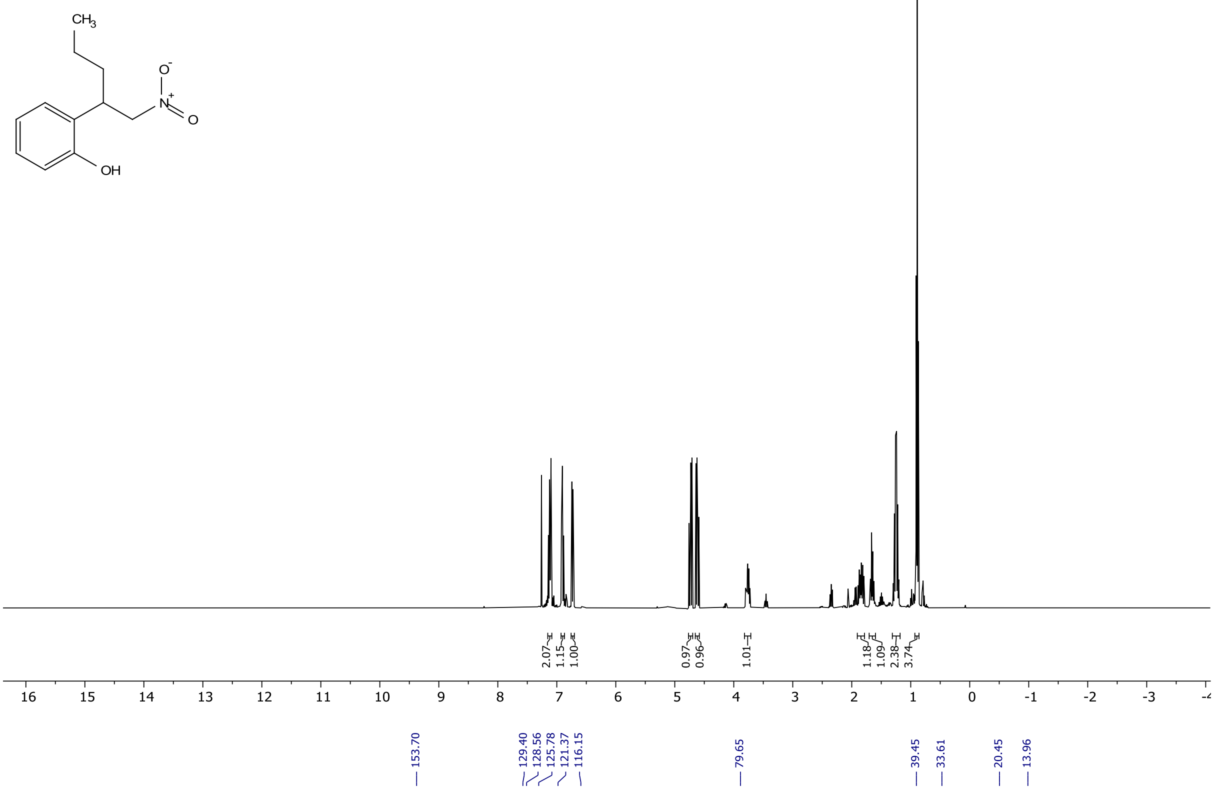

^{13}C{^{1}H} NMR(101 MHz, Chloroform-*d*)

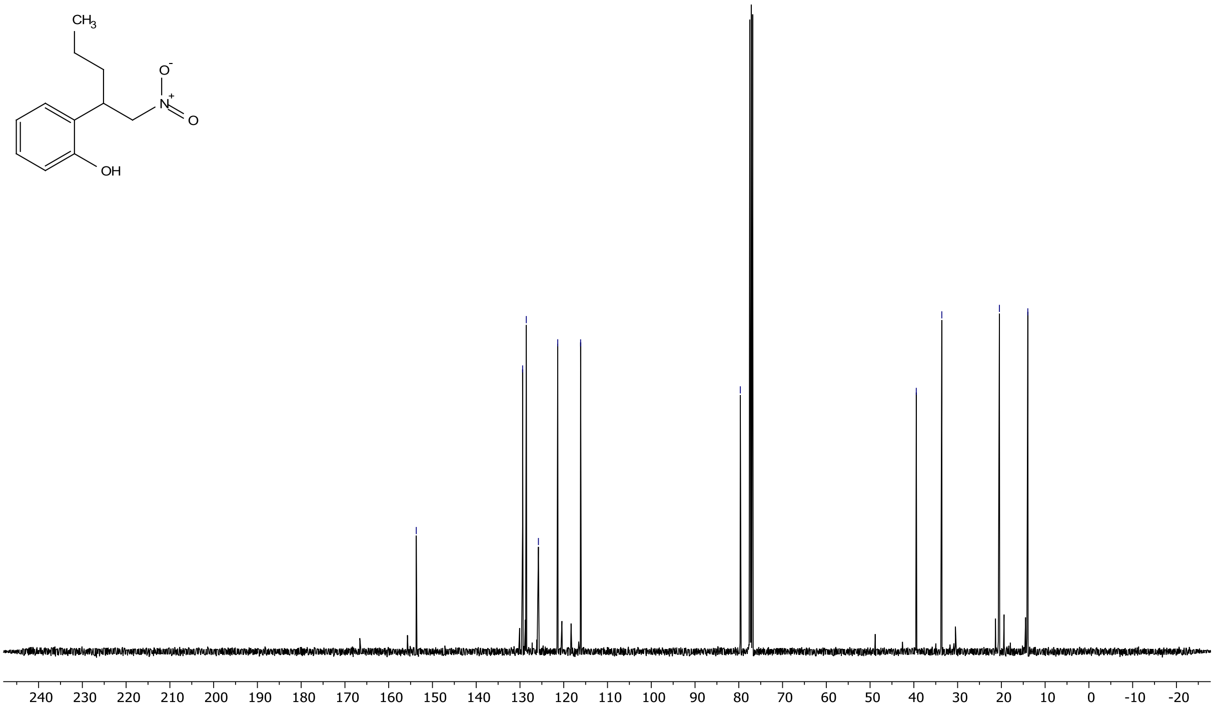

1H NMR(400 MHz, Chloroform-d)

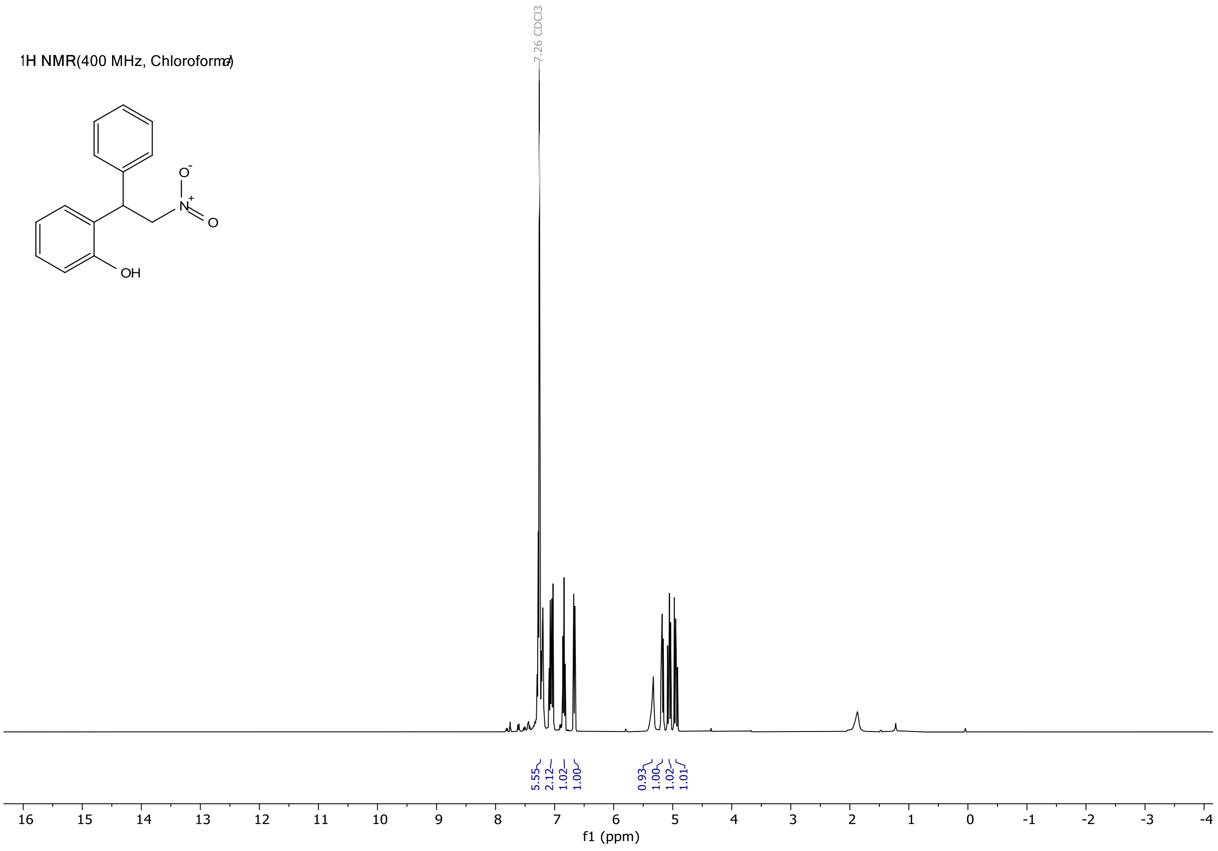

13C{1H} NMR(101 MHz, Chloroform-d)

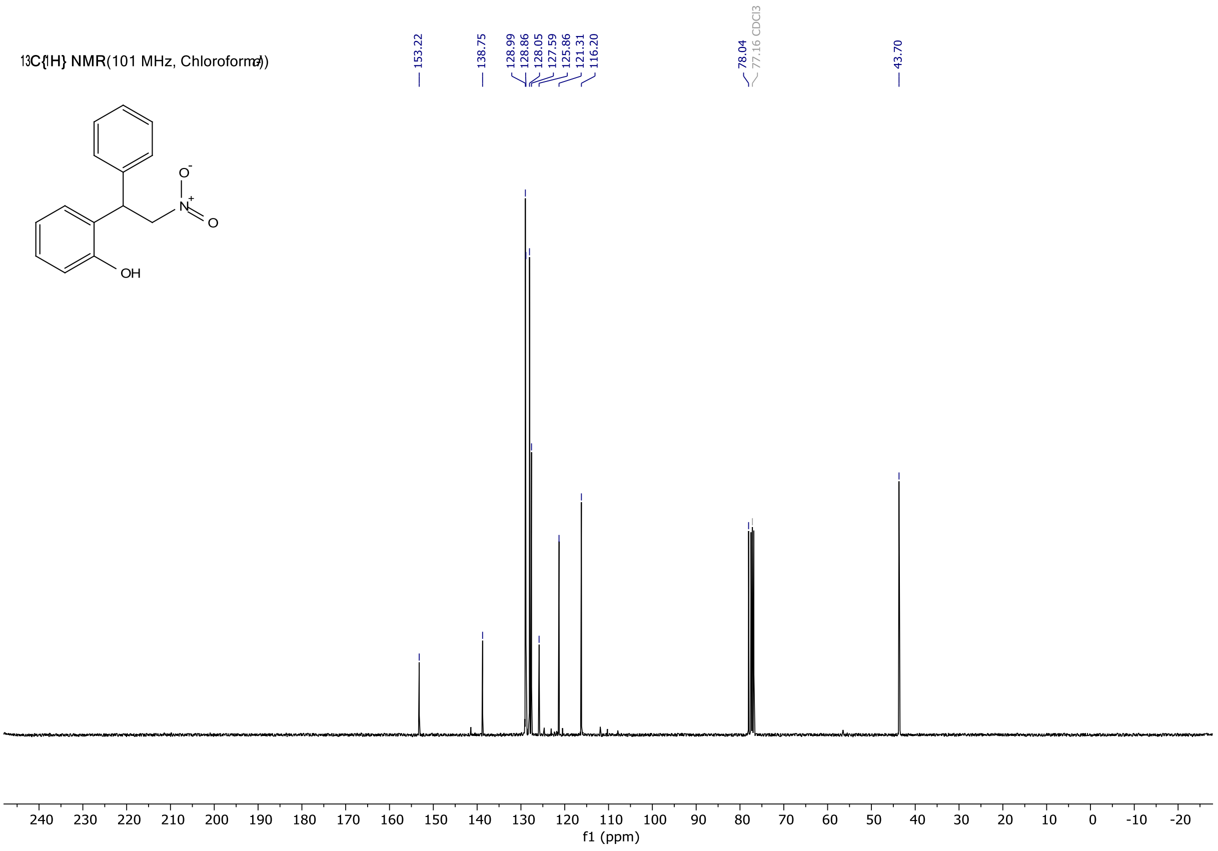

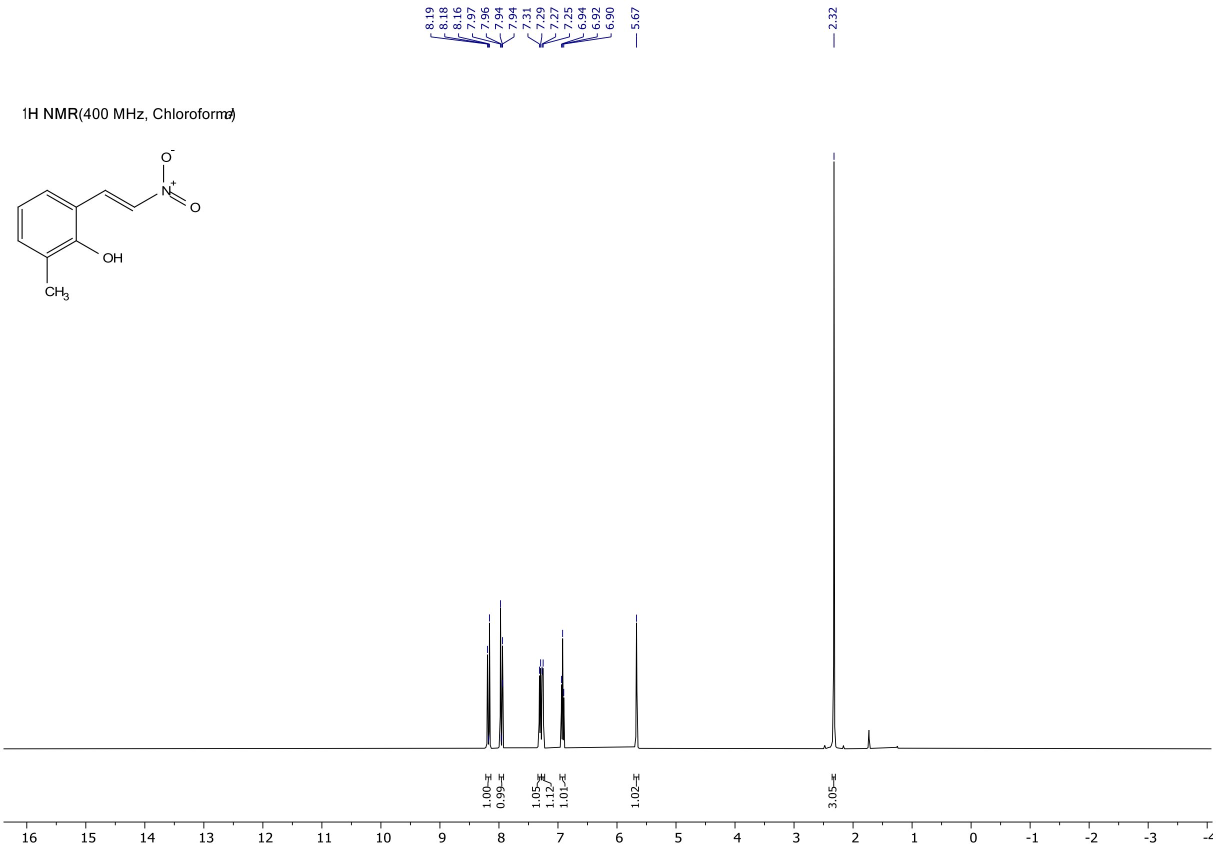
1H NMR(400 MHz, Chloroform-d)
8.19 8.18 8.16 7.97 7.96 7.94 7.94 7.31 7.29 7.27 7.25 6.94 6.92 6.90
5.67
2.32
OH
CH3
1.00 0.99 1.05 1.12 1.01 1.02 3.05
16 15 14 13 12 11 10 9 8 7 6 5 4 3 2 1 0 -1 -2 -3

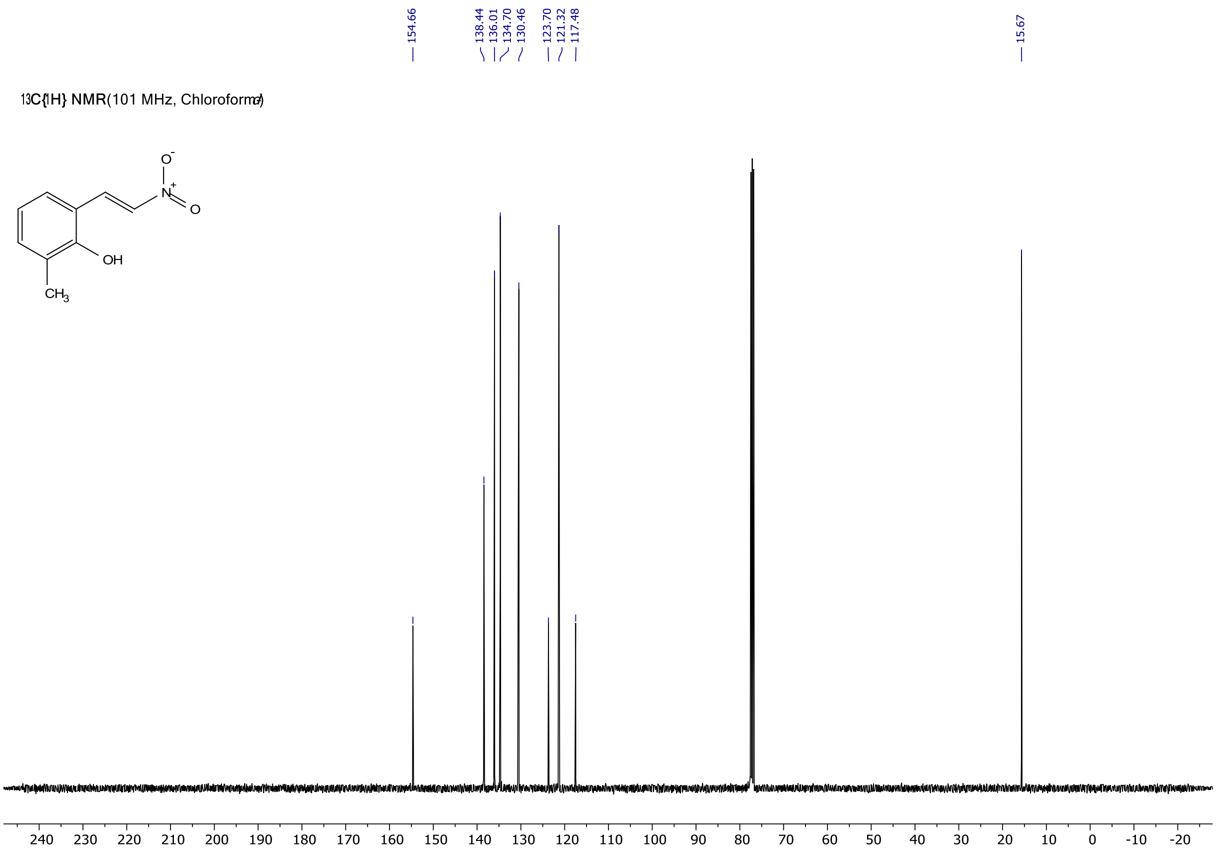
13C{1H} NMR(101 MHz, Chloroform-d)
154.66
138.44 136.01 134.70 130.46
123.70 121.32 117.48
15.67
OH
CH3
240 230 220 210 200 190 180 170 160 150 140 130 120 110 100 90 80 70 60 50 40 30 20 10 0 -10 -20

1H NMR(400 MHz, Chloroform-*d*)

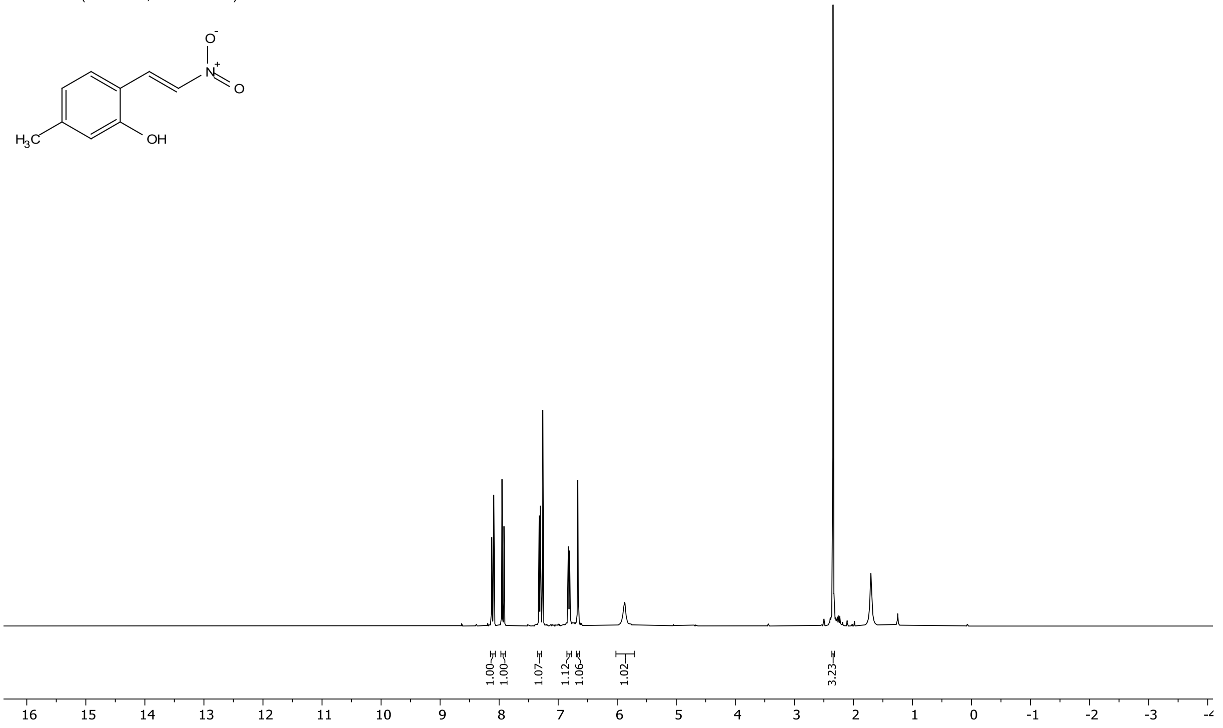

13C{1H} NMR(101 MHz, Chloroform-*d*)

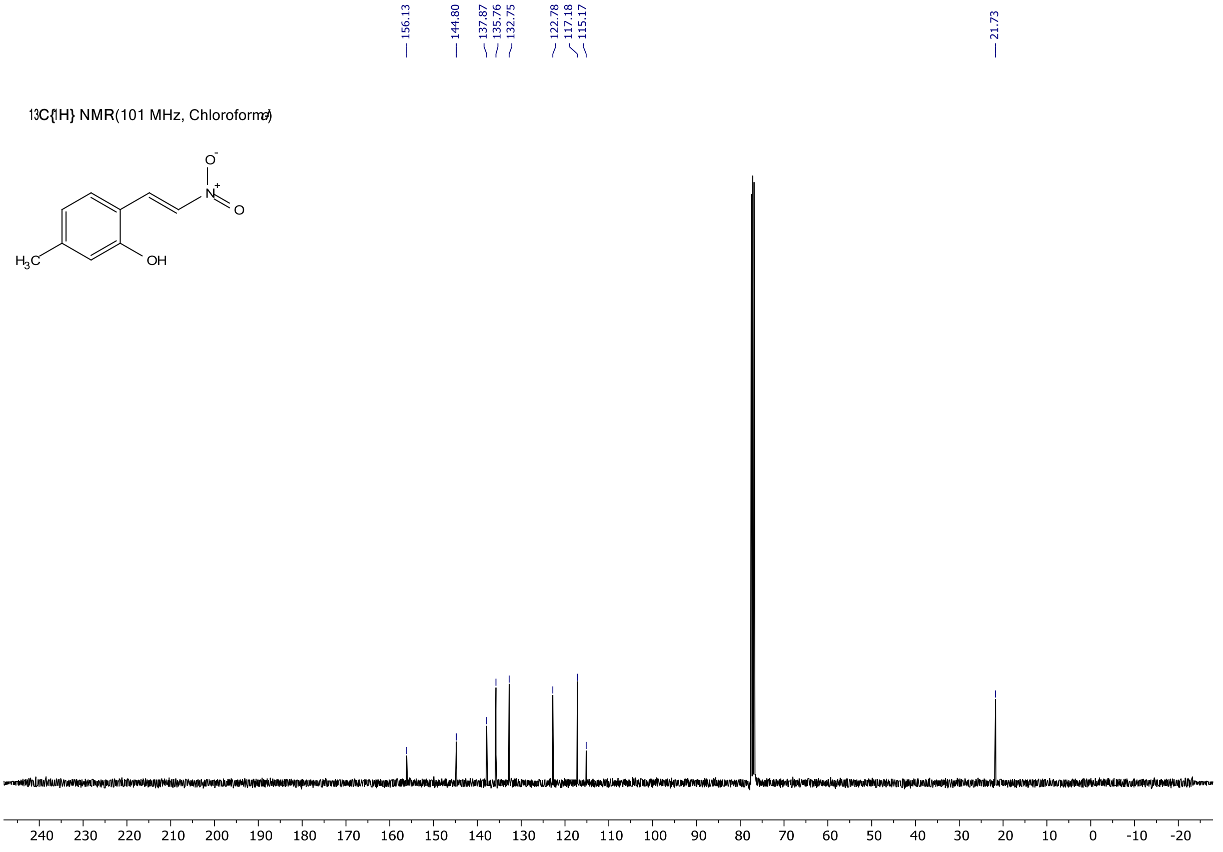

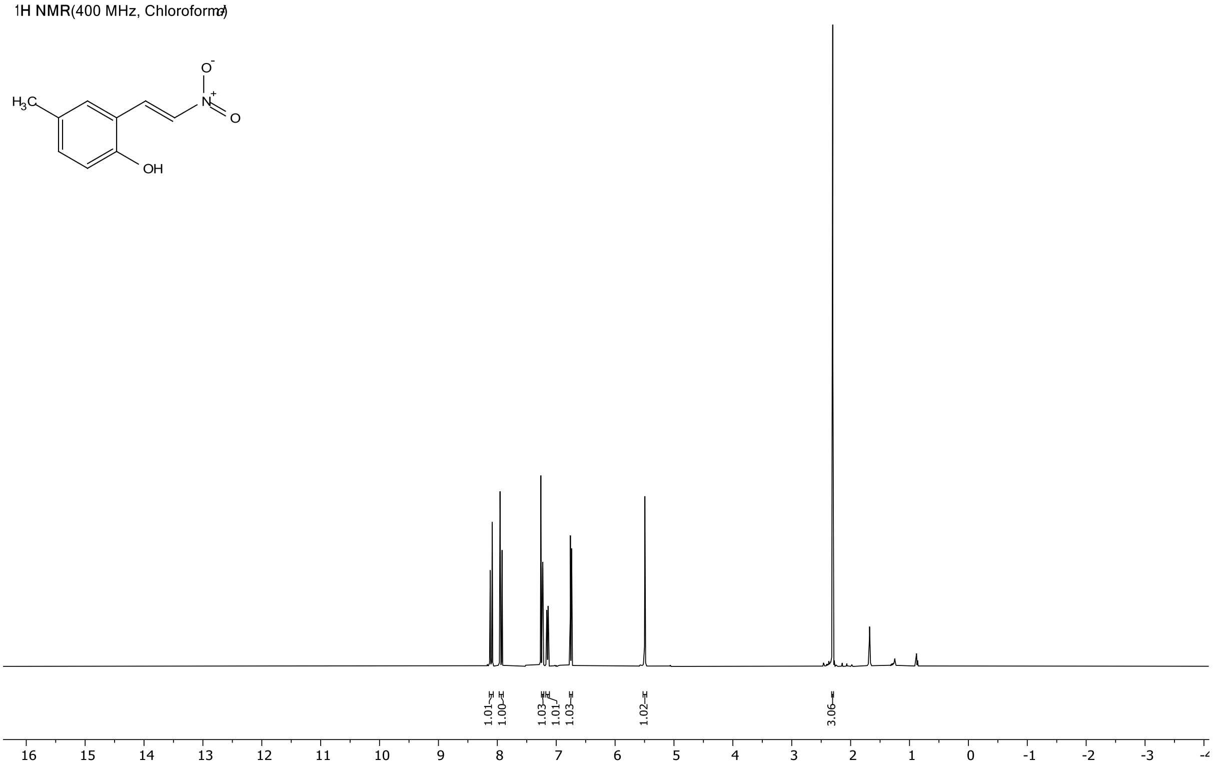
1H NMR(400 MHz, Chloroform-d)
H3C
O-
N+
O
OH
1.01
1.00
1.03
1.01
1.03
1.02
3.06
16 15 14 13 12 11 10 9 8 7 6 5 4 3 2 1 0 -1 -2 -3

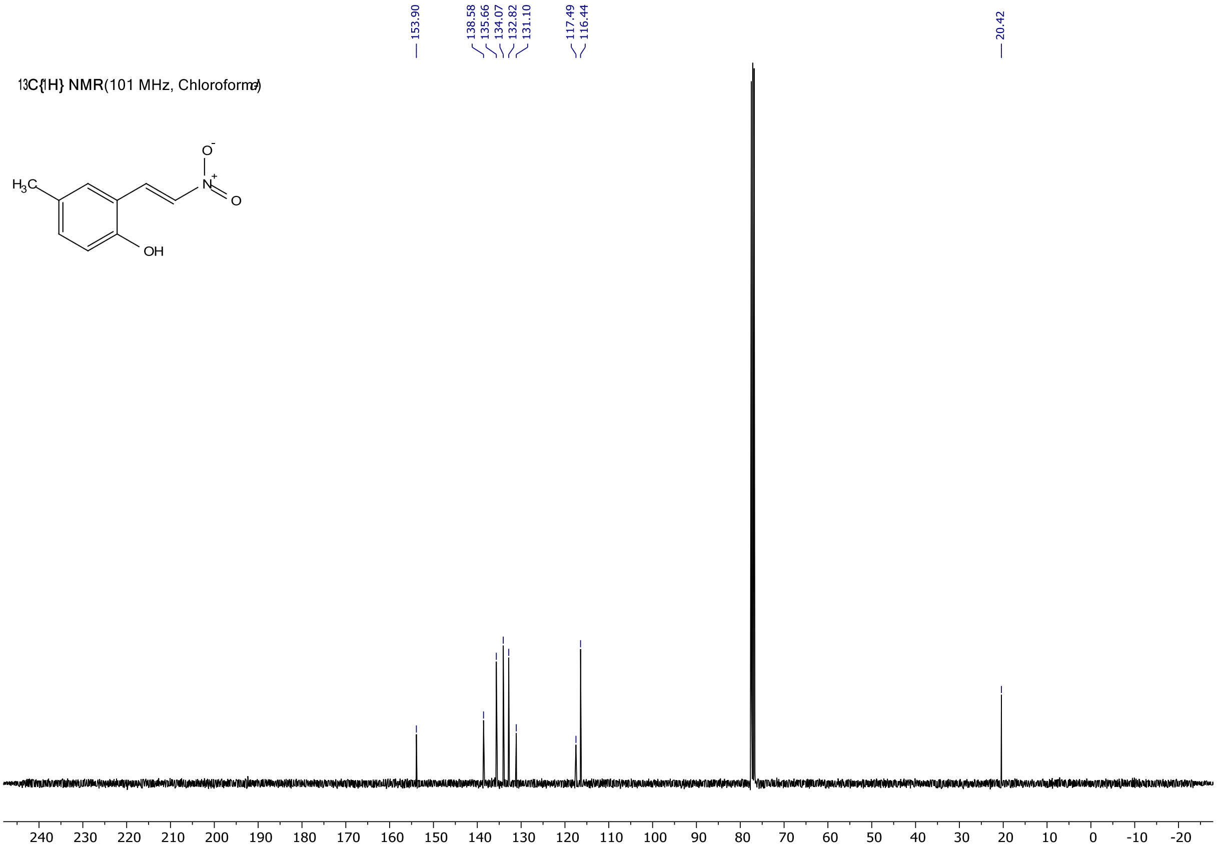
153.90
138.58
135.66
134.07
132.82
131.10
117.49
116.44
20.42
13C{1H} NMR(101 MHz, Chloroform-d)
H3C
O-
N+
O
OH
240 230 220 210 200 190 180 170 160 150 140 130 120 110 100 90 80 70 60 50 40 30 20 10 0 -10 -20

1H NMR(400 MHz, Chloroform-d)

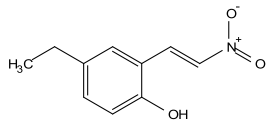

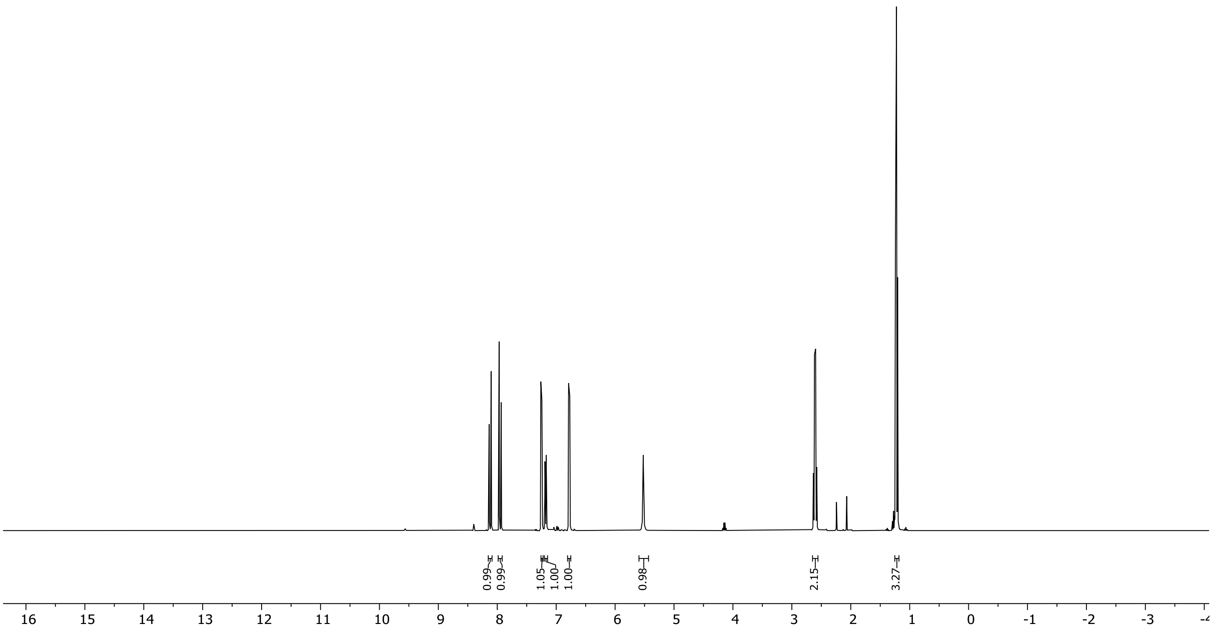

13C{1H} NMR(101 MHz, Chloroform-d)

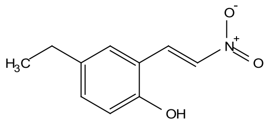

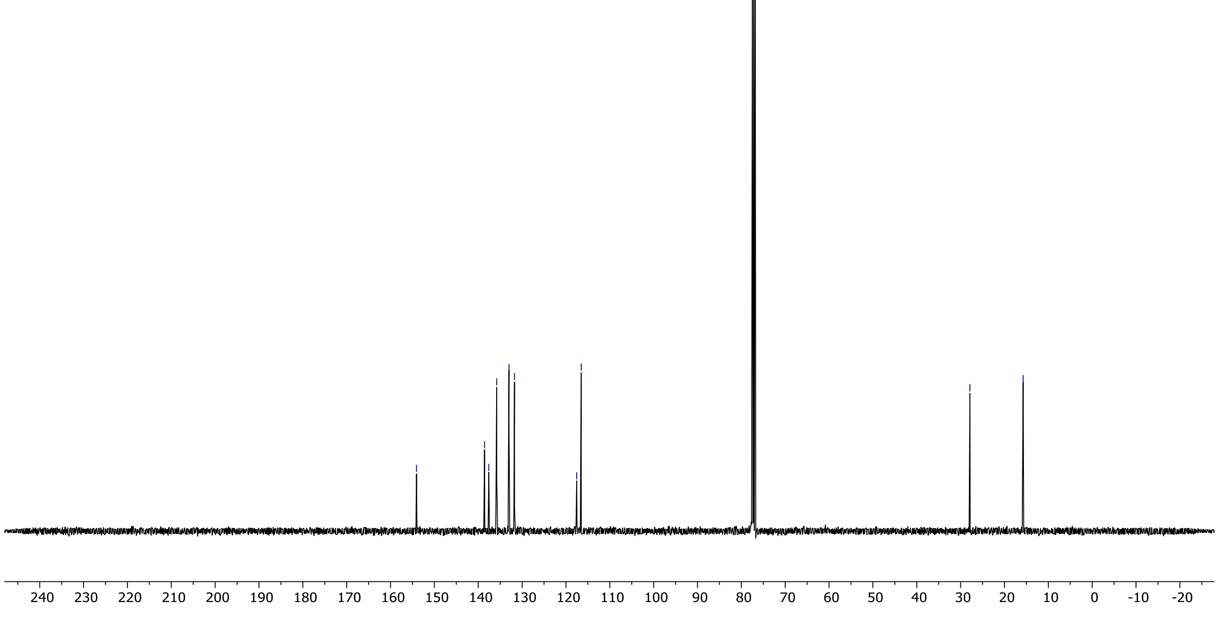

1H NMR(400 MHz, Chloroform-d)

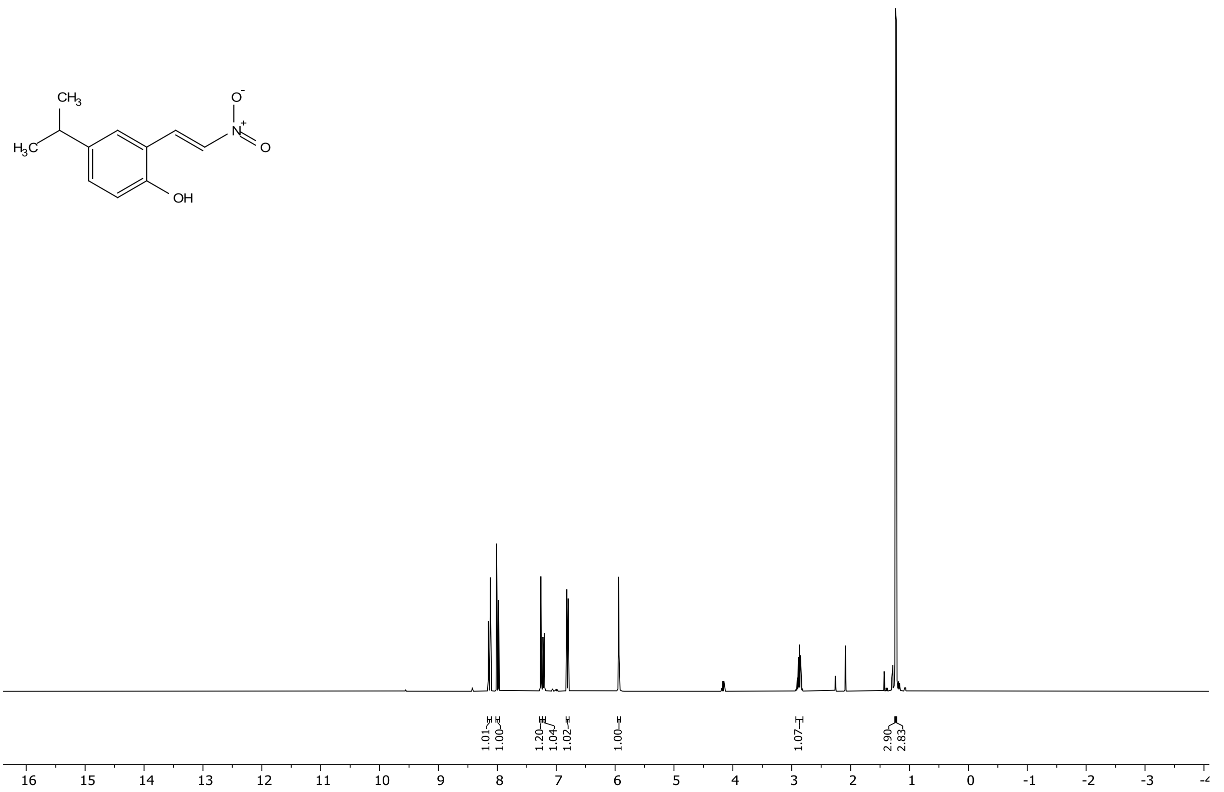

13C{1H} NMR(101 MHz, Chloroform-d)

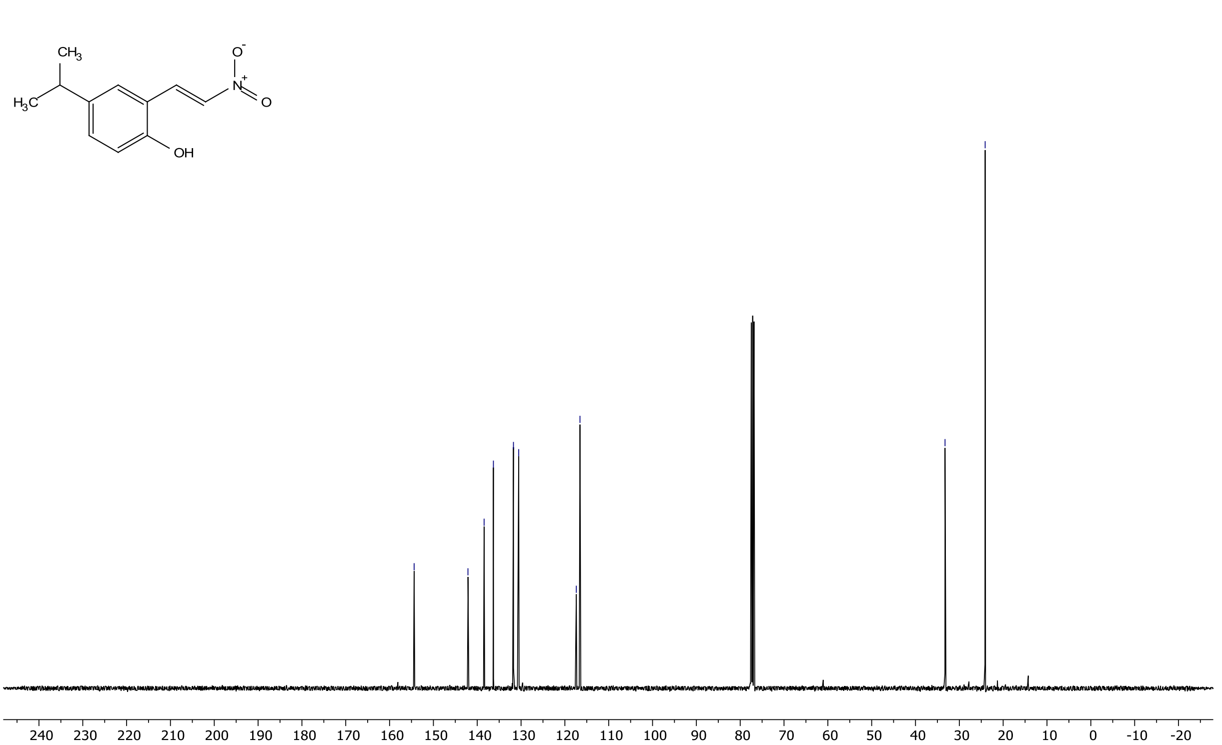

1H NMR(400 MHz, Chloroform-d)

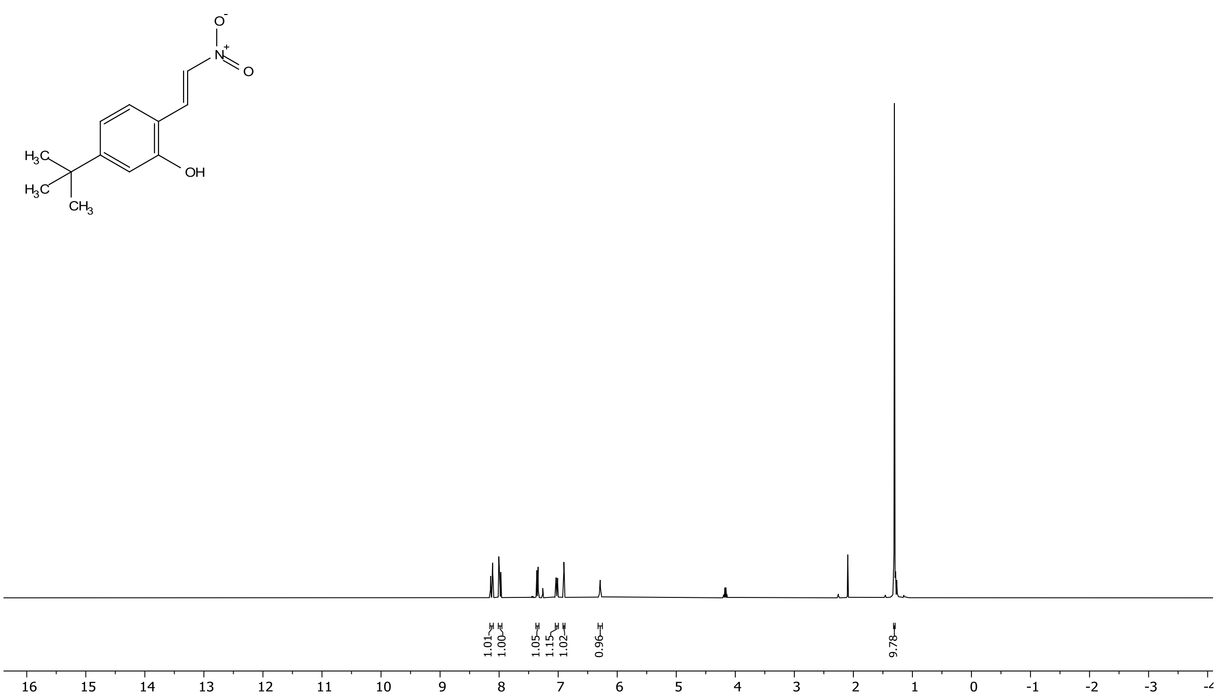

13C{1H} NMR(101 MHz, Chloroform-d)

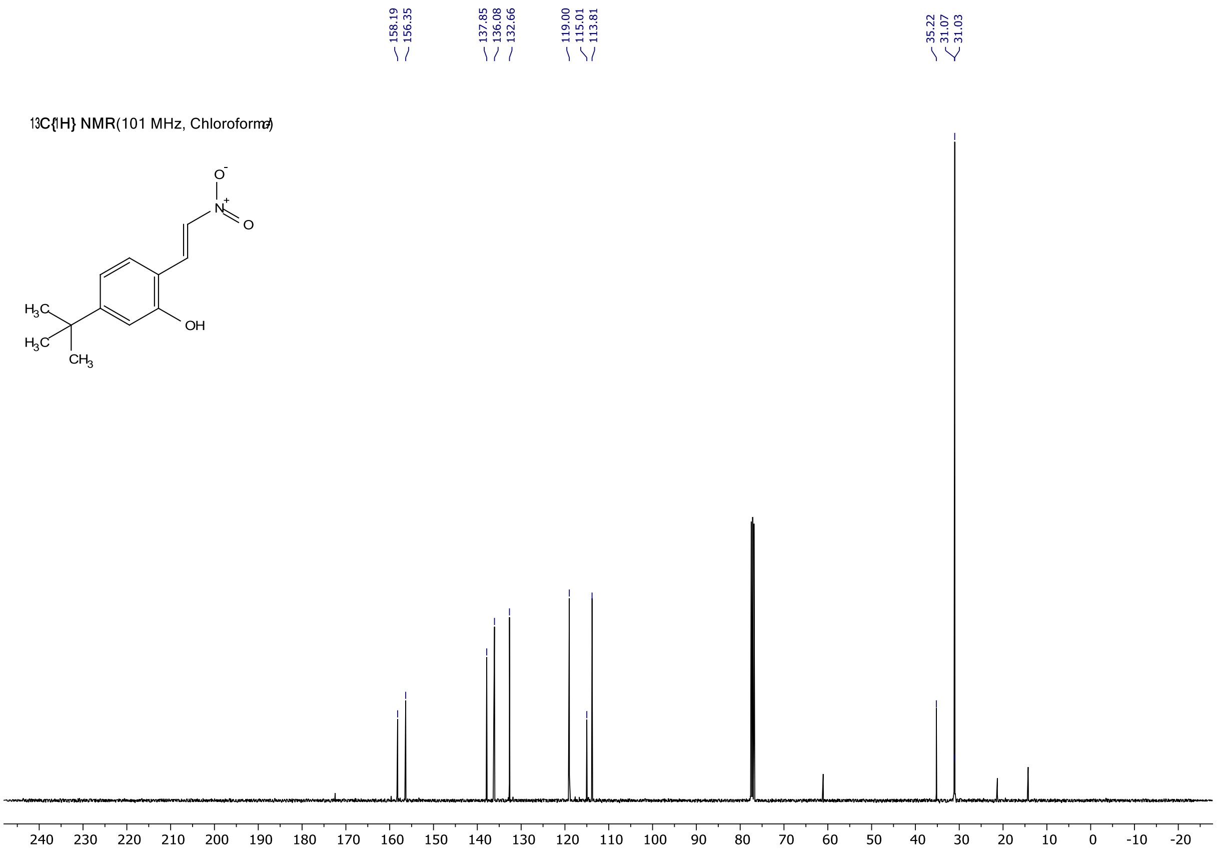

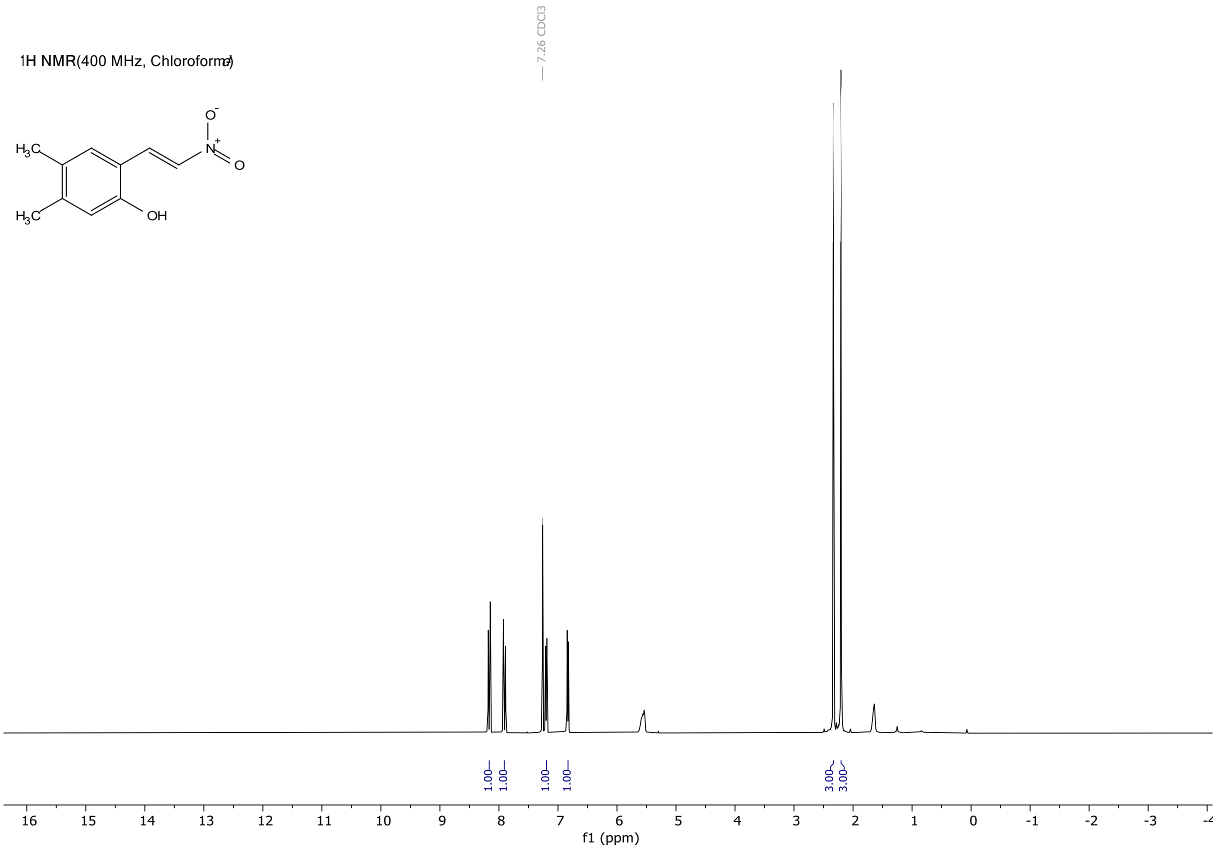
1H NMR(400 MHz, Chloroform-d)
7.26 CDCl3
1.00
1.00
1.00
1.00
3.00
3.00
f1 (ppm)

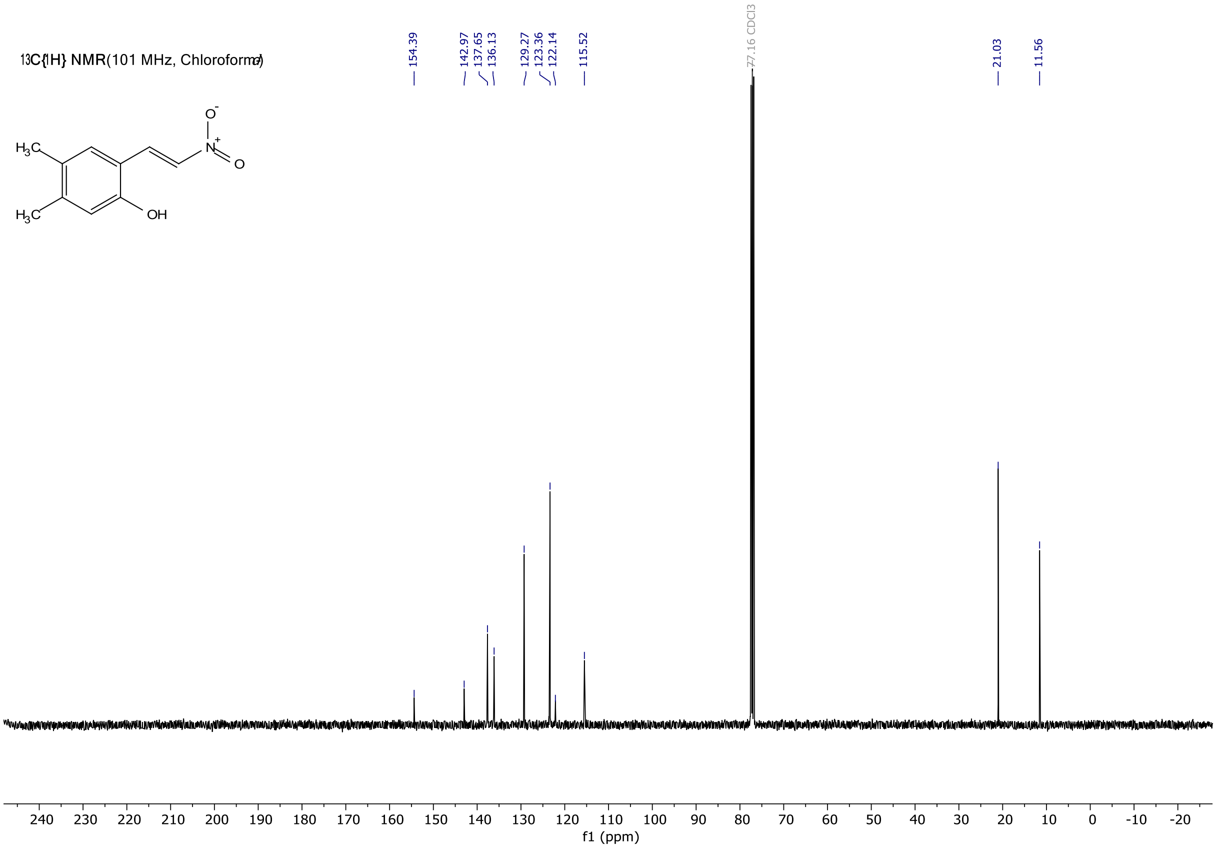
13C{1H} NMR(101 MHz, Chloroform-d)
154.39
142.97
137.65
136.13
129.27
123.36
122.14
115.52
77.16 CDCl3
21.03
11.56
f1 (ppm)

1H NMR(400 MHz, Methanol-d_4)

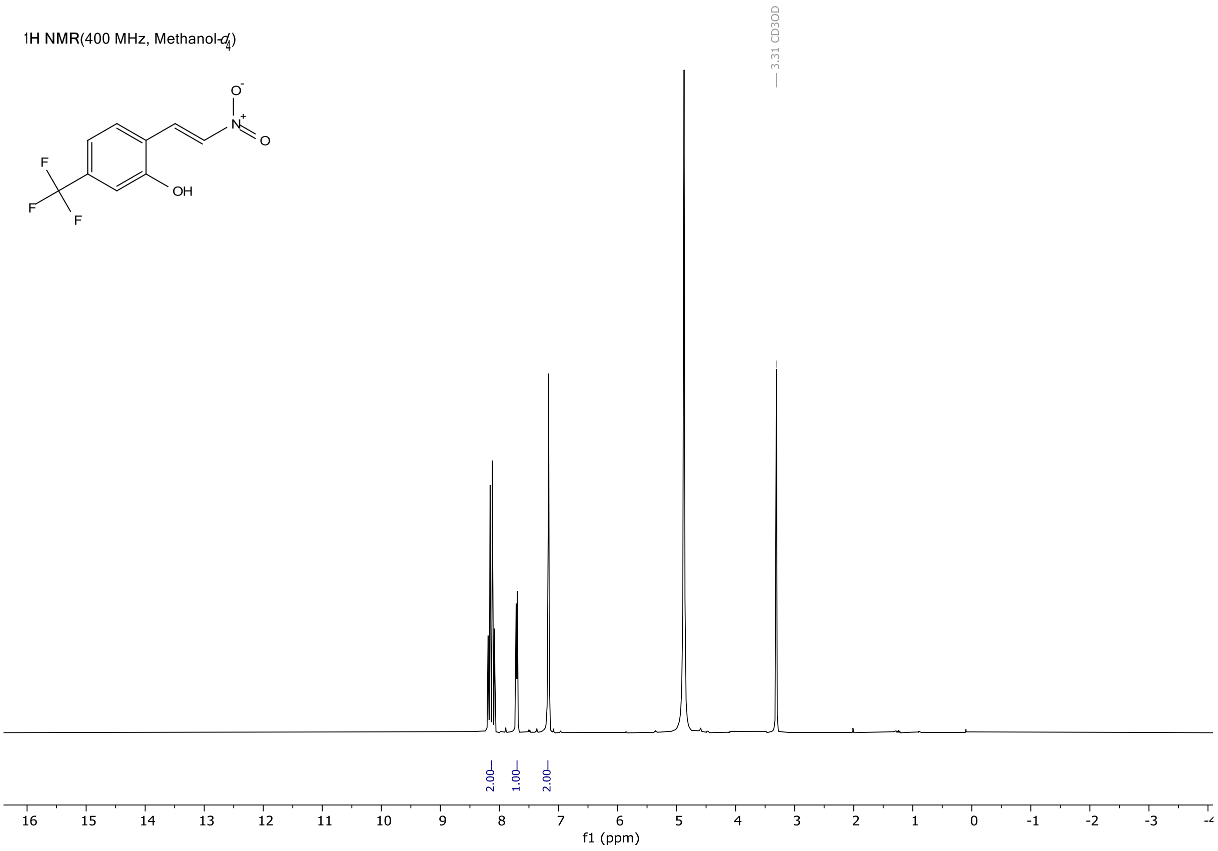

13C{1H} NMR(101 MHz, Methanol-d_4)

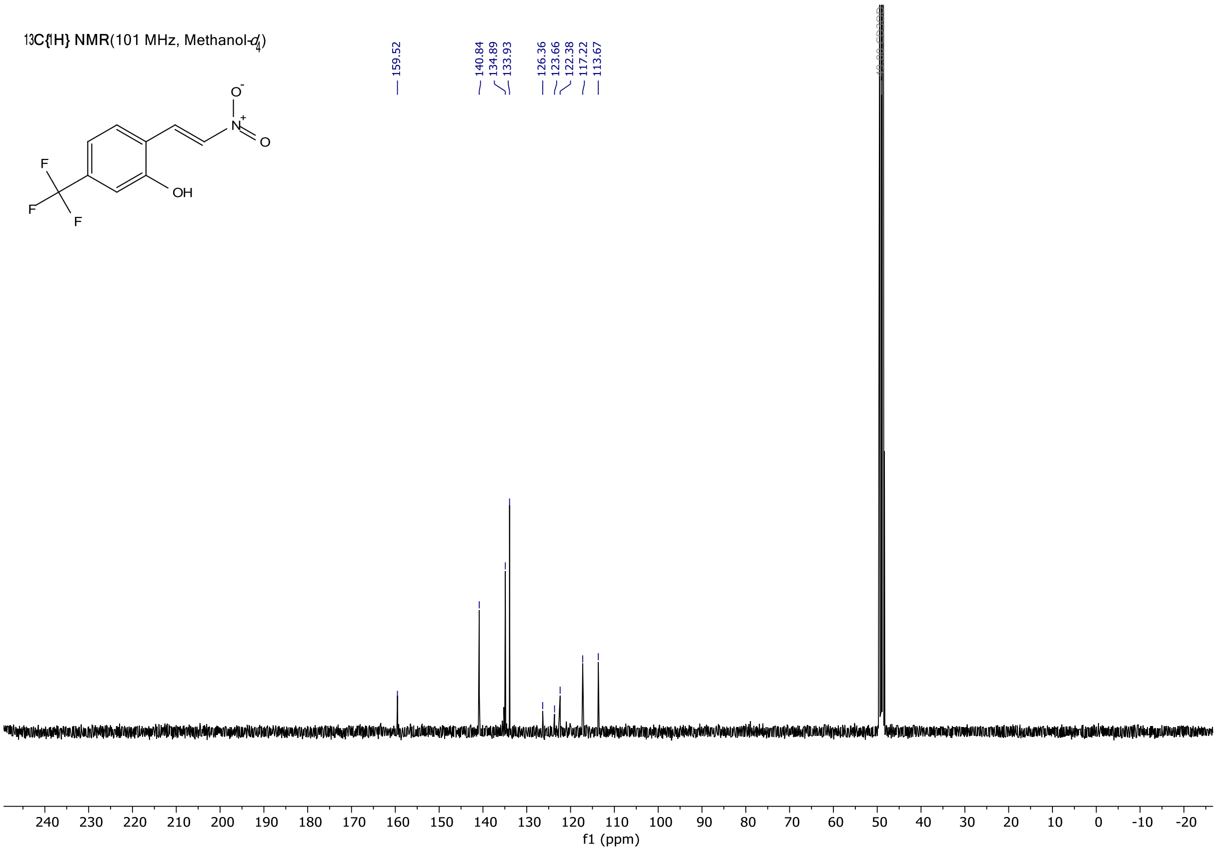

19F{1H}-NMR(376 MHz, Chloroform-d)

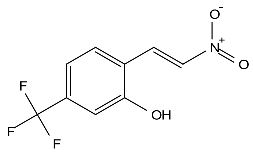

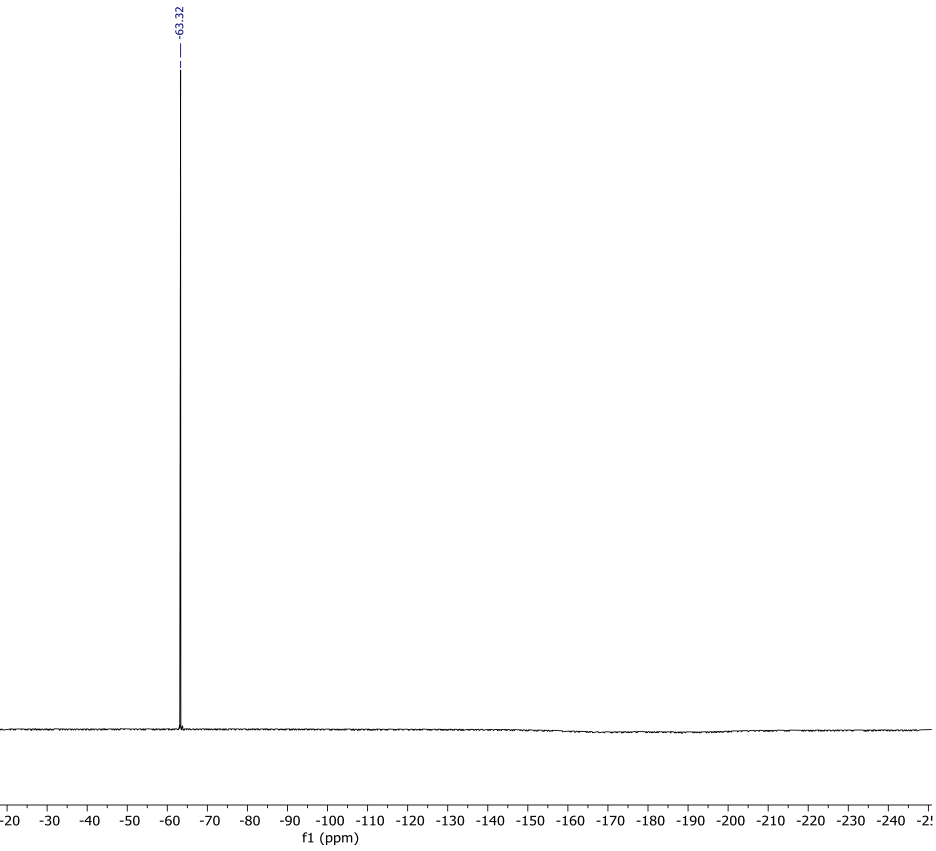

1H NMR(400 MHz, Dimethyl sulfoxide-*d6*)

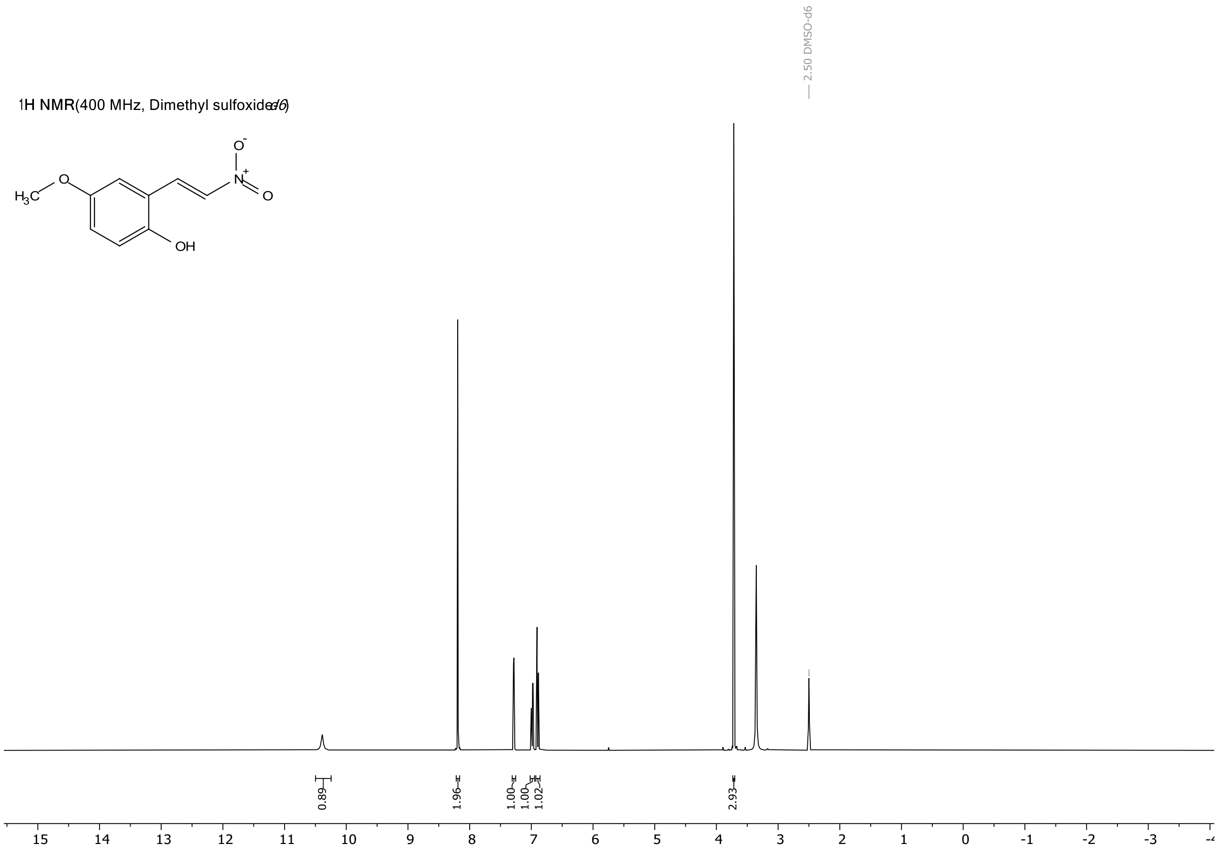

13C{1H} NMR(101 MHz, Dimethyl sulfoxide-*d6*)

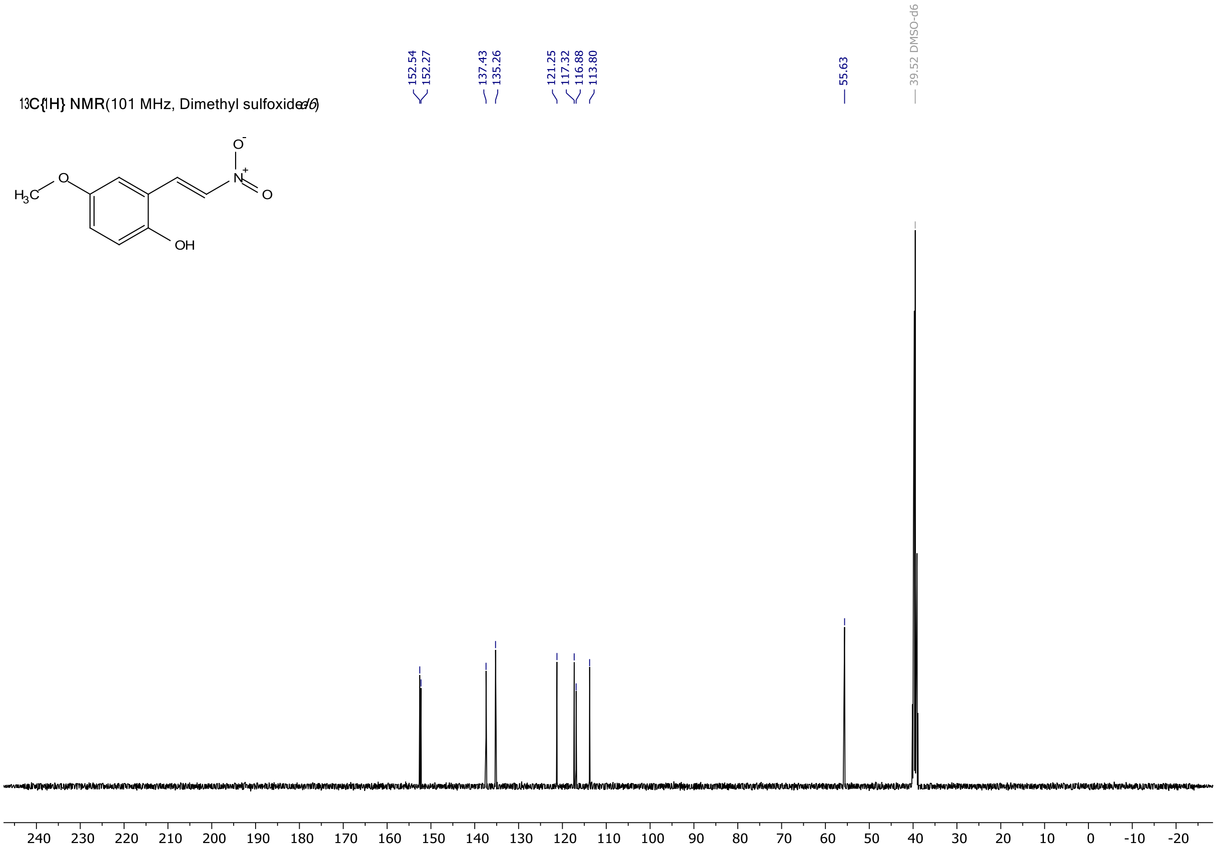

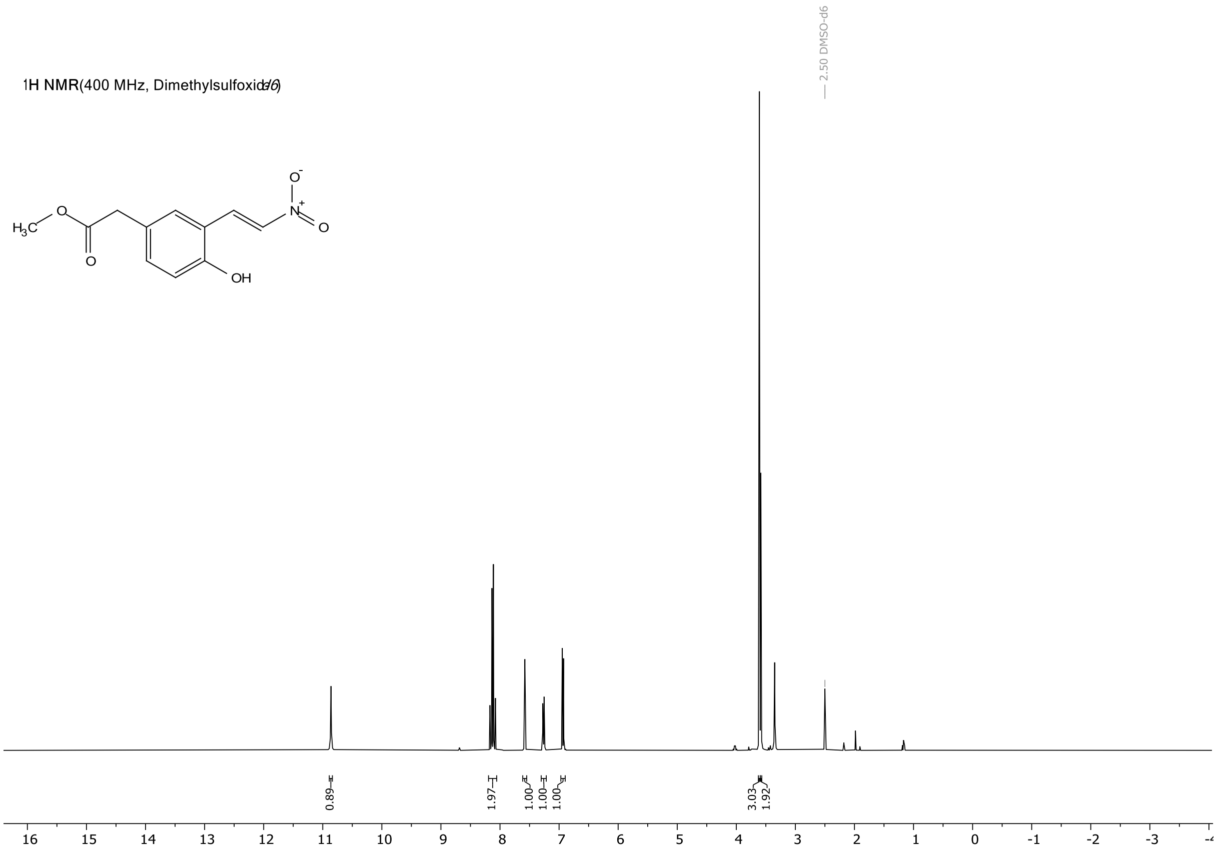

1H NMR(400 MHz, Dimethylsulfoxide-d6)
2.50 DMSO-d6
H3C O O O- N+ O OH
0.89
1.97
1.00
1.00
1.00
3.03
1.92
16 15 14 13 12 11 10 9 8 7 6 5 4 3 2 1 0 -1 -2 -3

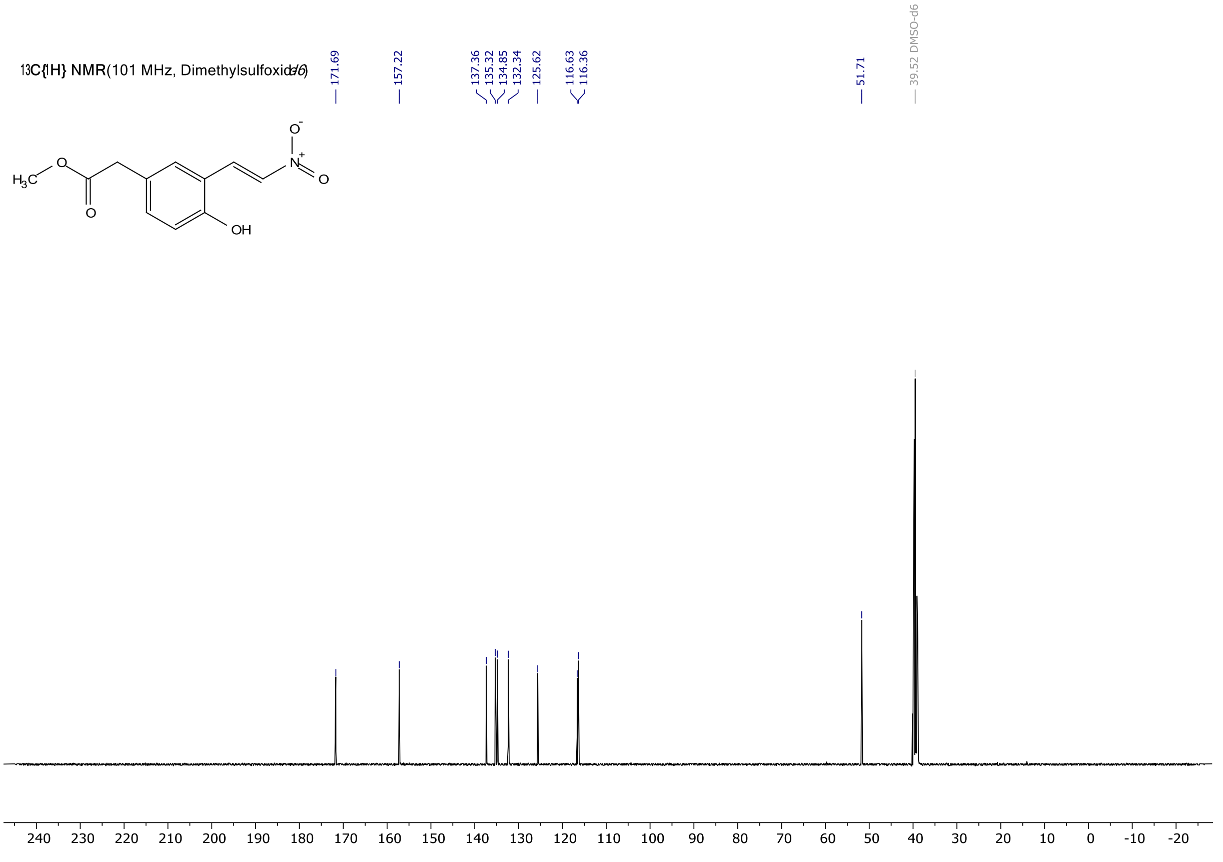

13C{1H} NMR(101 MHz, Dimethylsulfoxide-d6)
171.69
157.22
137.36
135.32
134.85
132.34
125.62
116.63
116.36
51.71
39.52 DMSO-d6
H3C O O O- N+ O OH
240 230 220 210 200 190 180 170 160 150 140 130 120 110 100 90 80 70 60 50 40 30 20 10 0 -10 -20

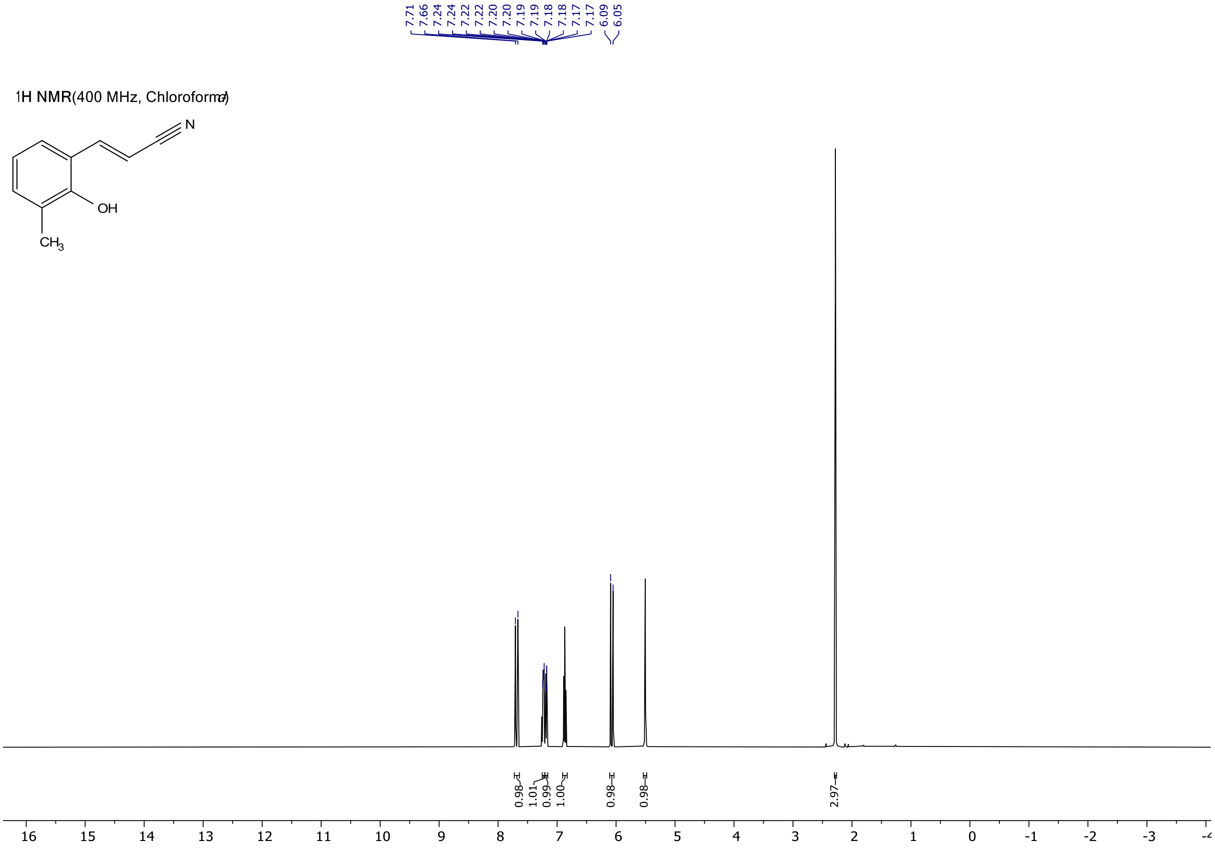
7.71
7.66
7.24
7.24
7.22
7.22
7.20
7.20
7.19
7.19
7.18
7.18
7.17
7.17
6.09
6.05
1H NMR(400 MHz, Chloroform-d)
N
OH
CH3
0.98
1.01
0.99
1.00
0.98
0.98
2.97
16
15
14
13
12
11
10
9
8
7
6
5
4
3
2
1
0
-1
-2
-3

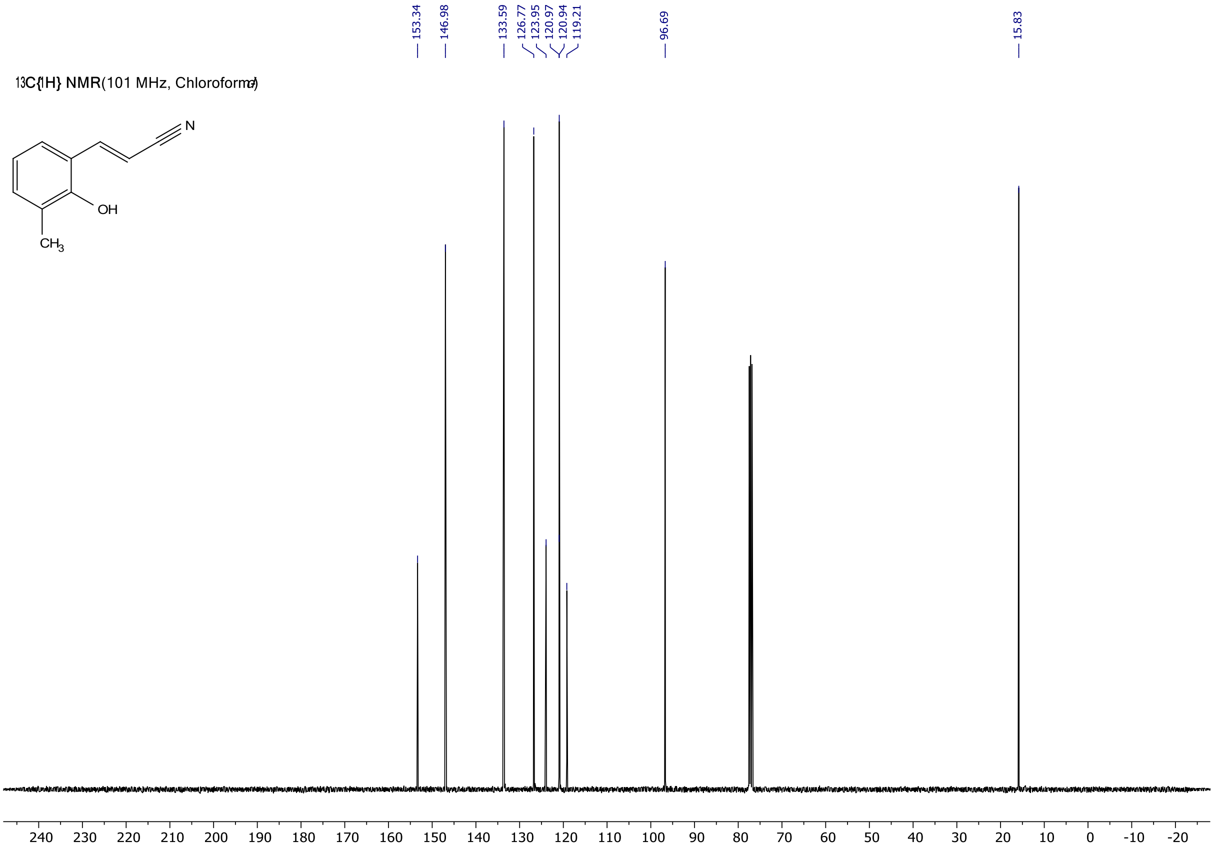
153.34
146.98
133.59
126.77
123.95
120.97
120.94
119.21
96.69
15.83
13C{1H} NMR(101 MHz, Chloroform-d)
N
OH
CH3
240
230
220
210
200
190
180
170
160
150
140
130
120
110
100
90
80
70
60
50
40
30
20
10
0
-10
-20

1H NMR(400 MHz, Chloroform-d)

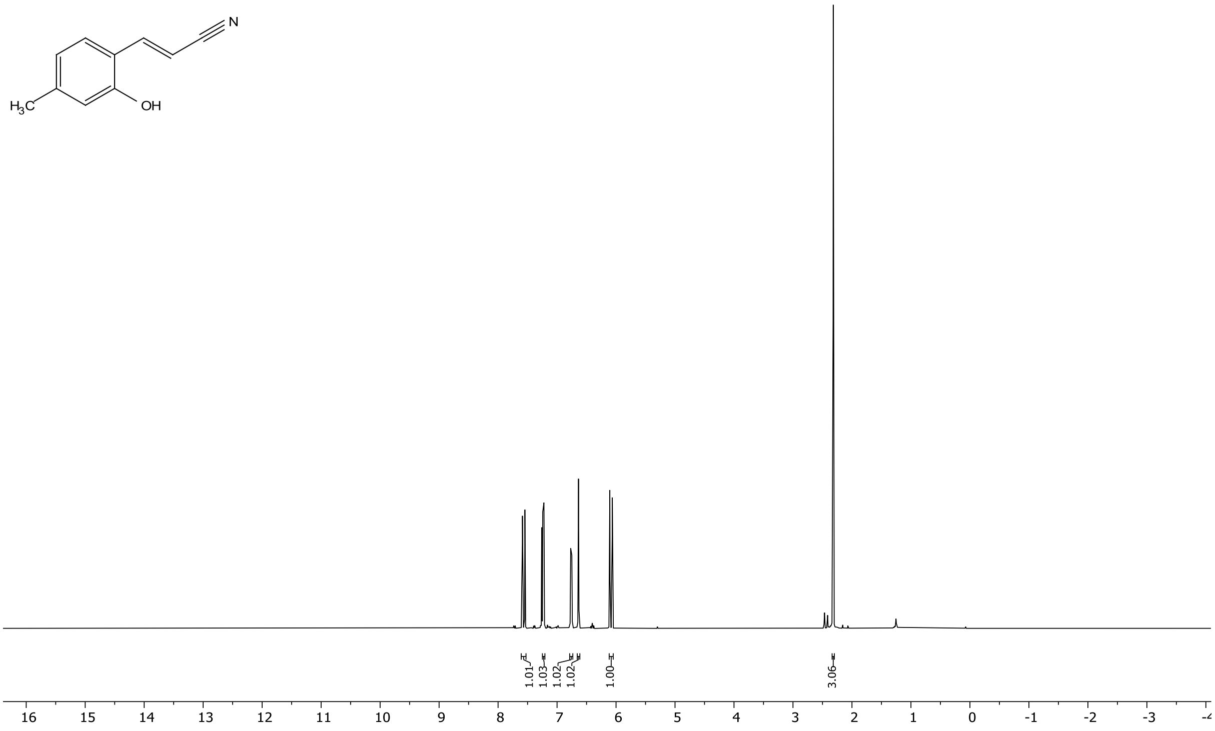

13C{1H} NMR(101 MHz, Chloroform-d)

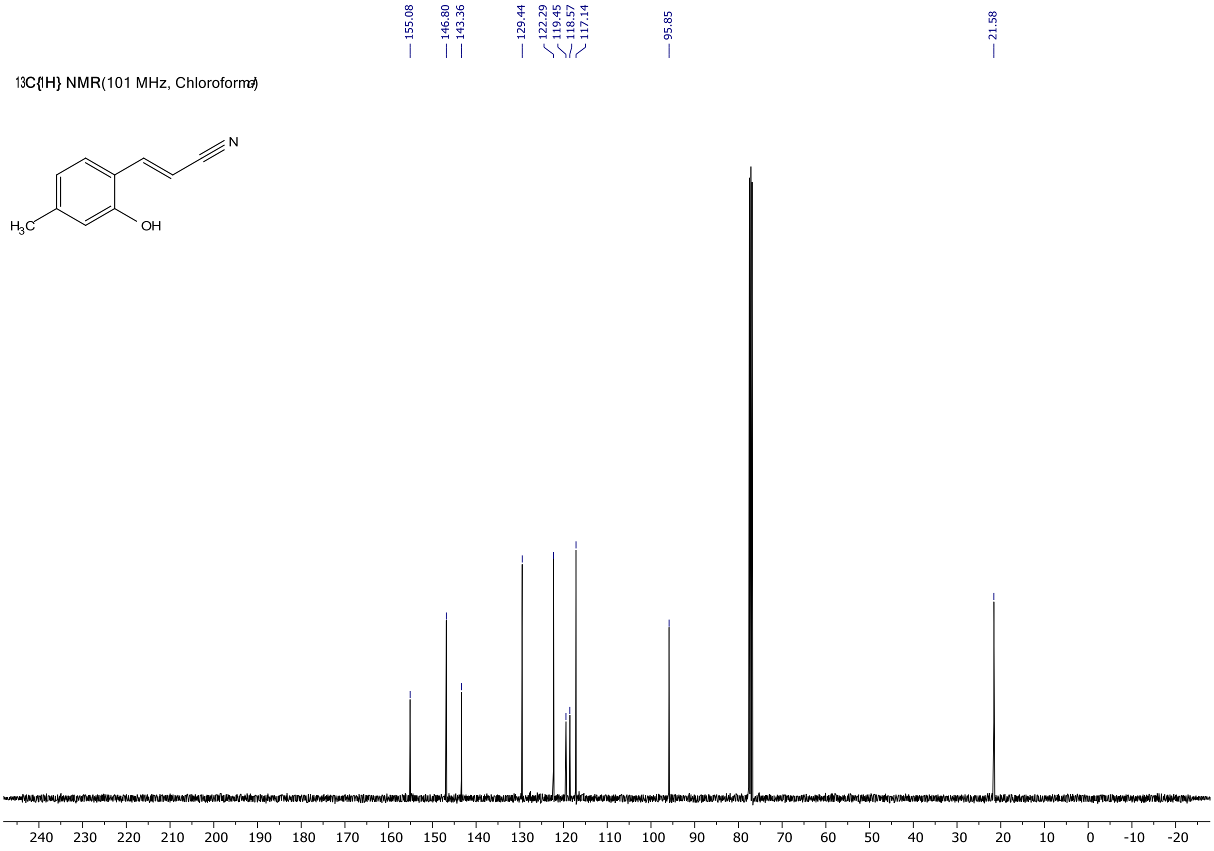

1H NMR(400 MHz, Chloroform-d)

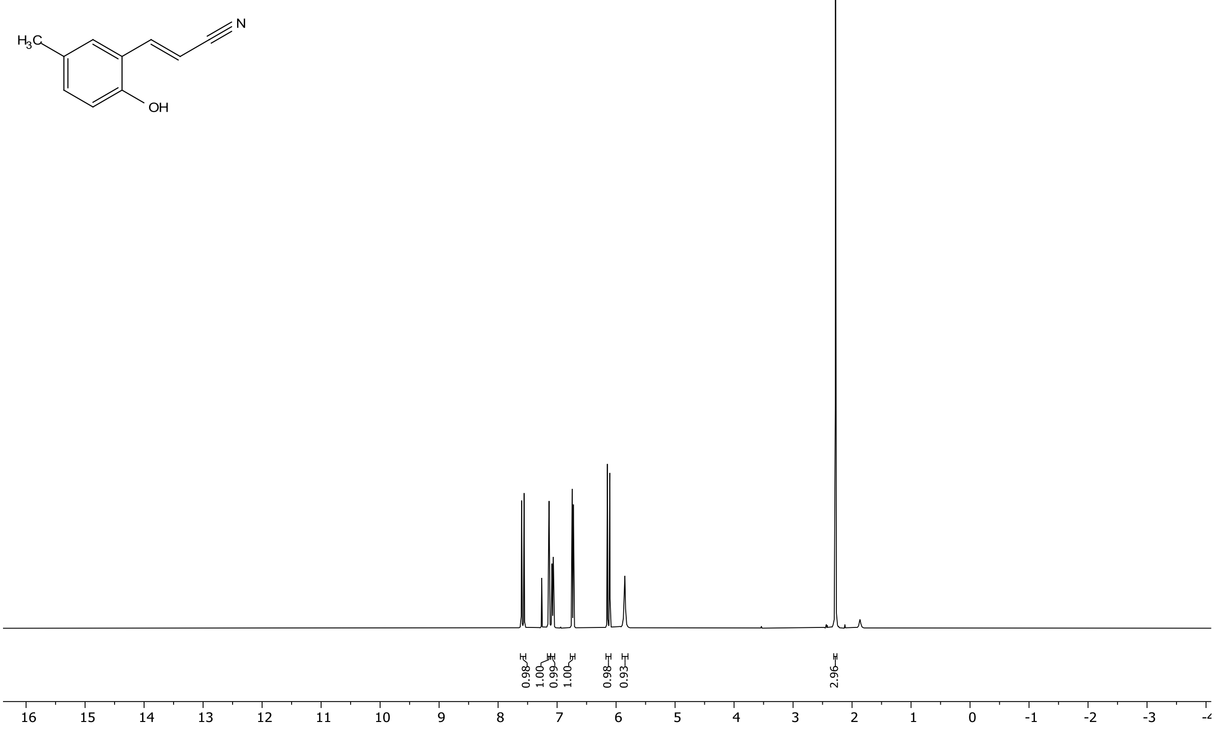

13C{1H} NMR(101 MHz, Chloroform-d)

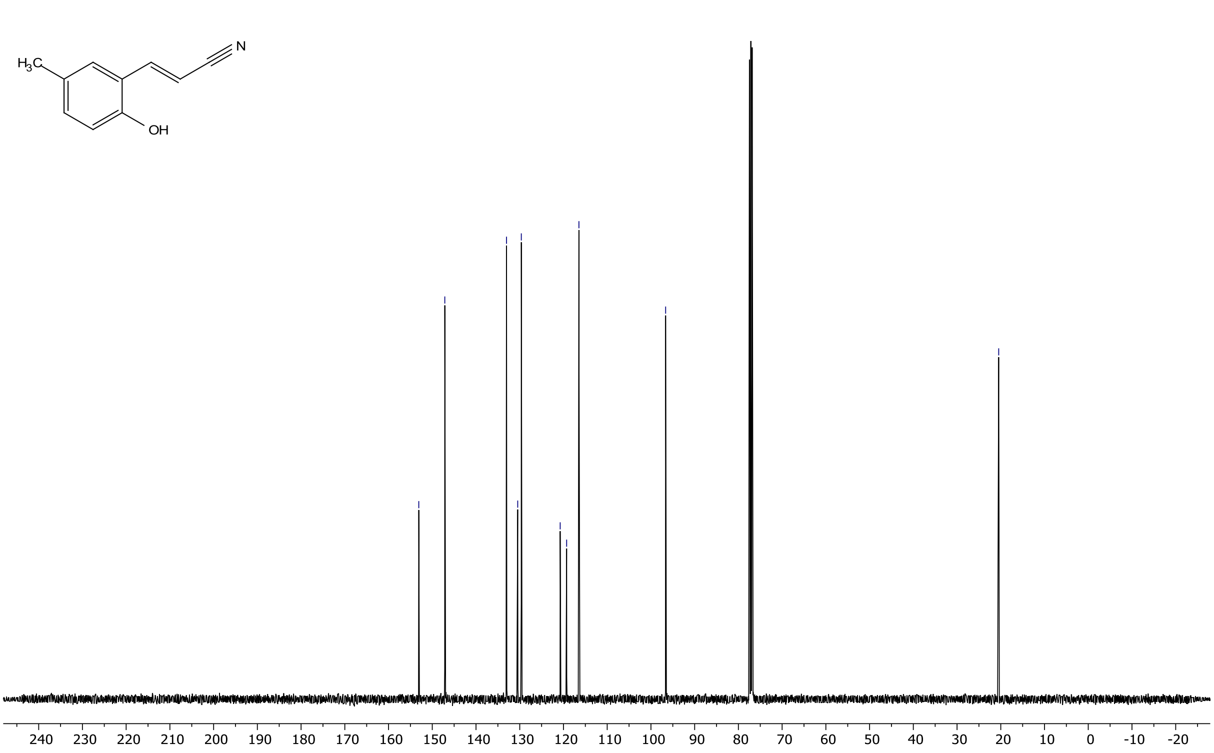

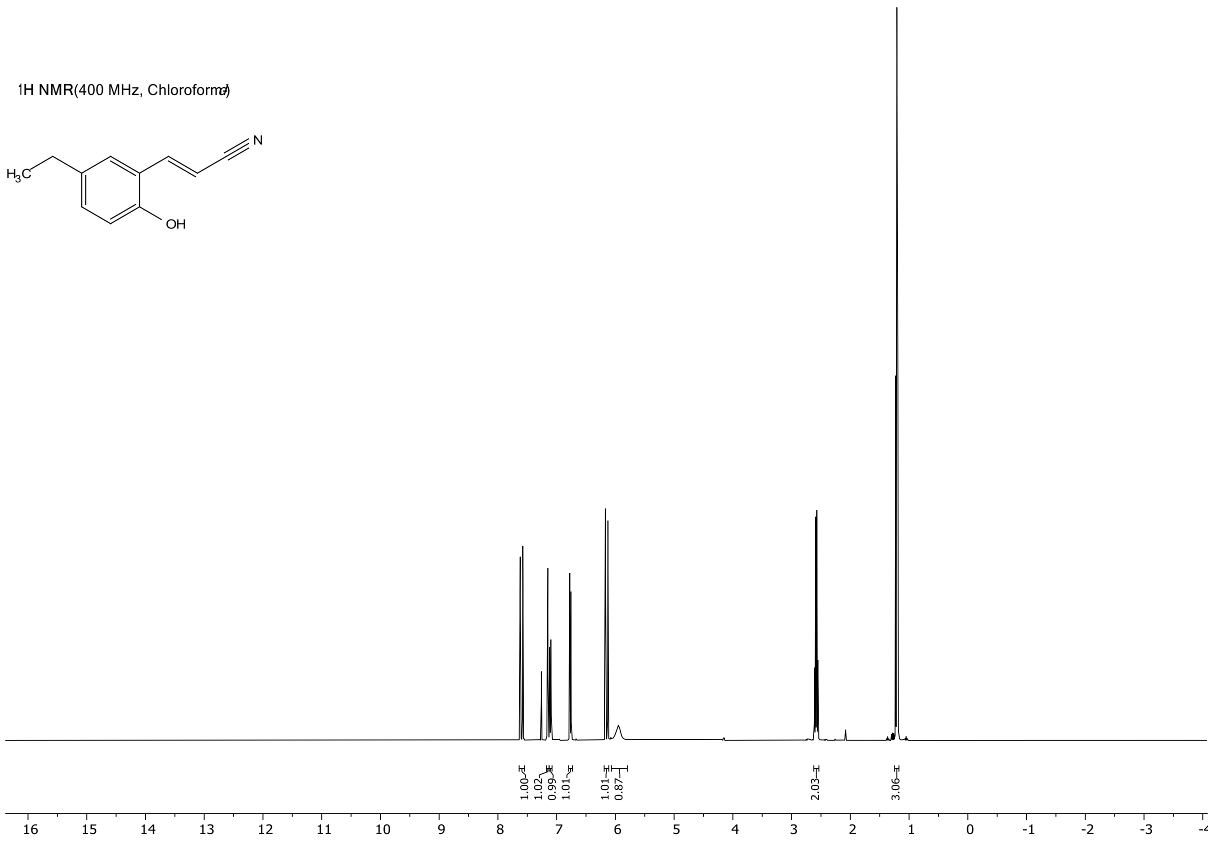
1H NMR(400 MHz, Chloroform-d)
N
H3C
OH
1.00
1.02
0.99
1.01
1.01
0.87
2.03
3.06
16 15 14 13 12 11 10 9 8 7 6 5 4 3 2 1 0 -1 -2 -3

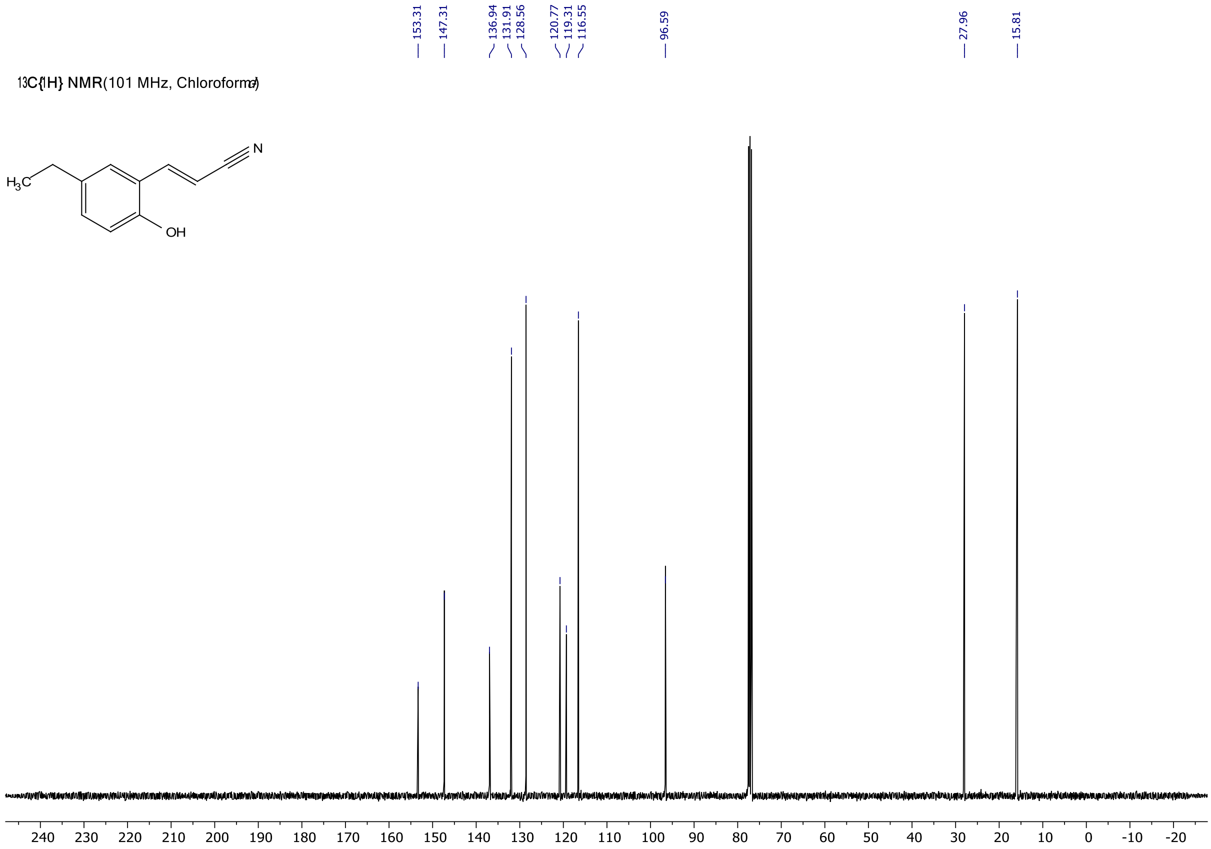
153.31
147.31
136.94
131.91
128.56
120.77
119.31
116.55
96.59
27.96
15.81
13C{1H} NMR(101 MHz, Chloroform-d)
N
H3C
OH
240 230 220 210 200 190 180 170 160 150 140 130 120 110 100 90 80 70 60 50 40 30 20 10 0 -10 -20

1H NMR(400 MHz, Chloroform-d)

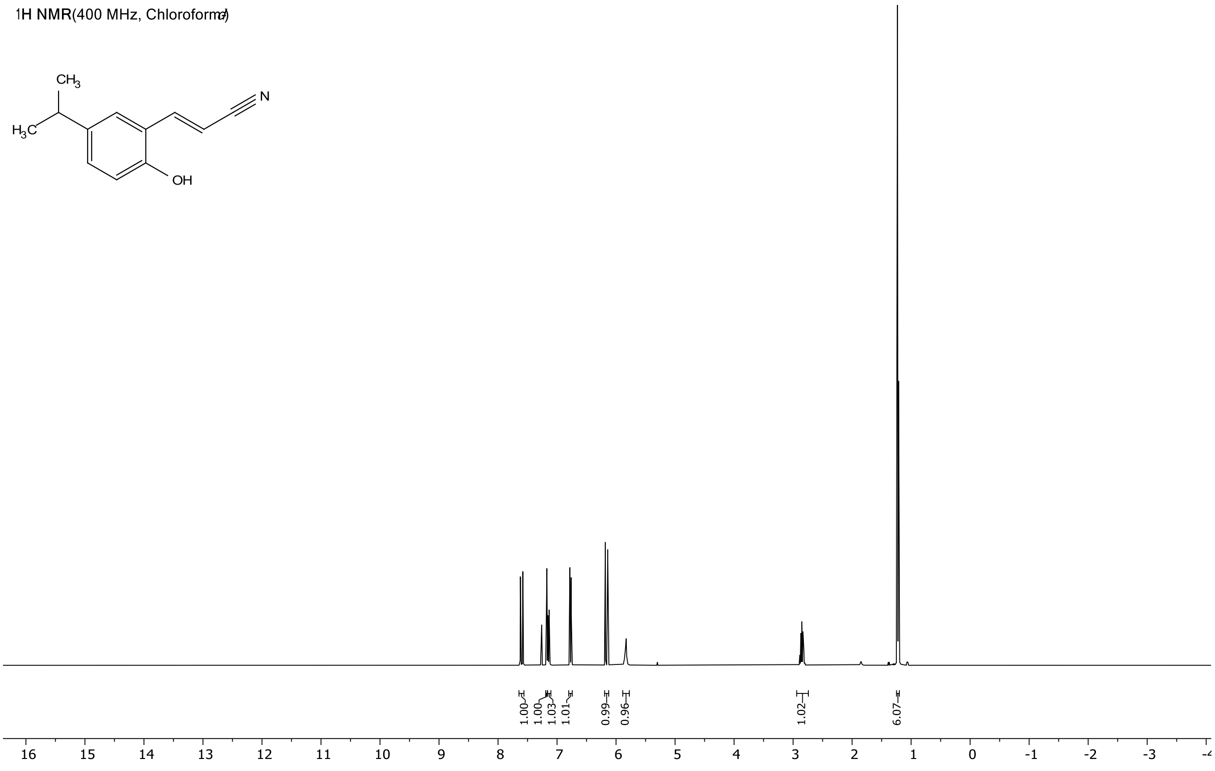

13C{1H} NMR(101 MHz, Chloroform-d)

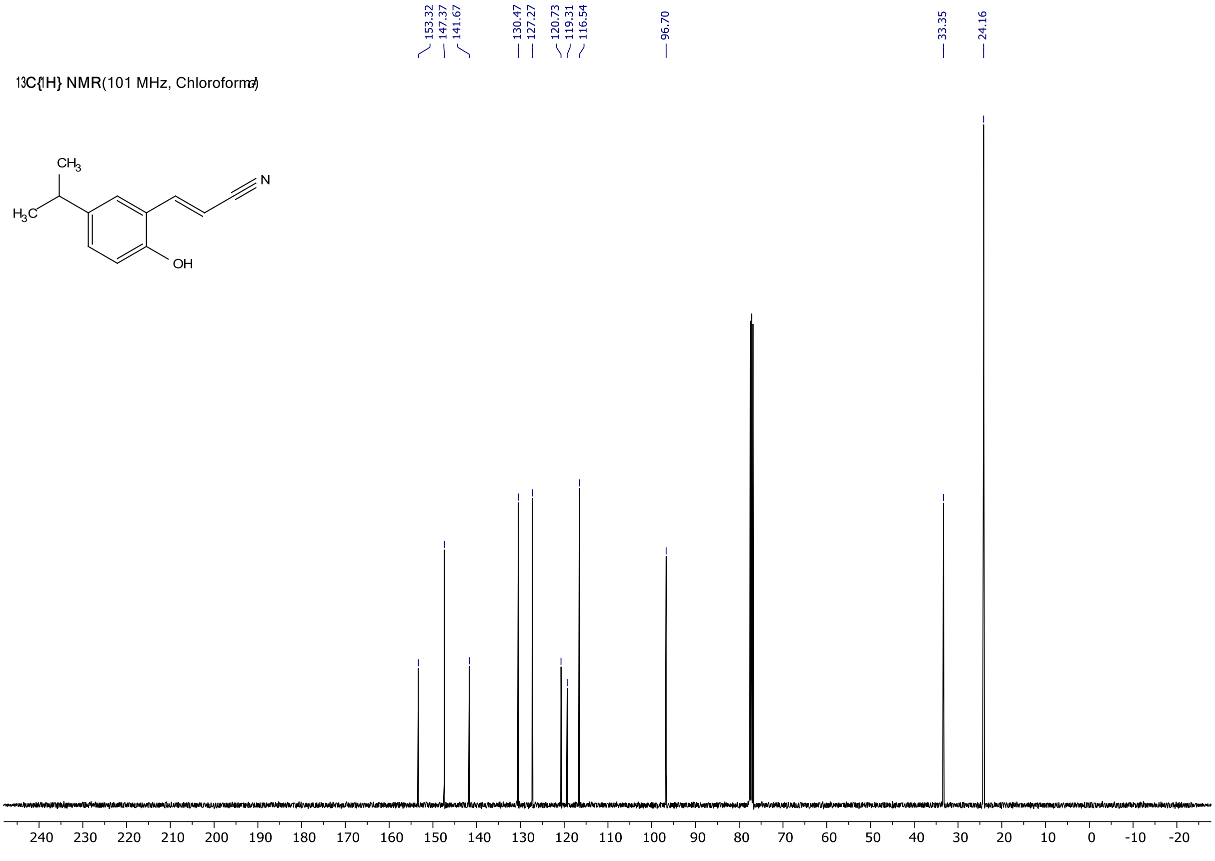

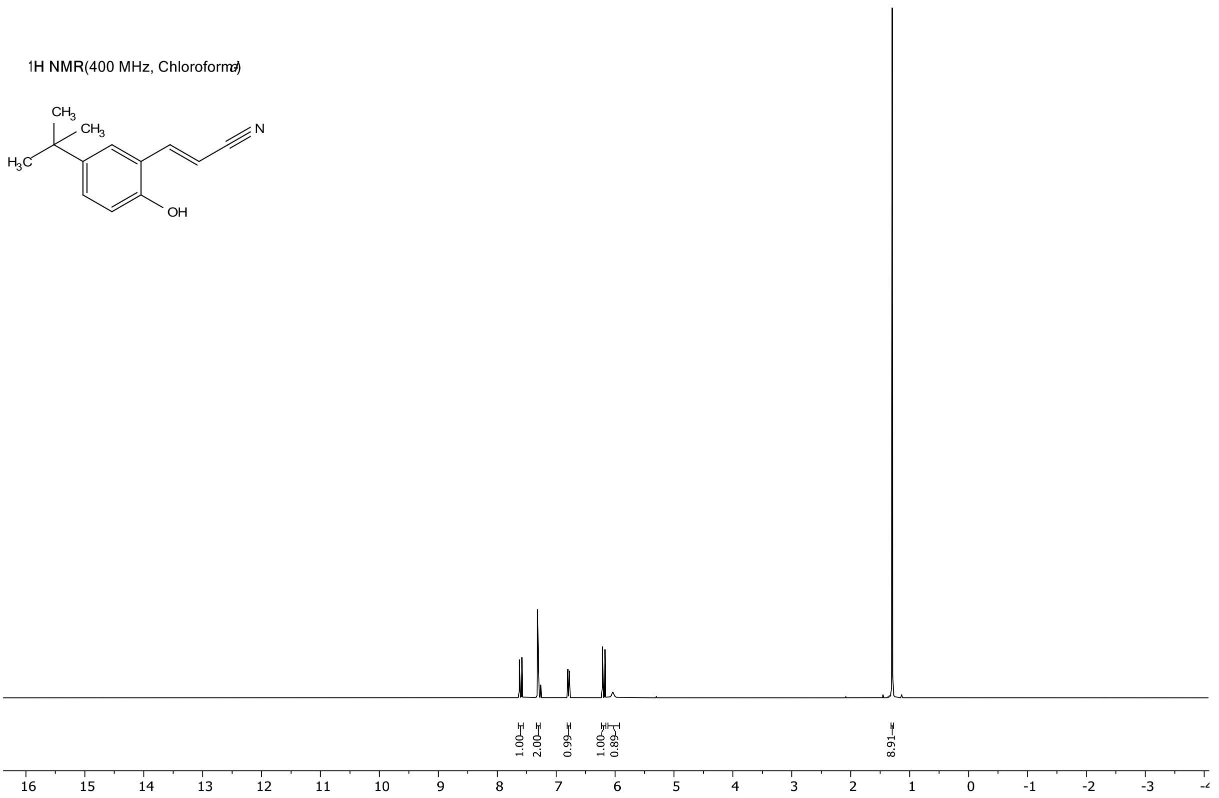
1H NMR(400 MHz, Chloroform-d)
CH3
CH3
H3C
N
OH
1.00
2.00
0.99
1.00
0.89
8.91
16 15 14 13 12 11 10 9 8 7 6 5 4 3 2 1 0 -1 -2 -3

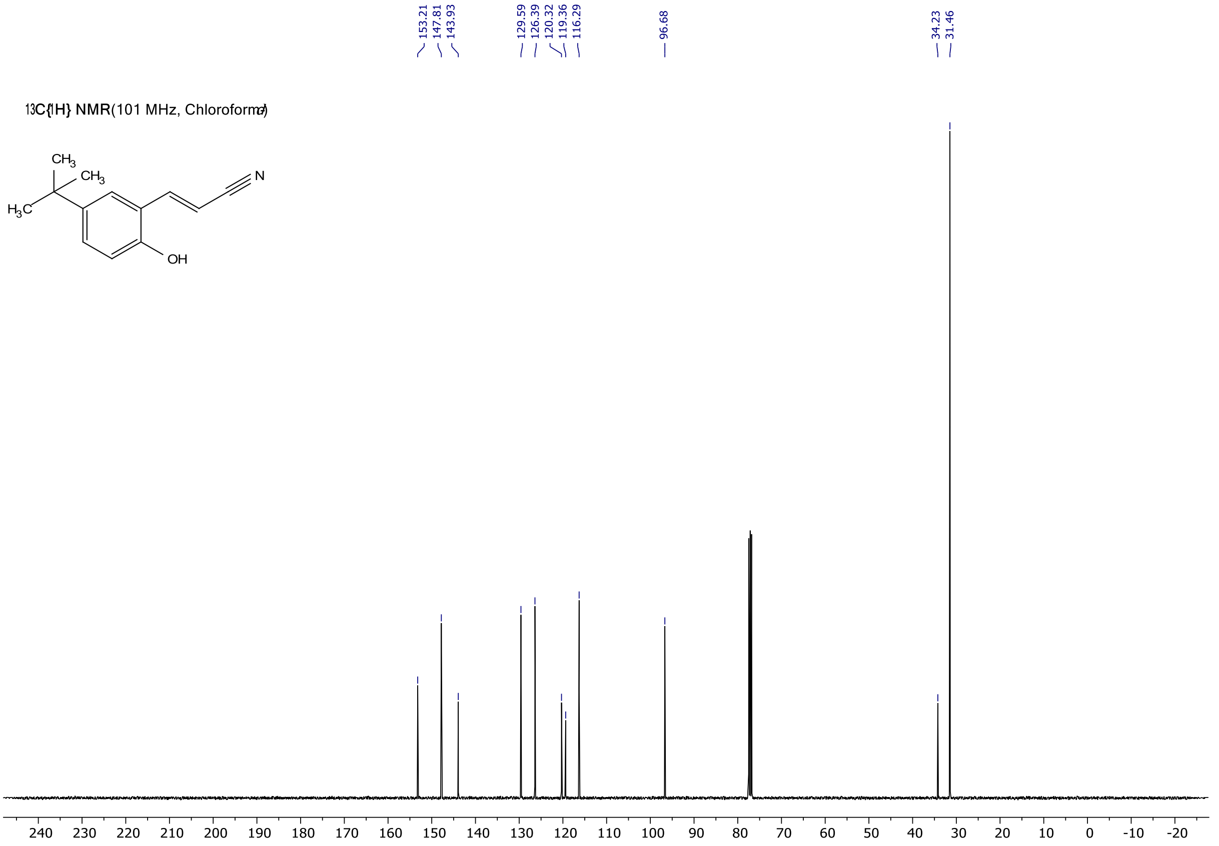
153.21
147.81
143.93
129.59
126.39
120.32
119.36
116.29
96.68
34.23
31.46
13C{1H} NMR(101 MHz, Chloroform-d)
CH3
CH3
H3C
N
OH
240 230 220 210 200 190 180 170 160 150 140 130 120 110 100 90 80 70 60 50 40 30 20 10 0 -10 -20

1H NMR(500 MHz, 363 K, Dimethylsulfoxide-*d6*)

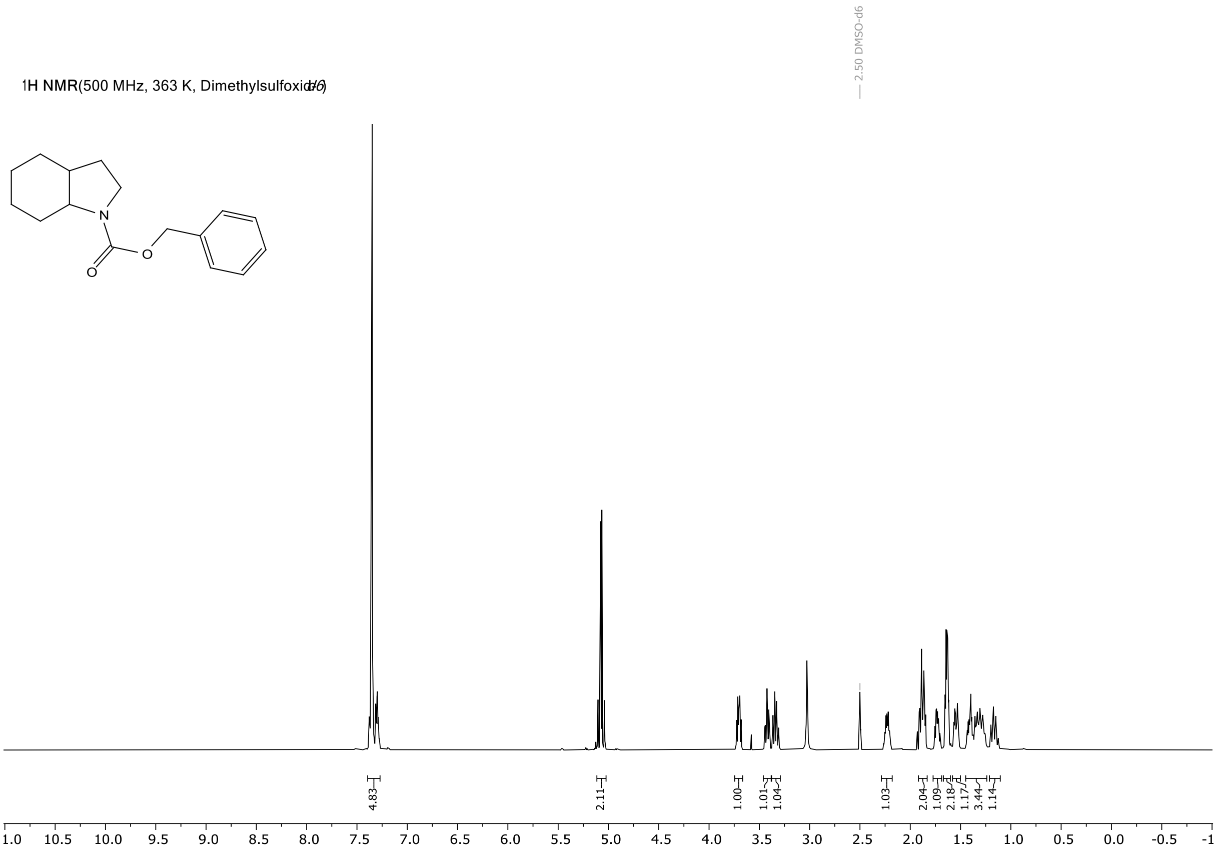

13C{1H} NMR(121 MHz, 363 K, Dimethylsulfoxide-*d6*)

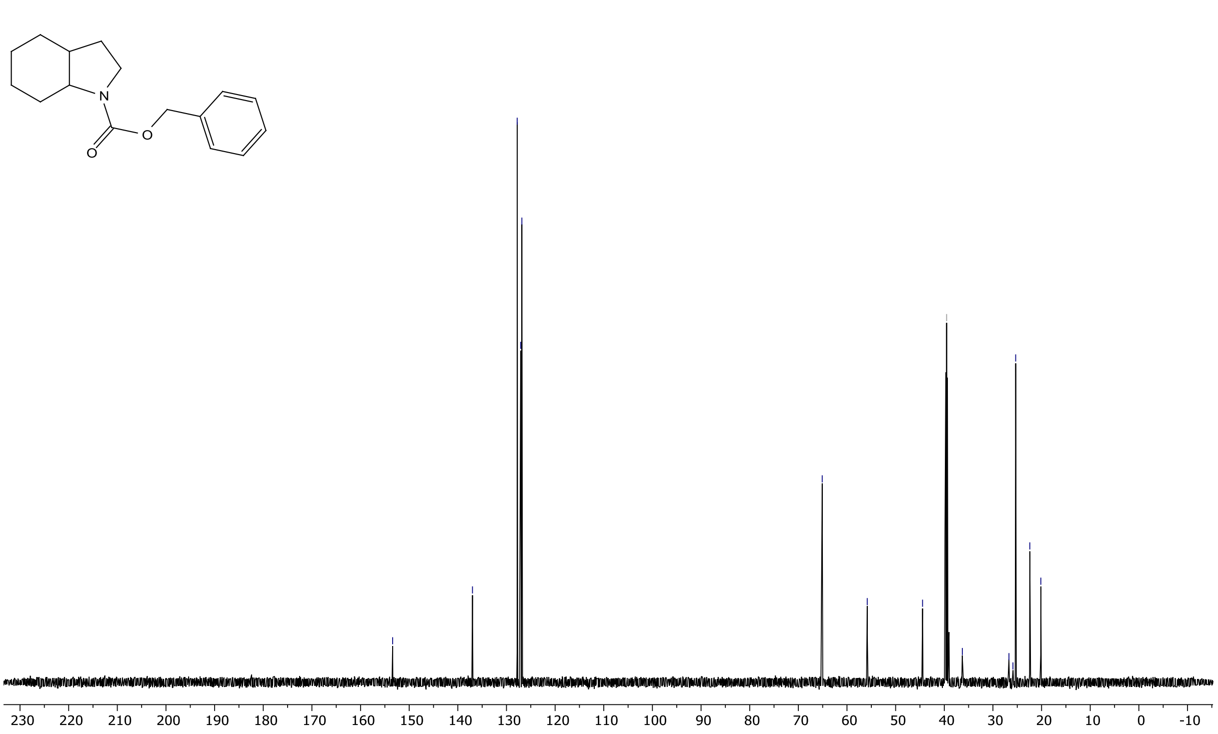

1H NMR(500 MHz, 363 K, Dimethylsulfoxide-d6)

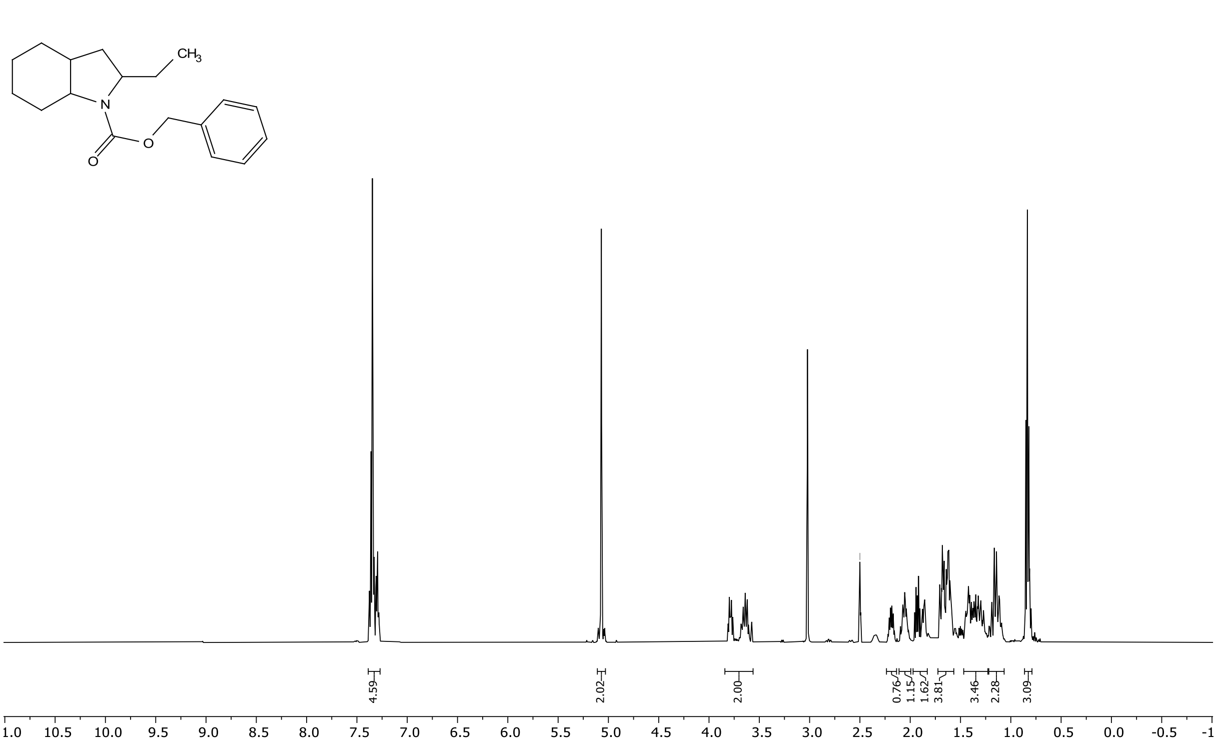

13C{1H} NMR(121 MHz, 363 K, Dimethylsulfoxide-d6)

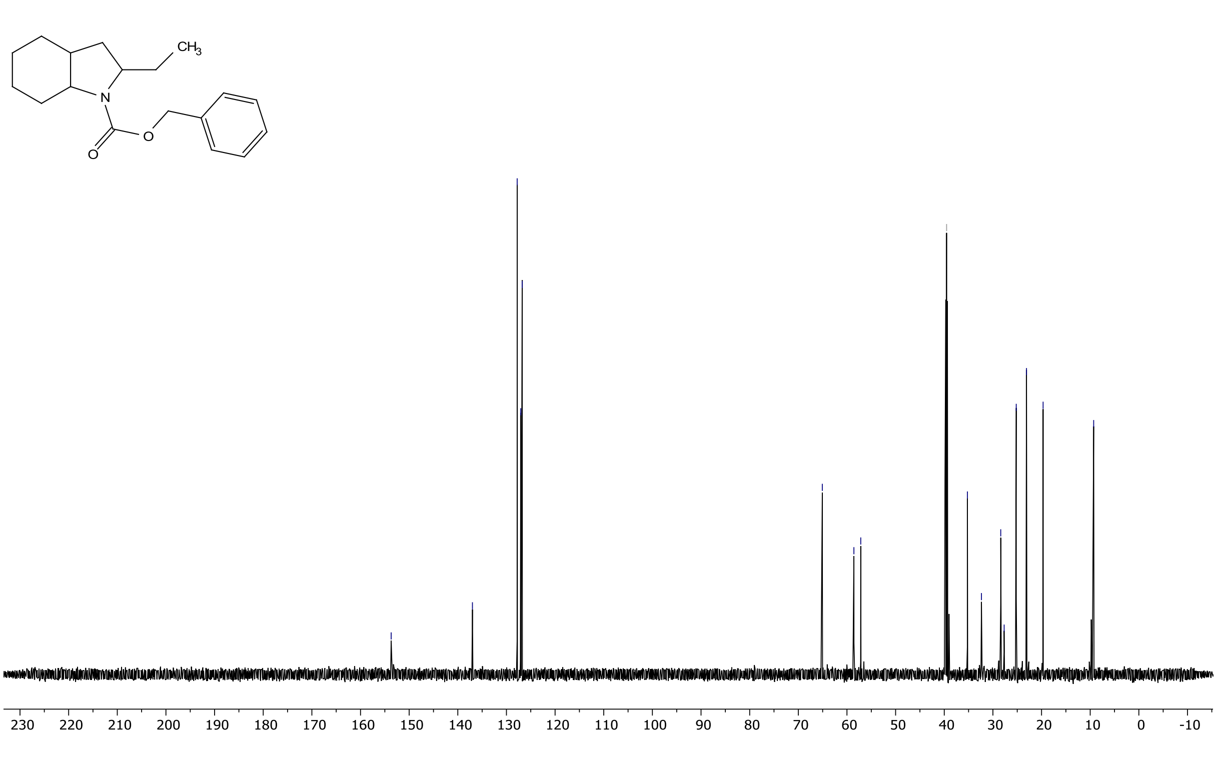

1H NMR(500 MHz, 363 K, Dimethylsulfoxide-d6)

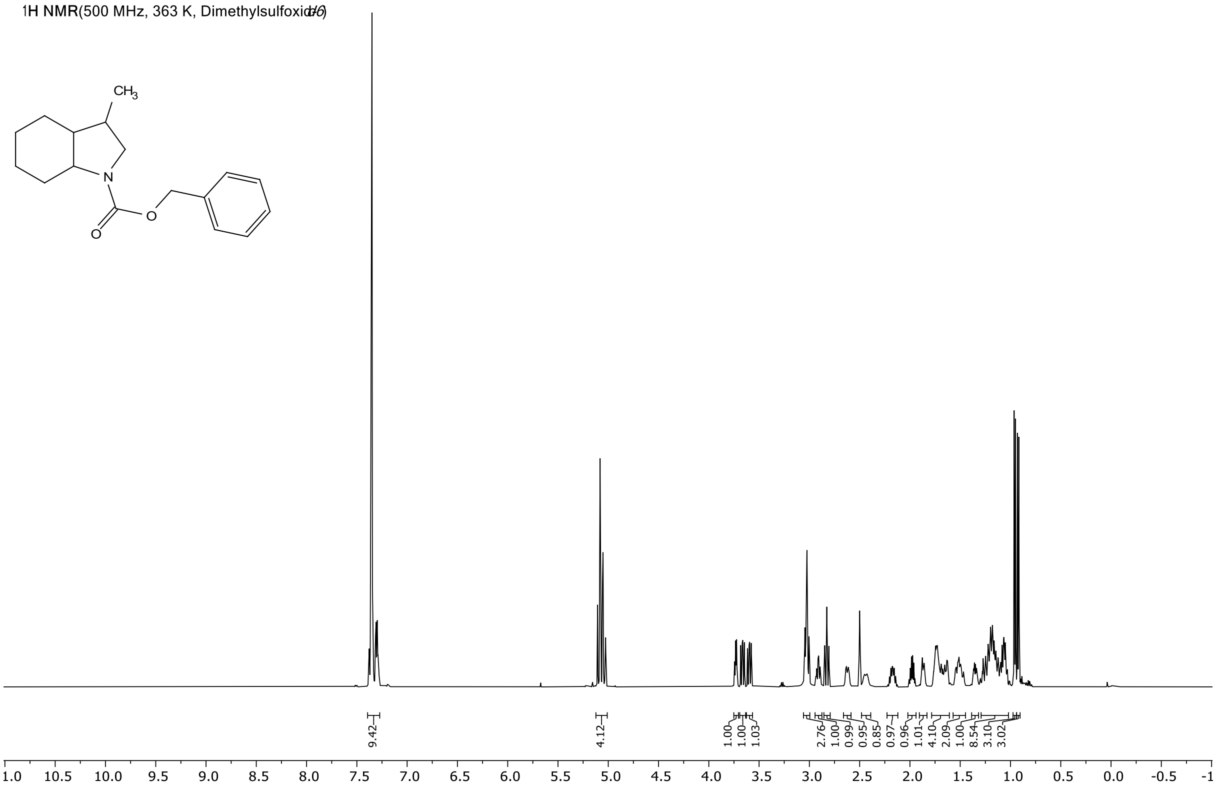

13C{1H} NMR(126 MHz, 363 K, Dimethylsulfoxide-d6)

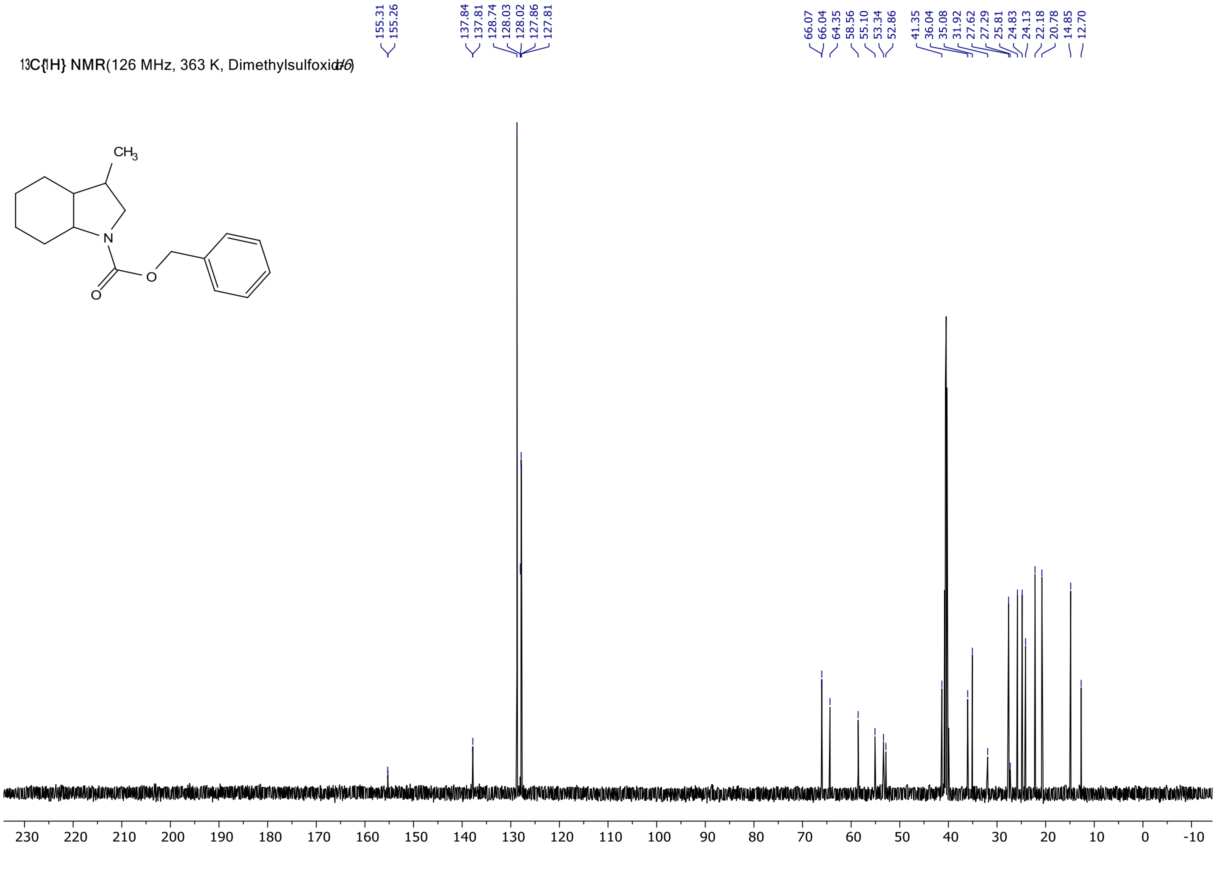

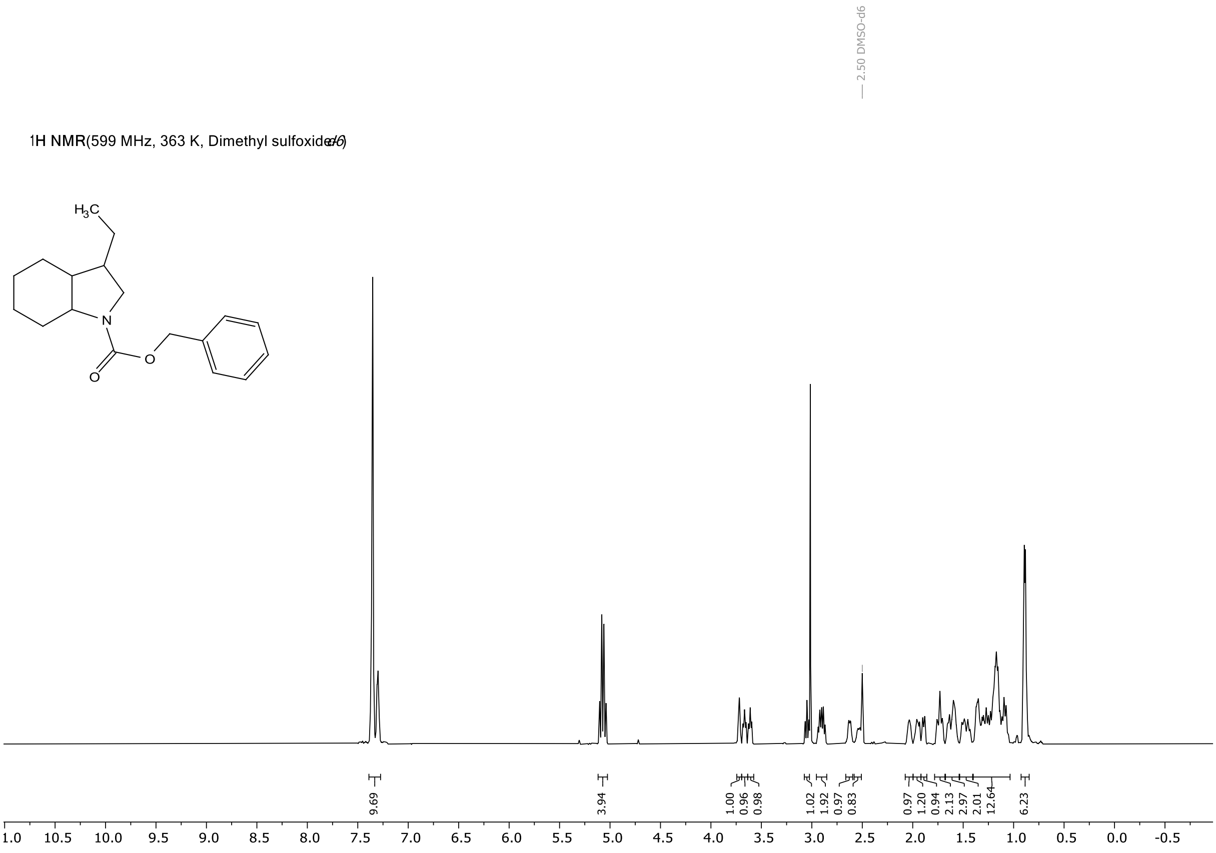
2.50 DMSO-d6
1H NMR(599 MHz, 363 K, Dimethyl sulfoxide-d6)
H3C
N
O
O
9.69
3.94
1.00
0.96
0.98
1.02
1.92
0.97
0.83
0.97
1.20
0.94
2.13
2.97
2.01
12.64
6.23
1.0 10.5 10.0 9.5 9.0 8.5 8.0 7.5 7.0 6.5 6.0 5.5 5.0 4.5 4.0 3.5 3.0 2.5 2.0 1.5 1.0 0.5 0.0 -0.5

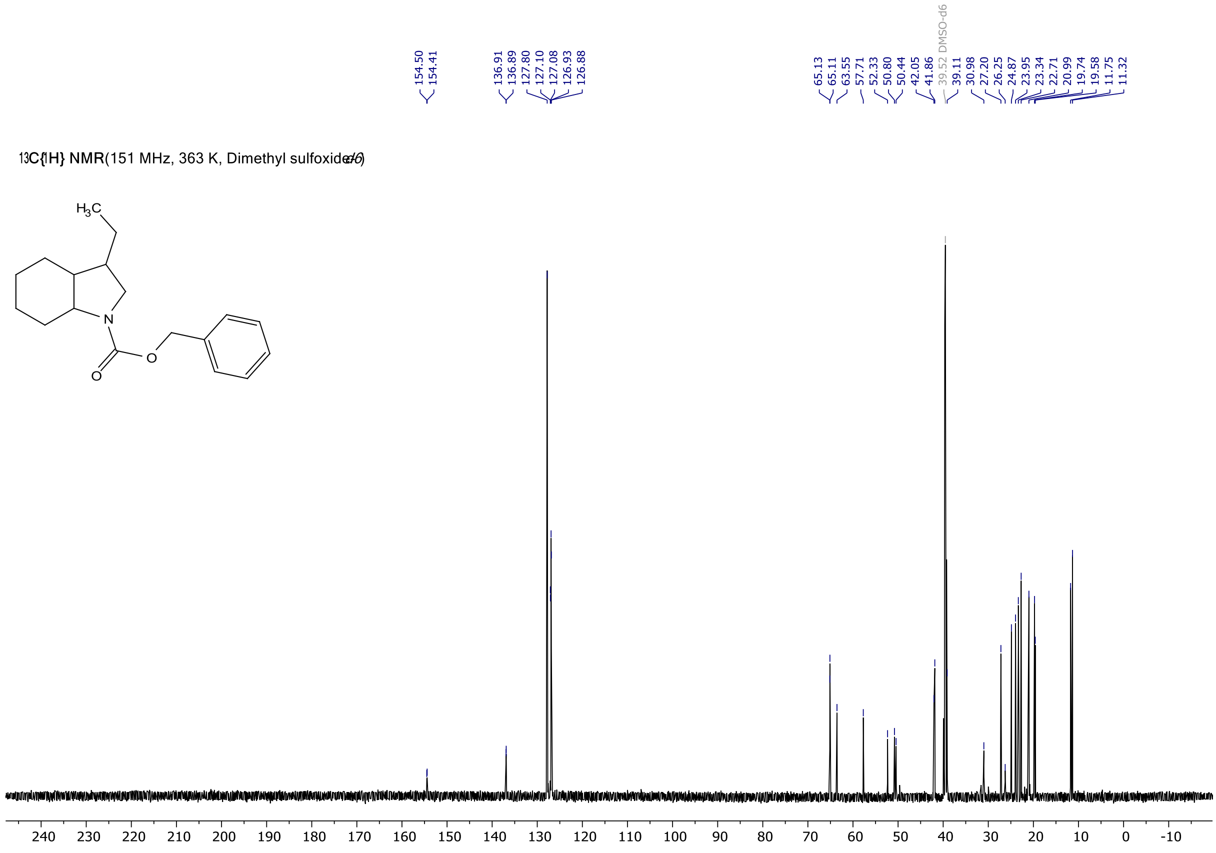
154.50
154.41
136.91
136.89
127.80
127.10
127.08
126.93
126.88
65.13
65.11
63.55
57.71
52.33
50.80
50.44
42.05
41.86
39.52 DMSO-d6
39.11
30.98
27.20
26.25
24.87
23.95
23.34
22.71
20.99
19.74
19.58
11.75
11.32
13C{1H} NMR(151 MHz, 363 K, Dimethyl sulfoxide-d6)
H3C
N
O
O
240 230 220 210 200 190 180 170 160 150 140 130 120 110 100 90 80 70 60 50 40 30 20 10 0 -10

1H NMR(500 MHz, 363 K, Dimethylsulfoxide-d6)

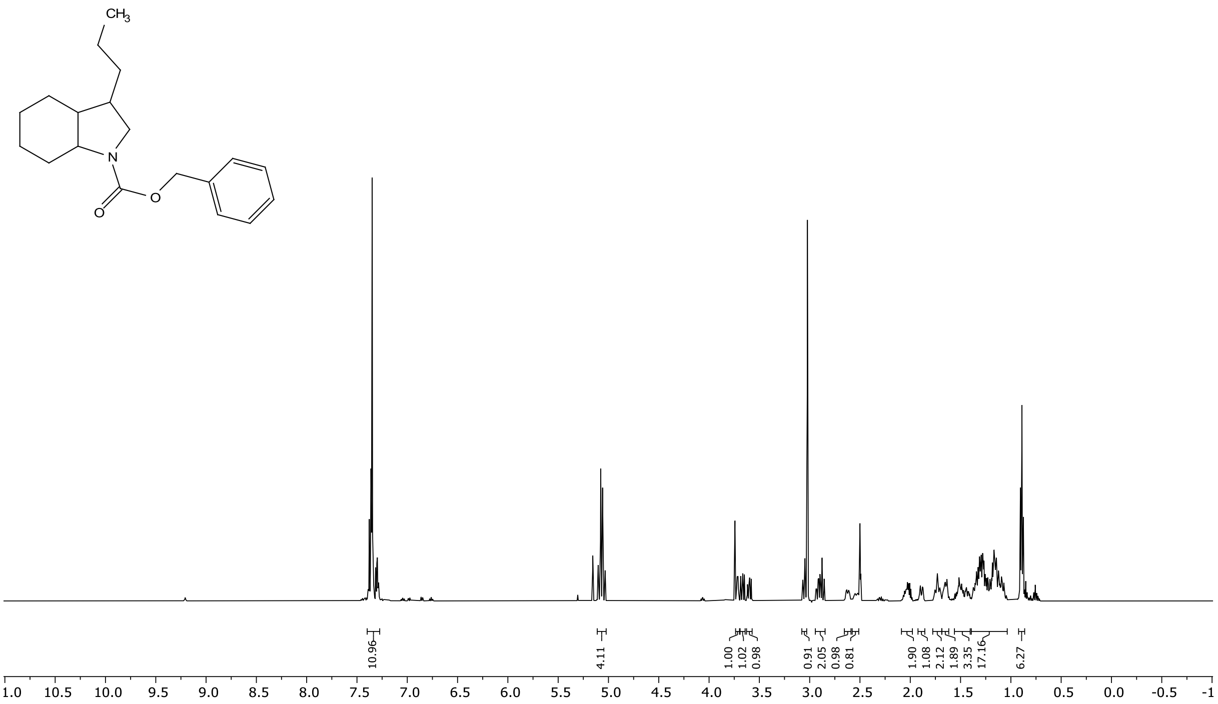

13C{1H} NMR(126 MHz, 363 K, Dimethylsulfoxide-d6)

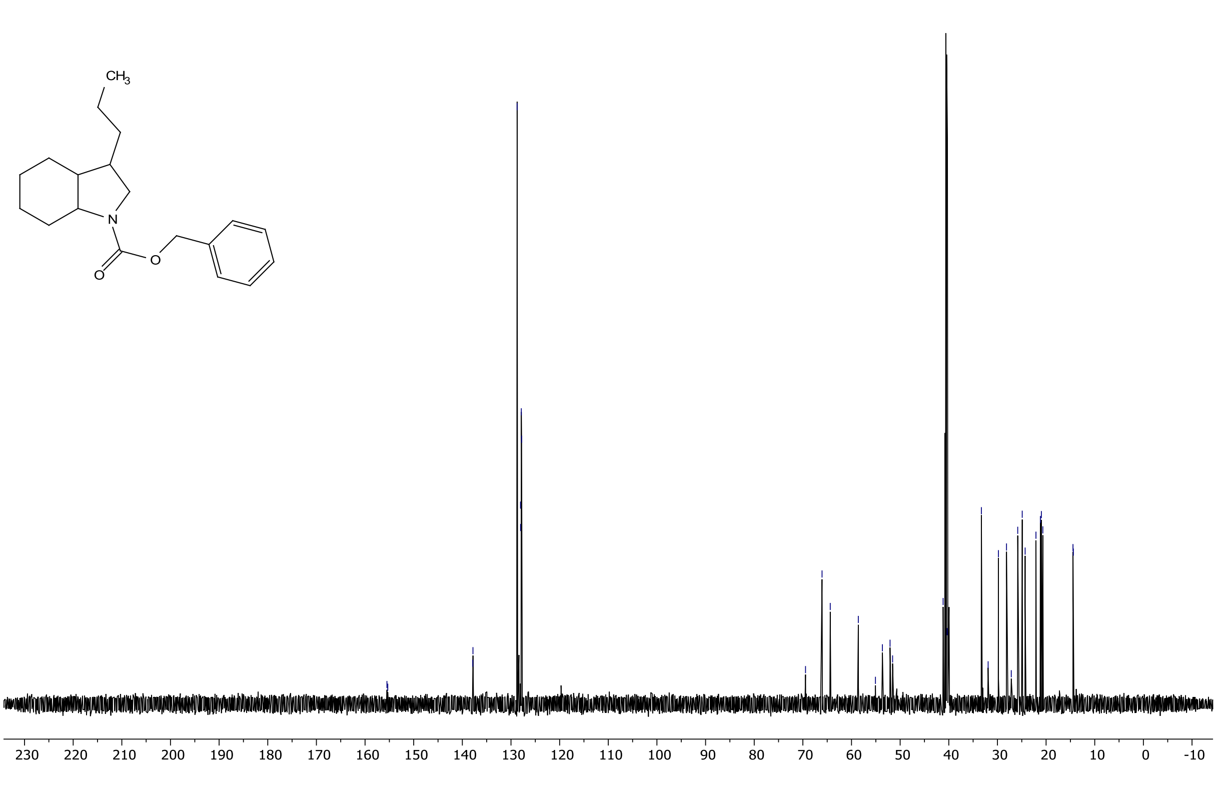

1H NMR(599 MHz, 363 K, Dimethyl sulfoxide-d6)

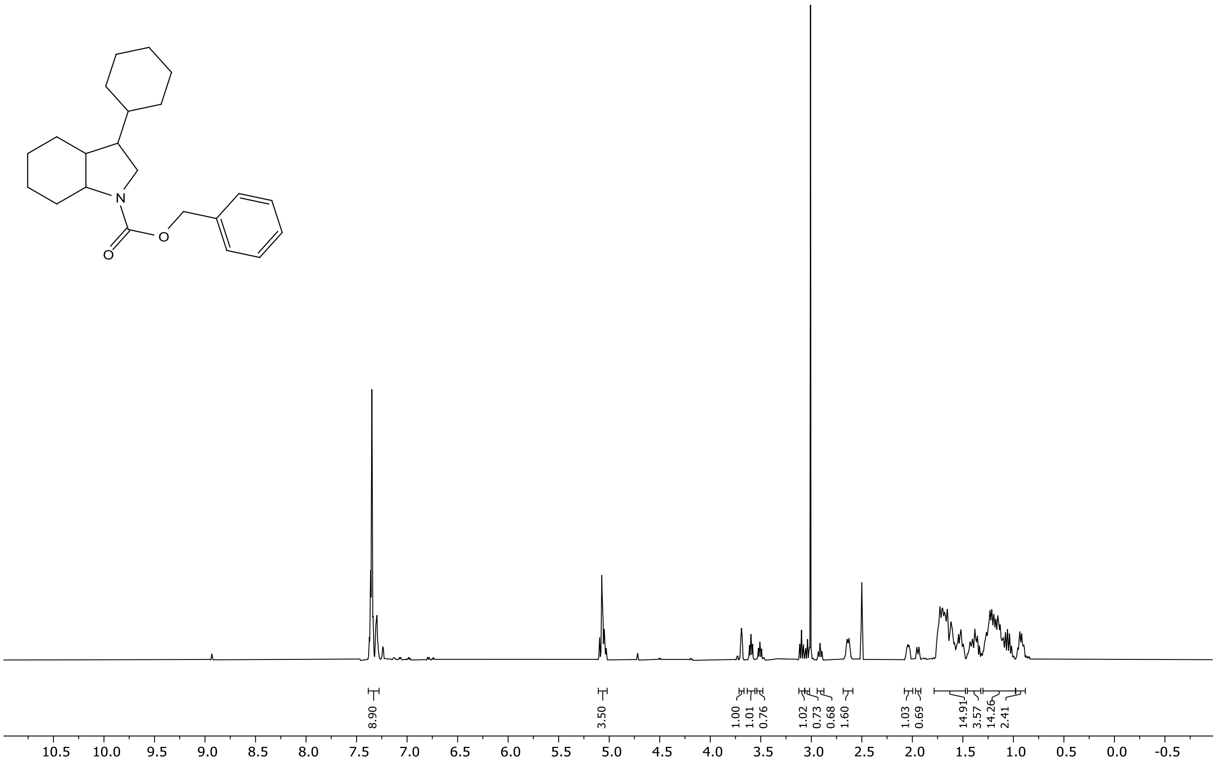

13C{1H} NMR(151 MHz, 363 K, Dimethyl sulfoxide-d6)

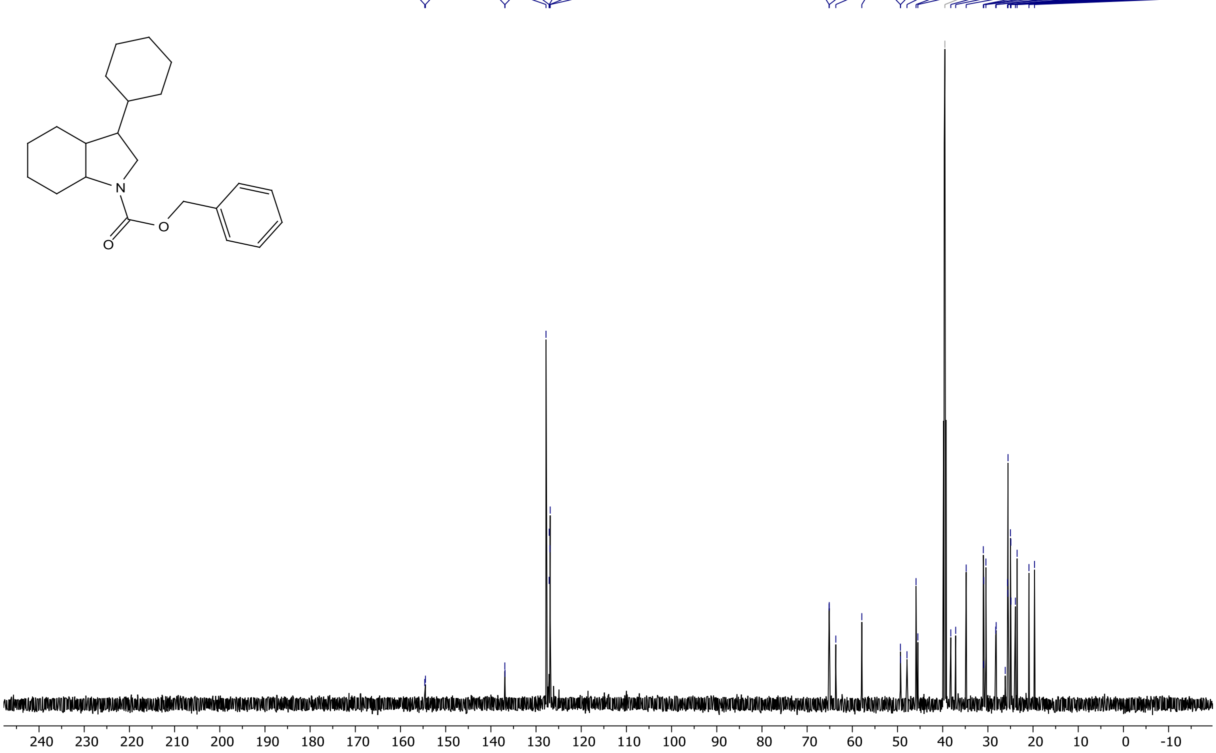

1H NMR(500 MHz, 363 K, Dimethyl sulfoxide-d6)

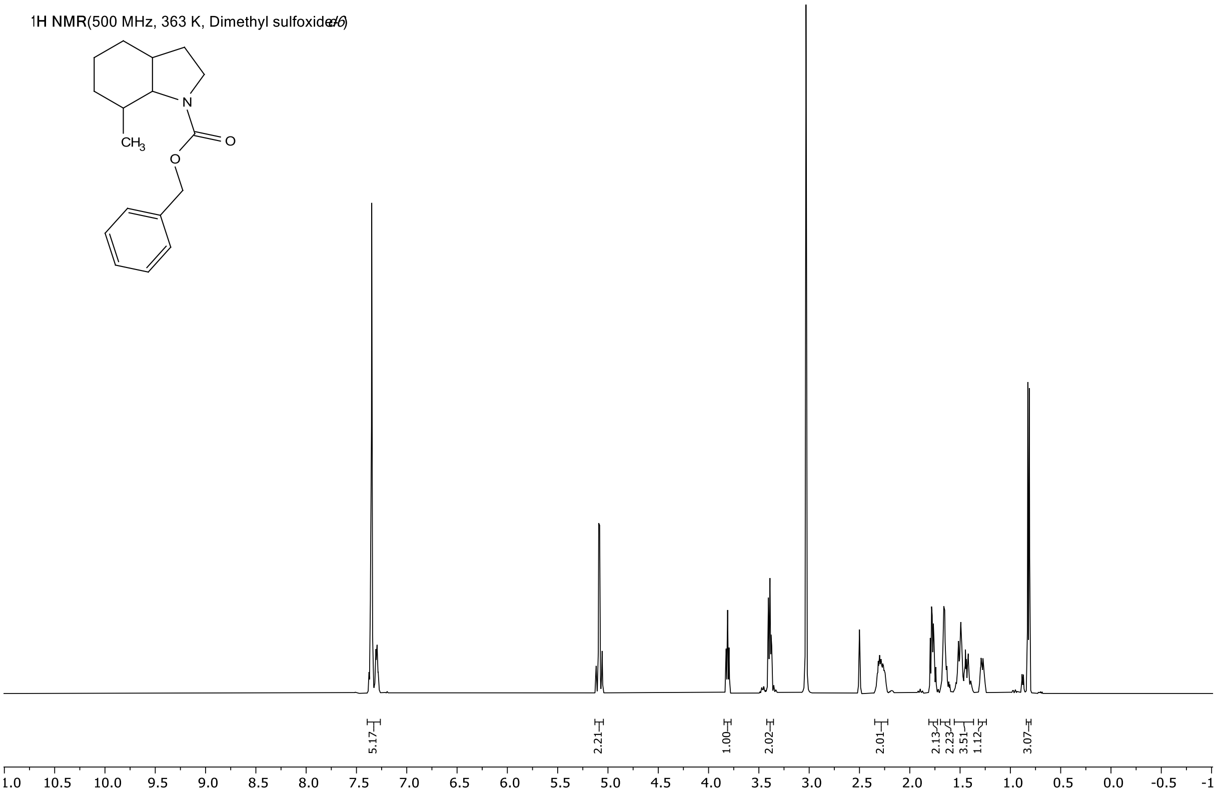

13C{1H} NMR(126 MHz, 363 K, Dimethyl sulfoxide-d6)

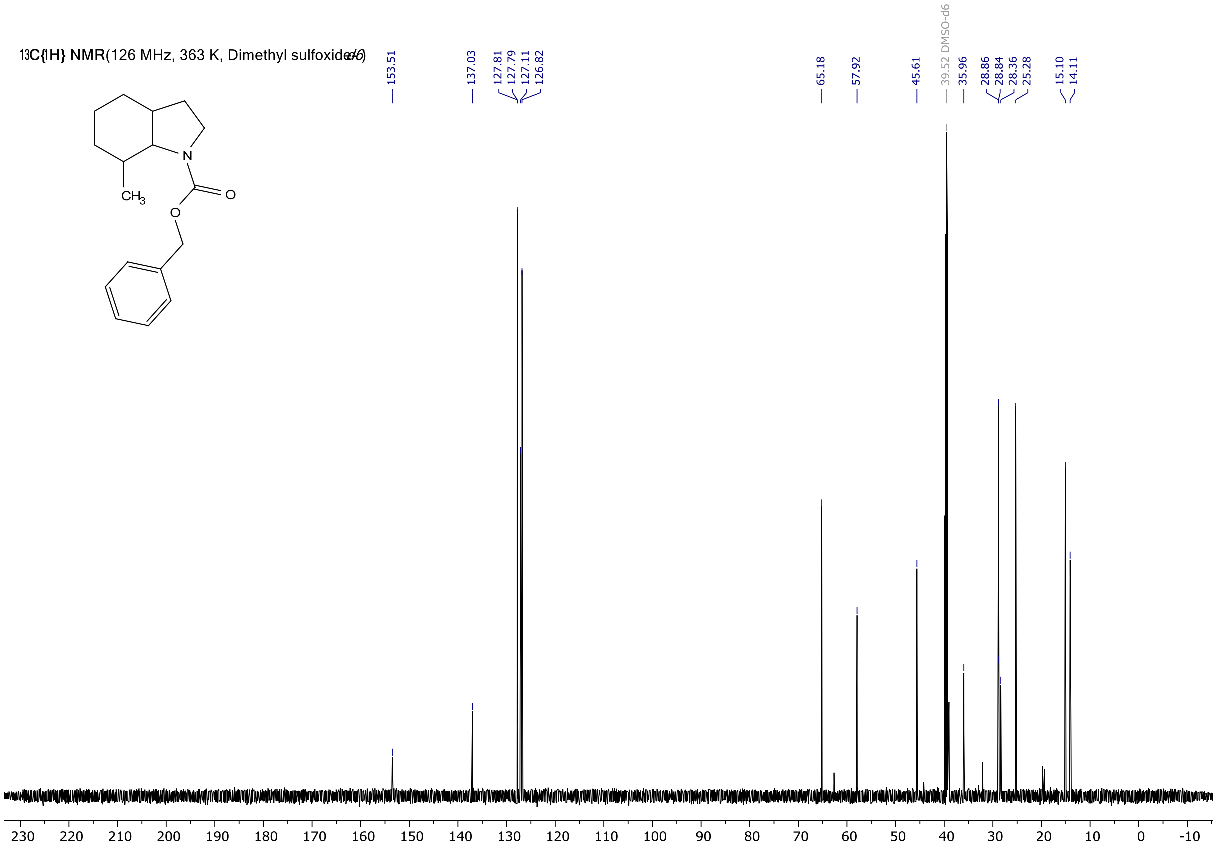

1H NMR(500 MHz, 363 K, Dimethylsulfoxide-d6)

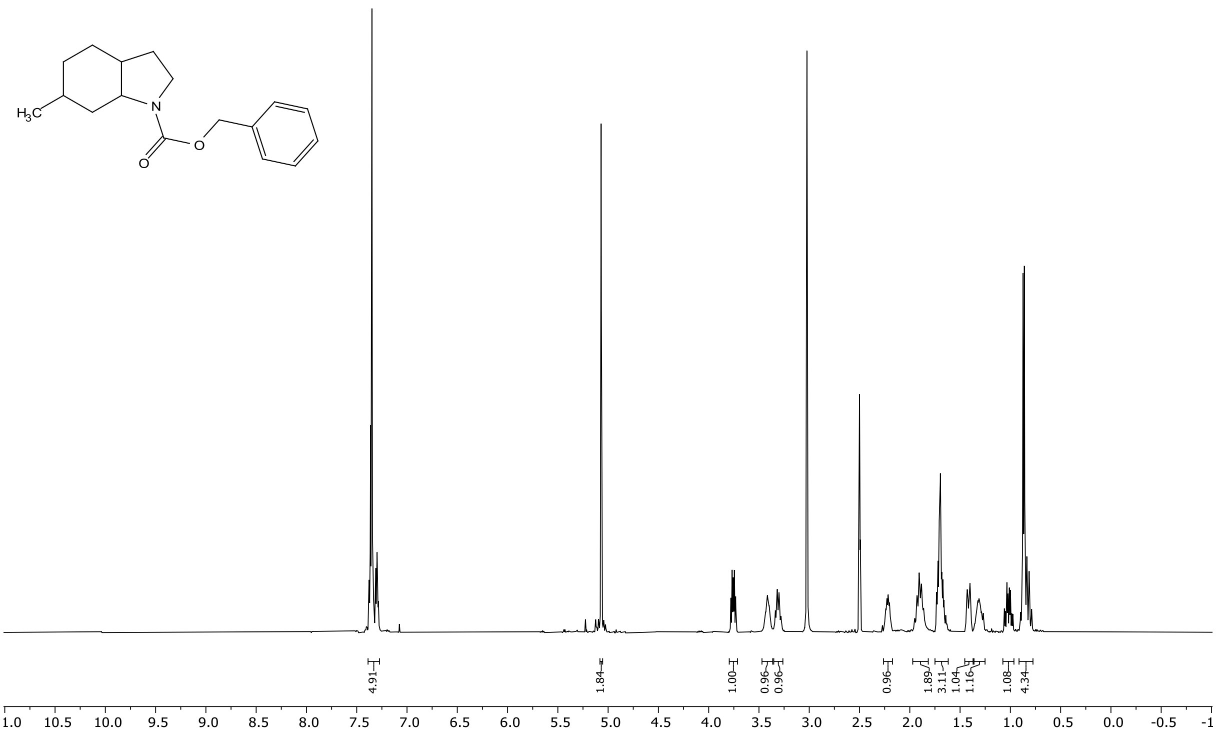

13C{1H} NMR(126 MHz, 363 K, Dimethylsulfoxide-d6)

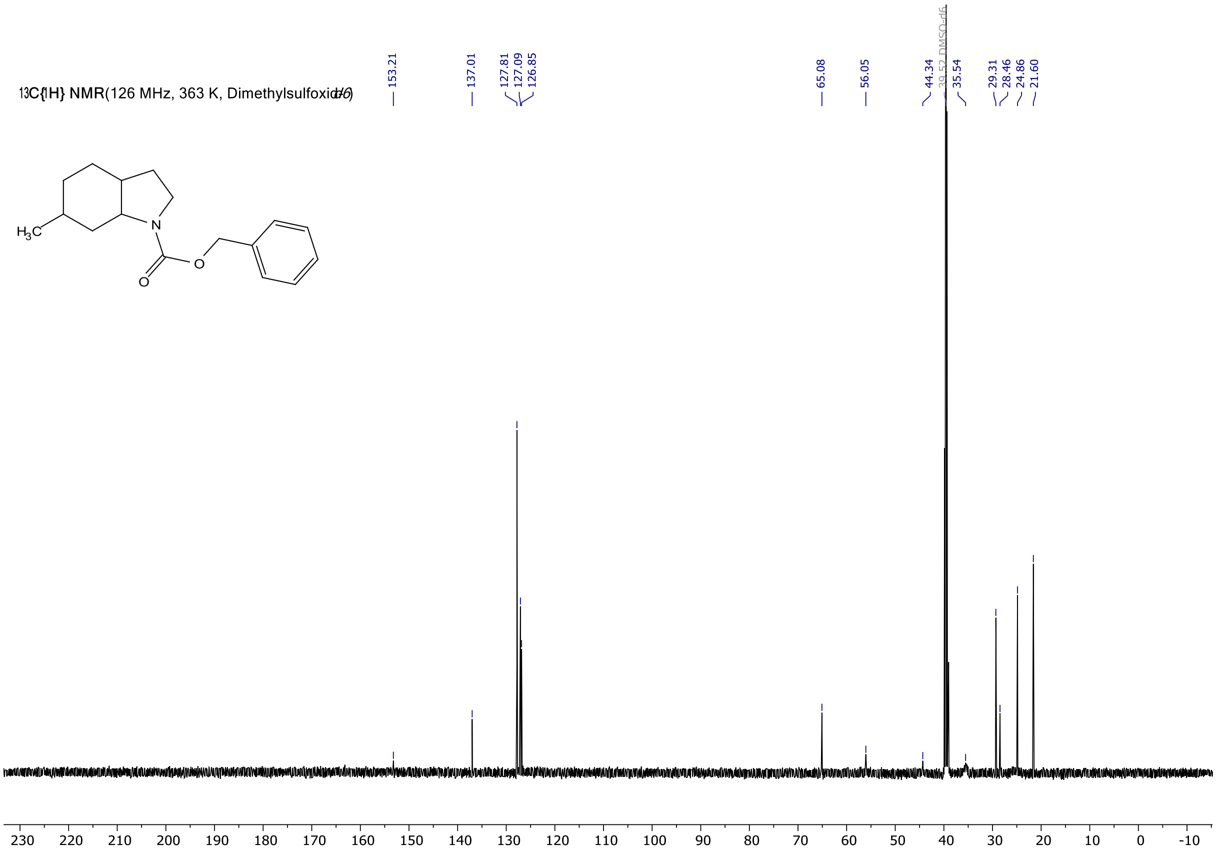

1H NMR(500 MHz, 363 K, Dimethylsulfoxide-d6)

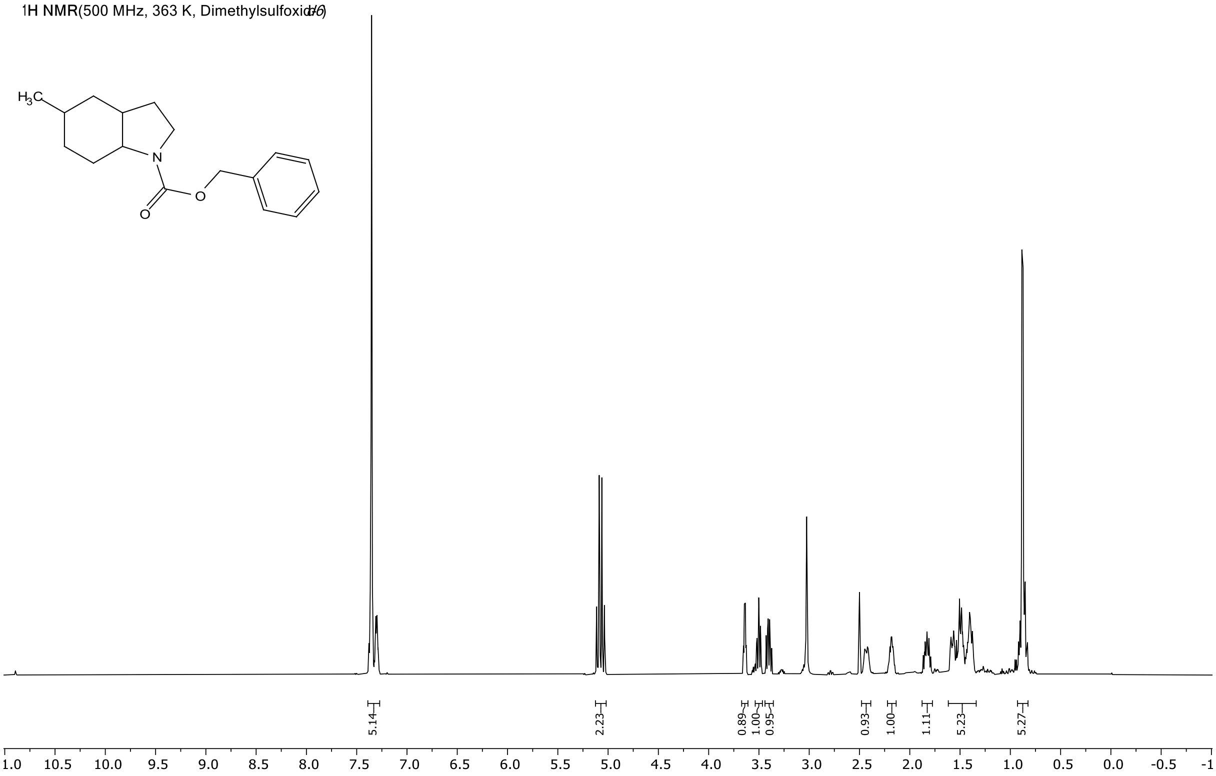

13C{1H} NMR(121 MHz, 363 K, Dimethylsulfoxide-d6)

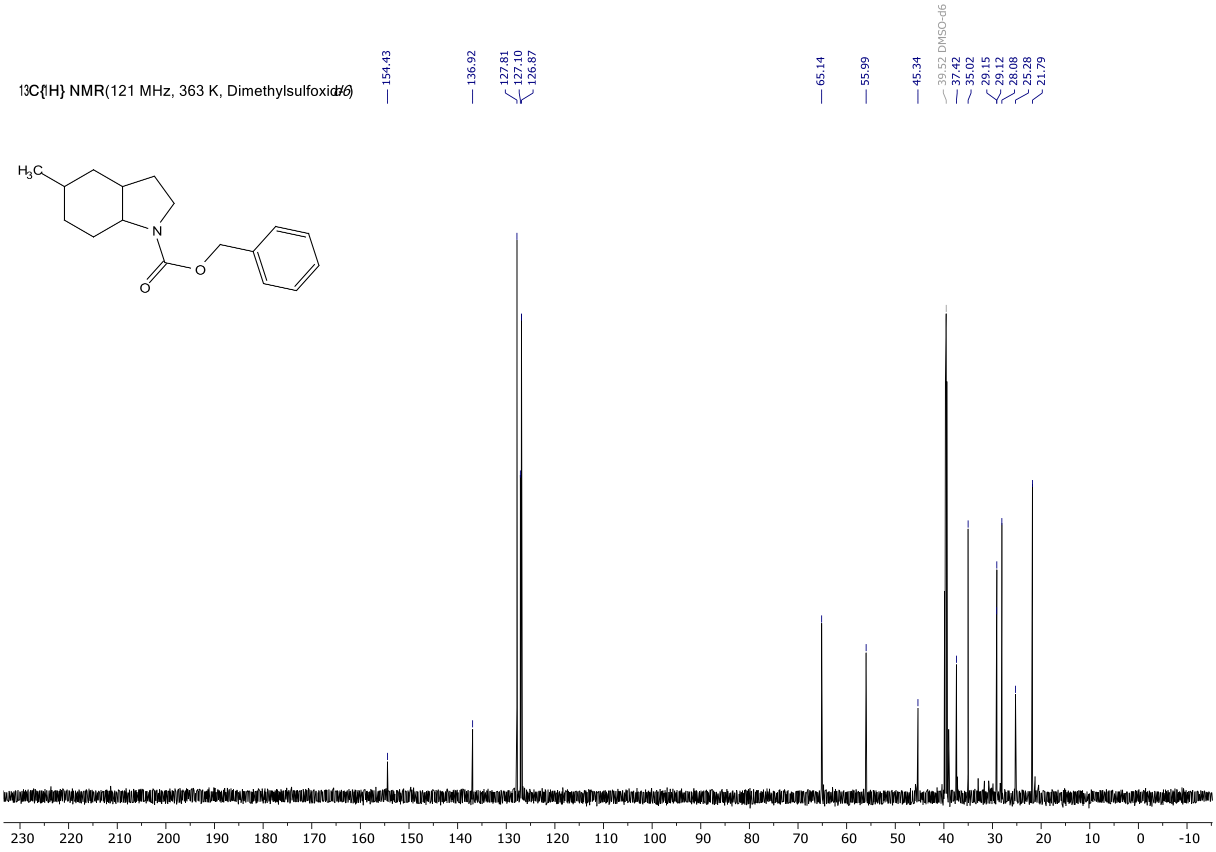

1H NMR(500 MHz, 363 K, Dimethylsulfoxide-d6)

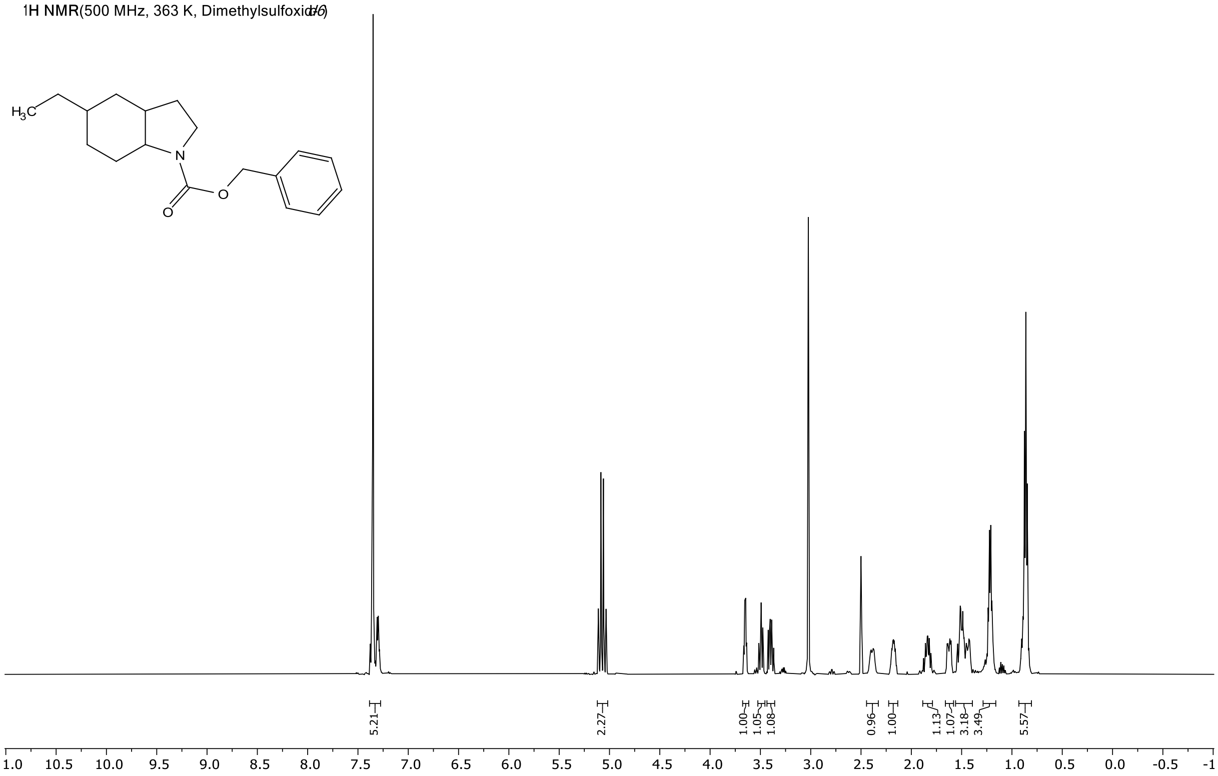

13C{1H} NMR(126 MHz, 363 K, Dimethylsulfoxide-d6)

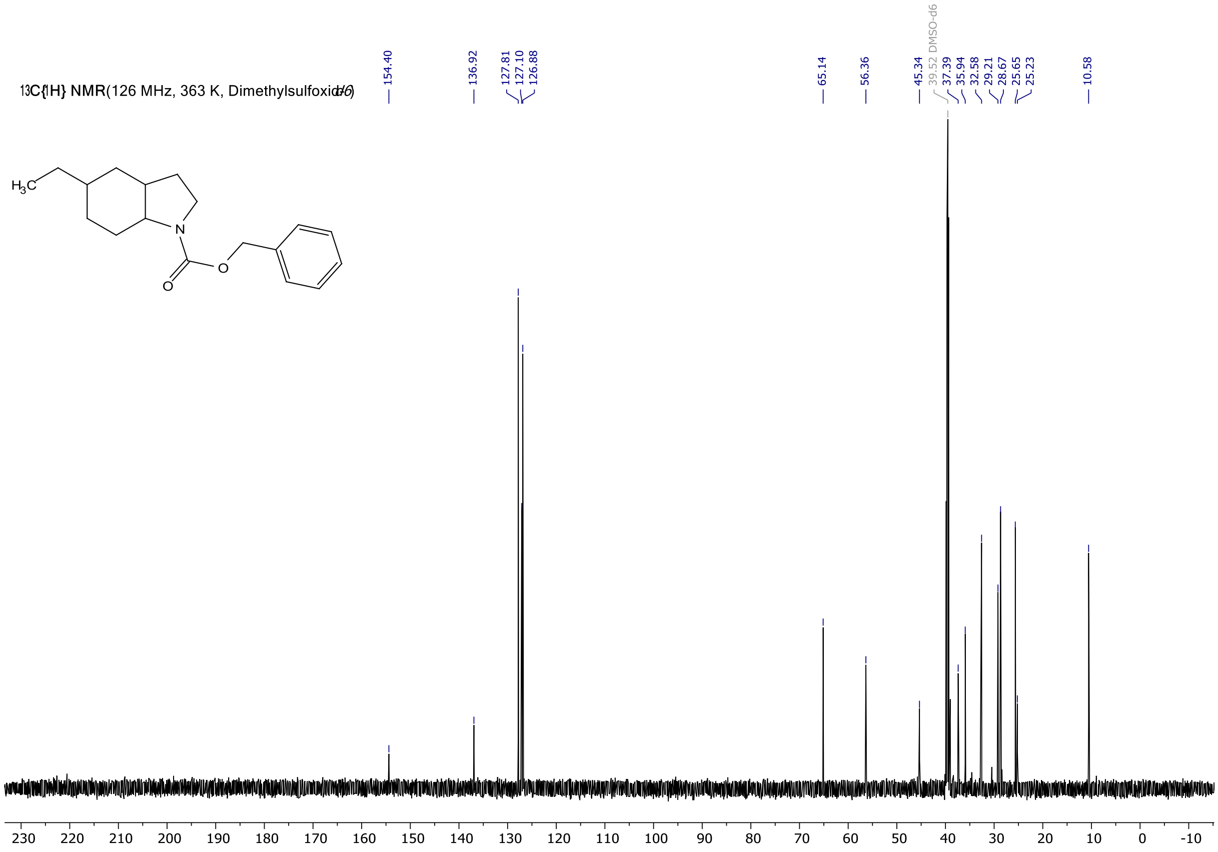

1H NMR(599 MHz, 363 K, Dimethylsulfoxide-d6)

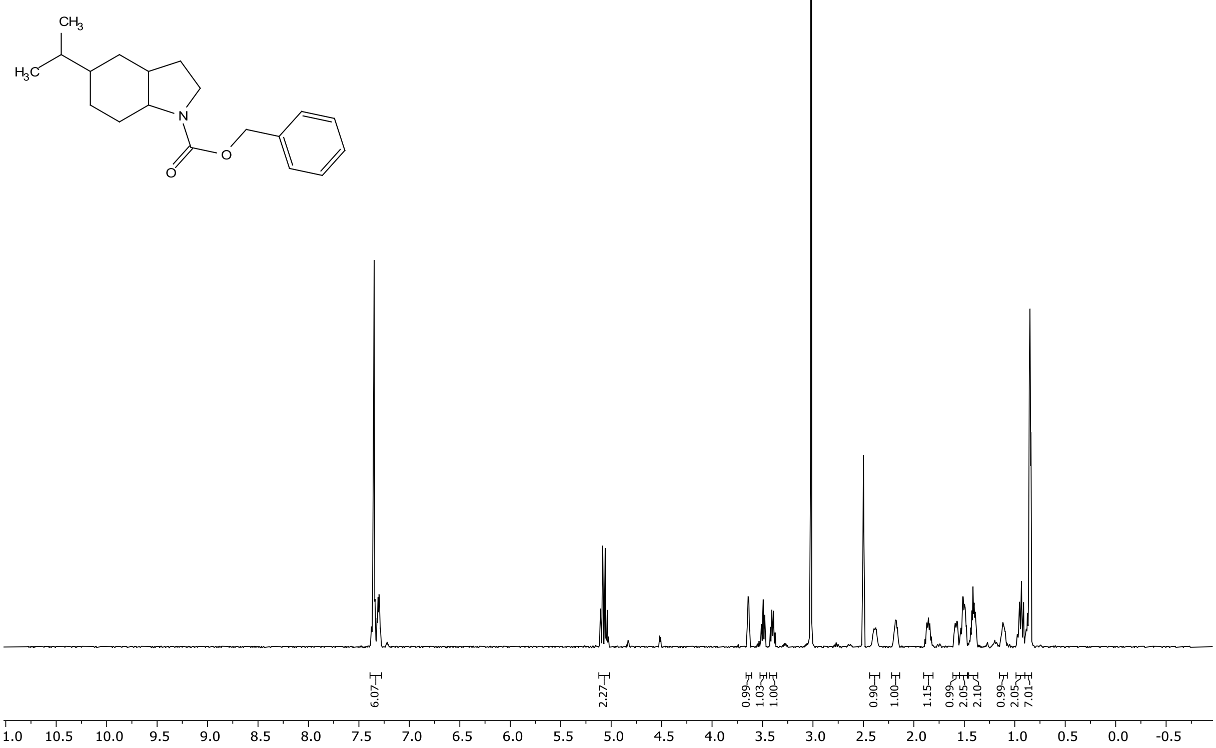

13C{1H} NMR(151 MHz, 363 K, Dimethylsulfoxide-d6)

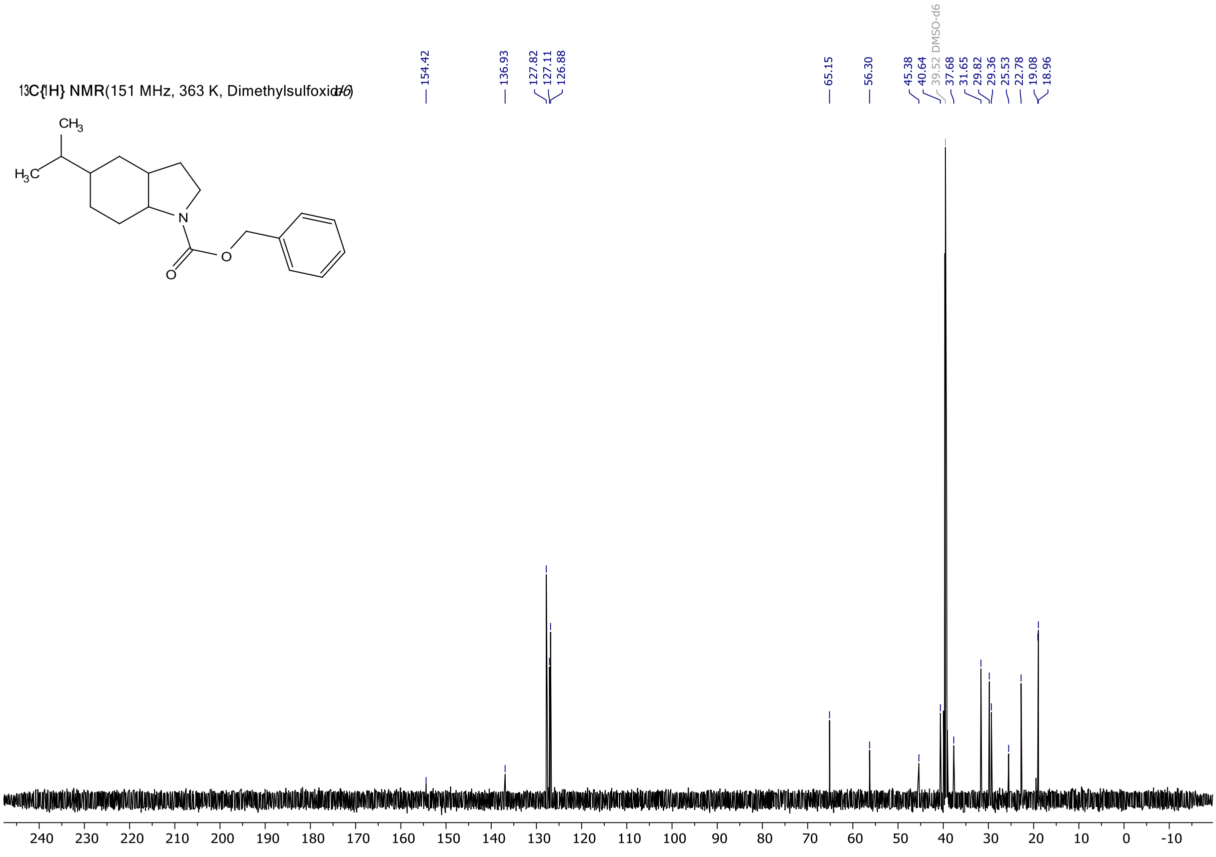

1H NMR(500 MHz,Dimethylsulfoxide-d6)

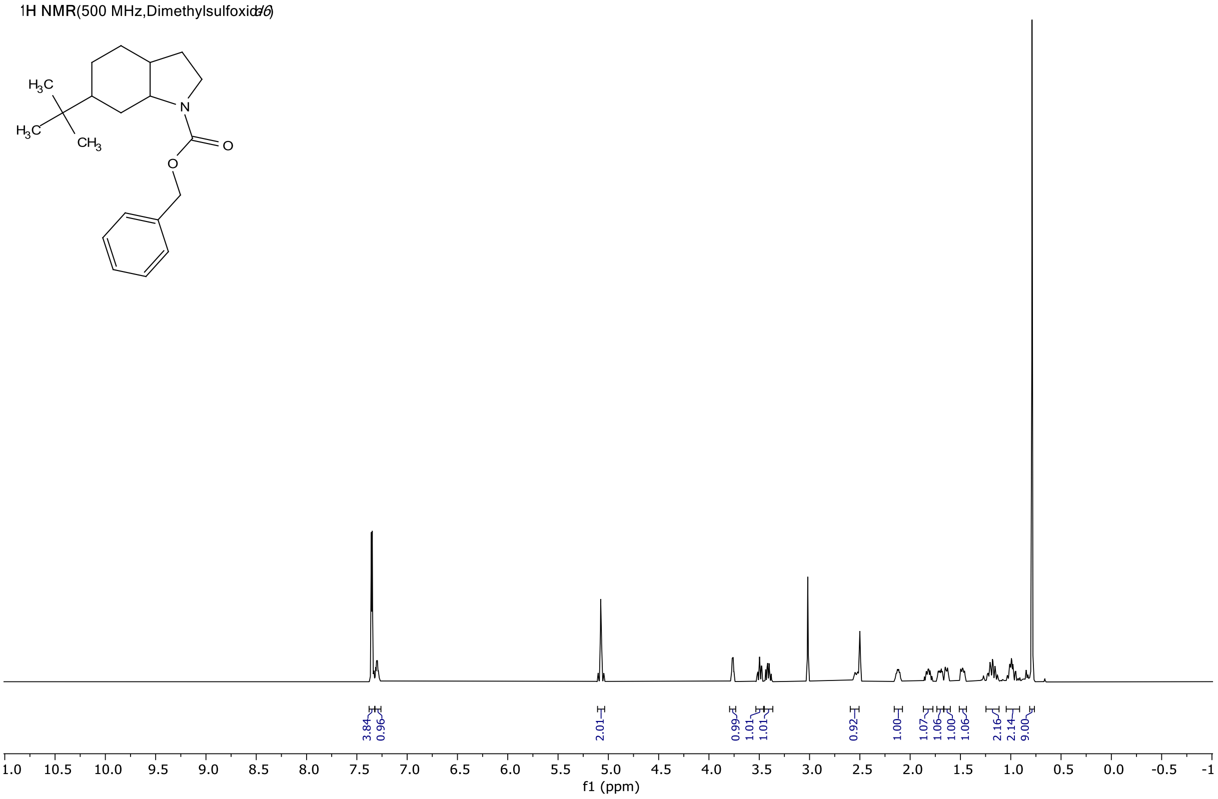

13C{1H} NMR(126 MHz,Dimethylsulfoxide-d6)

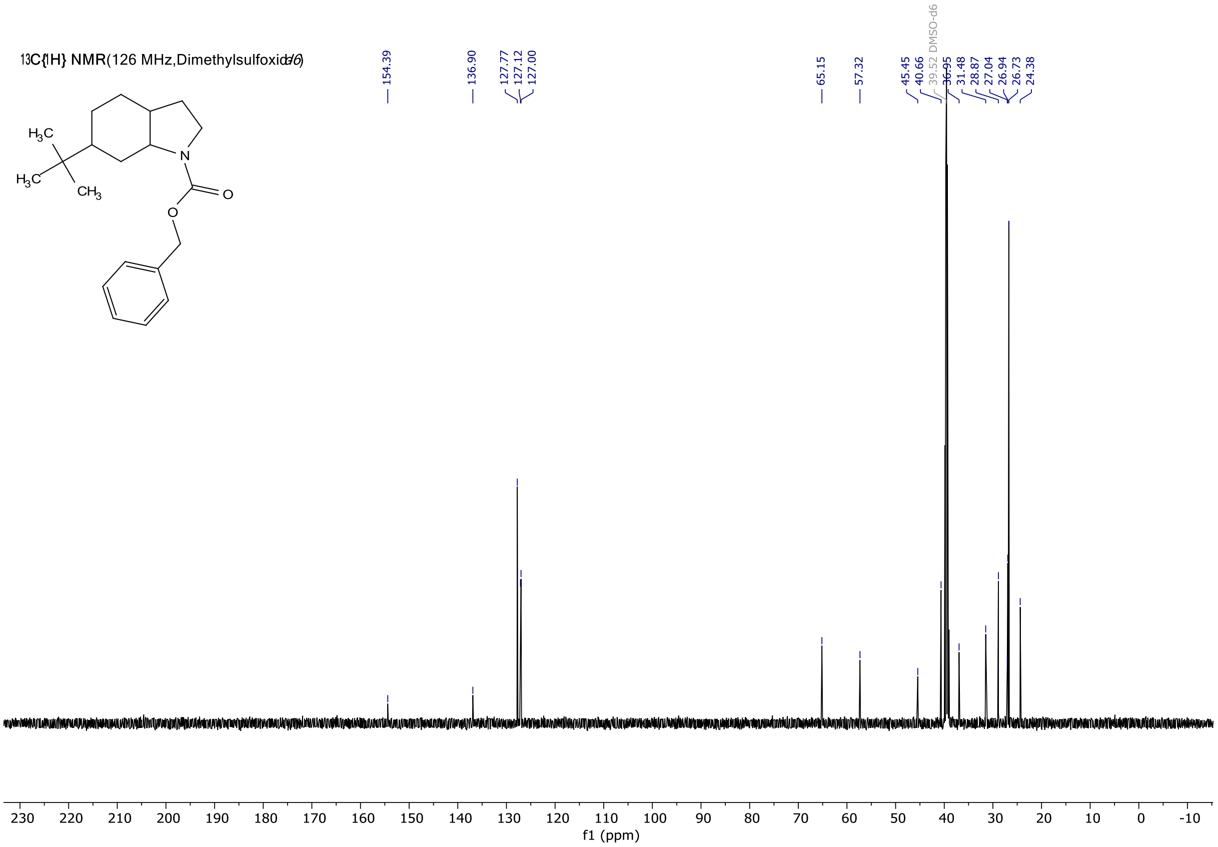

1H NMR(500 MHz, 363 K, Dimethylsulfoxide-d6)

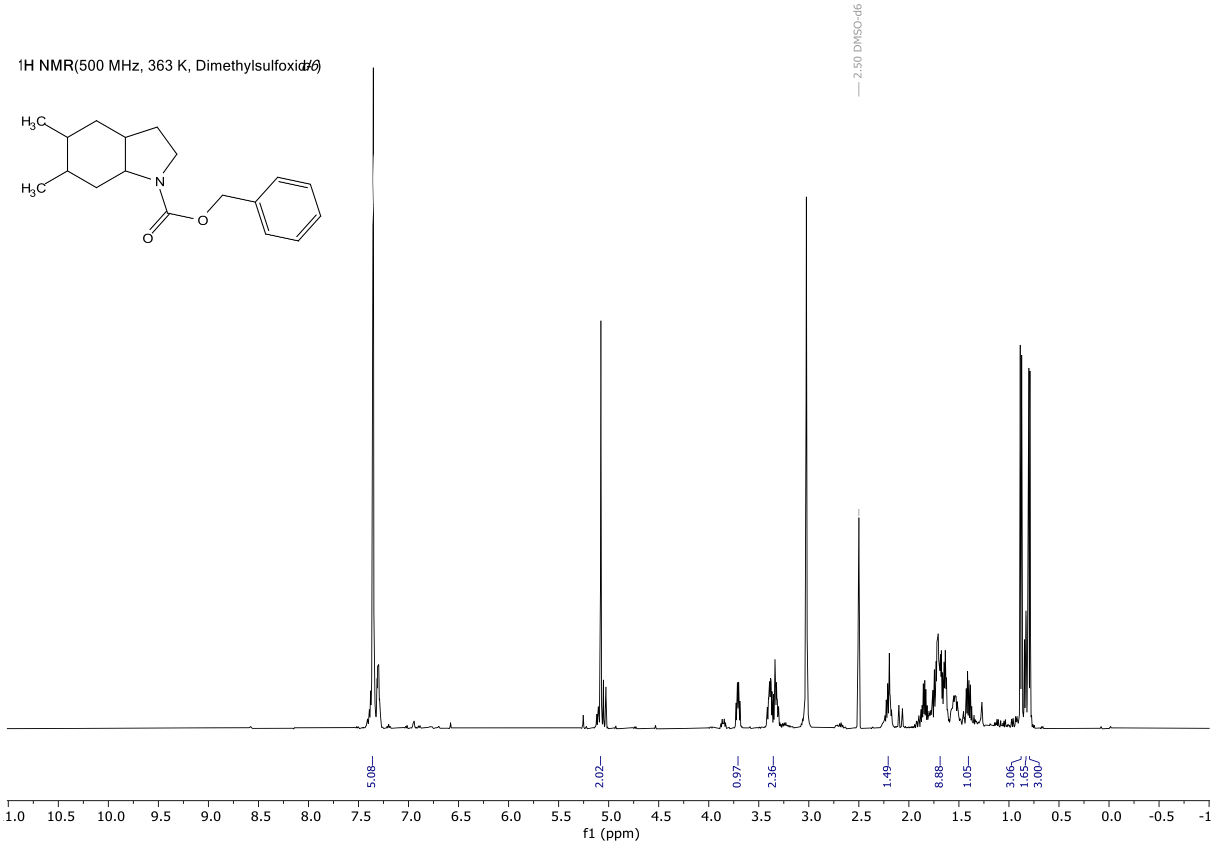

13C{1H} NMR(126 MHz, 363 K, Dimethylsulfoxide-d6)

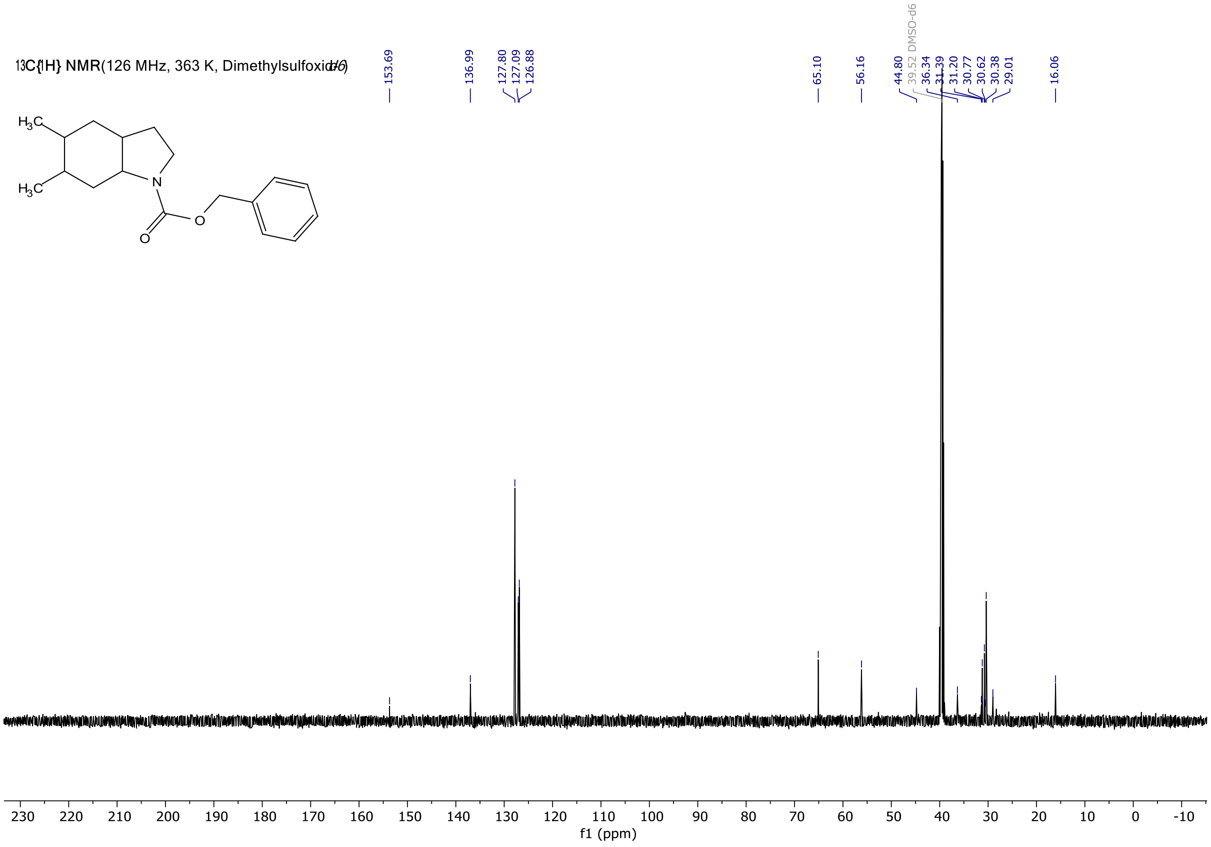

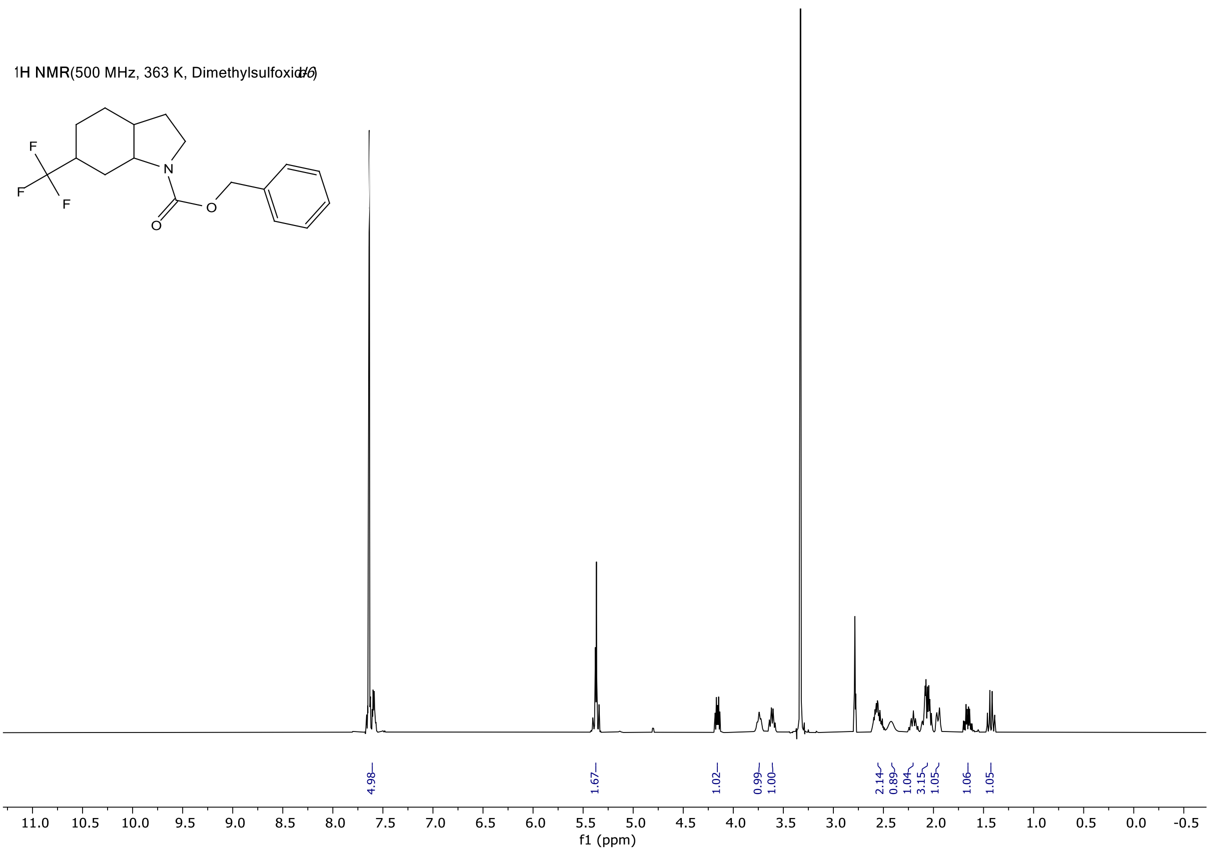
1H NMR(500 MHz, 363 K, Dimethylsulfoxide-d6)
F
F
F
N
O
O
4.98
1.67
1.02
0.99
1.00
2.14
0.89
1.04
3.15
1.05
1.06
1.05
11.0 10.5 10.0 9.5 9.0 8.5 8.0 7.5 7.0 6.5 6.0 5.5 5.0 4.5 4.0 3.5 3.0 2.5 2.0 1.5 1.0 0.5 0.0 -0.5
f1 (ppm)

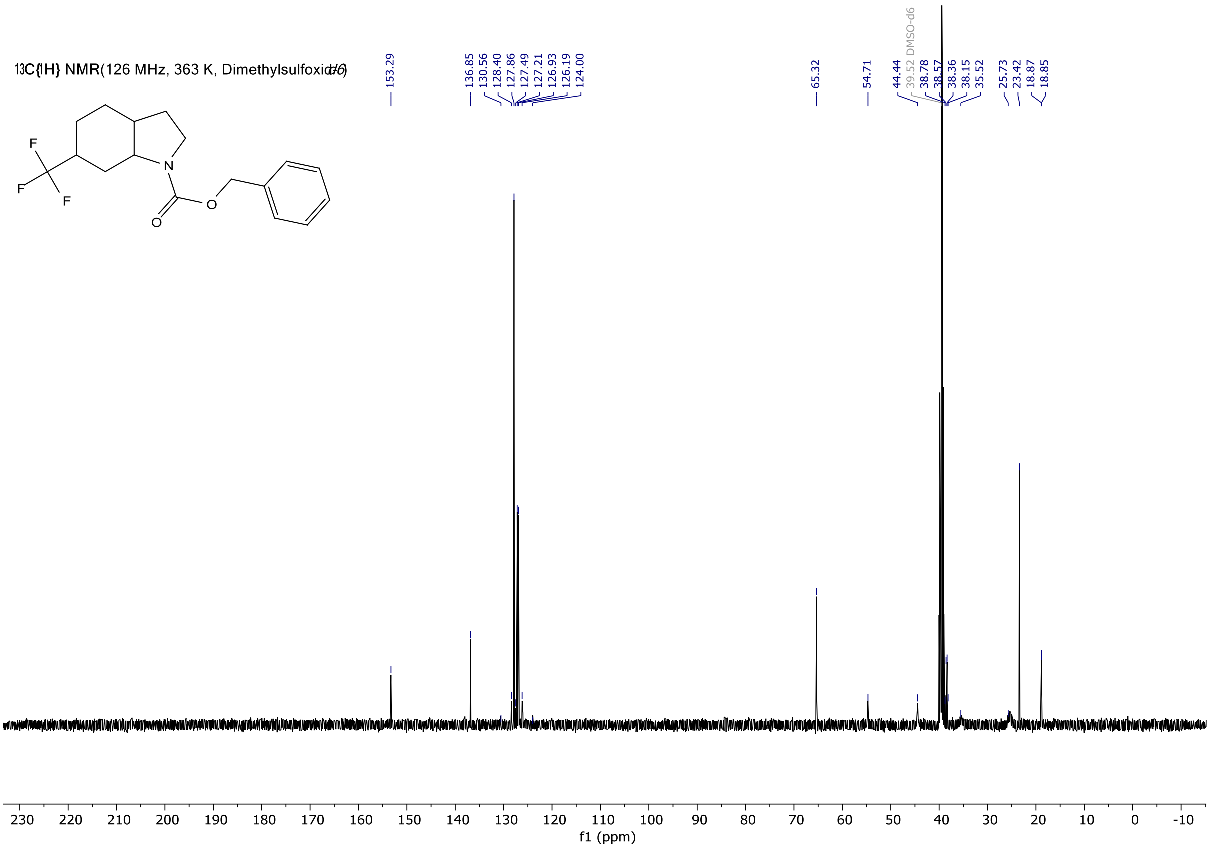
13C{1H} NMR(126 MHz, 363 K, Dimethylsulfoxide-d6)
153.29
136.85
130.56
128.40
127.86
127.49
127.21
126.93
126.19
124.00
65.32
54.71
44.44
39.52 DMSO-d6
38.78
38.57
38.36
38.15
35.52
25.73
23.42
18.87
18.85
F
F
F
N
O
O
230 220 210 200 190 180 170 160 150 140 130 120 110 100 90 80 70 60 50 40 30 20 10 0 -10
f1 (ppm)

19{1H} NMR(470 MHz, Dimethylsulfoxide-*d6*)

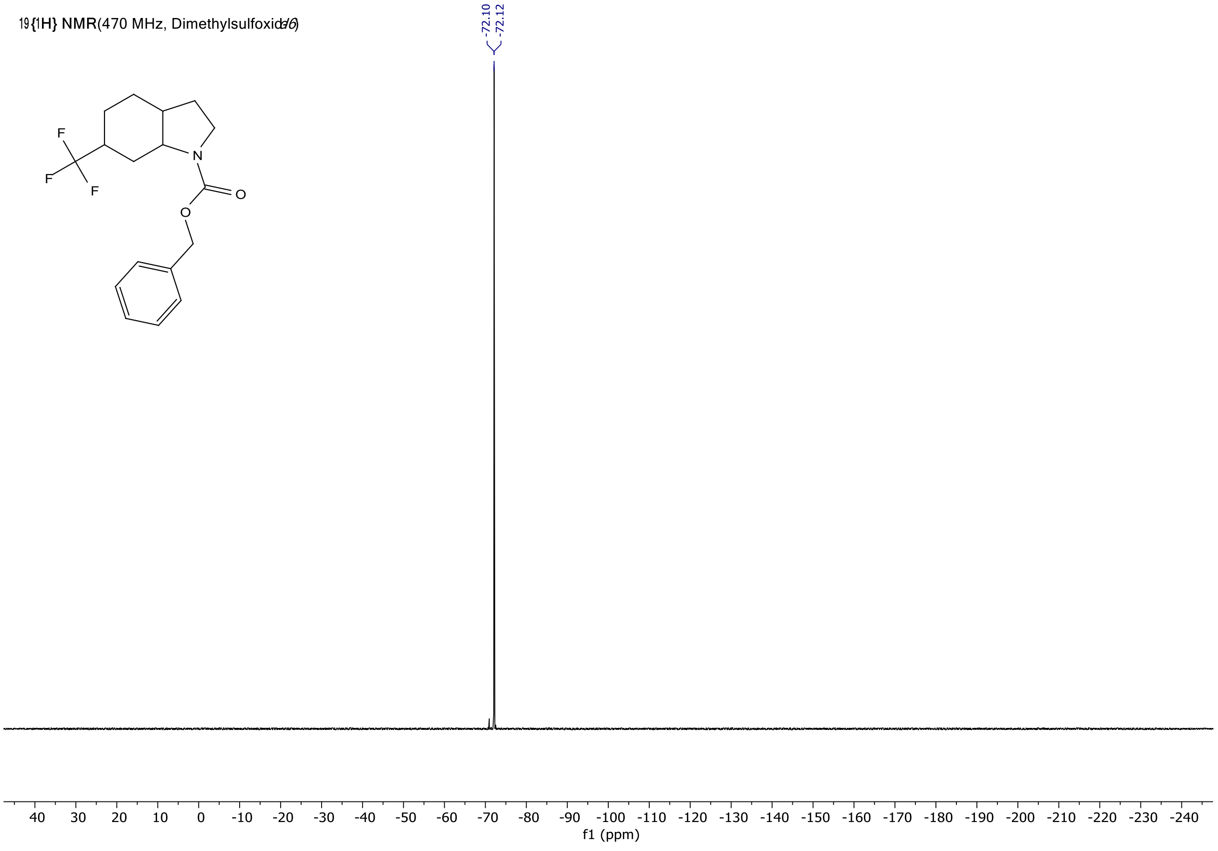

1H NMR(500 MHz, 363 K, Dimethylsulfoxide-*d6*)

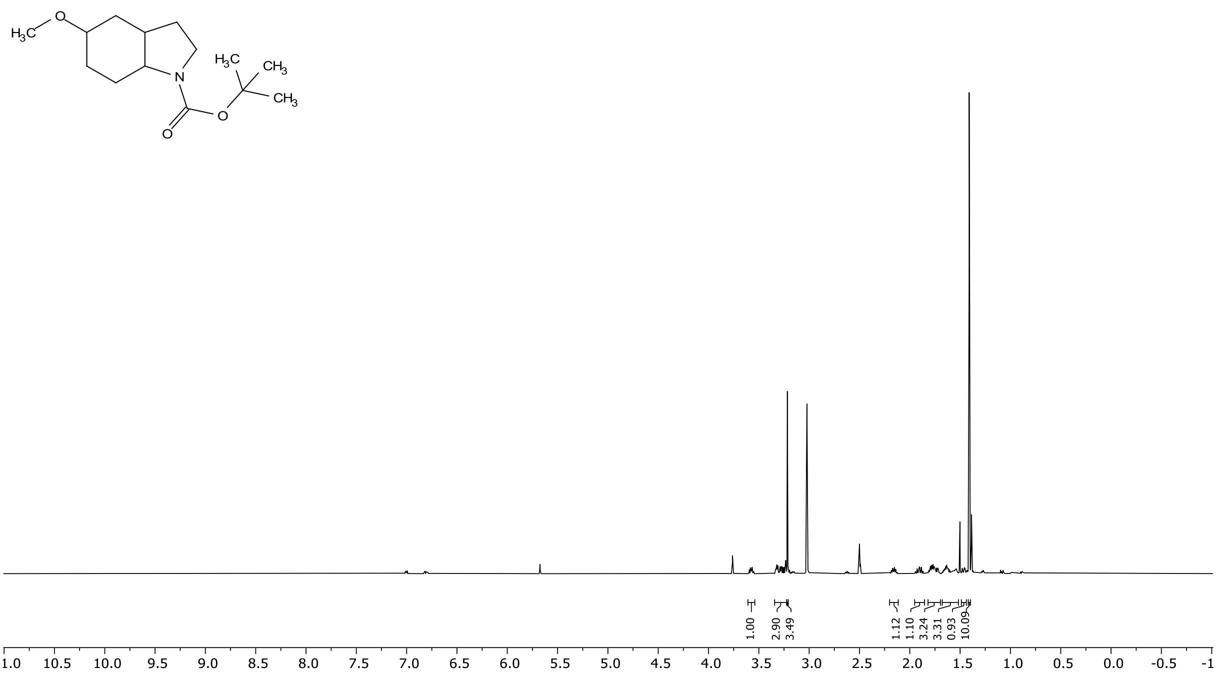

13C{1H} NMR(126 MHz, 363 K, Dimethylsulfoxide-*d6*)

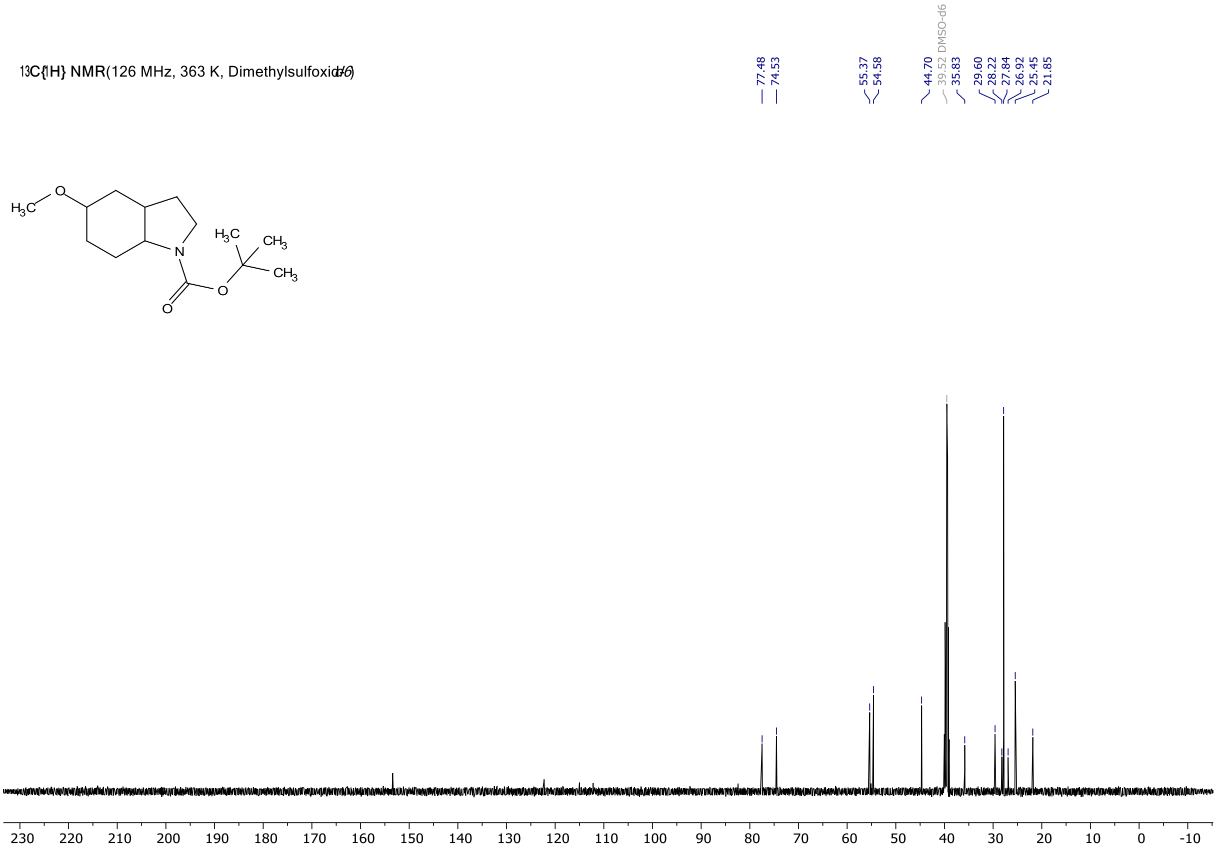

1H NMR(500 MHz, 363 K, Dimethylsulfoxide-d6)

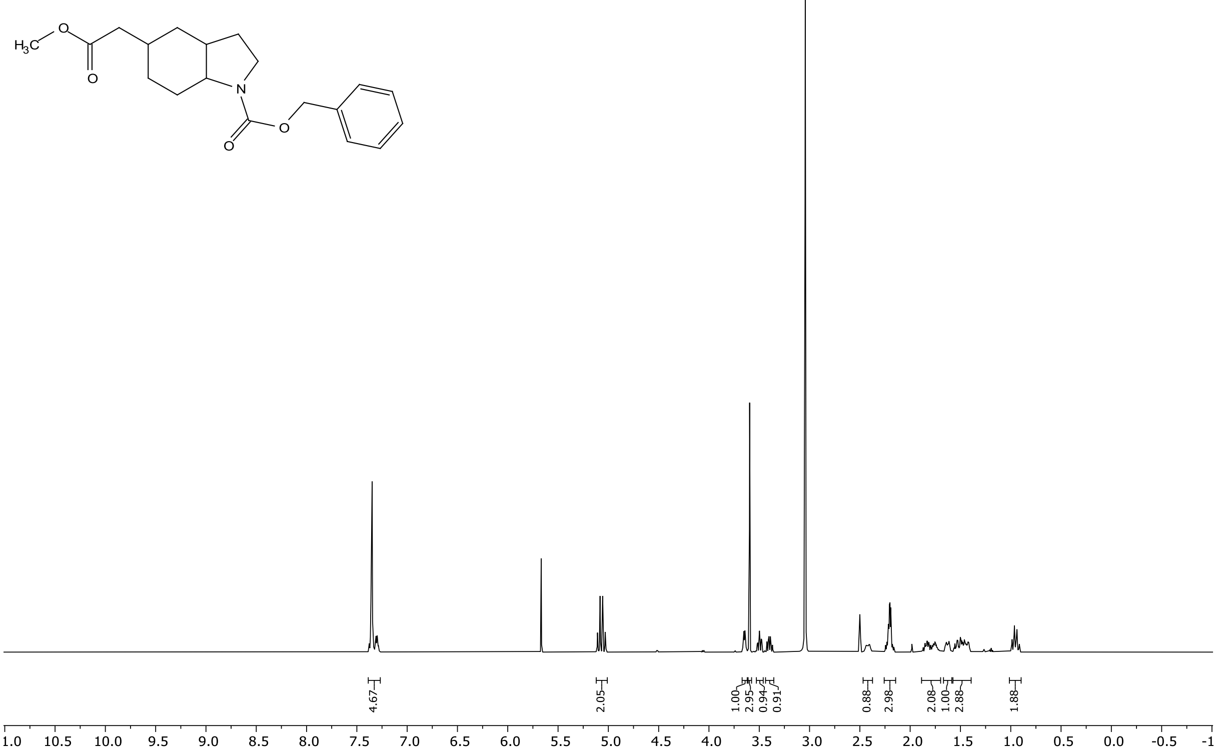

13C{1H} NMR(126 MHz, 363 K, Dimethylsulfoxide-d6)

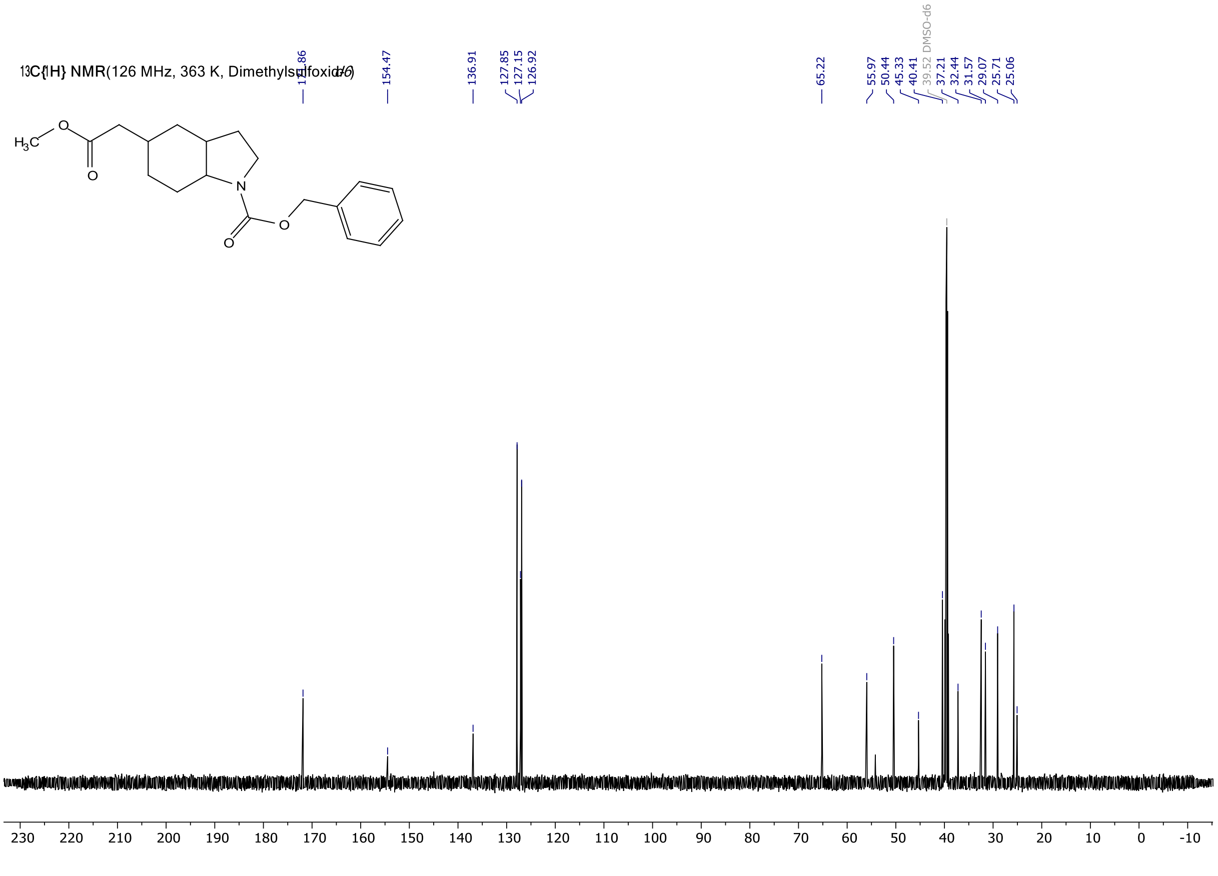

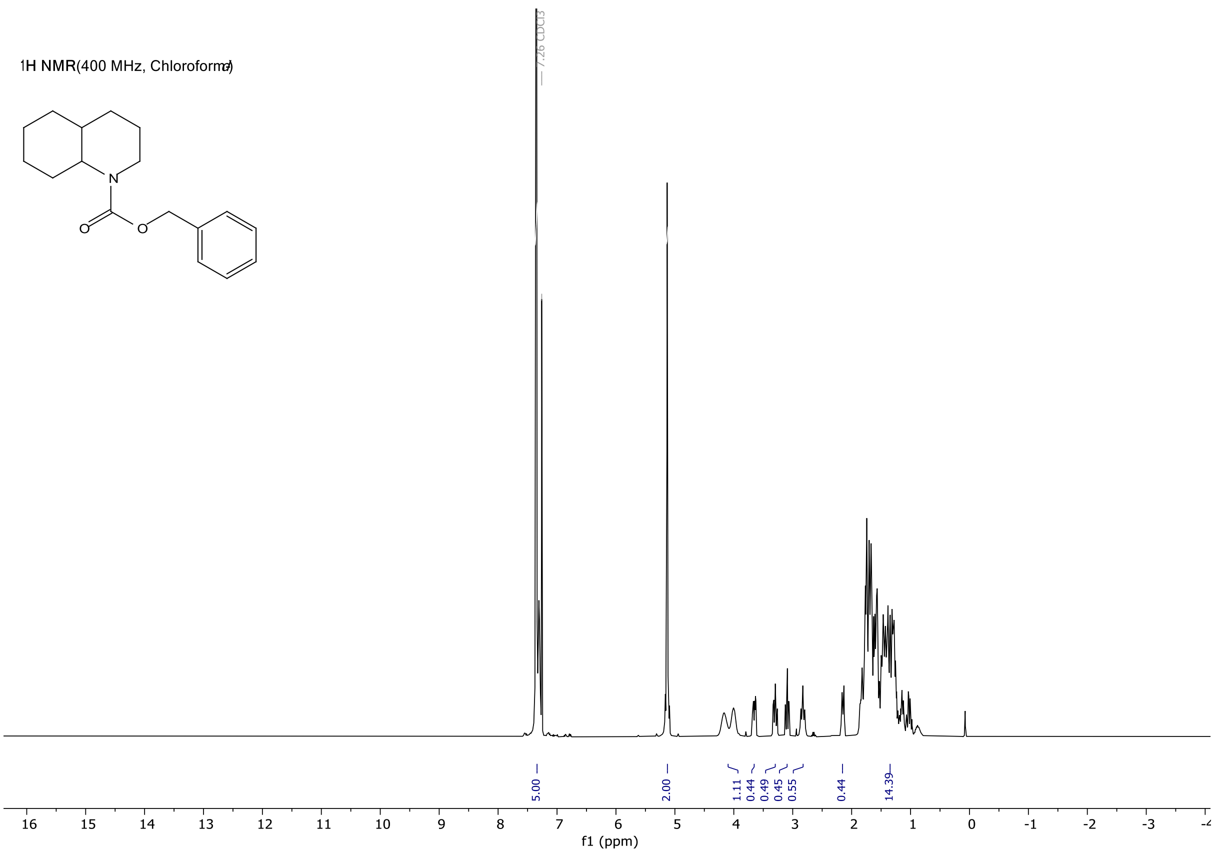
1H NMR(400 MHz, Chloroform-d)
N
O
O
7.26 CDCl3
5.00
2.00
1.11
0.44
0.49
0.45
0.55
0.44
14.39
f1 (ppm)

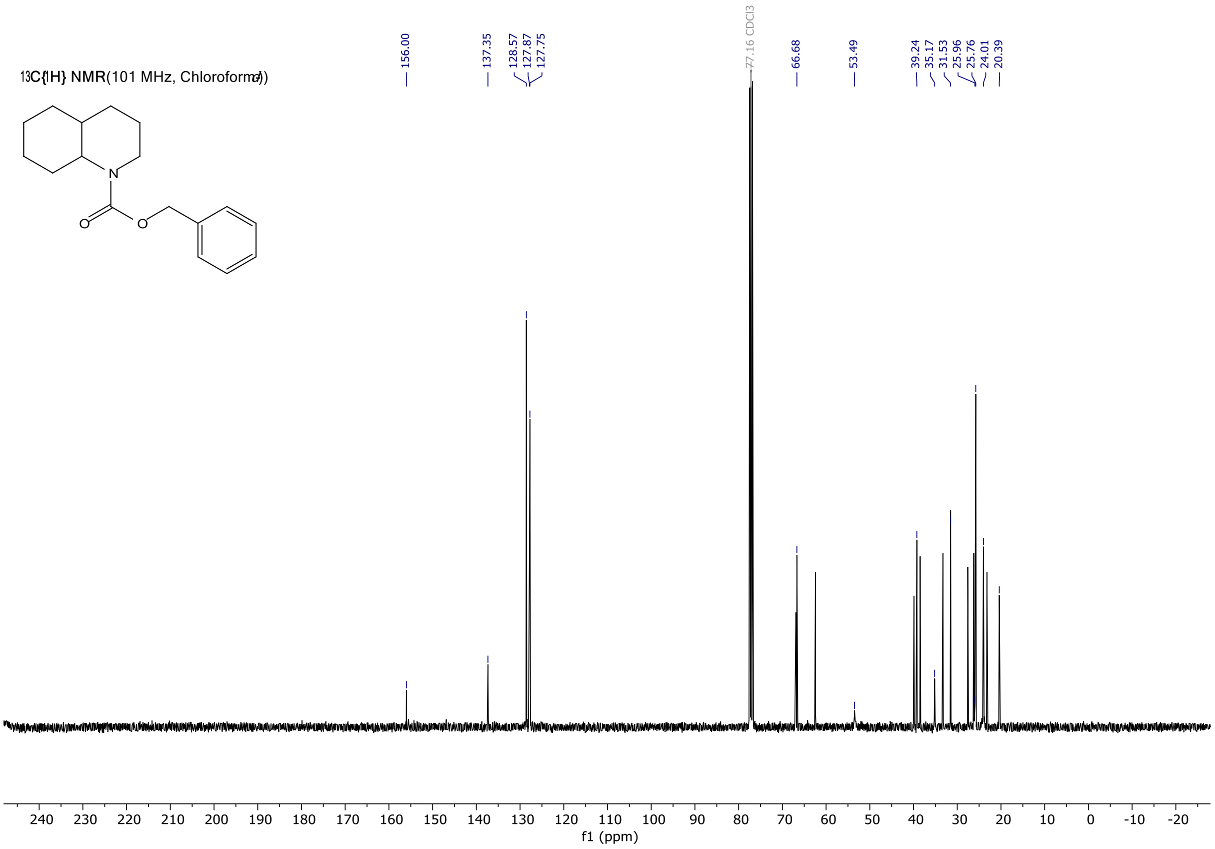
13C{1H} NMR(101 MHz, Chloroform-d)
N
O
O
156.00
137.35
128.57
127.87
127.75
77.16 CDCl3
66.68
53.49
39.24
35.17
31.53
25.96
25.76
24.01
20.39
f1 (ppm)

1H NMR(599 MHz, Chloroform-*d*)

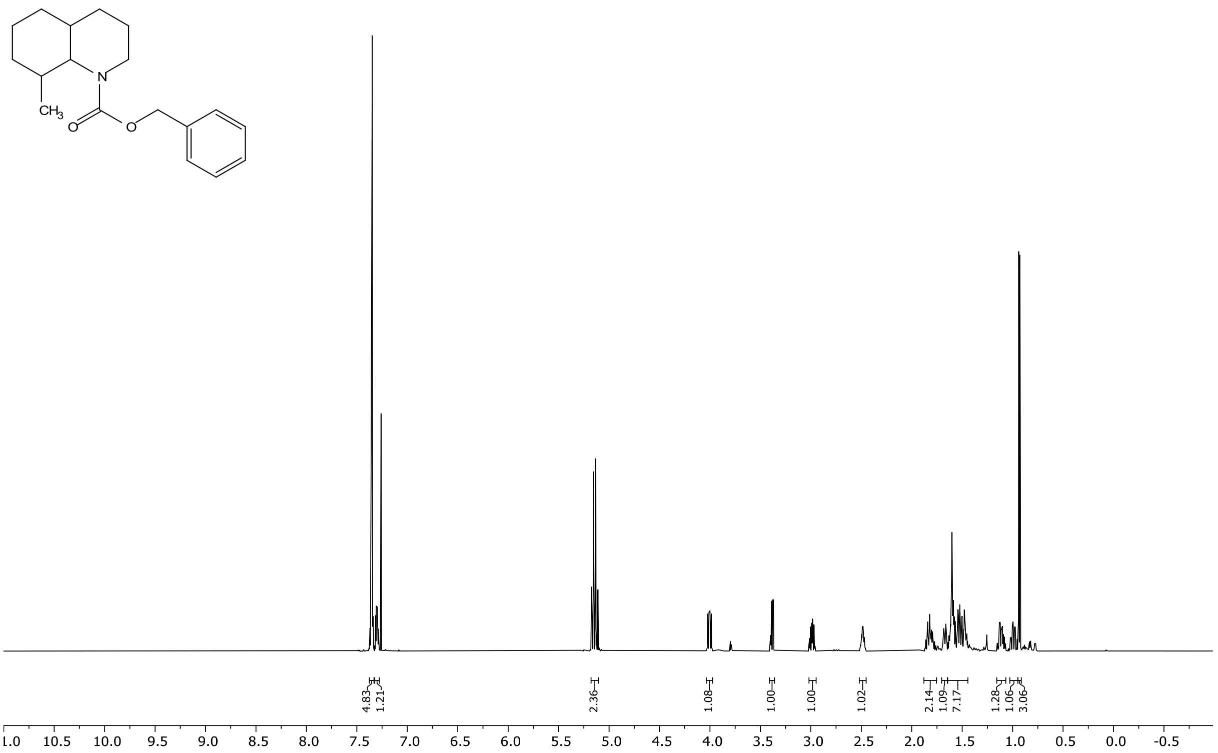

13C{1H} NMR(151 MHz, Chloroform-*d*)

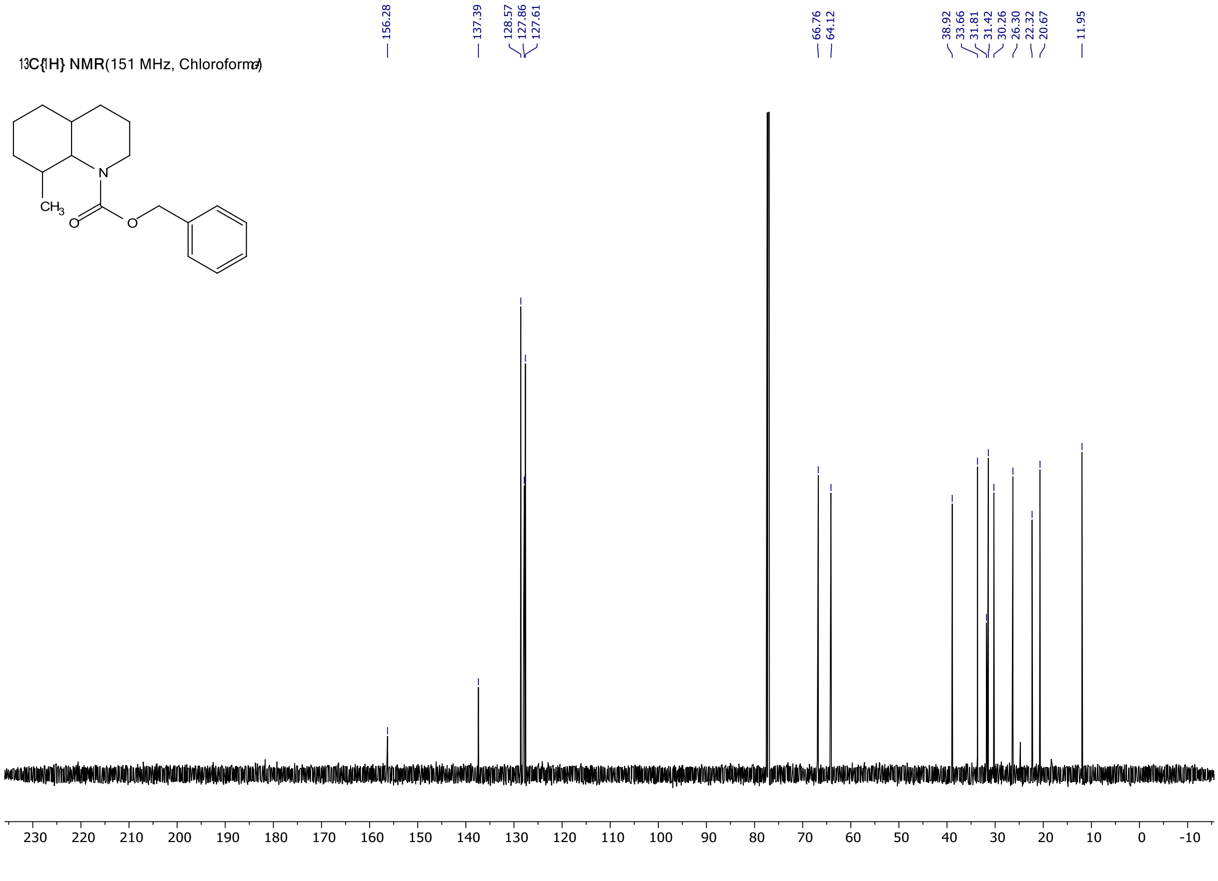

1H NMR(599 MHz, 363 K, Dimethyl sulfoxide-d6)

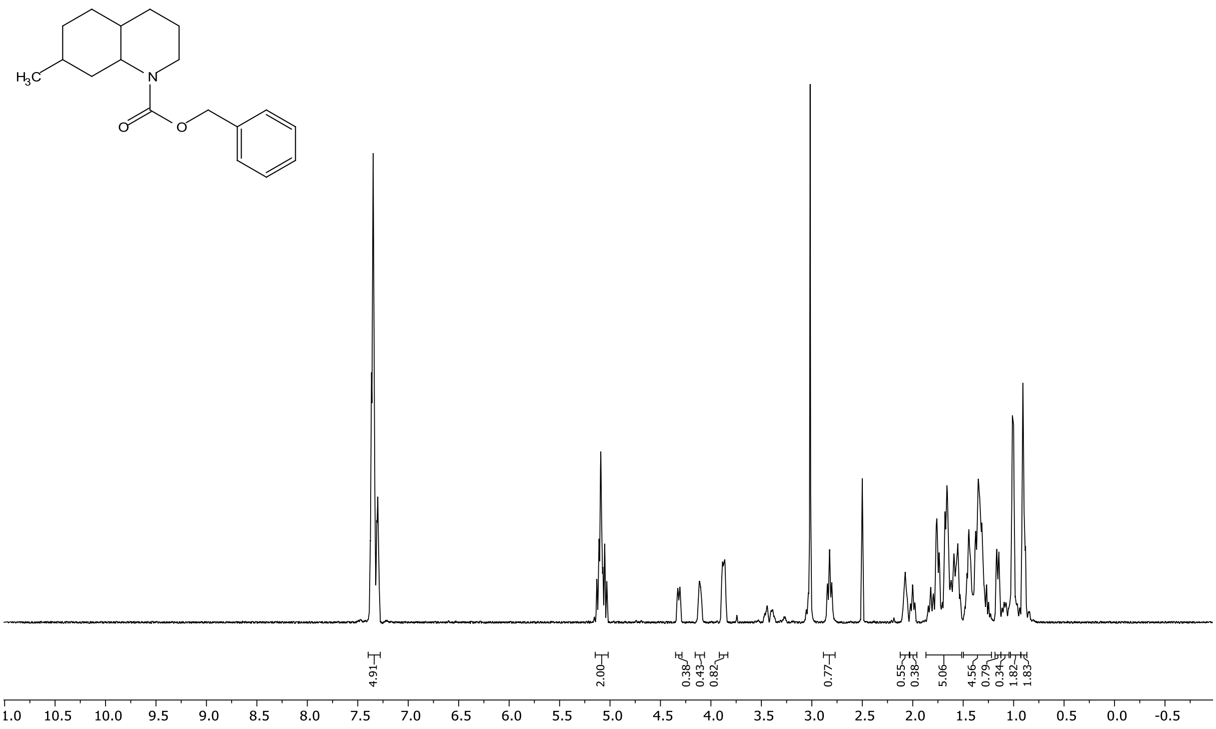

13C{1H} NMR(151 MHz, 363 K, Dimethyl sulfoxide-d6)

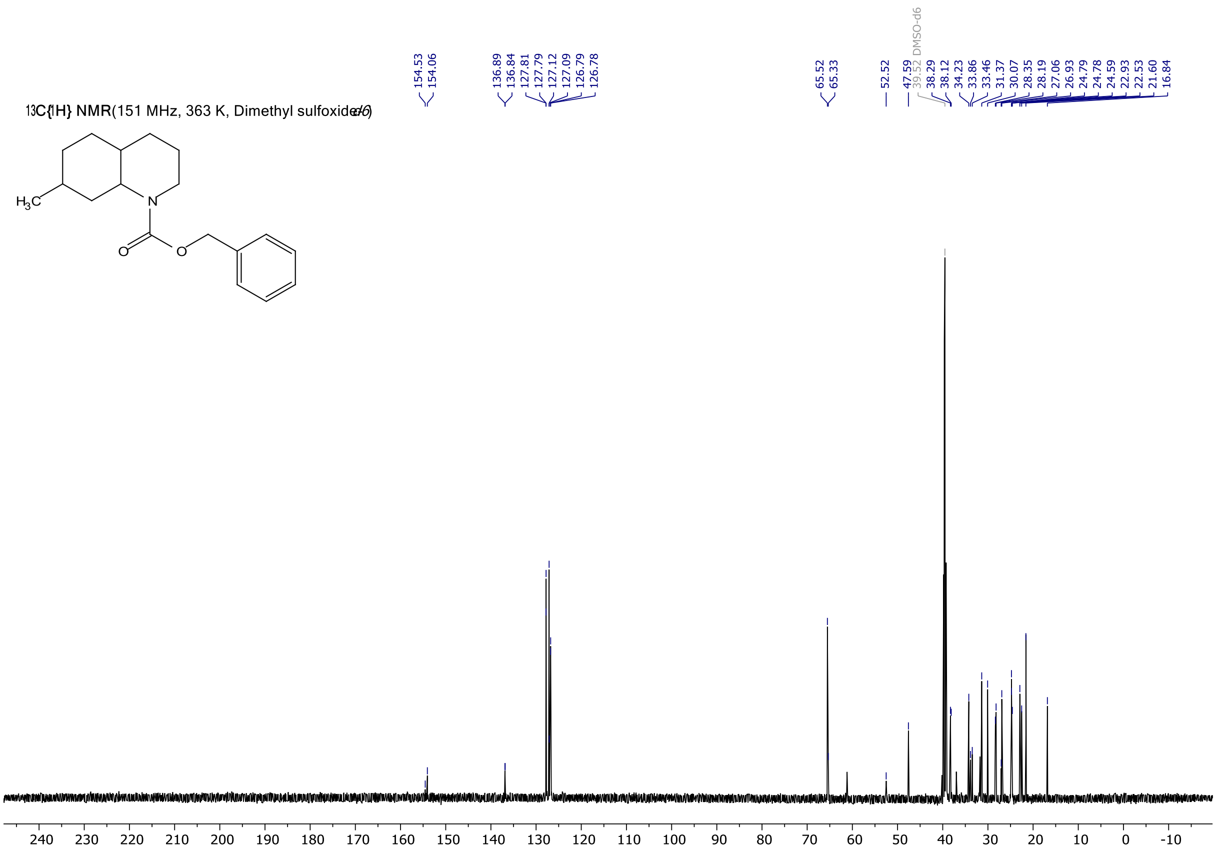

1H NMR(400 MHz, Chloroform-d)

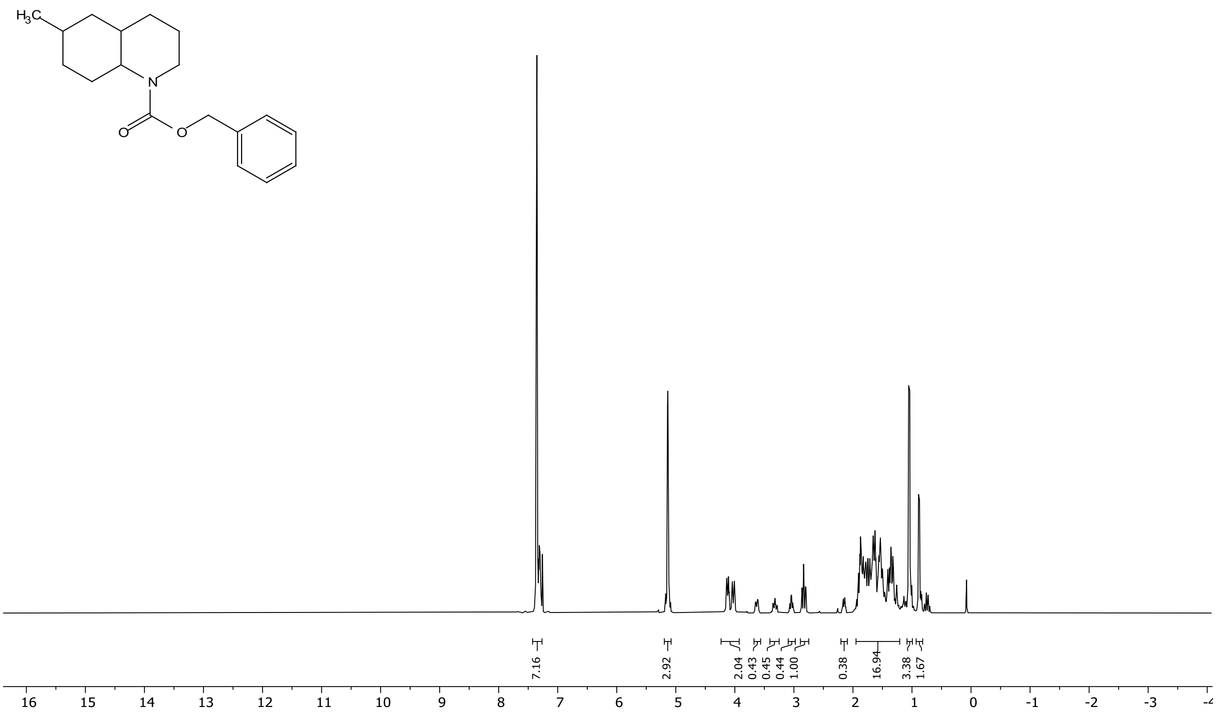

155.61
137.32
128.56
127.91
127.79
66.90
54.00
39.85
37.03
35.13
30.96
27.92
26.30
26.13
21.74
19.73

13C{1H} NMR(126 MHz, Chloroform-d)

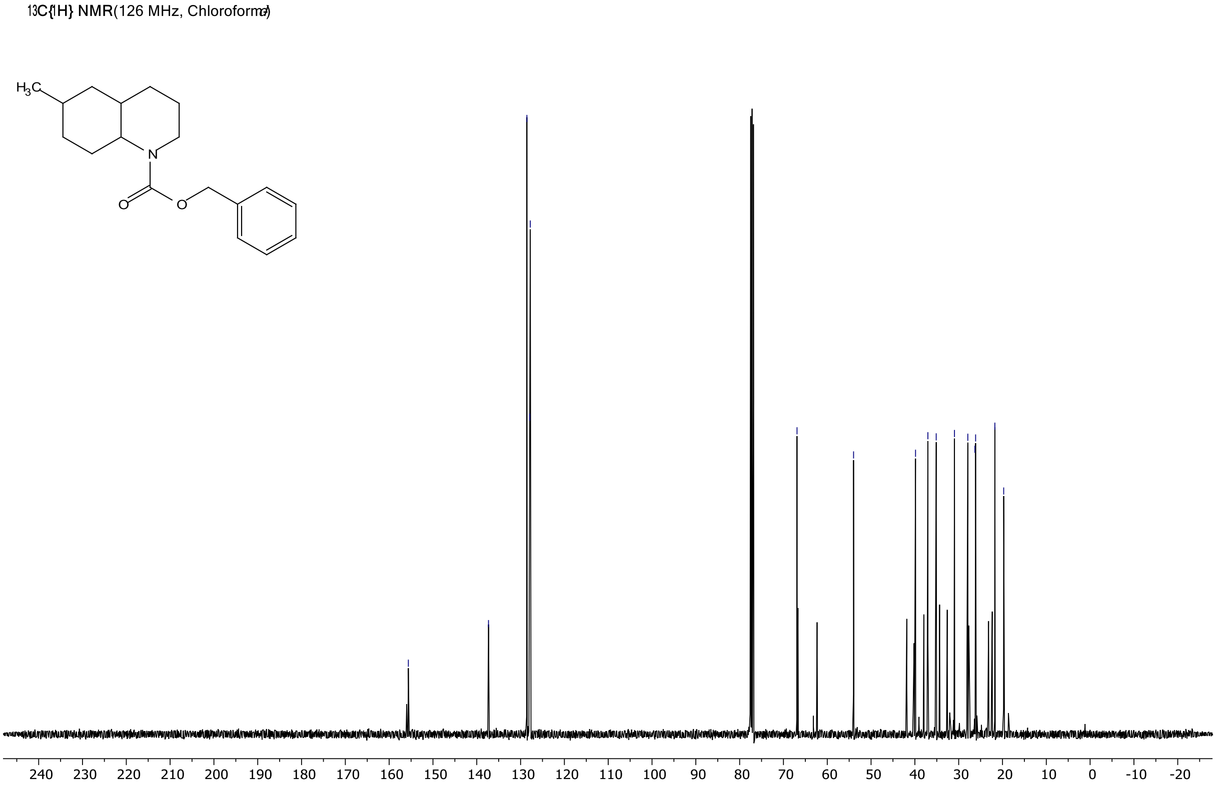

1H NMR(599 MHz, Chloroform-d)

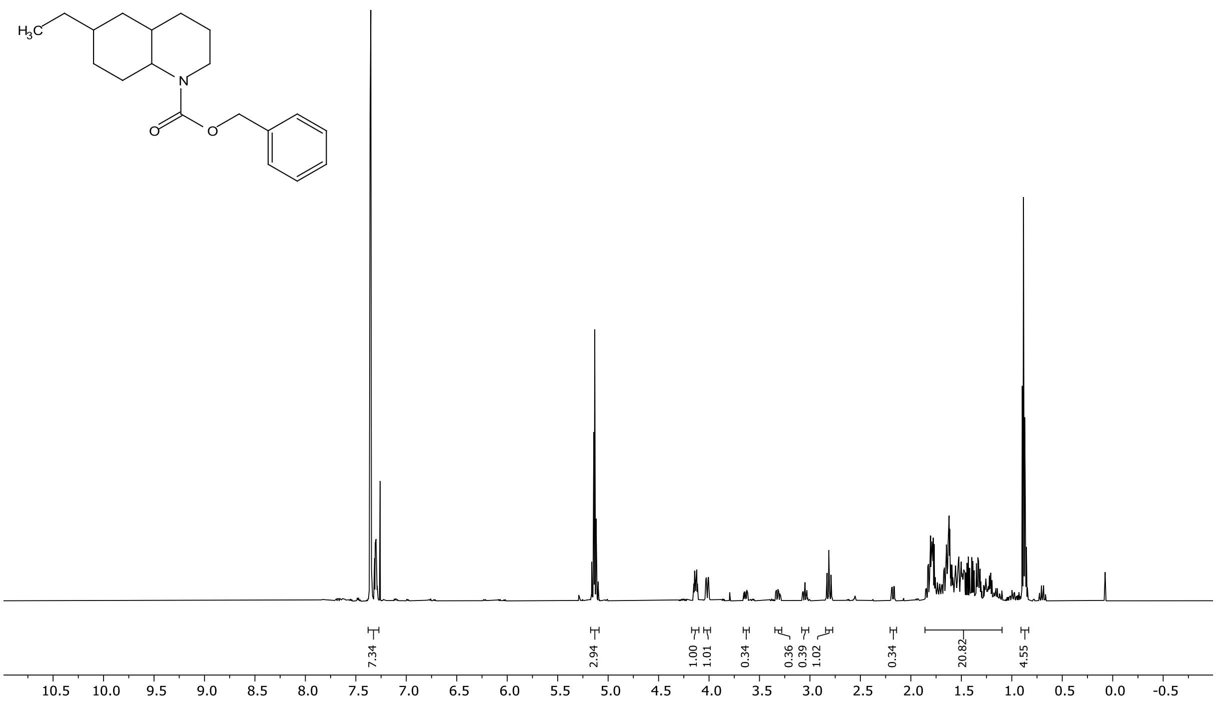

13C{1H} NMR(151 MHz, Chloroform-d)

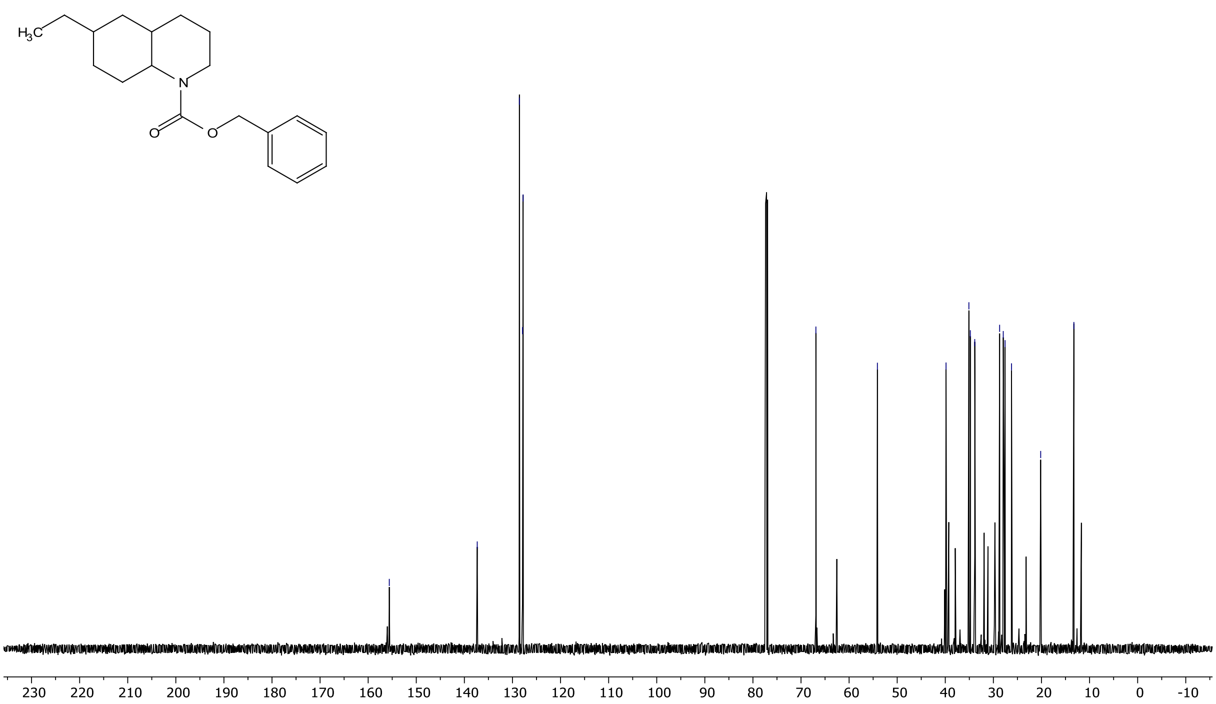

1H NMR(599 MHz, Chloroform-d)

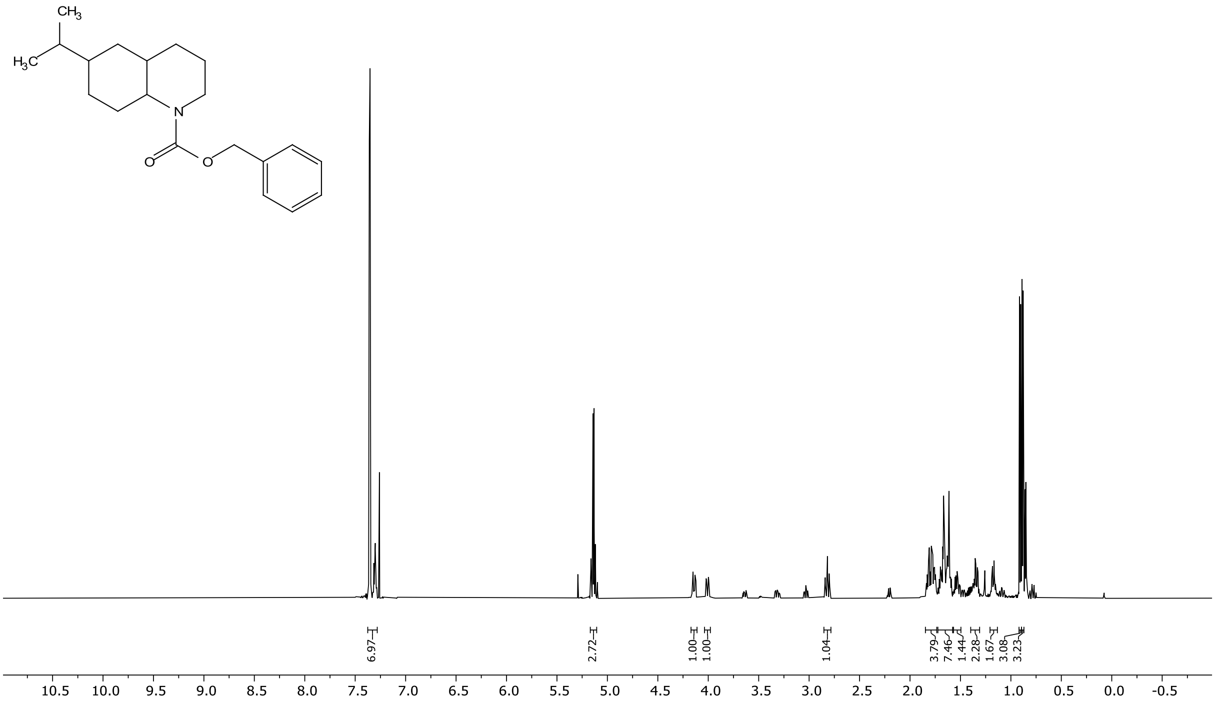

13C{1H} NMR(151 MHz, Chloroform-d)

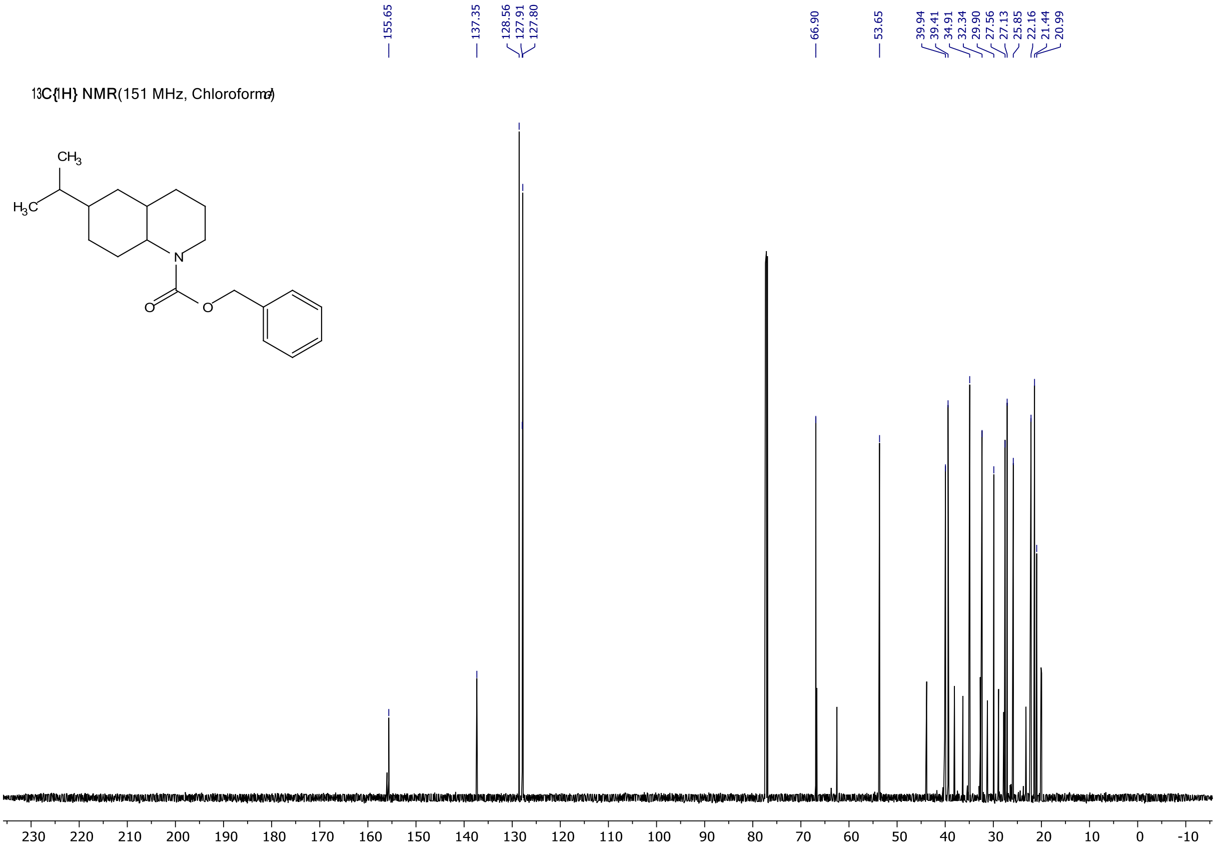

1H NMR(599 MHz, Chloroform-d)

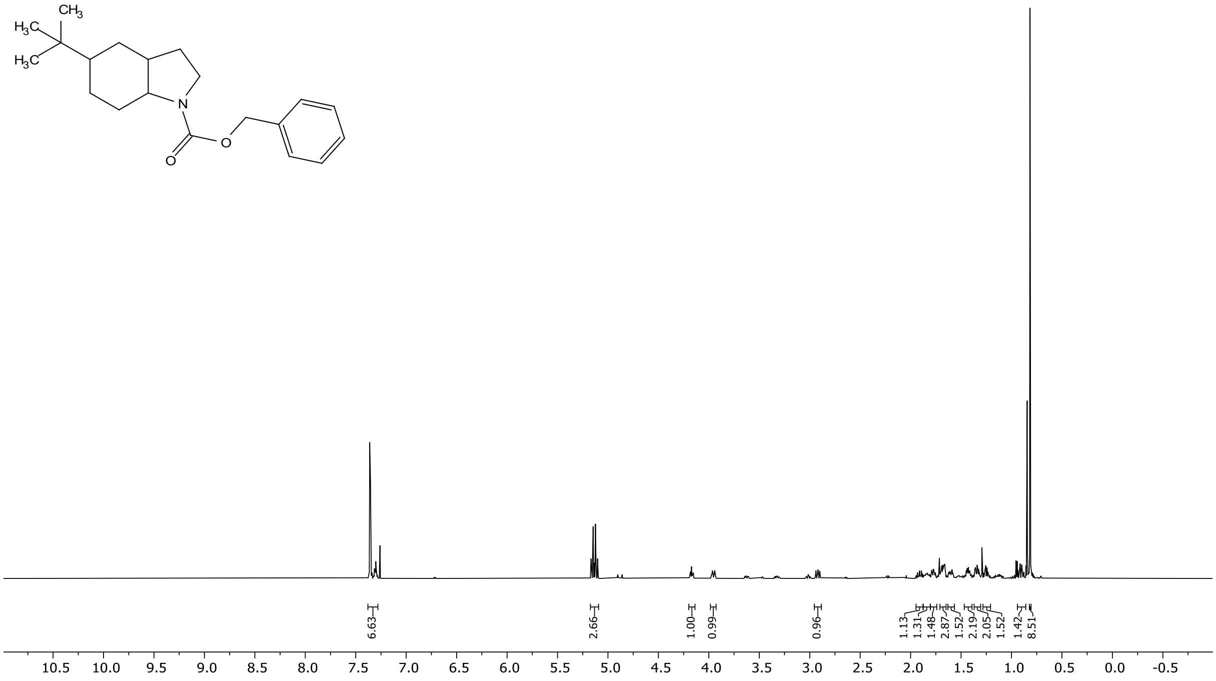

13C{1H} NMR(151 MHz, Chloroform-d)

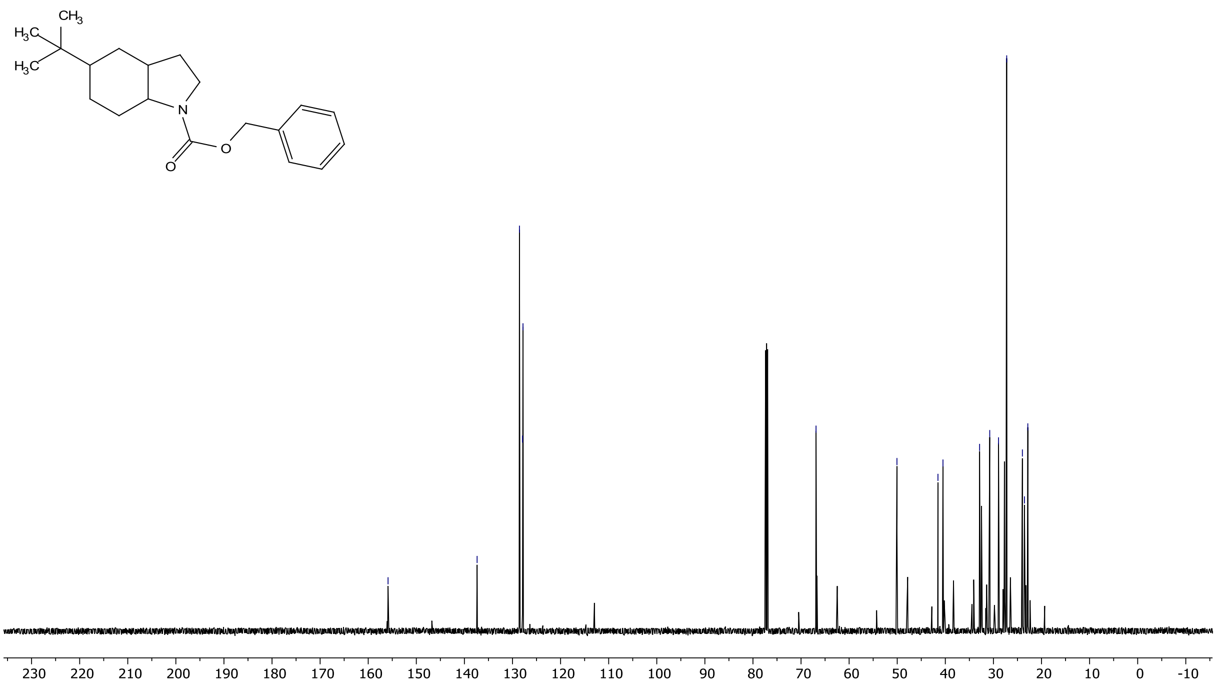

^{1}H NMR(599 MHz, Dimethyl sulfoxide-d_6)

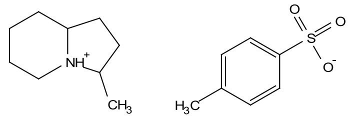

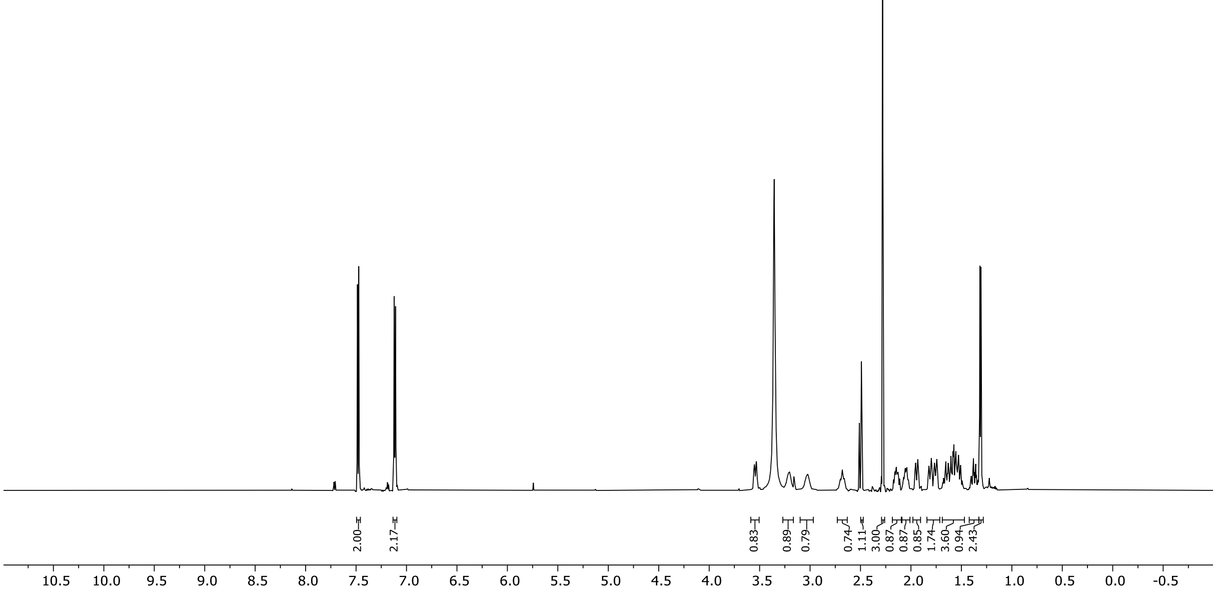

^{13}C{^{1}H} NMR(151 MHz, Dimethyl sulfoxide-d_6)

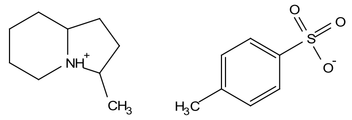

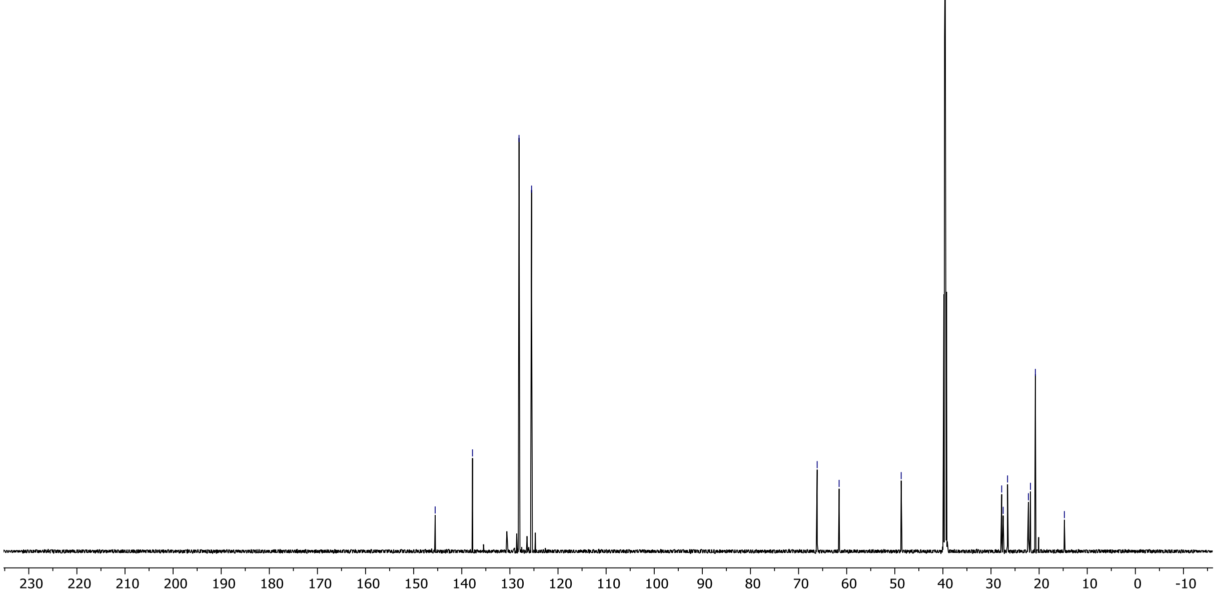

1H NMR(599 MHz, 363 K, Dimethyl sulfoxide-d6)

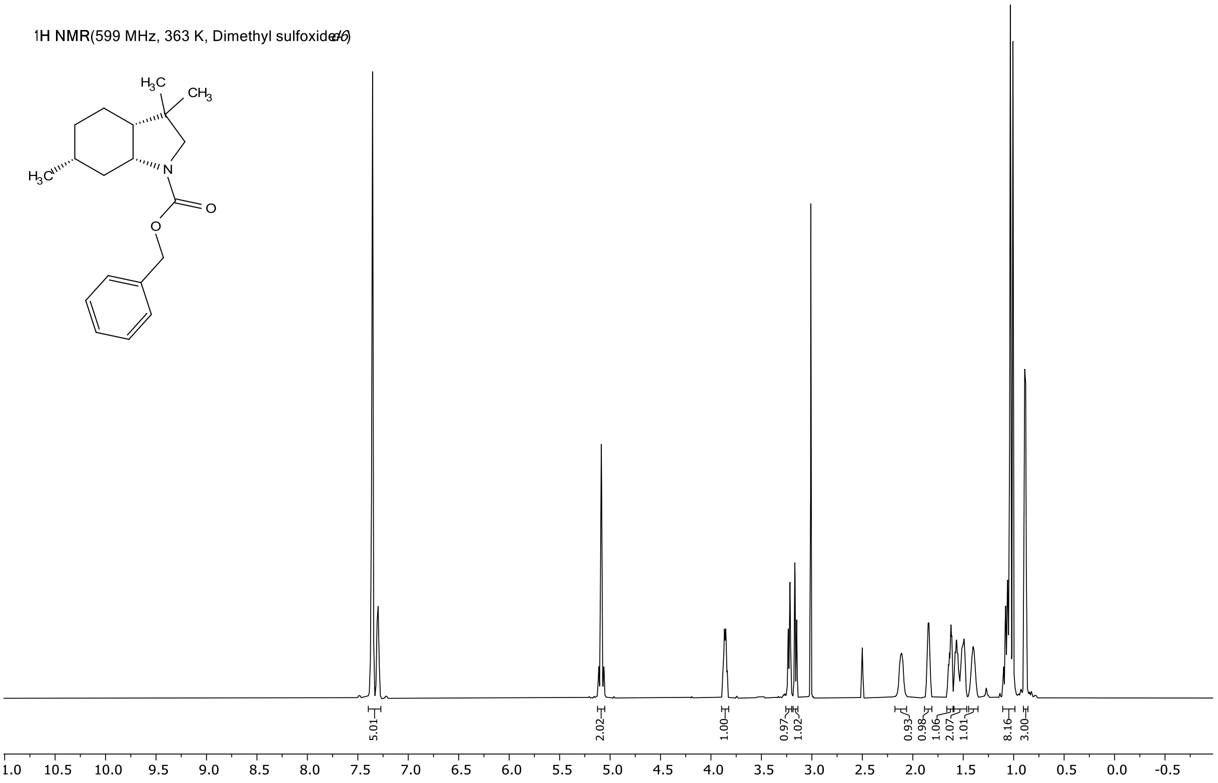

13C{1H} NMR(151 MHz, 363 K, Dimethyl sulfoxide-d6)

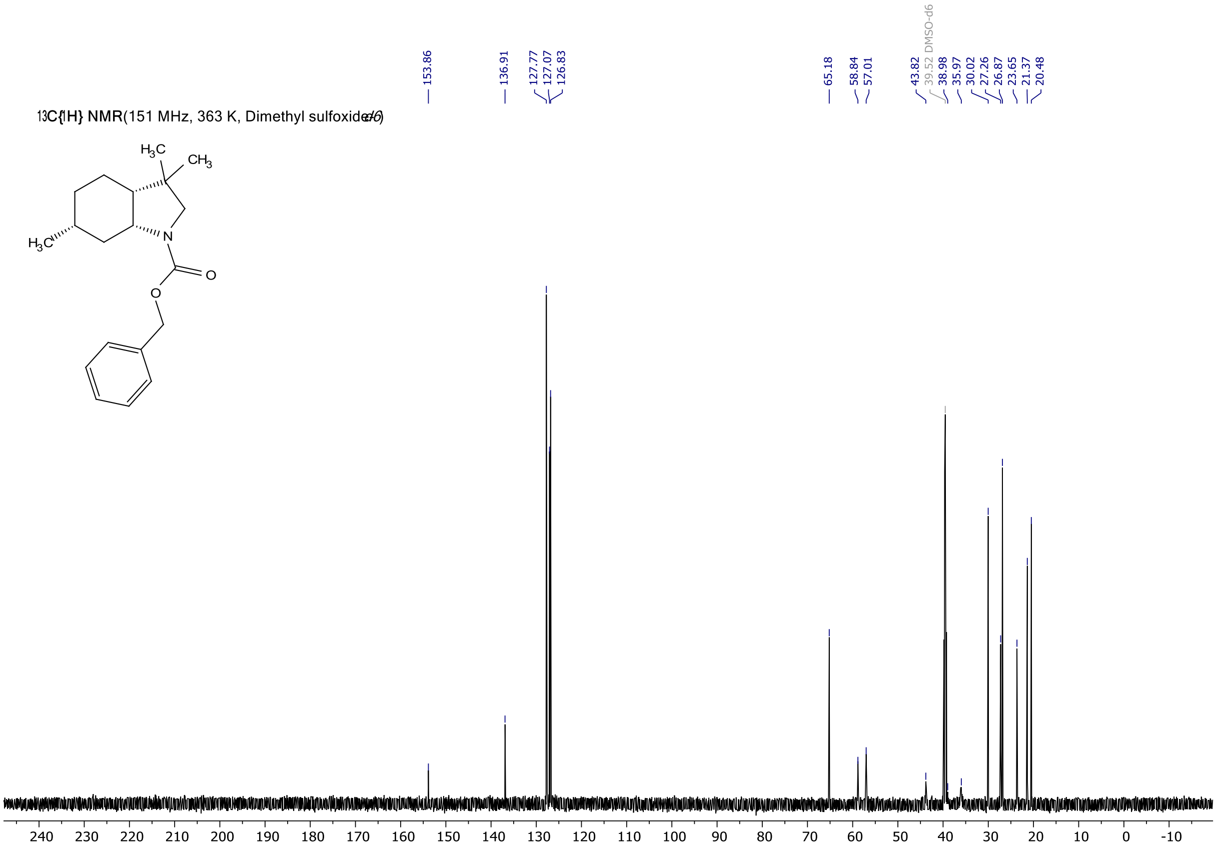

Supplement: Supplementary file 1 — Supporting Information [file ADSC-364-3366-s001.pdf]
